# Sociodemographic characteristics and reproductive health factors associated with maternal knowledge and practice of infection prevention in neonates in North Dayi District, Ghana

**DOI:** 10.3389/fpubh.2023.1062268

**Published:** 2023-04-17

**Authors:** Lawrence Sena Tuglo, Benjamin Adu Agyekum, Edward Delali Darku, Natabou Morine Alida, Kitso Palesa Seelo, Khauhelo Magaga, Kudzai Victor Chiambiro, Jessica Dzigbordi Tuglo, Sylvia Mawusinu Sakre, Desmond Dzidzornu Otoo, Jonathan Mawutor Gmanyami

**Affiliations:** ^1^Department of Nutrition and Dietetics, School of Allied Health Sciences, University of Health and Allied Sciences, Ho, Ghana; ^2^Department of Dentistry and Surgery, School of Medicine, Nantong University, Nantong, China; ^3^Department of Clinical Medicine, School of Medicine, Fujian Medical University, Fuzhou, China; ^4^Department of Pharmaceutics, School of Pharmacy, Nantong University, Nantong, China; ^5^Department of Midwifery, School of Nursing and Midwifery, University of Health and Allied Sciences, Ho, Ghana; ^6^Department of Community Health Nursing, Nurses Training College, Ho, Ghana; ^7^Department of Public Administration and Health Services Management, School of Business, University of Ghana, Accra, Ghana; ^8^Global Health and Infectious Diseases Group, Kumasi Centre for Collaborative Research in Tropical Medicine, Kumasi, Ghana

**Keywords:** sociodemographic characteristics, reproductive health factors, knowledge, practice, infection prevention in neonates, mothers, Ghana

## Abstract

**Background:**

Neonates are at a greater risk of infection, but data on the maternal knowledge and practice of infection prevention in neonates (IPNs) are scarce. This study aimed to assess sociodemographic characteristics and reproductive health factors associated with maternal knowledge and practice of IPNs in North Dayi District, Ghana.

**Methods:**

This was a multicenter cross-sectional study conducted among 612 mothers. A structured questionnaire was used for data collection adapted from previous studies and the IPN guidelines of the World Health Organization (WHO). Bivariable analyses were performed to determine the association between maternal knowledge and practice of IPNs and sociodemographic characteristics and reproductive health factors.

**Results:**

Analysis showed that less than one-fifth of the mothers (12.9%) had poor knowledge of IPNs, while 21.6% incorrectly practiced it. Mothers who had poor knowledge of IPNs [adjusted odds ratio (AOR) = 13.33 (95% CI: 7.69–23.26), *p* < 0.001] were more likely to have a poor practice of IPNs.

**Conclusion:**

About one-fifth of the mothers in this study had poor knowledge or poor practice of IPNs according to the WHO’s guidelines. The Health Directorate of North Dayi District should explore the risk factors associated with poor IPNs and increase successful guideline adherence with intensified educational outreach and campaigns.

## Introduction

Globally, the WHO report of 2020 showed that an estimated 2.4 million neonatal deaths occurred in the first month of newborn life (1). Of these, 43% occurred in developing countries, mostly in Sub-Saharan Africa (SSA), followed by 36% in central and southern Asia (1). Neonatal infection (35.8%) is the major cause, followed by prematurity (27.5%) and other birth complications (22.9%) during the neonatal period (2, 3). Neonatal infection refers to sepsis involving the bloodstream in newborns less than 28 days old (1, 4). Without a substantial reduction in infection-related neonatal deaths in developing countries, it is questionable that the Sustainable Development Goal (SDG), which targets the reduction of neonatal mortality to at least 12 per 1,000 live births by 2030, will be met (5).

The WHO recommends the following measures to prevent neonatal death: ANC visits (a minimum of eight), skilled delivery, and PNC visits (a minimum of three) (1, 2, 4). Other recommendations of the WHO include immunization against tetanus, education on neonatal danger signs and skilled care such as thermal care, cord care, breastfeeding, and bathing of neonates (1, 2, 4). Additionally, the WHO endorses that babies who are delivered at home should be visited by healthcare professionals on the day of birth (1, 2). Studies have shown that home deliveries may pose serious complications for babies because of the traditional care they receive from relatives and traditional birth attendants (6–8).

Despite this, the high incidence of neonatal death can be prevented by proper infection prevention in neonates (IPNs) (1). IPNs refer to a comprehensive approach intended to improve the health of neonates from pregnancy through childbirth and the postnatal phase (4). Studies have found an association between the knowledge of mothers (2, 4–6, 9–13) and the practice of mothers (2, 7–9, 14–17) and IPNs. Other studies have shown sociodemographic characteristics (mother age (14, 18), marital status (8) educational status (6–8, 11, 16, 19), income level (6, 9, 15, 16), employment status (2, 5, 8, 10), type of residence (4, 8, 20) and access to media (19, 20)) and reproductive health factors [parity (9, 10, 12, 15, 16, 20), number of ANC visits (2, 4, 9–11, 15, 19), place of delivery (2, 9, 15), number of PNC visits (2, 4, 6, 9, 19), and walking distance (9)] to be associated with IPNs.

In Ghana, despite the extra effort and measures adopted to improve IPNs, the status among mothers is still a major concern (5, 14). Studies performed in Ghana have found poor knowledge (5), poor practice (14), home delivery (2), and less than minimum attendance of ANC and PNC according to WHO recommendations (2) as major concerns of IPNs. In the North Dayi District, the levels and factors associated with IPNs had not been studied, and no studies were found for comparison, despite the steady rise of neonatal deaths attributed to infection in the district. It is necessary to conduct a study in which the findings will add to the literature and serve as useful information for educating mothers on IPNs to reduce the rates of neonatal mortality in North Dayi District and beyond. Hence, this study aimed to assess the sociodemographic characteristics and reproductive health factors associated with maternal knowledge and practice of IPNs in North Dayi District, Ghana.

## Materials and methods

### Study design and setting

**Figure 1:**
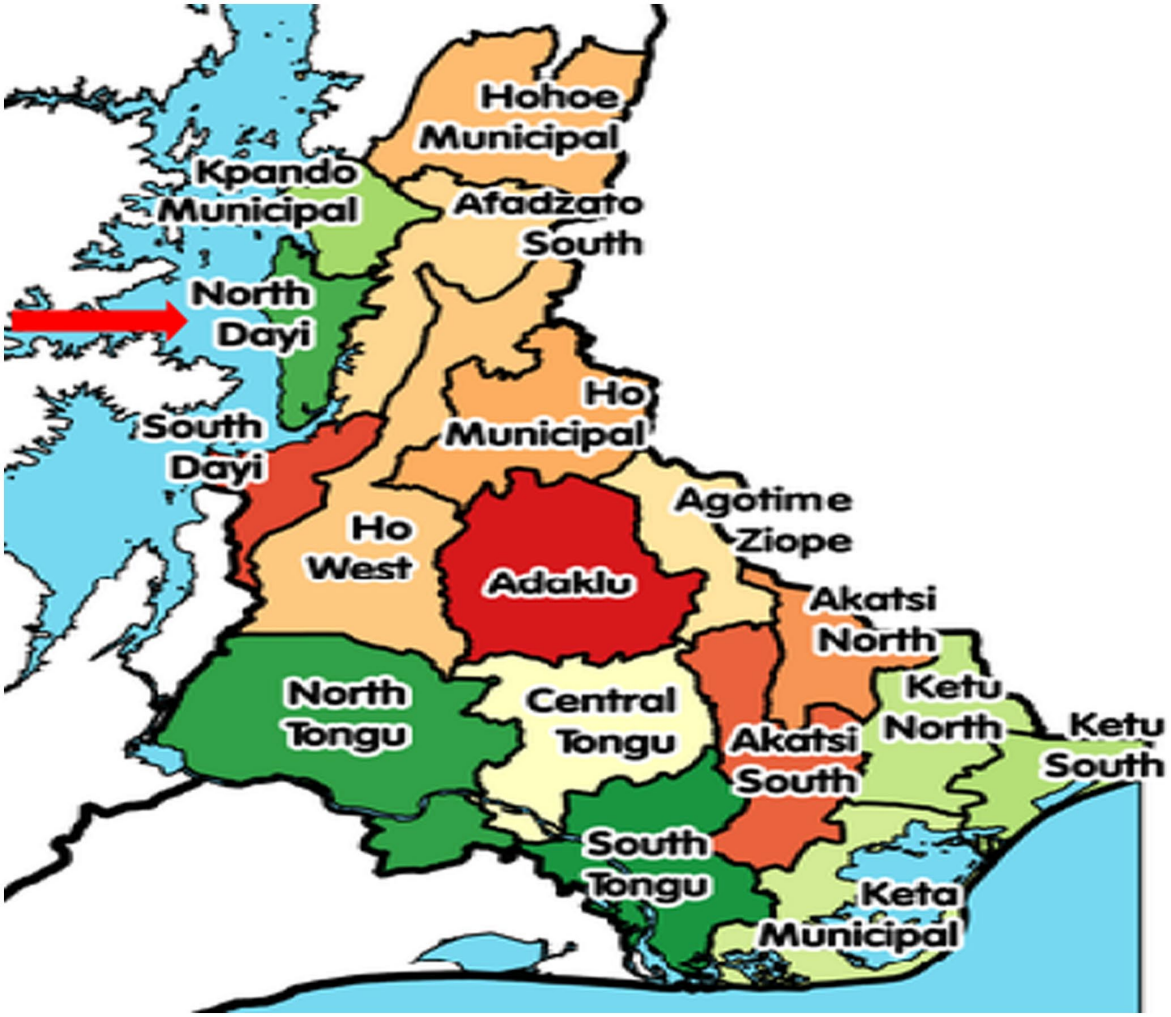
Map showing the North Dayi district where the study was conducted in the Volta Region, Ghana.

A multicenter cross-sectional study was conducted between June 2020 and August 2020 in North Dayi District, Ghana. The North Dayi District is one of the 261 Metropolitan, Municipal and District Assemblies (MMDAs) in Ghana and forms part of the 18 municipalities and districts in the Volta Region. The North Dayi District was established by the Legislative Instrument (LI) 2076 of 2012. Its capital is Anfoega. It borders Kpando municipality to the north, South Dayi District to the south, and Afazato South District to the east (21). The total population is 39,913, representing 46.7% males and 53.3% females. The villages include Anfoega, Vakpo, Wusuta, Tsrukpe, Botoku, Tsoxor, Awate, Aveme, and Tsyome Sabadu. The study design provides an accurate report of IPNs among mothers through the analysis and interpretation of the questionnaire-based survey. Additionally, it is suitable, reduces cost and saves time for conducting the study (Figure 1).

### Eligibility criteria

Included were mothers who visited the various facilities with babies aged less than a year and consented to participate in the study. The reason for the inclusion of mothers having babies aged less than a year was based on the 2020 WHO report that babies who die within the first 28 days of birth suffer from conditions associated with a lack of quality care (1). Excluded were mothers who visited the various facilities with babies aged at least a year or above, those unable to communicate properly and those who disagreed to take part in the study.

### Study population and sample size

Mothers who visited the various facilities had babies who were less than a year old. On average, these are the numbers of mothers who visited the various facilities during the study period. Aveme Danyigba Health Centre (*n* = 187), Tsyome Sabadu Health Centre (n = 190), Awate Health Centre (*n* = 205), Tsrukpe Health Centre (*n* = 194), Botoku Health Centre (n = 180), Vakpo Health Centre (*n* = 210) and Wusuta Health Centre (*n* = 185). In 1 month, the total monthly attendance was 1,351. Yamane’s formula *n* = *N*/1 + *N*(e^2^) (22) was used to calculate the sample size, where *n* = sample size, *N* = population size, e = margin of error (5%), plus 10% nonresponse, and an estimated sample size of 339 was obtained. This sample size was distributed proportionally across the various facilities to avoid bias during the data collection.

### Sampling technique and procedure

Data collection was performed systematically to reduce the effects of bias. Thus, the study population was divided by the estimated sample size, *r* = *N*/*n* = 1351/339 = 3.985, to obtain a fraction that was approximated to four. In every facility, the first mother and, sequentially, every fifth mother thereafter were sampled to participate in the study. The data collection commenced with the first through to the fifth mother at the various facilities. It continued until the last mother in attendance was served. A total of 659 questionnaires were administered face-to-face, with 612 (92.9%) fully answered and used for the analysis.

### Data collection tool

A structured questionnaire was designed and divided into three sections. It was adapted from previous studies (2, 4, 6, 7, 9–11, 18). The questions on the knowledge and practice of IPNs were also adapted from previous studies (2, 4, 6, 7, 9–11, 18) and standardized according to the IPN guidelines of the WHO (23, 24). Section 1: sociodemographic characteristics (mother’s age, marital status, educational status and income level), reproductive health factors (parity, number of ANC visits, gestational age, place of last delivery, baby’s condition after birth and number of PNC visits). Section 2: Knowledge of IPNs. Section 3: Practice of IPNs.

### Assessment of knowledge and practice of infection prevention In neonates

These sections of the questionnaire comprised 20 closed-ended questions. Ten questions for each on knowledge of IPNs with three responses (“True,” False,” and “Do not know”) and on the practice of IPNs with three answers (“Yes,” “No,” and “Not sure”). The correct answer on knowledge or practice of IPNs was given 1 point, and the incorrect answer was awarded 0 points (including “Do not know” or “Not sure”). The “do not know” and “not sure” responses were included to avoid biases in answering the questions (25). A cumulative percentage method of assessment was used to score the answers and rounded up to 100%. Cumulative scores of <70% of the correct answers were considered “poor,” and cumulative scores of ≥70% were considered “good” (2, 6).

### Operational definitions

*Good knowledge of IPNs*: Mothers who scored at least 70% out of 10 questions on knowledge adapted from IPN guidelines of the WHO. *Poor knowledge of IPNs*: Mothers who scored less than 70% out of 10 questions on knowledge adapted from IPN guidelines of the WHO. *Good practice of IPNs*: Mothers who scored at least 70% out of 10 questions on practice adapted from IPN guidelines of the WHO. *Poor practice of IPNs*: Mothers who scored less than 70% out of 10 questions on practice adapted from IPN guidelines of the WHO.

### Validity and reliability

To ensure validity, the questionnaire was designed in consultation with experts in the field to adequately address all aspects of the issues being studied. To ensure reliability, the questionnaire was pretested on 5% of the sample size at Ve Dafor Health Center. The questions were then revised after the pretest to ensure correct wording, clarity, transparency, simplicity and proper rephrasing to ensure the collection of consistent and reliable data.

### Data quality assurance

After validating and pretesting the questionnaire, the data were collected by trained research assistants through face-to-face interviews. The questionnaire was initially designed in English but translated into the local languages (Ewe and Twi) of the mothers when necessary during the process of data collection. The data collected were cross-checked for consistency and completeness and kept with the principal researcher.

### Data analysis

Data collected were double-checked for completeness and entered into the Excel spreadsheet 2013. It was imported into Statistical Package for Social Sciences (version 25.00) for analysis. Descriptive data are presented as frequencies and percentages. Bivariable analyses were performed to determine the association between the knowledge and practice of IPNs and the mother’s sociodemographic characteristics and reproductive health factors. We considered it appropriate to set statistical significance as *p* < 0.01 instead of *p* < 0.05. With a *p*-value of <0.05, there is a 1 in 20 chance that a variable will appear to be significant when it is not and purely by chance because we are testing for many explanatory variables. Significant variables (*p* < 0.01) in the bivariable analyses were included in multivariable logistic regression. In the multivariable logistic regression, a *p* < 0.01 was considered significant.

### Ethical considerations

Approval was sought from the North Dayi District, Ghana Health Service with the identification number (NDDHD/GR/002/20) on 11/06/2020 and the management of the health facilities. The introduction was made by research assistants, and consent was sought from each mother by their thumbprint. The aims of the study were explained to the mothers, and approval was granted for the data collection. Privacy and confidentiality were ensured by filling out the questionnaire individually without asking for private information.

## Results

### Sociodemographic characteristics and reproductive health factors

A total of 612 mothers were included as respondents in the study. The majority (65.8%) were between the ages of 25 and 40 years. About 88.9% were married. Over half (*n* = 347) had attained secondary education. Forty-eight percent earned an income between GHc200 and 500 GHc. More than half (52.8%) were primiparous mothers. About 51.8% had visited ANC four times or more. The majority (62.1%) had delivered between 37 and 41 weeks of gestation. Over three-quarters (*n* = 497) had delivered at the health facilities. Approximately 85 % had babies without complications after birth. Fifty-eight percent (*n* = 354) had attended PNC three times or more (Table 1).

**Table 1:** Sociodemographic characteristics and reproductive health factors of mothers (*n* = 612).

Variable	Frequency	Percentage
Total	612	100.0
Mother age		
<25 years	94	15.4
25–40 years	403	65.8
>40 years	115	18.8
Marital status		
Single	57	9.3
Married	544	88.9
Divorced	11	1.8
Educational status		
None	19	3.1
Basic	81	13.2
Secondary	347	56.7
Tertiary	165	27.0
Income level		
<GHc200	201	32.8
GHc200-500	293	47.9
>GHc500	118	19.3
Parity		
Primipara	323	52.8
Multipara	289	47.2
Number of ANC visits		
<4 visits	295	48.2
4+ visits	317	51.8
Gestational age		
<37 weeks of gestation	134	21.9
37–41 weeks of gestation	380	62.1
>41 weeks of gestation	98	16.0
Place of last delivery		
Home	115	18.8
Health facility	497	81.2
Baby’s condition after birth		
Complication	94	15.4
No complication	518	84.6
Number of PNC visits		
<3 visits	258	42.2
3+ visits	354	57.8

Data are presented as the frequency and percentage.

### Knowledge of infection prevention in neonates

Over three-quarters (83.2%) knew that sterile scissors should be used in cutting the umbilical cord. The majority (86.3%) knew that a clean umbilical cord prevents infection. Approximately 80 % were aware that sterile plastic clamps should be used to tie off the cord. The majority (*n* = 517) knew that babies covered with clean clothes prevented infection. Over half (84.8%) knew that skin-to-skin contact between the mother and baby prevents heat loss. Eighty-nine percent knew that babies could be bathed at least 6 h after birth. About 89.2% were aware that eye discharge is a sign of inflammation of the conjunctiva. More than half (*n* = 556) knew that yellowish skin and eyes were signs of jaundice. More than three-quarters (88.7%) knew that vaccination of the baby helps to prevent infection. The majority (86.6%) knew that childhood vaccination provides immunity against disease (Table 2).

**Table 2:** Knowledge of infection prevention in neonates (*n* = 612).

Knowledge statements	Responses *n* (%)		
	True	False	Do not know
Cord care			
Use sterile scissors in cutting the umbilical cord	509 (83.2)	73 (11.9)	30 (4.9)
A clean umbilical cord prevents infection	528 (86.3)	50 (8.2)	34 (5.6)
Use sterile plastic clamps to tie off the cord	490 (80.1)	83 (13.6)	39 (6.4)
Skincare			
Covering the baby with clean clothes prevents infection	517 (84.5)	55 (9.0)	40 (6.5)
Skin-to-skin contact between mother and baby prevents heat loss	519 (84.8)	57 (9.3)	36 (5.9)
Bathing			
Bath the baby at least 6 h after birth	545 (89.0)	50 (8.2)	17 (2.8)
Sign of infection			
Eye discharge is a sign an inflammation of the conjunctiva	546 (89.2)	35 (5.7)	31 (5.1)
The yellowish skin and eyes are the sign of jaundice	556 (90.8)	37 (6.0)	19 (3.1)
Vaccination			
Vaccination of the baby helps to prevent infection	543 (88.7)	43 (7.0)	26 (4.2)
Childhood vaccination provides immunity against disease	530 (86.6)	56 (9.2)	26 (4.2)

Data are presented as the frequency with the corresponding percentage in parentheses.

### Practices of infection prevention in neonates

The majority (78.3%) uncovered the umbilical stump to keep the area dry. Over three-fourths (*n* = 515) cleaned the soiled umbilical stump with warm water. More than three-quarters (*n* = 535) cleaned the cord without smearing any ingredient. The majority (89.4%) gave colostrum to build the baby’s immune system. Over half (77.8%) practiced no diversified feeding before 6 months. About 76.1% breastfed at night to prevent infection. Approximately 73% washed their hands before and after breastfeeding. Over two-thirds (*n* = 411) cleaned their breasts very well before initiating breastfeeding. More than half (*n* = 420) bathed their babies with cold water after birth. The majority (72.1%) received childhood vaccines for babies at the health facility (Table 3).

**Table 3:** Practices of infection prevention in neonates (*n* = 612).

Practice questions	Answers *n* (%)		
	Yes	No	Not sure
Cord care			
Uncovered the umbilical stump to keep the area dry	479 (78.3)	88 (14.4)	45 (7.4)
Clean the soiled umbilicus stump with a warm water	515 (84.2)	61 (10.0)	36 (5.9)
Clean the cord without smearing any ingredient	535 (87.4)	48 (7.8)	29 (4.7)
Breastfeeding			
Give colostrum to build the baby’s immune system	547 (89.4)	33 (5.4)	32 (5.2)
No diversified feeds before 6 months	476 (77.8)	114 (18.6)	22 (3.6)
No breastfeeding at night to prevent infection	85 (13.9)	466 (76.1)	61 (10.0)
Cleaning			
Wash hands before and after breastfeeding	445 (72.7)	134 (21.9)	33 (5.4)
Clean breasts very well before initiating breastfeeding	411 (67.2)	155 (25.3)	46 (7.5)
Bathing			
Bath baby with cold water after birth	121 (19.8)	420 (68.6)	71 (11.6)
Vaccination			
Get childhood vaccines for babies at the health facility	441 (72.1)	150 (24.5)	21 (3.4)

Data are presented as the frequency with the corresponding percentage in parentheses.

### Levels of maternal knowledge and practices of infection prevention in neonates

Less than one-fifth (12.9%) and (21.6%) of the mothers had poor knowledge and poor practices of IPNs, respectively.

### Factors associated with knowledge of infection prevention in neonates

The study found significant differences in the levels of knowledge of IPNs with age, parity, place of current delivery and baby’s condition after birth (*p* < 0.01; Table 4). In the multivariable analyses, none of these variables was statistically significant (*p* < 0.01; Table 5).

**Table 4:** Association between knowledge and practice of infection prevention in neonates and mothers’ sociodemographic characteristics and reproductive health factors.

Variable	Knowledge				Practice			
	Good = 533	Poor = 79	*χ*^2^	*p* value	Good = 480	Poor = 132	*χ*^2^	*p* value
Mother age			11.59	**0.003**			6.88	0.032
<25 years	78 (83.0)	16 (17.0)			66 (70.2)	28 (29.8)		
25–40 years	344 (85.4)	59 (14.6)			316 (78.4)	87 (21.6)		
>40 years	111 (96.5)	4 (3.5)			98 (85.2)	17 (14.8)		
Marital status			1.70	0.427			5.38	0.068
Single	50 (87.7)	7 (12.3)			40 (70.2)	17 (29.8)		
Married	472 (86.8)	72 (13.2)			429 (78.9)	115 (21.1)		
Divorced	11 (100.0)	0 (0.0)			11 (100.0)	0 (0.0)		
Educational status			8.61	0.035			7.49	0.058
None	18 (94.7)	1 (5.3)			14 (73.7)	5 (26.3)		
Basic	75 (92.6)	6 (7.4)			72 (88.9)	9 (11.1)		
Secondary	306 (88.2)	41 (11.8)			262 (75.5)	85 (24.5)		
Tertiary	134 (81.2)	31 (18.8)			132 (80.0)	33 (20.0)		
Income level			0.66	0.718			10.67	**0.005**
<GHc200	178 (88.6)	23 (11.4)			150 (74.6)	51 (25.4)		
GHc200-500	254 (86.7)	39 (13.3)			246 (84.0)	47 (16.0)		
>GHc500	101 (85.6)	17 (14.4)			84 (71.2)	34 (28.8)		
Parity			8.83	**0.003**			4.98	0.026
Primipara	269 (83.3)	54 (16.7)			242 (74.9)	81 (25.1)		
Multipara	264 (91.3)	25 (8.7)			238 (82.4)	51 (17.6)		
Number of ANC visits			0.00	0.985			9.14	**0.002**
<4 visits	257 (87.1)	38 (12.9)			216 (73.2)	79 (26.8)		
4+ visits	276 (87.1)	41 (12.9)			264 (83.3)	53 (16.7)		
Gestational age			4.78	0.092			3.73	0.155
<37 weeks of gestation	115 (85.8)	19 (14.2)			97 (72.4)	37 (27.6)		
37–41 weeks of gestation	326 (85.6)	54 (14.2)			305 (80.3)	75 (19.7)		
>41 weeks of gestation	92 (93.9)	6 (6.1)			78 (79.6)	20 (20.4)		
Place of last delivery			11.20	**0.001**			3.86	0.050
Home	111 (96.5)	4 (3.5)			98 (85.2)	17 (14.8)		
Health facility	422 (84.9)	75 (15.1)			382 (76.9)	115 (23.1)		
Baby’s condition after birth			9.33	**0.002**			1.36	0.244
Complication	91 (96.8)	3 (3.2)			78 (83.9)	16 (16.1)		
No complication	442 (85.3)	76 (14.7)			402 (77.6)	116 (22.4)		
Number of PNC visits			0.65	0.420			6.04	**0.014**
<3 visits	228 (88.4)	30 (11.6)			190 (73.6)	68 (26.4)		
3+ visits	305 (86.2)	49 (13.8)			290 (81.9)	64 (18.1)		

Data are presented as the frequency with the corresponding percentage in parentheses, significant at *p* < 0.01. Bold values indicates significant at *p* < 0.001.

**Table 5:** Factors associated with knowledge of infection prevention in neonates.

Variable	Knowledge		Unadjusted		Adjusted	
	Good = 533	Poor = 79	UOR (95% CI)	*p* value	AOR (95% CI)	*p* value
Mother age						
< 25 years	78 (83.0)	16 (17.0)	5.69 (1.83–17.68)	0.003	3.06 (0.42–22.21)	0.269
25–40 years	344 (85.4)	59 (14.6)	4.76 (1.69–13.40)	0.003	2.56 (0.37–17.56)	0.339
>40 years	111 (96.5)	4 (3.5)	1		1	
Parity						
Primipara	269 (83.3)	54 (16.7)	2.12 (1.28–3.51)	0.003	1.48 (0.86–2.54)	0.155
Multipara	264 (91.3)	25 (8.7)	1		1	
Place of last delivery						
Home	111 (96.5)	4 (3.5)	0.20 (0.07–0.57)	0.002		
Health facility	422 (84.9)	75 (15.1)	1			
Baby’s condition after birth						
Complication	91 (96.8)	3 (3.2)	0.19 (0.06–0.62)	0.006	0.63 (0.07–5.48)	0.676
No complication	442 (85.3)	76 (14.7)	1		1	

Significant at *p* < 0.01; CI, confidence interval; UOR, unadjusted odds ratio; AOR, adjusted odds ratio; and 1, reference.

### Factors associated with practices of infection prevention in neonates

The study found significant associations in the levels of practice of IPNs with income level, number of ANC visits, number of PNC visits and mothers’ knowledge of IPNs (*p* < 0.01; Table 4). In the multivariable analyses, only mothers’ knowledge of IPNs was statistically significant (*p* < 0.01). Mothers who had poor knowledge of IPNs [AOR = 13.33 (95% CI: 7.69–23.26), *p* < 0.001] were 13.33 more likely to have a poor practice of IPNs compared to those who had good knowledge of IPNs (Table 6).

**Table 6:** Factors associated with practices of infection prevention in neonates.

	Practice		Unadjusted		Adjusted	
Variable	Good = 480	Poor = 132	UOR(95% CI)	*p* value	AOR (95% CI)	*p* value
Income level						
<GHc200	150 (74.6)	51 (25.4)	0.84 (0.51–1.40)	0.502	0.61 (0.29–1.26)	0.181
GHc200-500	246 (84.0)	47 (16.0)	0.47 (0.29–0.78)	0.004	0.39 (0.14–1.14)	0.087
>GHc500	84 (71.2)	34 (28.8)	1		1	
Number of ANC visits						
<4 visits	216 (73.2)	79 (26.8)	1.82 (1.23–2.70)	0.003	0.83 (0.28–2.51)	0.742
4+ visits	264 (83.3)	53 (16.7)	1		1	
Number of PNC visits						
<3 visits	190 (73.6)	68 (26.4)	1.62 (1.10–2.39)	0.014	1.85 (0.91–3.76)	0.088
3+ visits	290 (81.9)	64 (18.1)	1		1	
Mothers’ knowledge of IPNs						
Good	454 (85.2)	79 (14.8)	1		1	
Poor	26 (32.9)	53 (67.1)	11.72 (6.92–19.84)	<0.001	13.33 (7.69–23.26)	**<0.001**

Significant at *p* < 0.01; CI: confidence interval, UOR: unadjusted odds ratio, AOR: adjusted odds ratio and 1 is the reference.

## Discussion

In Ghana, neonatal deaths occur every 15 min, and the mortality rate of 29 per 1,000 in 2014 shows that Ghana’s neonatal mortality rate (NMR) is greater than Africa’s average of 27 per 1,000, which is the maximum NMR in West Africa (26). This implies that without intensive efforts, Ghana is unable to attain Sustainable Development Goal (SDG) 3 of reducing under five and NMRs to 25 and 12 per 1,000 live births, respectively (26). Per the Ghana Demographic and Health Survey, from 1993 to 2014, the NMR in Ghana declined by 44%, while the northern region reported a 67% reduction, 25% in the Volta Region and 10% in the Upper West Region (27). Presently, data are scarce on neonatal mortality due to infection in Ghana and particularly in the Volta region, and reducing NMR to acceptable levels is difficult without good maternal knowledge and practice regarding infection prevention. This study aimed to assess sociodemographic characteristics and reproductive health factors associated with maternal knowledge and practice of IPNs in North Dayi District, Ghana.

The findings of this study showed that less than one-fifth of the mothers had poor knowledge of IPNs according to the WHO’s guidelines. Similar findings were shown in studies conducted in Ghana, South Sudan, Nepal and Kenya (2, 9–11), which found that less than half of the mothers had poor knowledge of IPNs. In the present study, over three-quarters of the mothers had a formal background of education, and the types of education provided to mothers by the knowledgeable healthcare professionals and their prioritization of ANC and PNC could contribute to their good knowledge of IPNs. In contrast, another study performed in Ghana (5), Ethiopia (6, 12) and Saudi Arabia (13) found that over half of the mothers had poor knowledge of IPNs. The disparities across these studies compared with the present study could be the cut-off point used for knowledge scores.

In our study, the percentage of mothers who incorrectly practiced IPNs was less than half, which corresponds to studies performed in Ghana, Ethiopia and Nepal (2, 8, 9) that found the poor practice of IPNs in less than half of the mothers. The reason is that the majority of the mothers in the present study were compliant with the acquired knowledge of IPNs, which is shown in their practice. The opposite was found in studies performed in Ghana (14), Uganda (15), Ethiopia (7, 16) and Nigeria (17), which reported that over half of the mothers poorly practiced IPNs. The disparities could be due to the study population, cut-off point used for categorization of the practice of IPNs, noncompliance of mothers with knowledge of IPNs and cultural differences in regards to practices of IPNs.

This study revealed that mothers who had poor knowledge of IPNs were more likely to have poor IPN practices than those who had good IPN knowledge. This finding concurs with studies performed in Ethiopia (7, 8) and Nepal (9), which found that mothers who had good knowledge of IPNs were more likely to have good practices of IPNs. This means that the good knowledge of IPNs among the majority of the mothers in the present study increases their practices of IPNs. The findings would be useful for the Health Directorate of North Dayi District to develop appropriate strategies for mothers with inadequate knowledge of IPNs, which can be reflected in their practice of IPNs.

### Strengths and limitations

The study provides information on maternal knowledge and practice of IPNs in North Dayi District, which has not been thoroughly presented by previous studies in Ghana, to aid in strategy and policy development to prevent and reduce neonatal infection. Regardless of the strengths, the study has some limitations. First, the results regarding the maternal knowledge and practice of IPNs are likely to be based on mothers’ recall, which could result in social desirability bias. Second, the sample size excluded mothers who did not attend the various health facilities during the study period, those unable to communicate properly and those who attended but disagreed to take part in the study. Excluding mothers who did not attend the clinic for various reasons, could undermine the reported rates in this study. Thirdly, we did not study IPN with neonatal deaths secondary to infection. To date, we could not find any reports on the percentage of neonatal deaths due to infection in Ghana, particularly in North Dayi District, whether low or high and perhaps more focus needs to be given to further studies.

## Conclusion

Less than one-fifth of the mothers had poor knowledge and poor practice of IPNs according to the WHO’s guidelines. Mothers who had poor knowledge of IPNs were more likely to have a poor practice of IPNs. The Ghana Health Service should continue to enforce home visits by nurse assistants to identify and educate mothers who are unable to attend ANC and PNC. The Health Directorate of North Dayi District should encourage the use of IPN guidelines by the WHO for outreach and campaigns. Finally, the limitations identified in our study highlight the need for similar studies in communities to develop strategies to prevent and reduce neonatal infection.

## Data availability statement

The original contributions presented in the study are included in the article/Supplementary material, further inquiries can be directed to the corresponding author.

## Ethics statement

Approval was sought from the North Dayi District Health Directorate, Ghana Health Service with identity (NDDHD/GR/002/20) on 11/06/2020. Permission was granted by the management of the health centers. The research assistants introduced themselves, and consent was granted from each neonatal mother before the data collection. The aims of the study were clarified by the language of the neonatal mothers. Informed approval was granted by the neonatal mother by a thumbprint. No place was allowed for personal documentation for confidentiality purposes. The questionnaires were answered individually to certify privacy. Written informed consent to participate in this study was provided by the participants’ legal guardian/next of kin.

## Author contributions

LT, BA, ED, DO, and JG conceptualized and designed the study. LT, JT, NA, KS, KM, SS, and KC coordinated and participated in the data collection. LT drafted the manuscript. All authors were involved in reviewing it for intellectual content. All authors contributed to the article and approved the submitted version.

## Conflict of interest

The authors declare that the research was conducted in the absence of any commercial or financial relationships that could be construed as a potential conflict of interest.

## Publisher’s note

All claims expressed in this article are solely those of the authors and do not necessarily represent those of their affiliated organizations, or those of the publisher, the editors and the reviewers. Any product that may be evaluated in this article, or claim that may be made by its manufacturer, is not guaranteed or endorsed by the publisher.

## Abbreviations

WHO, World Health Organization
SSA, Sub-Saharan Africa
ANC, Antenatal Clinic
PNC, Postnatal Clinic
SDG, Sustainable Development Goal
AOR, adjusted odds ratio
UOR, Unadjusted Odds Ratio
Cl, confidence interval
NMR, Neonatal mortality rate
