# Supplementary material for: Sociodemographic characteristics and reproductive health factors associated with maternal knowledge and practice of infection prevention in neonates in North Dayi District, Ghana
Source: Front Public Health. 2023 Apr 17;11:1062268. doi: 10.3389/fpubh.2023.1062268 (PMC10149744; doi:10.3389/fpubh.2023.1062268)
Supplement: Supplementary file 1 [file Data_Sheet_1.PDF]

Factors associated with knowledge and practice of infection prevention in neonates among neonatal mothers in North Dayi District, Ghana

Please tick (√) where appropriate. All responses will be coded, kept confidential and analyzed so that no personal information is revealed.

I have given my consent to partake in this study Yes ◯ No ◯

Sociodemographic and reproductive health data

1	Mother age (years)	< 25 ◯	25-40 ◯	>40 ◯
2	Marital status	Single ◯	Married ◯	Divorced ◯
3	Educational status	None ◯ Basic ◯	Secondary ◯	Tertiary ◯
4	Income level	<GHc200 ◯	200-500GHc ◯	>GHc500 ◯
5	Parity	Primipara ◯	Multipara ◯	
6	Number of ANC visits	<4 visits ◯	4+ visits ◯	
7	Gestational age	<37 weeks ◯	37-41 weeks ◯	>41 weeks ◯
8	Place of last delivery	Home ◯	Health facility ◯	
9	Baby's condition after birth	Complication ◯	No complication ◯	
10	Number of PNC visits	<3 visits ◯	3+ visits ◯	

Knowledge of infection prevention in neonates

True (T), False (F) and Do not know (DNK)

	Statement	Responses		
		T	F	DNK
11	Use sterile scissors in cutting the umbilical cord	◯	◯	◯
12	A clean umbilical cord prevents infection	◯	◯	◯
13	Use sterile plastic clamps to tie off the cord	◯	◯	◯
14	Covering the baby with clean clothes prevents infection	◯	◯	◯
15	Skin-to-skin contact between mother and baby prevents heat loss	◯	◯	◯
16	Bath the baby at least six hours after birth	◯	◯	◯
17	Eye discharge is a sign an inflammation of the conjunctiva	◯	◯	◯
18	The yellowish skin and eyes are the sign of jaundice	◯	◯	◯
19	Vaccination of the baby helps to prevent infection	◯	◯	◯
20	Childhood vaccination provides immunity against disease	◯	◯	◯

Practices of infection prevention in neonates				
Yes (Y), No (N) and Not sure (NS)				
Statement		Responses		
21	Uncovered the umbilical stump to keep the area dry	Y ◯	N ◯	NS ◯
22	Clean the soiled umbilicus stump with a warm water	Y ◯	N ◯	NS ◯
23	Clean the cord without smearing any ingredient	Y ◯	N ◯	NS ◯
24	Give colostrum to build the baby's immune system	Y ◯	N ◯	NS ◯
25	No diversified feeds before six months	Y ◯	N ◯	NS ◯
26	No breastfeeding at night to prevent infection	Y ◯	N ◯	NS ◯
27	Bath baby with cold water after birth	Y ◯	N ◯	NS ◯
28	Wash hands before and after breastfeeding	Y ◯	N ◯	NS ◯
29	Clean breasts very well before initiating breastfeeding	Y ◯	N ◯	NS ◯
30	Get childhood vaccines for babies at the health facility	Y ◯	N ◯	NS ◯

The questions on the knowledge and practice of infection prevention in neonates (IPNs) were adapted from the previous studies [2, 4, 6, 7, 9–11, 18] and standardized according to the IPN guidelines of the WHO [23, 24]. 23. World Health Organization WHO. WHO recommendations on maternal and newborn care for a positive postnatal experience. https://www.who.int/publications/i/item/9789240045989. March 2022. 24. Maternal and Child Survival Program MCSP. Postnatal Care for Mothers and Newborns Highlights from the World Health Organization 2013 Guidelines. httpwww.mcsprogram.org. 2015. The references to the previous studies are provided in the manuscript.

World Health Organization

human reproduction programme hrp
research for impact
UNDP · UNFPA · UNICEF · WHO · WORLD BANK

WHO recommendations on **maternal and newborn care for a positive postnatal experience**

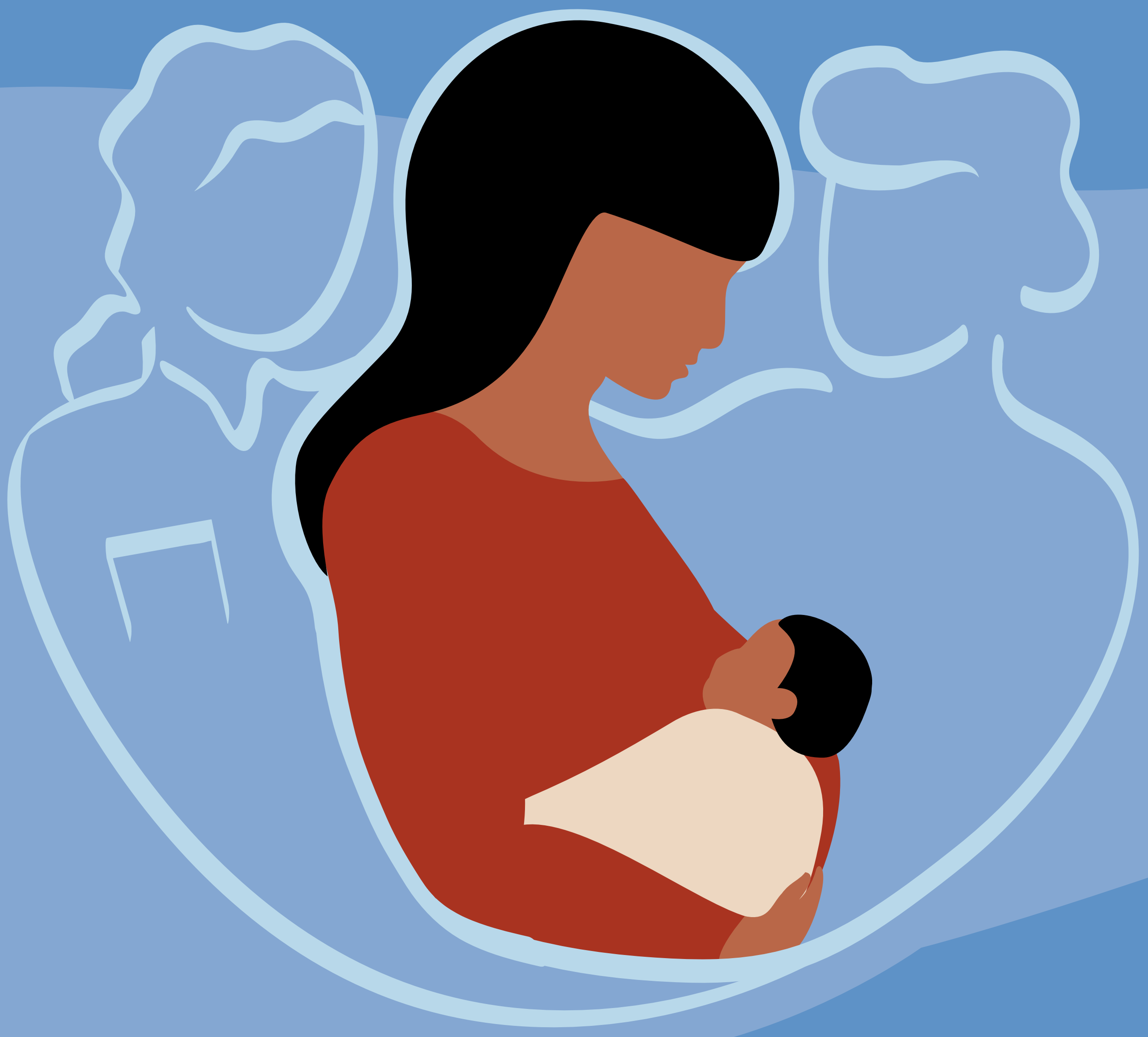

WHO recommendations on

maternal and newborn care for a positive postnatal experience

World Health Organization

human reproduction programme hrp

research for impact

UNDP · UNFPA · UNICEF · WHO · WORLD BANK

WHO recommendations on maternal and newborn care for a positive postnatal experience

This publication is the update of the document published in 2014 entitled "WHO recommendations on postnatal care of the mother and newborn".

ISBN 978-92-4-004598-9 (electronic version)
ISBN 978-92-4-004599-6 (print version)

© World Health Organization 2022

Some rights reserved. This work is available under the Creative Commons Attribution-NonCommercial-ShareAlike 3.0 IGO licence (CC BY-NC-SA 3.0 IGO; https://creativecommons.org/licenses/by-nc-sa/3.0/igo).

Under the terms of this licence, you may copy, redistribute and adapt the work for non-commercial purposes, provided the work is appropriately cited, as indicated below. In any use of this work, there should be no suggestion that WHO endorses any specific organization, products or services. The use of the WHO logo is not permitted. If you adapt the work, then you must license your work under the same or equivalent Creative Commons licence. If you create a translation of this work, you should add the following disclaimer along with the suggested citation: "This translation was not created by the World Health Organization (WHO). WHO is not responsible for the content or accuracy of this translation. The original English edition shall be the binding and authentic edition".

Any mediation relating to disputes arising under the licence shall be conducted in accordance with the mediation rules of the World Intellectual Property Organization (http://www.wipo.int/amc/en/mediation/rules/).

Suggested citation. WHO recommendations on maternal and newborn care for a positive postnatal experience. Geneva: World Health Organization; 2022. Licence: CC BY-NC-SA 3.0 IGO.

Cataloguing-in-Publication (CIP) data. CIP data are available at http://apps.who.int/iris.

Sales, rights and licensing. To purchase WHO publications, see http://apps.who.int/bookorders. To submit requests for commercial use and queries on rights and licensing, see https://www.who.int/copyright.

Third-party materials. If you wish to reuse material from this work that is attributed to a third party, such as tables, figures or images, it is your responsibility to determine whether permission is needed for that reuse and to obtain permission from the copyright holder. The risk of claims resulting from infringement of any third-party-owned component in the work rests solely with the user.

General disclaimers. The designations employed and the presentation of the material in this publication do not imply the expression of any opinion whatsoever on the part of WHO concerning the legal status of any country, territory, city or area or of its authorities, or concerning the delimitation of its frontiers or boundaries. Dotted and dashed lines on maps represent approximate border lines for which there may not yet be full agreement.

The mention of specific companies or of certain manufacturers' products does not imply that they are endorsed or recommended by WHO in preference to others of a similar nature that are not mentioned. Errors and omissions excepted, the names of proprietary products are distinguished by initial capital letters.

All reasonable precautions have been taken by WHO to verify the information contained in this publication. However, the published material is being distributed without warranty of any kind, either expressed or implied. The responsibility for the interpretation and use of the material lies with the reader. In no event shall WHO be liable for damages arising from its use.

Cover design by Lushomo. Design and layout by Green Ink Publishing Services Ltd.

Contents

Acknowledgements iv

Acronyms and abbreviations v

Executive summary vii

1. Introduction 1

2. Methods 3

3. Evidence and recommendations 12

   A. Maternal care 13

   B. Newborn care 97

   C Health systems and health promotion interventions 150

4. Implementation of the WHO postnatal care recommendations 195

5. Dissemination 198

6. Applicability issues 199

7. Monitoring and evaluating the impact of the guideline 200

8. Updating of the guideline 201

9. References 202

Annex 1: Contributors to the guideline 218

Annex 2: Summary of declarations of interests from the Guideline Development Group (GDG) members and how they were managed 222

Annex 3: Template “summary of judgements” table for evidence-to-decision domains 224

*WHO recommendations on maternal and newborn care for a positive postnatal experience. Web Annexes**

Web Annex 1: Priority questions and outcomes

Web Annex 2: Changes from the approved scope of this guideline

Web Annex 3: Other WHO guidelines with recommendations relevant to routine postnatal care

Web Annex 4: Research implications

Web Annex 5: Implementation considerations specific to individual recommendations

*WHO recommendations on maternal and newborn care for a positive postnatal experience. Web Supplement. Evidence base**

The standardized criteria used in grading the evidence and the GRADE tables have been published in this separate Web Supplement. These evidence tables are referred to within this document by number, prefixed with "EB" (for evidence base), for ease of reference.

* Available at: https://www.who.int/publications/i/item/9789240045989

Acknowledgements

The WHO Departments of Maternal, Newborn, Child and Adolescent Health and Ageing (MCA), Mental Health and Substance Use (MSD), Nutrition and Food Safety (NFS), and Sexual and Reproductive Health and Research (SRH) gratefully acknowledge the contributions that many individuals and organizations have made to the development of this guideline.

Work on this guideline was initiated by Mercedes Bonet, Olufemi Oladapo (SRH), Rajiv Bahl and Anayda Portela (MCA). Mercedes Bonet and Anayda Portela coordinated the guideline development process with a WHO Steering Group including Fernando Althabe, Rajiv Bahl, Neerja Chowdhary, Tarun Dua, Karen Edmond, Shuchita Gupta, Olufemi Oladapo, Lisa Rogers and João Paulo Souza. The following WHO headquarters' staff provided inputs on the guideline content: Avni Amin, Robert Butchart, Shelly Chadha, Bernadette Daelmans, Batool Fatima, Mary Lyn Gaffield, Claudia García-Moreno, Tracey Goodman, Hebe Gouda, Laurence Grummer-Strawn, Cheryl Johnson, Michelle McIsaac, Cecily Miller, Antonio Montresor, Allisyn Moran, Denise Mupfasoni, Morkor Newman Owiredu, Silvio Paolo Mariotti, Vladimir Poznyak, Vinayak Prasad, Michelle Rodolph, Marcus Stahlhofer, Chiara Servili, Özge Tunçalp, Sabine Verkuijls and Juana Willumsen. WHO regional advisors who contributed to the guideline include Jamela Al-Raiby, Bremen de Mucio, Pablo Duran, Karima Gholbzouri, Anoma Jayathilaka, Oleg Kuzmenko, Assumpta Muriithi and Triphonie Nkurunziza.

WHO extends sincere thanks to the chairs of the Guideline Development Group (GDG): Jane Fisher, James Neilson and Siddarth Ramji for leading the GDG meetings; and to the GDG members: Shabina Ariff, Abdullah Baqui, Blami Dao, Louise Tina Day, Abiy Seifu Estifanos, Duncan Fisher, Zelee Hill, Caroline Homer, Tamar Kabakian-Khasholian, Mary Kinney, Tina Lavender, Pisake Lumbiganon, Address Malata, Ibone Olza, Malvarappu Prakasamma, Parminder Suchdev, Mark Tomlinson and Hayfaa Wahabi. WHO also thanks Rafat Jan, Silke Mader, Matthews Mathai, Linda Richter, Jane Sandall and Steve Wall, who were members of the External Review Group (ERG). WHO acknowledges the various organizations that were represented as observers at the GDG meetings, including: Jeffrey Smith (Bill & Melinda Gates Foundation), Ann Yates (International Confederation of Midwives), Carlos Fuchtner (International Federation of Gynecology and Obstetrics), William Keenan (International Pediatric Association), Smita Kumar and Rebecca Levine (United States Agency for International Development [USAID]). WHO appreciates the contributions of Petra ten Hoope-Bender, Willibald Zeck (United Nations Population Fund), Gagan Gupta and Tedbabe Degefie Hailegebriel (United Nations Children's Fund).

WHO appreciates the contributions of international stakeholders who participated in the scoping exercise for the guideline development process. For the content of this guideline, special thanks to: the authors of the systematic reviews used to develop the recommendations for their assistance and collaboration; Frances Kellie and Leanne Jones for coordinating the updates of the relevant Cochrane systematic reviews; and Edgardo Abalos, Monica Chamillard, Soo Downe, Virginia Diaz, Kenneth Finlayson, Ani Movsisyan, Susan Munabi-Babigumira, Julia Pascale and Yanina Sguassero, who performed quality appraisal of the evidence from the reviews. Ani Movsisyan and Aleena Wojcieszek double-checked the evidence profiles from all systematic reviews and, with members of the Technical Working Group and WHO Steering Group, prepared the corresponding narrative summaries and evidence-to-decision frameworks. Mercedes Bonet, Anayda Portela and Aleena Wojcieszek drafted the final guideline document with valuable inputs from Karen Edmond and Shuchita Gupta, before it was reviewed by other members of the WHO Steering Group and the GDG.

USAID and the UNDP-UNFPA-UNICEF-WHO-World Bank Special Programme of Research, Development and Research Training in Human Reproduction (HRP), a cosponsored programme executed by the WHO, funded this work. The views of the funding bodies have not influenced the content of this guideline.

Acronyms and abbreviations

**25(OH)D**	25-hydroxyvitamin D
**AABR**	automated auditory brainstem response
**AF**	anti-secretory factor
**ALTE**	apparent life-threatening event
**AU$**	Australian dollar
**BIND**	bilirubin-induced neurological dysfunction
**Can$**	Canadian dollar
**CASP**	Critical Appraisal Skills Programme
**CERQual**	Confidence in the Evidence from Reviews of Qualitative research
**CHC**	combined hormonal contraception
**CHEC**	Consensus Health Economic Criteria
**CHW**	community health worker
**CI**	confidence interval
**CREP**	Centro Rosarino de Estudios Perinatales
**CRVS**	civil registration and vital statistics
**Cu-IUD**	copper-bearing intrauterine device
**DALY**	disability-adjusted life-year
**dB**	decibel
**DDST**	Denver Developmental Screening Test
**DECIDE**	Developing and Evaluating Communication Strategies to Support Informed Decisions and Practice Based on Evidence
**DHS**	Demographic and Health Survey
**DOI**	declaration of interest
**EB**	evidence base
**ECP**	emergency contraceptive pill
**EPDS**	Edinburgh Postnatal Depression Scale
**ERG**	External Review Group
**EtD**	evidence-to-decision
**ETG**	etonogestrel
**FIGO**	International Federation of Gynecology and Obstetrics
**GDG**	Guideline Development Group
**GRADE**	Grading of Recommendations Assessment, Development and Evaluation
**HIC**	high-income country
**HRP**	UNDP-UNFPA-UNICEF-WHO-World Bank Special Programme of Research, Development and Research Training in Human Reproduction
**ICM**	International Confederation of Midwives
**IPA**	International Pediatric Association
**IPD**	individual patient data
**IU**	international unit
**LMIC**	low- and middle-income country
**LNG**	levonorgestrel
**LNG-IUD**	levonorgestrel-releasing intrauterine device
**MCA**	Department of Maternal, Newborn, Child and Adolescent Health and Ageing (at WHO)
**MD**	mean difference
**MEC**	medical eligibility criteria
**MLCC**	midwife-led continuity of care
**MNH**	maternal and newborn health
**MSD**	Department of Mental Health and Substance Use (at WHO)
**NFS**	Department of Nutrition and Food Safety (at WHO)
**NSAID**	non-steroidal anti-inflammatory drug
**OAE**	otoacoustic emissions
**OR**	odds ratio
**PBHL**	permanent bilateral hearing loss
**PCG**	Pregnancy and Childbirth Group (Cochrane)
**PFMT**	Pelvic floor muscle training
**PHQ-9**	Patient Health Questionnaire-9
**PICO**	population (P), intervention (I), comparator (C), outcome (O)
**POI**	progestogen-only injectable contraceptive
**POP**	progestogen-only pill
**PrEP**	pre-exposure prophylaxis
**PSBI**	possible serious bacterial infection
**PVR**	progesterone-releasing vaginal ring
**QALY**	quality-adjusted life-year
**RCT**	randomized controlled trial

**RR**	risk ratio
**SDG**	Sustainable Development Goal
**SDM**	standard mean difference
**SIDS**	sudden infant death syndrome
**spp.**	several species (plural)
**SRH**	Department of Sexual and Reproductive Health and Research (at WHO)
**SUDI**	sudden unexpected death in infancy
**TB**	tuberculosis
**TcB**	transcutaneous bilirubinometer/ bilirubinometry
**TDF**	tenofovir disoproxil fumarate
**TSB**	total serum bilirubin
**TWG**	Technical Working Group
**UNDP**	United Nations Development Programme
**UNFPA**	United Nations Population Fund
**UNHS**	universal newborn hearing screening
**UNICEF**	United Nations Children's Fund
**US$**	United States dollar
**USAID**	United States Agency for International Development
**VTE**	venous thromboembolism
**WHO**	World Health Organization

Executive summary

Introduction

The postnatal period, defined here as the period beginning immediately after the birth of the baby and extending up to six weeks (42 days), is a critical time for women, newborns, partners, parents, caregivers and families. Yet, during this period, the burden of maternal and neonatal mortality and morbidity remains unacceptably high, and opportunities to increase maternal well-being and to support nurturing newborn care have not been fully utilized. Postnatal care services are a fundamental component of the continuum of maternal, newborn and child care, and key to achieving the Sustainable Development Goals (SDGs) on reproductive, maternal and child health, including targets to reduce maternal mortality rates and end preventable deaths of newborns.

In line with the SDGs and the Global Strategy for Women's, Children's and Adolescents' Health, and in accordance with a human rights-based approach, postnatal care efforts must expand beyond coverage and survival alone to include quality of care. This guideline aims to improve the quality of essential, routine postnatal care for women and newborns with the ultimate goal of improving maternal and newborn health and well-being. It recognizes a "positive postnatal experience" as a significant end point for all women giving birth and their newborns, laying the platform for improved short- and long-term health and well-being. A positive postnatal experience is defined as one in which women, newborns, partners, parents, caregivers and families receive information, reassurance and support in a consistent manner from motivated health workers; where a resourced and flexible health system recognizes the needs of women and babies, and respects their cultural context.

This is a consolidated guideline of new and existing recommendations on routine postnatal care for women and newborns receiving facility- or community-based postnatal care in any resource setting. It provides a comprehensive set of recommendations for care during the postnatal period, focusing on the essential package that all women and newborns should receive, with due attention to quality of care; that is, the provision and experience of care. This guideline updates and expands upon the 2014 *WHO recommendations on postnatal care of the mother and newborn*, and complements existing WHO guidelines on the management of postnatal complications.

Target audience

The recommendations in this guideline are intended to inform the development of relevant national and subnational health policies, clinical protocols and programmatic guides. Therefore, the target audience includes national and subnational public health policy-makers, implementers and managers of maternal, newborn and child health programmes, health facility managers, health workers (including midwives, auxiliary nurse-midwives, nurses, obstetricians, paediatricians, neonatologists, general medical practitioners and community health workers), nongovernmental organizations, professional societies involved in the planning and management of maternal, newborn and child health services, academic staff involved in training health workers, and women's and parents' groups.

The terms woman, mother, partner, parents and caregivers have been used throughout this guideline. These terms have been defined in an attempt to promote inclusivity of all individuals who have given birth, and in recognition of the diverse roles of all individuals involved in providing care and support during the postnatal period.

Guideline development methods

The guideline was developed using standard operating procedures in accordance with the process described in the *WHO handbook for guideline development*. Briefly, these procedures include: (i) identification of priority questions and outcomes; (ii) evidence retrieval and synthesis; (iii) assessment of the evidence; (iv) formulation of recommendations; and (v) planning for implementation, dissemination, impact evaluation and updating of the guideline. The quality of the scientific evidence underpinning the recommendations was graded using the Grading of Recommendations Assessment, Development and Evaluation (GRADE) and Confidence in the Evidence from Reviews of Qualitative research (CERQual) approaches for quantitative and qualitative evidence, respectively. Findings from individual cost-effectiveness studies were assessed using the Consensus Health Economic Criteria (CHEC) checklist. The DECIDE framework (Developing and Evaluating Communication Strategies to Support Informed Decisions and Practice Based on Evidence), an evidence-to-decision tool, was used to guide the compilation of evidence, judgements on the different criteria, and the formulation of recommendations by the Guideline Development Group (GDG), including: the effects of an intervention on maternal, newborn and health systems outcomes, and considerations around values of women, parents and health workers; resources; equity; acceptability; and the feasibility of the interventions. The GDG is an international group of experts assembled for the purpose of developing this guideline – at nine virtual GDG meetings held between September 2020 and June 2021. In addition, existing recommendations from current Guideline Review Committee-approved WHO guidelines that were relevant to postnatal care were identified and integrated into this guideline for the purpose of providing a comprehensive document for end-users.

Recommendations

The GDG meetings led to 63 recommendations to improve provision, utilization and experience of postnatal care: 31 are newly developed GDG recommendations and 32 are recommendations integrated from existing WHO guidelines. Recommendations are grouped according to maternal care, newborn care, and health systems and health promotion interventions. Interventions were classified as recommended, not recommended, or recommended under certain conditions based on the GDG's judgements according to the DECIDE criteria, which informed the direction and category of the recommendation. Where the GDG recommended or did not recommend an intervention, the resulting recommendation is relevant to all women in the postpartum period and newborns, unless otherwise indicated in the recommendation. Where the GDG recommended an intervention only in specific contexts, it judged the evidence to be applicable only to these situations, settings or populations. For all recommendations, the GDG provided remarks, including additional contextual information relating to context-specific recommendations, where needed. Users of the guideline should refer to these remarks, which are presented along with the evidence summaries in the full version of the guideline.

Summary list of recommendations on maternal and newborn care for a positive postnatal experience

Care category	Recommendation	Category of recommendation
**A. MATERNAL CARE**		
**Maternal assessment**		
**Physiological assessment of the woman**[1]	**1.** All postpartum women should have regular assessment of vaginal bleeding, uterine tonus, fundal height, temperature and heart rate (pulse) routinely during the first 24 hours, starting from the first hour after birth. Blood pressure should be measured shortly after birth. If normal, the second blood pressure measurement should be taken within 6 hours. Urine void should be documented within 6 hours.<br>At each subsequent postnatal contact beyond 24 hours after birth, enquiries should continue to be made about general well-being and assessments made regarding the following: micturition and urinary incontinence, bowel function, healing of any perineal wound, headache, fatigue, back pain, perineal pain and perineal hygiene, breast pain and uterine tenderness and lochia.	Recommended
**HIV catch-up testing**[2]	**2a.** In high HIV burden settings,[a] catch-up postpartum HIV testing is needed for women of HIV-negative or unknown status who missed early antenatal contact testing or retesting in late pregnancy at a third trimester visit.	Context-specific recommendation
	**2b.** In low HIV burden settings,[b] catch-up postpartum HIV testing can be considered for women of HIV-negative or unknown status who missed early antenatal contact testing or retesting in late pregnancy at a third trimester visit as part of the effort to eliminate mother-to-child transmission of HIV. Countries could consider this only for women who are in serodiscordant relationships, where the partner is not virally suppressed on ART, or who had other known ongoing HIV risks in late pregnancy at a third trimester visit.	Context-specific recommendation
**Screening for tuberculosis disease**[3]	**3a.** Systematic screening for tuberculosis (TB) disease may be conducted among the general population, including of women in the postpartum period, in areas with an estimated TB disease prevalence of 0.5% or higher.	Context-specific recommendation
	**3b.** In settings where the TB disease prevalence in the general population is 100/100 000 population or higher, systematic screening for TB disease may be conducted among women in the postpartum period.	Context-specific recommendation
	**3c.** Household contacts and other close contacts of individuals with TB disease, including women in the postpartum period and newborns, should be systematically screened for TB disease.	Recommended
**Interventions for common physiological signs and symptoms**		
**Local cooling for perineal pain relief**	**4.** Local cooling, such as with ice packs or cold pads, can be offered to women in the immediate postpartum period for the relief of acute pain from perineal trauma sustained during childbirth, based on a woman's preferences and available options.	Recommended

a High-prevalence settings are defined in the 2015 WHO publication *Consolidated guidelines on HIV testing services* as settings with greater than 5% HIV prevalence in the population being tested.
b Low-prevalence settings are settings with less than 5% HIV prevalence in the population being tested.

1 Adapted and integrated from the 2014 *WHO recommendations on postnatal care of the mother and newborn*.
2 Adapted and integrated from the 2019 WHO *Consolidated guidelines on HIV testing services*.
3 Adapted and integrated from the 2021 *WHO consolidated guidelines on tuberculosis. Module 2: screening – systematic screening for tuberculosis disease*.

Care category	Recommendation	Category of recommendation
**Oral analgesia for perineal pain relief**	**5.** Oral paracetamol is recommended as first-line choice when oral analgesia is required for the relief of postpartum perineal pain.	Recommended
**Pharmacological relief of pain due to uterine cramping/ involution**	**6.** Oral non-steroidal anti-inflammatory drugs (NSAIDs) can be used when analgesia is required for the relief of postpartum pain due to uterine cramping after childbirth, based on a woman's preferences, the clinician's experience with analgesics and availability.	Recommended
**Postnatal pelvic floor muscle training for pelvic floor strengthening**	**7.** For postpartum women, starting routine pelvic floor muscle training (PFMT) after childbirth for the prevention of postpartum urinary and faecal incontinence is not recommended.	Not recommended
**Non-pharmacological interventions to treat postpartum breast engorgement**	**8.** For treatment of breast engorgement in the postpartum period, women should be counselled and supported to practice responsive breastfeeding, good positioning and attachment of the baby to the breast, expression of breastmilk, and the use of warm or cold compresses, based on a woman's preferences.	Recommended
**Pharmacological interventions to treat postpartum breast engorgement**	**9.** The use of pharmacological interventions such as subcutaneous oxytocin and proteolytic enzyme therapy for the treatment of breast engorgement in the postpartum period is not recommended.	Not recommended
**Preventive measures**		
**Non-pharmacological interventions to prevent postpartum mastitis**	**10.** For the prevention of mastitis in the postpartum period, women should be counselled and supported to practice responsive breastfeeding, good positioning and attachment of the baby to the breast, hand expression of breastmilk, and the use of warm or cold compresses, based on a woman's preferences.	Recommended
**Pharmacological interventions to prevent postpartum mastitis**	**11.** Routine oral or topical antibiotic prophylaxis for the prevention of mastitis in the postpartum period is not recommended.	Not recommended
**Prevention of postpartum constipation**	**12.** Dietary advice and information on factors associated with constipation should be offered to women for the prevention of postpartum constipation.	Recommended
	**13.** Routine use of laxatives for the prevention of postpartum constipation is not recommended.	Not recommended
**Prevention of maternal peripartum infection after uncomplicated vaginal birth**[4]	**14.** Routine antibiotic prophylaxis for women with uncomplicated vaginal birth is not recommended.	Not recommended
**Preventive anthelminthic treatment**[5]	**15.** Preventive chemotherapy (deworming), using annual or biannual[c] single-dose albendazole (400 mg) or mebendazole (500 mg), is recommended as a public health intervention for all non-pregnant adolescent girls and women of reproductive age, including postpartum and/or lactating women, living in areas where the baseline prevalence of any soil-transmitted helminth infection is 20% or more among adolescent girls and women of reproductive age, in order to reduce the worm burden of soil-transmitted helminths.	Context-specific recommendation

c Biannual administration is recommended where the baseline prevalence exceeds 50%.

4 Integrated from the 2015 *WHO recommendations for prevention and treatment of maternal peripartum infections.*

5 Adapted and integrated from the 2017 WHO guideline *Preventive chemotherapy to control soil-transmitted helminth infections in at-risk population groups.*

Care category	Recommendation	Category of recommendation
**Preventive schistosomiasis treatment**[6]	**16a.** In endemic communities with *Schistosoma* spp. prevalence of 10% or higher, WHO recommends annual preventive chemotherapy with praziquantel in a single dose for ≥ 75% up to 100% of pregnant women after the first trimester, and non-pregnant adolescent girls and women of reproductive age, including postpartum and/or lactating women, to control schistosomiasis morbidity and move towards eliminating the disease as a public health problem.	Context-specific recommendation
	**16b.** In endemic communities with *Schistosoma* spp. prevalence of less than 10%, WHO suggests one of two approaches based on the programmes' objectives and resources: (i) where there has been a programme of regular preventive chemotherapy, continuing preventive chemotherapy at the same or a reduced frequency towards interruption of transmission; and (ii) where there has not been a programme of regular preventive chemotherapy, a clinical approach of test-and-treat, instead of preventive chemotherapy targeting a population.	Context-specific recommendation
**Oral pre-exposure prophylaxis for HIV prevention**[7]	**17.** Oral pre-exposure prophylaxis (PrEP) containing tenofovir disoproxil fumarate (TDF) should be started or continued as an additional prevention choice for postpartum and/or lactating women at substantial risk[d] of HIV infection as part of combination HIV prevention approaches.	Context-specific recommendation
**Mental health interventions**		
**Screening for postpartum depression and anxiety**	**18.** Screening for postpartum depression and anxiety using a validated instrument is recommended and should be accompanied by diagnostic and management services for women who screen positive.	Recommended
**Prevention of postpartum depression and anxiety**	**19.** Psychosocial and/or psychological interventions during the antenatal and postnatal period are recommended to prevent postpartum depression and anxiety.	Recommended
**Nutritional interventions and physical activity**		
**Postpartum oral iron and folate supplementation**[8]	**20.** Oral iron supplementation, either alone or in combination with folic acid supplementation, may be provided to postpartum women for 6–12 weeks following childbirth for reducing the risk of anaemia in settings where gestational anaemia is of public health concern.[e]	Context-specific recommendation
**Postpartum vitamin A supplementation**[9]	**21.** Vitamin A supplementation in postpartum women for the prevention of maternal and infant morbidity and mortality is not recommended.	Not recommended

d Substantial risk is provisionally defined as HIV incidence greater than 3 per 100 person-years in the absence of PrEP.
e WHO considers a 20% or higher population prevalence of gestational anaemia to be a moderate public health problem.

6 Adapted and integrated from the 2022 *WHO guideline on control and elimination of human schistosomiasis.*
7 Adapted and integrated from the 2016 *Consolidated guidelines on the use of antiretroviral drugs for treating and preventing HIV infection: Recommendations for a public health approach – Second edition.*
8 Integrated from the 2016 WHO publication *Iron supplementation in postpartum women.*
9 Integrated from the 2011 WHO publication *Vitamin A supplementation in postpartum women.*

Care category	Recommendation	Category of recommendation
**Physical activity and sedentary behaviour**[10]	**22.** All postpartum women without contraindication should:<br>• undertake regular physical activity throughout the postpartum period;<br>• do at least 150 minutes of physical activity throughout the week for substantial health benefits; and<br>• incorporate a variety of physical and muscle-strengthening activities; adding gentle stretching may also be beneficial.	Recommended
	**23.** Postpartum women should limit the amount of time spent being sedentary. Replacing sedentary time with physical activity of any intensity (including light intensity) provides health benefits.	Recommended
**Contraception**		
**Postpartum contraception**[11]	**24.** Provision of comprehensive contraceptive information and services during postnatal care is recommended.	Recommended
**B. NEWBORN CARE**		
**Newborn assessment**		
**Assessment of the newborn for danger signs**[12]	**25.** The following signs should be assessed during each postnatal care contact, and the newborn should be referred for further evaluation if any of the signs is present: not feeding well; history of convulsions; fast breathing (breathing rate > 60 per minute); severe chest in-drawing; no spontaneous movement; fever (temperature > 37.5 °C); low body temperature (temperature < 35.5 °C); any jaundice in first 24 hours after birth, or yellow palms and soles at any age.<br><br>The parents and family should be encouraged to seek health care early if they identify any of the above danger signs between postnatal care visits.	Recommended
**Universal screening for abnormalities of the eye**	**26.** Universal newborn screening for abnormalities of the eye is recommended and should be accompanied by diagnostic and management services for children identified with an abnormality.	Recommended
**Universal screening for hearing impairment**	**27.** Universal newborn hearing screening (UNHS) with otoacoustic emissions (OAE) or automated auditory brainstem response (AABR) is recommended for early identification of permanent bilateral hearing loss (PBHL). UNHS should be accompanied by diagnostic and management services for children identified with hearing loss.	Recommended
**Universal screening for neonatal hyperbilirubinaemia**	**28.** Universal screening for neonatal hyperbilirubinaemia by transcutaneous bilirubinometer (TcB) is recommended at health facility discharge.	Recommended
	**29.** There is insufficient evidence to recommend for or against universal screening by total serum bilirubin (TSB) at health facility discharge.	No recommendation issued
**Preventive measures**		
**Timing of first bath to prevent hypothermia and its sequelae**	**30.** The first bath of a term, healthy newborn should be delayed for at least 24 hours after birth.	Recommended
**Use of emollients for the prevention of skin conditions**	**31.** Routine application of topical emollients in term, healthy newborns for the prevention of skin conditions is not recommended.	Not recommended

10 Adapted and integrated from the 2020 *WHO guidelines on physical activity and sedentary behaviour.*

11 Adapted and integrated from the 2013 WHO document *Ensuring human rights in the provision of contraceptive information and services: guidance and recommendations.*

12 Adapted and integrated from the 2014 *WHO recommendations on postnatal care of the mother and newborn.*

Care category	Recommendation	Category of recommendation
**Application of chlorhexidine to the umbilical cord stump for the prevention of neonatal infection**	**32a.** Clean, dry umbilical cord care is recommended.	Recommended
	**32b.** Daily application of 4% chlorhexidine (7.1% chlorhexidine digluconate aqueous solution or gel, delivering 4% chlorhexidine) to the umbilical cord stump in the first week after birth is recommended only in settings where harmful traditional substances (e.g. animal dung) are commonly used on the umbilical cord.	Context-specific recommendation
**Sleeping position for the prevention of sudden infant death syndrome**	**33.** Putting the baby to sleep in the supine position during the first year is recommended to prevent sudden infant death syndrome (SIDS) and sudden unexpected death in infancy (SUDI).	Recommended
**Immunization for the prevention of infections**[13]	**34.** Newborn immunization should be promoted as per the latest existing WHO recommendations for routine immunization.	Recommended
**Nutrition interventions**		
**Neonatal vitamin A supplementation**	**35a.** Routine neonatal vitamin A supplementation is not recommended to reduce neonatal and infant mortality.	Not recommended
	**35b.** In settings with recent (within the last five years) and reliable data that indicate a high infant mortality rate (greater than 50 per 1000 live births)[f] and a high prevalence of maternal vitamin A deficiency (≥ 10% of pregnant women with serum retinol concentrations < 0.70 µmol/L), providing newborns with a single oral dose of 50 000 IU of vitamin A within the first three days after birth may be considered to reduce infant mortality.	Context-specific recommendation
**Vitamin D supplementation for breastfed, term infants**	**36.** Vitamin D supplementation in breastfed, term infants is recommended for improving infant health outcomes only in the context of rigorous research.	Context-specific recommendation
**Infant growth and development**		
**Whole-body massage**	**37.** Gentle whole-body massage may be considered for term, healthy newborns for its possible benefits to growth and development.	Recommended
**Early childhood development**[14]	**38.** All infants and children should receive responsive care between 0 and 3 years of age; parents and other caregivers should be supported to provide responsive care.	Recommended
	**39.** All infants and children should have early learning activities with their parents and other caregivers between 0 and 3 years of age; parents and other caregivers should be supported to engage in early learning with their infants and children.	Recommended
	**40.** Support for responsive care and early learning should be included as part of interventions for optimal nutrition of newborns, infants and young children.	Recommended
	**41.** Psychosocial interventions to support maternal mental health should be integrated into early childhood health and development services.	Recommended

f The proposed infant mortality rate of greater than 50 per 1000 live births was calculated based on several assumptions: 50% of the total infant mortality rate are neonatal deaths; 50% of neonatal mortality occurs within the first day after birth; the post-neonatal mortality rate up to 6 months of age makes up two thirds of the total infant mortality rate, and the mortality rate between 6 and 12 months of age makes up the remaining one third; the rate of 30 deaths per 1000 used in the studies accounts for deaths between enrolment in the study up to 6 months of age; and dosing/enrolment almost always occurred within the first 24 hours after birth.

13 Adapted and integrated from the 2013 *WHO recommendations on postnatal care of the mother and newborn*.

14 Adapted and/or integrated from the 2020 *Improving early childhood development: WHO guideline*.

Care category	Recommendation	Category of recommendation
**Breastfeeding**		
**Exclusive breastfeeding**[15]	**42.** All babies should be exclusively breastfed from birth until 6 months of age. Mothers should be counselled and provided with support for exclusive breastfeeding at each postnatal contact.	Recommended
**Protecting, promoting and supporting breastfeeding in facilities providing maternity and newborn services**[16]	**43a.** Facilities providing maternity and newborn services should have a clearly written breastfeeding policy that is routinely communicated to staff and parents.	Recommended
	**43b.** Health-facility staff who provide infant feeding services, including breastfeeding support, should have sufficient knowledge, competence and skills to support women to breastfeed.	Recommended

C. HEALTH SYSTEMS AND HEALTH PROMOTION INTERVENTIONS		
**Schedules for postnatal care contacts**	**44.** A minimum of four postnatal care contacts is recommended.<br>If birth is in a health facility, healthy women and newborns should receive postnatal care in the facility for at least 24 hours after birth. If birth is at home, the first postnatal contact should be as early as possible within 24 hours of birth. At least three additional postnatal contacts are recommended for healthy women and newborns, between 48 and 72 hours, between 7 and 14 days, and during week six after birth.	Recommended
**Length of stay in health facilities after birth**	**45.** Care for healthy women and newborns in the health facility is recommended for at least 24 hours after vaginal birth.	Recommended
**Criteria to be assessed prior to discharge from the health facility after birth**	**46.** Prior to discharging women and newborns after birth from the health facility to the home, health workers should assess the following criteria to improve maternal and newborn outcomes:<br>• the woman's and baby's physical well-being and the woman's emotional well-being;<br>• the skills and confidence of the woman to care for herself and the skills and confidence of the parents and caregivers to care for the newborn; and<br>• the home environment and other factors that may influence the ability to provide care for the woman and the newborn in the home, and care-seeking behaviour.	Recommended
**Approaches to strengthen preparation for discharge from the health facility to home after birth**	**47.** Information provision, educational interventions and counselling are recommended to prepare women, parents and caregivers for discharge from the health facility after birth to improve maternal and newborn health outcomes, and to facilitate the transition to the home. Educational materials, such as written/digital education booklets, pictorials for semi-literate populations and job aids should be available.	Recommended
**Home visits for postnatal care contacts**	**48.** Home visits during the first week after birth by skilled health personnel or a trained community health worker are recommended for the postnatal care of healthy women and newborns. Where home visits are not feasible or not preferred, outpatient postnatal care contacts are recommended.	Recommended

15 Integrated from the 2014 *WHO recommendations on postnatal care of the mother and newborn.*

16 Integrated from the 2017 WHO guideline *Protecting, promoting and supporting breastfeeding in facilities providing maternity and newborn services.*

Care category	Recommendation	Category of recommendation
**Midwifery continuity of care**[17]	**49.** Midwife-led continuity-of-care (MLCC) models, in which a known midwife or small group of known midwives supports a woman throughout the antenatal, intrapartum and postnatal continuum, are recommended for women in settings with well-functioning midwifery programmes.	Context-specific recommendation
**Task sharing components of postnatal care delivery**[18]	**50a.** Task sharing the promotion of health-related behaviours for maternal and newborn health[g] to a broad range of cadres, including lay health workers, auxiliary nurses, nurses, midwives and doctors, is recommended.	Recommended
	**50b.** Task sharing the provision of recommended postpartum contraception methods[h] to a broad range of cadres, including auxiliary nurses, nurses, midwives and doctors, is recommended.	Recommended
**Recruitment and retention of staff in rural and remote areas**[19]	**51.** Policy-makers should consider a bundle of interventions covering education, regulation, incentives and personal and professional support to improve health workforce development, attraction, recruitment and retention in rural and remote areas.	Recommended
**Involvement of men in postnatal care and maternal and newborn health**[20]	**52.** Interventions to promote the involvement of men during pregnancy, childbirth and after birth are recommended to facilitate and support improved self-care of women, home care practices for women and newborns, and use of skilled care for women and newborns during pregnancy, childbirth and the postnatal period, and to increase the timely use of facility care for obstetric and newborn complications.<br><br>These interventions are recommended, provided they are implemented in a way that respects, promotes and facilitates women's choices and their autonomy in decision-making, and that supports women in taking care of themselves and their newborns.	Recommended with targeted monitoring and evaluation
**Home-based records**[21]	**53.** The use of home-based records, as a complement to facility-based records, is recommended for the care of pregnant and postpartum women, newborns and children, to improve care-seeking behaviour, men's involvement and support in the household, maternal and child home care practices, infant and child feeding, and communication between health workers and women, parents and caregivers.	Recommended
**Digital targeted client communication**[22]	**54.** WHO recommends digital targeted client communication for behaviour change regarding sexual, reproductive, maternal, newborn and child health, under the condition that concerns about sensitive content and data privacy are adequately addressed.	Context-specific recommendation

g Including promotion of the following: postnatal care, family planning (distribution of condoms [male and female] and other barrier methods, initiation and distribution of combined oral contraceptives, progestin-only oral contraceptives and emergency contraception, and information and general instructions on the Standard Days Method, TwoDay Method® and the lactational amenorrhoea method), postpartum HIV catch-up testing and retesting, sleeping under insecticide-treated nets, nutritional advice, nutritional supplements, basic newborn care, exclusive breastfeeding and immunization according to national guidelines.

h Including: initiate and maintain injectable contraceptives using a standard syringe with needle for intramuscular or subcutaneous injection, insertion of intrauterine device (IUDs), insertion of contraceptive implants.

17 Integrated from the 2016 *WHO recommendations on antenatal care for a positive pregnancy experience*.

18 Adapted and integrated from the 2012 WHO publication *Optimizing health worker roles to improve access to key maternal and newborn health interventions through task shifting*.

19 Adapted and integrated from the updated 2021 *WHO guideline on health workforce development, attraction, recruitment and retention in rural and remote areas*.

20 Retained (following review of new evidence) from the 2015 *WHO recommendations on health promotion interventions for maternal and newborn health*.

21 Adapted and integrated from the 2018 *WHO recommendations on home-based records for maternal, newborn and child health*.

22 Integrated from the 2019 *WHO guideline: recommendations on digital interventions for health system strengthening*.

Care category	Recommendation	Category of recommendation
**Digital birth notifications**[23]	**55.** WHO recommends the use of digital birth notifications under these conditions:<br>• in settings where the notifications provide individual-level data to the health system and/or a civil registration and vital statistics (CRVS) system;<br>• the health system and/or CRVS system has the capacity to respond to the notifications.	Context-specific recommendation

Implementation

These recommendations need to be delivered within an appropriate model of postnatal care, and adapted to the needs of different countries, local contexts, and individual women, newborns, parents, caregivers and families. The GDG proposed implementation considerations for each of the new and/or updated recommendations, and overall considerations for the adoption, adaptation and implementation of the set of recommendations to ensure respectful, individualized, person-centred care at every contact, in accordance with a human rights-based approach.

The WHO postnatal care model places the woman–newborn dyad at the centre of care. The foundation of this postnatal care model is a minimum of four postnatal care contacts. In particular, the GDG considered the first two weeks after birth to be a key time to promote health, identify health problems, and support the transition to well-women and well-infant care. This current guideline confirms the importance of postnatal care during the first 24 hours after birth, regardless of the place of birth. More specifically, it recommends a minimum 24-hour stay after birth in the health facility, with continuous care and monitoring during that stay. Expanded criteria before discharge have been identified to assess and manage potential problems and to prepare for the transition to the home. At least three additional postnatal care contacts occur during the first six weeks after birth. This includes the provision of effective clinical practices, relevant and timely information, and psychosocial and emotional support, provided by kind, competent and motivated health workers who are working within a well-functioning health system. An effective referral system, including communication between facility- and community-based care providers, and between health and transport workers in case of complications, are also essential components of this postnatal care model.

Monitoring and evaluation

The implementation and impact of these recommendations will be monitored at the health service, sub-national and national levels, based on clearly defined criteria and indicators that are associated with locally agreed targets. The GDG suggests the following indicators to be considered, which have been adapted from current global recommended indicators.[24]

- Length of stay in health facilities after childbirth
- Early routine postnatal care for women (within two days)
- Early routine postnatal care for newborns (within two days)
- Hepatitis B birth dose vaccination

Updating of the recommendations

In accordance with the procedures for updating WHO guidelines, a systematic and continuous process of identifying and bridging evidence gaps following guideline dissemination will be employed. If new evidence that could potentially impact the current evidence base for any of the recommendations is identified, the recommendation will be updated. WHO welcomes suggestions regarding additional questions for inclusion in future updates of the guideline.

23 Integrated from the 2019 *WHO guideline: recommendations on digital interventions for health system strengthening*.
24 WHO maternal, newborn, child and adolescent health and ageing data portal: www.who.int/data/maternal-newborn-child-adolescent-ageing/maternal-and-newborn-data/maternal-and-newborn---coverage.

1. Introduction

1.1 Background

Global strategies, including the Global Strategy for Women's, Children's and Adolescents' Health (2016–2030) *(1)*, Strategies toward Ending Preventable Maternal Mortality *(2)*, the Every Newborn Action Plan *(3)* and other initiatives *(4, 5)* recognize the postnatal period, defined here as beginning immediately after the birth of the baby and extending up to six weeks (42 days) after birth *(6)*, as a critical time for women and newborns, partners, parents, caregivers and families. Despite these efforts, the burden of maternal and neonatal mortality and morbidity in the postnatal period is still high *(7, 8)*. Up to 30% of maternal deaths occur postpartum *(8)*. Infants face a high risk of dying in their first month after birth, with an average global rate of 17 deaths per 1000 live births in 2019 *(9, 10)*.

Yet coverage and quality of postnatal care for women and newborns tend to be relatively poor, and opportunities to increase well-being and support nurturing newborn care are lost. Length of stay in the health facility after birth varies widely across countries and substantial proportions of women and newborns receive inadequate postnatal care during the first 24 hours after childbirth *(11)*. The median coverage for routine postnatal care within two days after birth for women (71%) and newborns (64%) *(12)* still lags behind global targets for 2025 *(13)*.

In line with the Sustainable Development Goals (SDGs) *(14)* and the Global Strategy for Women Children and Adolescent Health *(1)*, the focus of the global agendas has now expanded beyond maternal and newborn survival, to also ensure that women and babies thrive and achieve their full potential for health and well-being, in accordance with a human rights-based approach. Quality of care throughout the pregnancy, childbirth and the postnatal periods is critical to the operationalization of these global agendas and the achievement of the health-related SDGs.

Postnatal care services are a fundamental component of this continuum. These services provide the platform for the care of women after childbirth and newborns, including the promotion of healthy practices, disease prevention, and detection and management of problems during the first six weeks after birth. Postnatal care aims at maintaining and promoting the health and well-being of women and children from birth, and fostering an environment that offers help and support to women, parents, caregivers and families for a wide range of health, social and developmental needs.

WHO published recommendations on postnatal care for mothers and newborns in 2014 *(15)*. However, in view of new recommendations from different WHO departments published after 2014 and the availability of new evidence related to the organization of care and interventions for better quality postnatal care, it is important that recommendations for postnatal care are reviewed, updated and consolidated accordingly. This guideline consolidates current guidance for effective and safe clinical and non-clinical interventions, as well as health systems and health promotion interventions for essential care during the postnatal period, to improve the quality (provision and experience) of postnatal care for women and newborns. It recognizes a "positive postnatal experience" as a significant end point for all women giving birth and their newborns, laying the platform for improved short- and long-term health and well-being. A positive postnatal experience is defined as one in which women, newborns, partners, parents, caregivers and families receive information and reassurance delivered in a consistent manner by motivated health workers. Both the women's and babies' needs are recognized, within a resourced and flexible health system that respects their cultural context.

1.2 Target audience

The recommendations in this guideline are intended to inform the development of relevant national and subnational health policies, clinical protocols and programmatic guides. Therefore, the target audience includes national and subnational public health policy-makers, implementers and managers of maternal, newborn and child health programmes, health facility managers, health workers (including midwives, auxiliary nurse-midwives, nurses, obstetricians, paediatricians, neonatologists, general medical practitioners, community health workers), nongovernmental organizations, professional societies involved in the planning and management

of maternal, newborn and child health services, academic staff involved in training health workers and women and parents' groups.

1.3 Scope of the guideline

This guideline is relevant for the care of all women and adolescent girls in the postpartum period, and newborns in any health facility or community-based setting, unless otherwise indicated in the recommendation. Based on the premise that all women and newborns deserve high-quality care, the guideline focuses on the core, essential postnatal care package. For the purpose of this guideline, the term "healthy women and newborns" is used to describe women or adolescent girls after childbirth and their newborns who have no apparent risk factors or illness, and who otherwise appear to be healthy. Some women and their newborns can have additional health and social needs that are not covered in this guideline, including in the case of death of the woman or baby. Individuals with additional needs may also include adolescent girls and those from priority groups, including, among others, those living in rural settings, those facing financial hardship, those from ethnic, religious and racial minorities, migrant and displaced or war-affected individuals, unmarried women and girls, survivors of sexual- and gender-based violence, surrogates, sex workers, transgender or nonbinary individuals, those with disabilities or mental health conditions, and those living with HIV. Additional management of women or newborns who develop complications in the postnatal period, those with high-risk pregnancies, those who presented with complications during labour and childbirth, or who require specialized postnatal care, are also not included in this guideline.

This guideline is therefore complementary to existing WHO guidance on the immediate care of the woman and newborn after birth, management of complications during pregnancy, childbirth and the postnatal period and care of preterm and low birthweight infant. Together with the WHO recommendations on antenatal care for a positive pregnancy experience *(16)* and the WHO recommendations on intrapartum care for a positive childbirth experience *(17)*, WHO has provided a set of integrated recommendations to address quality essential respectful care along the pregnancy to postnatal continuum.

This is a consolidated guideline of new and existing WHO recommendations on postnatal care for women and newborns receiving facility- or community-based postnatal care in any resource setting. It provides a comprehensive set of recommendations for care during the postnatal period, focusing on the essential package that all women and newborns should receive, paying due attention to quality of care. This guideline updates and expands upon the 2014 *WHO recommendations on postnatal care of the mother and newborn (15)*, and complements existing WHO guidelines on the management of postnatal complications.

The priority questions and outcomes that guided evidence synthesis and decision-making for this guideline are listed in Web Annex 1 and cover essential care that should be provided during the postnatal care period, including maternal and newborn assessments, interventions for common physiological symptoms, preventive measures, maternal mental health assessment and interventions, contraception, nutritional interventions, infant growth and development, breastfeeding, health systems, and health promotion interventions (to improve provision, utilization and experience of postnatal care). The priority questions and outcomes for existing WHO recommendations that have been integrated into this guideline can be found in the respective guidelines.

The terms woman, mother, partner, parents and caregivers have been used in different combinations throughout the guideline. The terms "woman" or "mother" include individuals who have given birth, even if they may not identify as a woman or as a mother. To be concise and to facilitate readability, the term "woman" is generally used, but sometimes "mother" is used when referring to the woman in relation to her newborn. It is recognized that there are those who have given birth, but identify as gender diverse individuals. Also, different types of couples and families exist. The term "partner" is used to refer to the woman's chosen supporter, such as the woman's partner, spouse/husband and/or the baby's father. The term "parents" refers to those responsible for caring for the newborn. This will often be the mother and the father, but may include single parents, co-parents, same sex parents or parents with gender diverse identities. The term "caregiver" recognizes the different persons or family members who may be responsible for the care of the newborn. Families can be an important support for women and parents and have a vital role in maternal and newborn health. For brevity, we have not mentioned these term consistently throughout the document *(18)*.

2. Methods

This document was developed using the standard operating procedures described in the *WHO handbook for guideline development (19)*. In summary, the development process included: (i) identifying priority questions and outcomes; (ii) retrieval of the evidence; (iii) assessment and synthesis of the evidence; (iv) formulation of the recommendations, and (v) planning for the dissemination, implementation, impact evaluation and updating of the guideline.

2.1 Contributors to the guideline

The different groups involved in the development of the guideline are described below. The members of these groups are listed in Annex 1.

2.1.1 WHO Steering Group

The guideline development process was supervised by the WHO Steering Group, comprising staff members from the Departments of Maternal, Newborn, Child and Adolescent Health and Ageing (MCA), Mental Health and Substance Use (MSD), Nutrition and Food Safety (NFS) and Sexual and Reproductive Health and Research (SRH). The group drafted the initial scope of the guideline, identified priority questions and outcomes, prepared the guideline planning proposal, and identified systematic review teams, guideline methodologists and members of the Guideline Development Group (GDG). Additionally, the Steering Group supervised the evidence retrieval, assessment and synthesis, organized the GDG meetings, prepared draft recommendations for the GDG and the final document, and managed the guideline publication and dissemination.

2.1.2 Guideline Development Group

The WHO Steering Group identified 21 external experts and stakeholders from the six WHO regions to form the GDG. This was a diverse group of individuals with expertise in research, clinical practice, policy and programmes, guideline development methods relating to postnatal care practices and service delivery, and patient/consumer representatives. The members were identified in a way that ensured geographic representation and gender balance with no important conflicts of interest. Selected members of this group participated in a scoping meeting held in April 2019 and provided input into the priority questions and outcomes that guided the evidence reviews. The GDG examined and interpreted the evidence and formulated the final recommendations at nine virtual meetings between September 2020 and June 2021. The group also reviewed and approved the final guideline document.

2.1.3 External Review Group

This group included six technical experts and stakeholders with an interest in the provision and experience of evidence-based postnatal care. The group was geographically balanced and gender-representative, and had no important conflicts of interest. The External Review Group (ERG) peer-reviewed the final document to identify any errors of fact and comment on clarity of language, contextual issues and implications for implementation. The group ensured that the guideline decision-making processes considered and incorporated the contextual values and preferences of persons affected by the recommendations, including postpartum women, partners, newborns, parents, caregivers and families, health workers and managers, and policy-makers. It was not within the remit of this group to change recommendations that were formulated by the GDG.

2.1.4 Technical Working Group

The Technical Working Group (TWG) comprised guideline methodologists and systematic review teams. Independent consultants and technical experts from Centro Rosarino de Estudios Perinatales (CREP), Argentina, served as guideline methodologists. In relation to quantitative evidence on the effects of different prioritized interventions, the Cochrane Pregnancy and Childbirth Group (PCG), provided input on the scoping of the guideline priority questions and supervised the updating of relevant systematic reviews related to maternal health clinical guidance, following the standard processes of Cochrane. Where there were no suitable systematic reviews (Cochrane or non-Cochrane) for priority questions, new systematic reviews of quantitative studies were commissioned by WHO from external experts. Additional systematic reviews were conducted for priority questions and other

considerations relevant to the domains of the Grading of Recommendations Assessment, Development and Evaluation (GRADE) evidence-to-decision (EtD) frameworks, including quantitative and qualitative reviews. The WHO Steering Group worked closely with members of the TWG to develop or update review protocols, review and appraise the evidence and prepare the GRADE EtD frameworks.

2.1.5 External partners and observers

Representatives of the Bill & Melinda Gates Foundation, International Federation of Gynecology and Obstetrics (FIGO), International Confederation of Midwives (ICM), International Pediatric Association (IPA), United Nations Children's Fund (UNICEF), United Nations Population Fund (UNFPA) and United States Agency for International Development (USAID) were invited to the guideline development meetings as observers. These organizations are potential implementers of the guideline with a history of collaboration with WHO in guideline dissemination and implementation. Observers were allowed to make comments during technical discussions at selected times during the GDG meetings. Observers were, however, asked to refrain from participation in discussions on the final recommendations.

2.2 Identifying priority questions and outcomes

The WHO Steering Group, in consultation with the systematic review teams, guideline methodologists and selected members of the GDG, drafted the priority questions for this guideline (see Web Annex 1 for detailed methods and the final list). From the priority questions identified, the associated interventions were then classified according to the WHO quality of care framework *(20)* and the nurturing care framework *(5)* to ensure the recommendations would respond to a maximum of domains, including: quality of care (provision and experience of care); nurturing care (health, nutrition, security and safety, responsive caregiving and early learning); and strengthening health systems. Changes from the approved scope of this guideline and the reasons for such changes are described in Web Annex 2.

Discussion of the key thematic areas for essential, routine postnatal care took account of interventions that are already covered in existing WHO guidelines. Considering available resources, the group agreed to limit the scope of prioritized questions to those not addressed by existing WHO guidelines or those identified for update, with the caveat that existing recommendations (that were developed according to WHO standard procedures) would be integrated into the final guideline document (see section 2.3).

In determining the guideline focus, the scoping process highlighted the need to identify person-centred interventions and outcomes for postnatal care. To this end, a qualitative evidence synthesis was conducted to understand what women want, need and value during the postnatal period *(21)*. The findings of this review suggest that the postnatal phase is a period of significant transition characterized by changes in self-identity, the redefinition of relationships, opportunities for personal growth, and alterations to sexual behaviour as women adjust to their new normal, both as parents and as individuals within their own cultural context. For many women, it is also marked by feelings of intense joy, happiness and love for the new baby. The definition of a positive postnatal experience has therefore been adapted to also consider the experience of newborns, parents and the family more broadly (Box 2.1).

Box 2.1 Positive postnatal experience

A positive postnatal experience is defined as one in which women, partners, parents, caregivers and families receive information and reassurance in a consistent manner from motivated health workers. Both the women's and babies' health, social and developmental needs are recognized, within a resourced and flexible health system that respects their cultural context.

Adapted from Finlayson et al. *(21)* and Harvey et al. *(22)*

Based on the prioritization exercise described above, a set of outcomes that were considered as critical or as important to women and newborns was prioritized for the postnatal period (Web Annex 1). Furthermore, due to important differences between the types of prioritized interventions and the range of potential outcomes, and with due consideration of what matters to women, parents and caregivers in the postnatal period, the outcomes were prioritized separately for individual guideline questions. Informed initially by the qualitative review of women's views, the list of outcomes was complemented by outcomes related to maternal and family functioning, well-being and experience of postnatal care; it

therefore reflects perceptions of the quality of care for all interventions prioritized.

2.3 Integration of recommendations from published WHO guidelines

In order to harmonize and consolidate all recommendations that are relevant to the care of healthy women and babies during the postnatal period into a single document, existing WHO recommendations that were within the scope of essential, routine postnatal care, and which were previously approved by the Guideline Review Committee, were identified, presented to the GDG and integrated into this guideline. These include recommendations relevant to maternal and neonatal assessments, preventive measures, and health systems and health promotion interventions. In most instances, the recommendations were taken from the associated guideline without modification or revalidation, as these recommendations were considered to be current (see Web Annex 3). Such recommendations are indicated in the guideline text by specifying that the recommendation has been "integrated from" the specific guideline. Some recommendations required adaptation for the purposes of the postnatal care guideline; relevant WHO departments that produced the specific guidance were consulted to confirm that adaptations were feasible given the evidence base. Such recommendations are indicated in the guideline text by specifying that the recommendation has been "adapted and integrated from" the specific guideline.

2.4 Focus and approach

The focus of this guideline is on essential postnatal care, which all women and adolescents after birth and their newborns should receive to facilitate a positive postnatal experience. To help decision-makers consider a range of relevant criteria – including the benefits, harms, values, resources, equity, acceptability and feasibility, of each intervention – the GRADE EtD framework tool *(23)* was used. The preparatory work for the guideline was organized into the work streams outlined in Table 2.1, to synthesize and examine evidence across the domains of this framework.

Table 2.1 WHO postnatal care guideline work streams

Work streams	Methodology	Assessment of evidence
Individual interventions for clinical, health system-level and health promotion interventions	Systematic reviews of effectiveness or observational studies	GRADE
Woman-, partner-, parent-, caregiver-, family-, and health worker-centred domains for values, acceptability and feasibility of implementing interventions related to postnatal care	Qualitative evidence synthesis, and review of studies and references included in effectiveness reviews	GRADE-CERQual, as applicable
Equity issues related to postnatal care	Literature searches of systematic reviews or single studies, review of studies and references included in effectiveness reviews, and 2015 WHO State of Inequality report *(24)*	Not applicable
Resource implications for individual interventions	Literature searches of systematic reviews of cost-effectiveness or single-study economic evaluations on resource use/cost or cost-effectiveness, and review of studies and references included in effectiveness reviews; additional internet searches where required to complete the "Main resource requirements" tables	CHEC, as applicable

GRADE: Grading of Recommendations Assessment, Development and Evaluation *(25)*; CERQual: Confidence in the Evidence from Reviews of Qualitative research *(26)*; CHEC: Consensus Health Economic Criteria *(27)*.

2.5 Evidence identification and retrieval

Evidence on effects for maternal clinical practices was derived mainly from Cochrane systematic reviews of randomized controlled trials (RCTs). The WHO Steering Group, in collaboration with the Cochrane PCG and methodologists from CREP, first identified all relevant Cochrane systematic reviews that addressed the prioritized maternal clinical practice questions. The Cochrane systematic reviews were based on studies identified from searches of the Cochrane PCG Trials Register.[25] In instances where the Cochrane reviews identified were found to be out-of-date, review authors were invited to update their Cochrane reviews in accordance with the standard process of the Cochrane PCG and with the support of Cochrane PCG staff.

Where new systematic reviews were commissioned from external experts, experts were asked to prepare a standard protocol with a clear PICO (population, intervention, comparator, outcome) question, criteria for identification of studies including search strategies for different bibliographic databases, methods for assessing risk of bias, and a data analysis plan before embarking on the review. The protocols were reviewed and approved by members of the WHO Steering Group.

Qualitative reviews were commissioned from external experts on what women want from postnatal care and how the outcomes impacted by an intervention are valued by women *(21)*; women's views, attitudes and experiences of attending postnatal care *(28)*; health workers' views, attitudes and experiences on provision of postnatal care *(29)*; women's, men's and health workers' perspectives on the involvement of men in maternal and newborn health *(30)*; and women's, men's and health workers' perspectives on discharge preparation and readiness from health facilities after birth *(22)*. In each case, the external experts were asked to prepare a standard protocol with a clear research question, criteria for identification of studies (including search strategies for different bibliographic databases), methods for assessing quality, and a data analysis plan, before embarking on the review. The protocols were reviewed and approved by members of the WHO Steering Group.

Structured searches were carried out to identify evidence around cost-effectiveness and health equity related to the maternal and newborn health interventions. Intervention search terms were taken from the corresponding effectiveness reviews where supplied, or else were developed ad hoc. Cost-effectiveness search terms were adapted from the National Health Service Economic Evaluation Database filters made available by the InterTASC Information Specialists' Sub-Group Search Filter Resource.[26] Health equity search terms were developed with reference to published guidance *(31)*. Searches were carried out across Embase and Medline for publication dates from 2010 onwards, limited to human studies. In addition, the NHS EED database was searched for relevant economic evaluations. Where evidence on cost-effectiveness was synthesized as part of the effectiveness reviews used for specific interventions, additional structured searches were not conducted. To retrieve evidence on cost-effectiveness and health equity implications of the mental health and health systems and health promotion interventions, broad searches were performed on Google Scholar using key terms, such as "costs", "cost-effectiveness", "cost-benefit analysis", and "equity", combined with terms related to the PICO elements of the specific guideline questions (e.g. postpartum depression and screening). For all interventions, studies and references included in the systematic reviews of effectiveness, as well as qualitative evidence synthesis conducted for corresponding guideline questions (where available), were screened to identify further information on equity, resources and costs of the interventions, as well as references to relevant studies reporting on these implications.

2.6 Quality assessment and grading of the evidence

2.6.1 Quality assessment of primary studies included in the reviews

The assessment of the quality of individual studies included in Cochrane systematic reviews follows

25 The Cochrane PCG Trials Register is maintained by the PCG's Trial Search Coordinator and contains trials identified from: monthly searches of the Cochrane Central Register of Controlled Trials (CENTRAL); weekly searches of MEDLINE; weekly searches of Embase; hand-searches of 30 journals and the proceedings of major conferences; weekly "current awareness" alerts for a further 44 journals; and monthly BioMed Central email alerts. For further information, see: http://pregnancy.cochrane.org/pregnancy-and-childbirth-groups-trials-register.

26 The InterTASC Information Specialists' Sub-Group Search Filter Resource is available at: https://sites.google.com/a/york.ac.uk/issg-search-filters-resource/home.

a specific and explicit method of risk of bias assessment using six standard criteria outlined in the *Cochrane handbook for systematic reviews of interventions (32)*. Each included study is assessed and rated by reviewers to be at low, high or unclear risk of bias for sequence generation, allocation concealment, blinding of study personnel and participants, attrition, selective reporting and other sources of bias such as publication bias. The assessment along these domains provides an overall risk of bias that indicates the likely magnitude and direction of the bias and how it is likely to impact on the review findings. In the case of the new systematic reviews on the effectiveness of interventions commissioned by the WHO Steering Group, each included study was assessed for risk of bias according to the Cochrane review methodology for randomized or non-randomized studies. One review used the CASP (Critical Appraisal Skills Programme).[27]

Studies identified for the qualitative reviews related to what women want from postnatal care and to women's experiences of postnatal care were subjected to a simple, quality appraisal system using a validated instrument that rated studies against 11 pre-defined criteria, and then allocated a score from A to D, with D indicating the presence of significant flaws that are very likely to affect the credibility, transferability, dependability and/or confirmability of the study *(33)*. The other qualitative reviews used CASP or a modified CASP.

2.6.2 Grading of the review evidence

The GRADE approach to appraising the certainty of quantitative evidence *(25)* was used for all the critical outcomes identified in the PICO questions. For every priority question, a GRADE evidence profile was prepared for each quantitative outcome. Accordingly, the certainty of evidence for each outcome was rated as "high", "moderate", "low" or "very low" based on a set of criteria. As a baseline, RCTs provided "high-certainty" evidence, while non-randomized trials and observational studies provided "low-certainty" evidence. This baseline certainty rating was then downgraded based on consideration of study design limitations (risk of bias), inconsistency, imprecision, indirectness and publication bias. For observational studies, other considerations, such as magnitude of effect, could lead to upgrading of the rating if there were no limitations that indicated a need for downgrading. The systematic review teams and methodologists from CREP performed grading of quantitative review evidence, in accordance with standard operating procedures approved by the WHO Steering Group.

The findings of the qualitative reviews was appraised using the GRADE-CERQual (Confidence in the Evidence from Reviews of Qualitative research) tool *(26)*. The GRADE-CERQual tool, which uses a similar approach conceptually to other GRADE tools, provides a transparent method for assessing and assigning the level of confidence that can be placed in evidence from reviews of qualitative research. The confidence in qualitative review findings were assigned to evidence domains on values, acceptability and feasibility according to four components: methodological limitations of the individual studies, adequacy of data, coherence, and relevance to the review question of the individual studies contributing to a review finding.

Findings from individual cost-effectiveness studies were reported narratively for each comparison of interest, and evidence was assessed using the CHEC checklist *(27)*.

2.7 Formulation of the recommendations

The WHO Steering Group supervised and finalized the preparation of evidence profiles and evidence summaries in collaboration with the TWG using the GRADE DECIDE (Developing and Evaluating Communication Strategies to Support Informed Decisions and Practice Based on Evidence) EtD framework. This EtD tool includes explicit and systematic consideration of evidence on prioritized interventions in terms of specified domains: effects, values, resources, equity, acceptability and feasibility. For each priority question, judgements were made on the impact of the intervention on each domain (or criterion) to inform and guide the decision-making process. Using the EtD framework template, the WHO Steering Group and TWG created summary documents for each priority question covering evidence on each of these domains as described below.

- **Effects:** The evidence on the prioritized outcomes was summarized in this domain to answer the questions, *"What are the desirable and undesirable*

27 CASP critical appraisal tools are available at: https://casp-uk.net/casp-tools-checklists/.

effects of the intervention/option?" and *"What is the certainty of the evidence on effects?"*. Where benefits clearly outweighed harms for outcomes that are highly valued by pregnant women, or vice versa, there was a greater likelihood of a clear judgement in favour of or against the intervention, respectively. Uncertainty about the net benefits or harms, and small net benefits, most likely led to a judgement that neither favoured the intervention nor the comparator. The higher the certainty of evidence on benefits across outcomes, the higher the likelihood of a judgement in favour of the intervention. In the absence of evidence of benefits, evidence of potential harm led to a recommendation against the option. Where evidence of potential harm was found for interventions that were also found to have evidence of important benefits, depending on the level of certainty and likely impact of the harm, such evidence of potential harm was more likely to result to a context-specific recommendation for the intervention (and the context is explicitly stated within the recommendation).

- **Values:** This relates to the relative importance assigned to the outcomes of the intervention by those affected by them, how such importance varies within and across settings, and whether this importance is surrounded by any uncertainty. The question asked was, *"Is there important uncertainty or variability in how much women, parents and caregivers value the main outcomes associated with the intervention/option?"* Qualitative evidence from the different systematic reviews on women, men and health workers' views and experience across postnatal care informed the judgements for this domain. Interventions that resulted in outcomes that most women, parents and caregivers consistently value regardless of settings were more likely to lead to a judgement in favour of the intervention. This domain, together with the "effects" domain, informed the "balance of effects" judgement.

- **Resources required:** This domain addressed the questions, *"What are the resources associated with the intervention/option?"* and *"Is the intervention/option cost-effective?"*. Most resource requirements, in the context of implementing the reviewed postnatal care interventions, are the costs of providing supplies, training, equipment and skilled human resources. A judgement in favour or against the intervention was likely where the resource implications were clearly advantageous or disadvantageous, respectively. Cost evaluation relied on reported estimates obtained during the evidence retrieval process, a 2013 treatment assumption report *(34)*, the WHO compendium of innovative health technologies for low-resource settings *(35)*, and specific literature searches, as well as experiences and opinions of the GDG members. Where available, direct evidence from systematic reviews of cost-effectiveness informed this domain.

- **Acceptability:** This domain addressed the question, *"Is the intervention/option acceptable to key stakeholders?"*. Qualitative evidence from the different systematic reviews on women, men and health workers' views and experience across postnatal care informed the judgements for this domain. Relevant evidence yielded from the included trials and from the database searches pertaining to health equity and/or cost-effectiveness was considered where appropriate. The lower the acceptability, the lower the likelihood of a judgement in favour of the intervention. If it was deemed necessary to recommend an intervention that was associated with low acceptability, the recommendation is accompanied by a strategy to address concerns about acceptability during implementation.

- **Feasibility:** The feasibility of implementing an intervention depends on factors such as the resources available, infrastructure, and training requirements. This domain addressed the question, *"Is it feasible to implement the intervention/option by the relevant stakeholders?"*. Qualitative evidence from the systematic reviews on women, parents, caregivers and health workers' views and experiences across postnatal care was used to inform judgements for this domain. Again, relevant evidence yielded from the included trials and from the database searches pertaining to health equity and/or cost-effectiveness was considered where appropriate. Where barriers were identified, it was less likely that a judgement would be made in favour of the intervention.

- **Equity:** This domain included evidence or considerations as to whether or not an intervention would reduce health inequities and therefore addressed the question, *"What is the anticipated impact of the intervention/option on equity?"*. The domain was informed by the findings of qualitative

Box 2.2 Health equity – general considerations

The 2015 World WHO state of inequality report *(24)* indicates that women who are poor, least educated and who reside in rural areas have lower coverage of health interventions and worse health outcomes than more advantaged women. A systematic review and meta-analysis on inequities in postnatal care in low- and middle-income countries reported significant variation, by socioeconomic status and geographical determinants, in the use of postnatal care *(36)*.

evidence syntheses of women, parents and health workers' views and experiences, the 2015 WHO report on inequalities in reproductive, maternal, newborn and child health *(24)*, a systematic review and meta-analysis on inequities in postnatal care in low- and middle-income countries (LMICs) *(36)* (Box 2.2), and specific literature searches, as well as the experiences and opinions of the GDG members. An intervention was likely to be recommended if its proven (or anticipated) effects reduce (or could reduce) health inequalities among different groups of women, parents and families.

For each of the above domains, additional evidence of potential benefits, harms or unintended consequences was described in the subsection Additional considerations. Such considerations were derived from studies that might not have directly addressed the priority question but provided pertinent information in addition to the direct evidence. These were extracted from single studies, systematic reviews, or other relevant sources.

Given that virtual meetings were held over an extended period of time, the WHO Steering Group provided the EtD frameworks, including evidence summaries, GRADE evidence profiles, and other documents related to each recommendation, to GDG members in batches as soon as the documents were drafted, and in advance of the virtual GDG meetings. The GDG was asked to review and provide comments on the documents electronically before the GDG meetings, where possible. At the virtual meetings, under the leadership of the respective GDG chairs, GDG members collectively reviewed the EtD frameworks, the draft recommendations and any comments received through preliminary feedback. The purpose of the meeting was to reach consensus on each recommendation, including its direction and context, based on explicit consideration of all the domains within the EtD frameworks. In line with other recently published WHO guidelines using EtD frameworks *(16, 17)*, the GDG classified each recommendation into one of the categories defined below.

- *Recommended:* This category indicates that the intervention or option should be implemented.
- *Not recommended:* This category indicates that the intervention or option should not be implemented.
- *Recommended only in specific contexts:* This category indicates that the intervention or option is applicable only to the condition, setting or population specified in the recommendation, and should only be implemented in these contexts.
- *Recommended only in the context of rigorous research:* This category indicates that there are important uncertainties about the intervention or option. In such instances, implementation can still be undertaken on a large scale, provided that it takes the form of research that is able to address unanswered questions and uncertainties related both to the effectiveness of the intervention or option, and its acceptability and feasibility.
- *Recommended with targeted monitoring and evaluation:* This category indicates that there are important uncertainties about the intervention being applicable to all contexts or about the net impact of the evidence across all the domains, including acceptability or feasibility. In such instances, implementation can still be undertaken on a large scale, provided it is accompanied by monitoring and evaluation.

For recommendations integrated from existing guidelines, the strength and certainty of the evidence, if specified in the source document, has been presented in the accompanying remarks. For consistency, integrated recommendations were also categorized according to the typology described above.

During the formulation of recommendations, the GDG identified important research gaps. Where the certainty of available evidence was rated as "low" or "very low", the GDG considered whether further research should be prioritized, based on whether such research would contribute to improvements in

postnatal care of women and newborns, be likely to promote equity, and be feasible to implement. The prioritized research gaps are listed in Web Annex 4.

2.8 Decision-making during the GDG meetings

The GDG meetings were designed to allow participants to discuss the supporting evidence in all the domains of the EtD, and to agree on each of the recommendations drafted by the WHO Steering Group. As needed, each of these recommendations was revised through a process of group discussion. The final adoption of each recommendation was made by consensus – defined as the agreement by three quarters or more of the participants – provided that those who disagreed did not feel strongly about their position. All disagreements were resolved during the meetings and subsequent exchanges with the GDG members. No strong disagreements were recorded. If participants had been unable to reach a consensus, the disputed recommendation, or any other decision, would have been put to a vote in accordance with the procedures described in the *WHO handbook for guideline development (19)*. Where required, the GDG determined the context of recommendations by the same process of consensus, based on discussions around the balance of evidence on the benefits and disadvantages of the interventions across different contexts, in the context of rigorous research or targeted monitoring and evaluation.

2.9 Declaration of interests by external contributors

In accordance with WHO procedures for declaration of interests (DOIs) *(37)*, all GDG, TWG and ERG members and other external collaborators were asked to declare in writing any competing interests (whether academic, financial or other) using the standard WHO form, before engaging in the guideline development process. All experts were instructed to notify the responsible technical officer of any change in relevant interests during the course of the process, in order to update and review conflicts of interest accordingly. In addition, experts were requested to submit an electronic copy of their curriculum vitae.

The WHO Steering Group reviewed all DOI forms and curriculum vitae, and determined whether a conflict of interest existed. All findings from the received DOI forms were managed in accordance with the WHO DOI guidelines on a case-by-case basis. To ensure consistency, the WHO Steering Group applied the criteria for assessing the severity of a conflict of interest in the *WHO handbook for guideline development (19)*.

No declared conflicts of interest were considered serious enough to pose any risk to the guideline development process or reduce its credibility, and therefore all experts were only required to declare such conflicts at the first GDG meeting. At each subsequent virtual GDG meeting, members were required to share any new conflict of interest with the group. Prior to the final virtual GDG meeting, all GDG and TWG members, and observers, were again asked to complete their DOI forms and declare any conflict at the meeting, to ensure information was up-to-date as the formulation of recommendations concluded. Conflicts of interest that warranted action by WHO staff arose where experts had performed primary research or a systematic review related to any guideline recommendations; in such cases, the experts were restricted from participating in discussions and/or formulating any recommendation related to the area of their conflict of interest. A summary of DOIs from the GDG and information on how conflicts of interest were managed are included in Annex 2.

The names and short biographies of the GDG members were published on the WHO website for public review and comment two weeks prior to the first GDG meeting.

2.10 Document preparation and peer review

Following the final GDG meeting, an independent consultant and the WHO responsible technical officers prepared a draft of the full guideline document to accurately reflect the deliberations and decisions of the GDG. Other members of the WHO Steering Group provided comments on the draft guideline document before it was sent electronically to the GDG members for further comments. The document was also sent to the ERG for peer review. The ERG members were asked to review the final draft guideline to identify errors of fact, comment on clarity of language, and express considerations related to implementation, adaptation

and contextual issues. The WHO Steering Group carefully evaluated the input of the GDG and peer reviewers for inclusion in the guideline document and made further revisions to the guideline draft as needed. After the GDG meetings and external peer review, further modifications to the guideline by the WHO Steering Group were limited to corrections of factual errors and improvements in language to address any lack of clarity.

2.11 Presentation of guideline content

A summary of the recommendations is presented in the executive summary of this guideline. For each recommendation, a summary of the evidence on effects, values, resources, equity, acceptability, feasibility, and other considerations reviewed at the virtual GDG meetings can be found in Chapter 3 (Evidence and recommendations). The language used to interpret the evidence on effects is consistent with the Cochrane Effective Practice and Organization of Care approach *(38)*. Implementation of the postnatal care guideline and recommendations is discussed in Chapter 4, and implementation considerations related to each GDG recommendation can be found in Web Annex 5.

Integrated recommendations and their associated remarks are also presented throughout Chapter 3. References are provided in the remarks to indicate the source guideline. For all recommendations, the reader is referred to the specific WHO guidance for more details, including the evidence-base and implementation considerations.

3. Evidence and recommendations

This guideline includes 63 evidence-based recommendations on postnatal care – 31 newly developed Guideline Development Group (GDG) recommendations, and 32 recommendations relevant to postnatal care from previously published WHO guidelines that were integrated into this guideline.

The corresponding GRADE tables for recommendations are referred to in this chapter as "evidence base" (EB) tables and are numbered according to the specific recommendations to which they refer. These tables are presented separately in the Web Supplement to this document.[28] Evidence-to-decision tables with GDG judgements related to the evidence and considerations for all domains are presented with the summary of evidence and considerations for each recommendation. "Summary of judgements" tables are provided, indicating the final judgement corresponding to each domain. A template summary of judgements table showing the range of possible judgement options is shown in Annex 3.

This chapter provides the recommendations with the corresponding narrative summaries, grouped according to the broad category of intervention, namely:

A. MATERNAL CARE

B. NEWBORN CARE

C. HEALTH SYSTEMS AND HEALTH PROMOTION INTERVENTIONS

28 The Web Supplement is available at: https://www.who.int/publications/i/item/9789240045989

A. Maternal care

A.1 MATERNAL ASSESSMENT

Background

Early detection of conditions that may adversely affect women's health and well-being postpartum – including their capacity to care for themselves and their newborns – is an important component of quality postnatal care. This section of the guideline includes three sets of recommendations that have been integrated from WHO guidelines that are relevant to routine postnatal care, including one recommendation on the physiological assessment of the woman.

For additional guidance on routine assessment of the woman, health workers should refer to WHO's operational manual *(39)*, in which detailed guidance on assessment of the woman is provided.

A.1.1 Physiological assessment of the woman

RECOMMENDATION 1

All postpartum women should have regular assessment of vaginal bleeding, uterine tonus, fundal height, temperature and heart rate (pulse) routinely during the first 24 hours, starting from the first hour after birth. Blood pressure should be measured shortly after birth. If normal, the second blood pressure measurement should be taken within 6 hours. Urine void should be documented within 6 hours.

At each subsequent postnatal contact beyond 24 hours after birth, enquiries should continue to be made about general well-being and assessments made regarding the following: micturition and urinary incontinence, bowel function, healing of any perineal wound, headache, fatigue, back pain, perineal pain and perineal hygiene, breast pain and uterine tenderness and lochia. *(Recommended)*

Remarks

- This recommendation has been adapted and integrated from the 2014 *WHO recommendations on postnatal care of the mother and newborn (15)*, in which the recommendation was developed by Guideline Development Group (GDG) consensus based on existing WHO guidelines.
- No remarks were noted by the GDG responsible for the original recommendation.
- The postnatal care GDG noted that postpartum abdominal uterine tonus assessment for early identification of uterine atony is recommended for all women, as in the 2012 *WHO recommendations for the prevention and treatment of postpartum haemorrhage (40)*.

A.1.2 HIV catch-up testing

RECOMMENDATION 2a

In high HIV burden settings,[a] catch-up postpartum HIV testing is needed for women of HIV-negative or unknown status who missed early antenatal contact testing or retesting in late pregnancy at a third trimester visit. *(Context-specific recommendation)*

RECOMMENDATION 2b

In low HIV burden settings,[b] catch-up postpartum HIV testing can be considered for women of HIV-negative or unknown status who missed early antenatal contact testing or retesting in late pregnancy at a third trimester visit as part of the effort to eliminate mother-to-child transmission of HIV. Countries could consider this only for women who are in serodiscordant relationships, where the partner is not virally suppressed on ART, or who had other known ongoing HIV risks in late pregnancy at a third trimester visit. *(Context-specific recommendation)*

Remarks

- These recommendations have been adapted and integrated from the 2019 WHO *Consolidated guidelines on HIV testing services (41)*.
- The postnatal care Guideline Development Group noted the following statements from the 2019 guideline.
  - All pregnant women should be tested for HIV and hepatitis B surface antigen (HBsAg), particularly in settings with a ≥ 2% HBsAg seroprevalence in the general population, at least once and as early as possible as part of antenatal care. Maternal HIV retesting is advised in late pregnancy at a third trimester visit in high HIV burden settings. Maternal retesting is not advised in late pregnancy in low HIV burden settings. If implemented, it should address only members of key populations or women with a sexual partner with HIV who is not virally suppressed on ART or from a key population.
  - In specific districts or regions with a high HIV burden or incidence and for HIV-negative women (or women of unknown status) from key populations and those whose partners have HIV that is not virally suppressed, an additional message could encourage retesting at 14 weeks, six months or nine months postpartum.
  - All women should be provided with pretest information and give consent before testing, with the option for women to decline testing.
- Following the 2016 WHO *Consolidated guidelines on the use of antiretroviral drugs for treating and preventing HIV infection (42)*, ART initiation should be offered to all women with HIV following a confirmed HIV diagnosis, clinical assessment and an assessment of a person's readiness in order to avoid high rates of loss to follow-up after HIV diagnosis. For HIV-exposed infants, virological testing for HIV as early as possible is recommended so that infants with an initial positive virological test result can start ART without delay to save lives.

a High-prevalence settings are defined in the 2015 WHO publication Consolidated guidelines on HIV testing services as settings with greater than 5% HIV prevalence in the population being tested.
b Low-prevalence settings are settings with less than 5% HIV prevalence in the population being tested.

A.1.3 Screening for tuberculosis (TB) disease

RECOMMENDATION 3a

Systematic screening for tuberculosis (TB) disease may be conducted among the general population, including of women in the postpartum period, in areas with an estimated TB disease prevalence of 0.5% or higher. *(Context-specific recommendation)*

RECOMMENDATION 3b

In settings where the TB disease prevalence in the general population is 100/100 000 population or higher, systematic screening for TB disease may be conducted among women in the postpartum period. *(Context-specific recommendation)*

RECOMMENDATION 3c

Household contacts and other close contacts of individuals with TB disease, including women in the postpartum period and newborns, should be systematically screened for TB disease. *(Recommended)*

Remarks

- These recommendations have been adapted and integrated from the 2021 *WHO consolidated guidelines on tuberculosis Module 2: Screening – Systematic screening for tuberculosis disease (43)* where Recommendations 3a and 3b were considered conditional recommendations based on low and very low-certainty evidence, respectively, and Recommendation 3c was considered a strong recommendation based on moderate-certainty evidence.
- Related recommendations from this guideline include the following.
  - In high-prevalence settings, systematic screening for active tuberculosis should be considered for pregnant women as part of antenatal care as per the 2016 *WHO recommendations on antenatal care for a positive pregnancy experience (16)*.
  - Systematic screening for TB disease may be conducted among women in the postpartum period in subpopulations with structural risk factors for TB. These include urban poor communities, homeless communities, communities in remote or isolated areas, Indigenous populations, migrants, refugees, internally displaced persons and other priority groups with limited access to health care.
  - Any newborn whose mother has tested positive or who has had close contact with someone with TB disease should be screened for TB with a symptom screen and/or chest radiograph as part of active contact tracing.

A.2 INTERVENTIONS FOR COMMON PHYSIOLOGICAL SIGNS AND SYMPTOMS

Background

Women's bodies undergo substantial changes during the postnatal period, which are brought about by both hormonal and mechanical effects. These changes lead to a variety of common symptoms, including pain and discomfort, which can negatively affect a woman's postnatal experience.

The GDG considered the evidence and other relevant information to inform recommendations relating to non-pharmacological and/or pharmacological treatments for relieving perineal pain, uterine cramping/involution pain, urinary and faecal incontinence, and breast engorgement.

Perineal and uterine cramping/involution pain

Perineal pain is a common symptom in the short and long term after vaginal birth *(44)*. Pain may result from perineal trauma or present among women with an intact perineum. This pain can negatively impact women's social and emotional well-being as a result of decreased mobility, discomfort and difficulty with passing urine or faeces, and interfere with their ability to care for their newborns and to establish breastfeeding. A variety of non-pharmacological methods for the relief of perineal pain have been proposed as alternatives or additional treatments to pharmacological interventions. Cooling is one of the most commonly used non-pharmacological methods to relieve perineal pain *(45)*, including: (i) solid or crushed ice applied directly to the perineum or between layers of a pad; (ii) a gel pack applied to the perineum; or (iii) bathing. Pharmacological analgesics to relieve postpartum perineal pain include oral and rectal analgesics, and topically applied local analgesics (either as gel, ointments or sprays). Oral analgesics are the most common mode of administration of perineal pain relief. These include paracetamol, aspirin, and oral non-steroidal anti-inflammatory drugs (NSAIDs).

Uterine involution is where women experience cramping pain (often called after birth pains) and discomfort caused by involution of the uterus for two to three days after childbirth, as the uterus contracts and returns to its pre-pregnancy size *(46)*. Pharmacological analgesics to relieve uterine cramping pain usually include paracetamol, NSAIDs (e.g. aspirin and naproxen) and opioids (e.g. codeine) *(46)*.

Urinary and faecal incontinence

Urinary and faecal incontinence refers to the involuntary leakage of urine and faeces, respectively. Approximately one third of women experience urinary incontinence in the first three months after childbirth, which then gradually decreases during the first postpartum year *(47)*. Both urinary and faecal incontinence can have a significant impact on quality of life, which may persist for years. Pelvic floor muscle training (PFMT) includes one or more daily sets of repeated voluntary contractions of the pelvic floor muscles, several days per week, for a variable period during pregnancy or the postpartum period to strengthen the pelvic floor muscles *(48)*. PFMT is mainly proposed to prevent urine or stool leakage in women who are continent, or as a supervised treatment for women developing symptoms of incontinence during pregnancy or the puerperium. PFMT could also improve sexual function and other pelvic floor disorders in postnatal women, including faecal incontinence and pelvic floor prolapse.

Breast engorgement

Breast engorgement is the pathological overfilling of the breasts with milk, characterized by hard, painful, tight breasts and difficult breastfeeding *(49)*. It is usually due to compromised milk removal, either from separation of mother and baby, restrictive feeding practices and/or ineffective sucking or, less commonly, overproduction of milk. Breast engorgement affects between 15% and 50% of women *(49)* and may lead to mastitis. As lactation complications such as mastitis are the main reasons cited for early weaning, interventions that alleviate problems related to breastfeeding may help with breastfeeding continuation *(50)*.

Interventions for treatment of breast engorgement should aim to: (i) provide rapid relief of breast pain; (ii) enable successful attachment of the baby to the breast; (iii) facilitate efficient drainage of milk from the breasts; and (iv) prevent known complications such as mastitis and breast abscesses *(49)*. Non-pharmacological interventions include applying moist heat to the breast prior to feeding to aid oxytocin

uptake, frequent feeding, softening the areola prior to attachment, correct positioning and attachment of the baby to the breast during breastfeeding, hand-expressing or pumping milk to comfort if direct feeding at the breast is not possible, gentle massage during feeding, and applying cold compresses after feeding. These non-pharmacological interventions are sometimes coupled with analgesics (e.g. paracetamol) and anti-inflammatory medication (e.g. ibuprofen) if needed. Other pharmacological interventions include the use of oxytocin, protease or serrapeptase. Oxytocin (subcutaneous, oral or nasal sprays) may induce the milk-ejection reflex. Enzyme therapy is believed to be able to suppress inflammation, abate and alleviate pain and oedema, and accelerate the circulation of blood and lymph.

Box 3.1 Values

Findings from a qualitative evidence synthesis exploring what women want from postnatal care *(21)* indicate that women want a positive experience in which they are able to adapt to their new self-identity and develop a sense of confidence and competence as a mother. They also want to adjust to changes in their intimate and family relationships (including their relationship to their baby), navigate ordinary physical and emotional challenges, and experience the dynamic achievement of personal growth as they adjust to their new normal, both as parents and as individuals in their own cultural context. Women often feel unprepared for the physical and psychological effects of labour and birth-induced trauma and the impact this has on their ability to provide appropriate care for their baby (and other children). Women experience feelings of fear and anxiety associated with the long-term management of caesarean birth wounds, perineal damage, bladder problems, vaginal bleeding and general discomfort. Some women would like more information from health workers about how to soothe/treat physical injuries, and some would welcome the opportunity to discuss their labour and birth with a health worker (high confidence in the evidence). Highlighted in the review was the importance women place on breastfeeding as a medium for establishing a relationship with their baby (moderate confidence in the evidence) and the unanticipated challenges they sometimes experience when breastfeeding is difficult or painful (moderate confidence in the evidence). The review findings suggest that women would welcome any additional support, information and, where appropriate, treatment (pharmacological or non-pharmacological) to facilitate successful breastfeeding (high confidence in the evidence).

Box 3.2 Acceptability of interventions

A qualitative evidence synthesis of women's experiences of postnatal care found no direct evidence relating to women's views on the interventions evaluated to treat common physiological signs and symptoms in the postnatal period *(28)*. Indirect evidence from this review suggests that women appreciate any techniques or treatments to enhance comfort, mobility, sexual relations and psychosocial well-being (high confidence in the evidence). Findings from the same review also indicate that, in some contexts, women may prefer to use traditional practices to treat common physiological signs and symptoms (moderate confidence in the evidence). Women would like more information about potential childbirth complications (highlighted during the antenatal phase) and the steps they can take to avoid these problems (high confidence in the evidence)

Box 3.3 Feasibility of interventions

A qualitative evidence synthesis of women's experiences of postnatal care found no direct evidence relating to women's views on the feasibility of the interventions evaluated to treat common physiological signs and symptoms in the postnatal period *(28)*. Likewise, a qualitative evidence synthesis of health workers' experiences of postnatal care found no direct evidence relating to views on the feasibility of these interventions *(29)*. Indirect evidence from the latter review suggests that lack of personnel, resources and training may limit the offer of interventions to treat the common physiological signs and symptoms addressed, including provision of information and counselling related to these issues (moderate confidence in the evidence). Indirect evidence from the same review indicates that some women in low- and middle-income countries may be less likely to seek help for issues such as breast engorgement if they perceive that health facilities lack the resources to offer appropriate treatments or if they believe that treatment will incur additional costs (moderate confidence in the evidence). The lack of continuity of care and common policies or guidelines across different cadres and levels of maternal health services may limit the offer of consistent information and breastfeeding counselling (moderate confidence in the evidence).

A.2.1 Local cooling for perineal pain relief

RECOMMENDATION 4

Local cooling, such as with ice packs or cold pads, can be offered to women in the immediate postpartum period for the relief of acute pain from perineal trauma sustained during childbirth, based on a woman's preferences and available options. *(Recommended)*

Remarks

- The evidence reviewed included intermittent application of local cooling in the form of crushed ice between layers of a pad, or a gel pack, for 10 to 20 minutes in a single application to multiple applications in the first 48 hours after childbirth.
- In making this recommendation, the Guideline Development Group agreed that perineal pain relief should be individualized, considering the presence of perineal trauma, intensity of the pain, multiple sources of postpartum pain (e.g. perineal, uterine, breast pain) and the use of other forms of pain relief. Local cooling is low cost and unlikely to cause harmful effects if performed as instructed, and some women find it to be soothing.
- Non-pharmacological pain relief options can vary widely across settings and contexts, which might favour other non-pharmacological pain relief interventions and traditional and complementary medicine that were not evaluated during the guideline process, such as sitz baths, acupuncture or acupressure, aromatherapy, music, relaxation techniques, therapeutic ultrasound, transcutaneous electrical nerve stimulation (TENS) and laser therapy.
- All women should be asked about perineal pain and other perineal conditions (e.g. perineal trauma healing and haemorrhoids) during their postpartum stay in health facilities and at each postnatal care contact. Women should be advised on danger signs and symptoms, including any exacerbation of perineal pain as a manifestation of postpartum complications such as haematomas, haemorrhoids and infection.

Summary of evidence and considerations

Effects of the interventions (EB Table A.2.1)

Evidence was derived from an updated Cochrane systematic review on local cooling for relieving pain from perineal trauma sustained during childbirth *(45)*. As the review was focused on local cooling techniques, non-pharmacological interventions other than local cooling have not been included in this evidence summary.

The review captured women who had sustained non-severe perineal trauma due to episiotomy or first- or second-degree tears. Women who sustained third- or fourth-degree tears were not included, and nor were women with an intact perineum. The review included 10 trials (1258 women), of which 8 trials (1182 women) contributed data. The included trials were published between 2000 and 2017, and were all conducted in hospital settings in Brazil (3), the Islamic Republic of Iran (1), Thailand (1), Turkey (1) and the United Kingdom of Great Britain and Northern Ireland (2). One trial evaluating iced sitz baths versus no intervention (60 women) did not present outcome data in a format that could be included in the systematic review. In a small pilot trial (16 women), regular application of ice packs with compression (pressure) and the horizontal position of the mother was compared with ad hoc application of ice packs (alone), thus results of this trial were not considered in this framework.

Two comparisons are presented below: (1) perineal local cooling compared with no pain relief or usual care, and (2) perineal local cooling compared with other forms of non-pharmacological perineal pain relief. The evidence and judgements related to the effects of interventions (desirable effects, undesirable effects, and certainty of the evidence) are presented separately for each of the comparisons by type of control group. The remaining domains (values, resources, equity, acceptability and feasibility) were considered to be similar across the different comparisons and by type of control group (no intervention or usual care, other non-pharmacological perineal pain relief techniques).

Comparison 1: Perineal local cooling compared with no pain relief or usual care

Five trials (744 women) were included in the comparison of localized perineal cooling compared with no intervention or usual care. One trial compared ice packs with usual care. Two three-arm trials compared ice packs versus cold gel packs versus no intervention (and for this comparison women allocated to any of the cooling interventions were analysed together). Another trial compared cold gel packs with hygienic, absorbent maternity pads.

Maternal outcomes

Relief of symptoms: It is uncertain whether perineal local cooling reduces perineal pain within 4–6 hours of birth, within 24 hours of birth, or 24–48 hours after birth (using a scale of 0 = no pain to 10 = worst possible pain), when compared with no intervention (very low-certainty evidence). It is uncertain whether perineal local cooling reduces moderate or severe perineal pain within 24 hours of birth, when compared with no intervention (very low-certainty evidence). Low-certainty evidence suggests perineal local cooling may reduce moderate or severe perineal pain 24–48 hours after birth, when compared with no intervention (1 trial, 316 women; RR 0.73, 95% CI 0.57 to 0.94). However, these results should be considered with caution due to the high rate of attrition (29.8%). Low-certainty evidence suggests perineal local cooling may make little or no difference to perineal oedema within 24 hours of birth, when compared with no intervention (1 trial, 316 women; RR 1.00, 95% CI 0.87 to 1.16). It is uncertain whether perineal local cooling reduces perineal oedema 24–48 hours after birth, when compared with no intervention (very low-certainty evidence). Low-certainty evidence suggests perineal local cooling may make little or no difference to perineal bruising within 24 hours of birth (1 trial, 316 women; RR 0.98, 95% CI 0.81 to 1.19); it is uncertain whether perineal local cooling reduces perineal bruising 24–48 hours after birth, when compared with no intervention (very low-certainty evidence). It is uncertain whether perineal local cooling effects the composite score including perineal redness, oedema, bruising, discharge, and wound gaping within 24 hours of birth, or 24–48 hours after birth, when compared with no intervention (very low-certainty evidence).

Health service use: It is uncertain whether perineal local cooling affects the use of additional non-prescription or prescription analgesia for relief of perineal pain within 24 hours, or 24–48 hours after birth, when compared with no intervention (very low-certainty evidence).

Maternal functioning/well-being: Low-certainty evidence suggests perineal local cooling may make little or no difference to women's self-assessed moderate and severe perineal pain associated with sitting within 24 hours (1 trial, 312 women; RR 1.03, 95% CI 0.98 to 1.09) or 24–48 hours after birth (1 trial, 312 women; RR 1.00, 95% CI 0.95 to 1.05), compared with no intervention. Low-certainty evidence suggests perineal local cooling may make little or no difference to women's self-assessed moderate and severe pain associated with walking within 24 hours (1 trial, 312 women; RR 1.00, 95% CI 0.94 to 1.08) or 24–48 hours after birth (1 trial, 312 women; RR 1.01, 95% CI 0.93 to 1.09), when compared with no intervention. It is uncertain whether perineal local cooling affects women's self-assessed moderate and severe pain associated with feeding the baby within 24 or 24–48 hours after birth, when compared with no intervention (very low-certainty evidence).

Experience of postnatal care: Low-certainty evidence suggests perineal local cooling may make little or no difference to maternal satisfaction with overall perineal care at day 10 after birth, when compared with no intervention (1 trial, 308 women; RR 1.07, 95% CI 0.97 to 1.18).

Adverse effects directly related to the use of cooling techniques (e.g. cold burn) were not reported in the systematic review.

Newborn outcomes

Breastfeeding status: It is uncertain whether perineal local cooling affects the number of women providing any breastmilk to the baby 24–48 hours after birth (very low-certainty evidence).

Comparison 2: Perineal local cooling compared with other forms of non-pharmacological perineal pain relief

Comparison 2a: Perineal cooling and compression compared with uncooled gel pads and compression after vaginal birth in women with non-severe perineal trauma

One trial conducted in Thailand among 250 primiparous women with episiotomy or second degree

tears compared cold gel pads plus compression with uncooled gel pads plus compression.

Maternal outcomes

Relief of symptoms: It is uncertain whether cold gel pads plus compression reduces perineal pain within 4–6 hours of birth, when compared with uncooled gel pads plus compression (very low-certainty evidence). Low-certainty evidence suggests cold gel pads plus compression may reduce perineal pain within 24–48 hours after birth, when compared with uncooled gel pads plus compression (1 trial, 250 women; MD 0.43 lower, 95% CI 0.73 lower to 0.13 lower). Low-certainty evidence suggests cold gel pads plus compression may reduce perineal oedema 24–48 hours after birth, when compared with uncooled gel pads plus compression (1 trial, 250 women; MD 0.15 lower, 95% CI 0.28 lower to 0.03 lower). It is uncertain whether cold gel pads plus compression reduces perineal bruising within 24–48 hours after birth, when compared with uncooled gel pads plus compression (very low-certainty evidence).

Experience of postnatal care: Low-certainty evidence suggests cold gel pads plus compression may increase satisfaction with perineal care when compared with uncooled gel pads plus compression (1 trial, 250 women; MD 0.88 higher, 95% CI 0.38 higher to 1.38 higher).

Health service use and *maternal functioning or well-being* were not reported in the trial.

Adverse effects directly related to the use of cooling techniques (e.g. cold burn) were not reported in the systematic review.

Newborn outcomes

Breastfeeding status was not reported in the trial.

Comparison 2b: Perineal cooling (ice packs) compared with room-temperature water packs after vaginal birth in women with non-severe perineal trauma

One trial including 80 women with a normal vaginal birth compared ice packs (latex glove filled with crushed ice, wrapped in wet surgical dressing) with room-temperature packs (latex glove filled with water at 20–25°C, wrapped in wet surgical dressing). Review authors included only data from 63 women experiencing non-severe perineal trauma (28 and 35 women in the intervention and control groups, respectively).

Maternal outcomes

Relief of symptoms: It is uncertain whether ice packs reduce perineal pain within 4–6 hours or 24 hours after birth, or perineal oedema within 4–6 hours or 24 hours after birth, when compared with room-temperature water packs (very low-certainty evidence).

Health service use: It is uncertain whether ice packs affect the use of additional analgesia for relief of perineal pain within 24 hours after birth, when compared with room-temperature water packs (very low-certainty evidence).

Maternal functioning/well-being: It is uncertain whether ice packs affect maternal exhaustion within 4–6 hours or within 24 hours after birth, when compared with room-temperature water packs (very low-certainty evidence).

Experience of postnatal care: Low-certainty evidence suggests ice packs may make little or no difference to maternal satisfaction with treatment when compared with room-temperature water packs (1 trial, 63 women; RR 0.91, 95% CI 0.77 to 1.08). Low-certainty evidence suggests ice packs may make little or no difference to women's willingness to repeat treatment in future childbirth, when compared with room-temperature water packs (1 trial, 63 women; RR 0.88, 95% CI 0.75 to 1.04). Low-certainty evidence suggests ice packs may make little or no difference to a woman's willingness to recommend the intervention, when compared with room-temperature water packs (1 trial, 63 women; RR 0.89, 95% CI 0.77 to 1.03).

Adverse effects directly related to the use of cooling techniques (e.g. cold burn) were not reported in the systematic review.

Newborn outcomes

Breastfeeding status: Low-certainty evidence suggests ice packs may make little or no difference to women providing any breastmilk to the baby 48 hours after giving birth, when compared with room-temperature water packs (1 trial, 63 women; RR 1.00, 95% CI 0.94 to 1.06).

Comparison 2c: Perineal cooling (ice packs) compared with cold gel pads after vaginal birth in women with non-severe perineal trauma

Three trials compared ice packs versus cold gel pads. Two of them were three-arm trials including a no-intervention arm, but only women in the groups

receiving ice packs and cold gel pads were considered in this comparison. Primary authors reported data in different ways, so they were presented combined in a meta-analysis when possible, or separately.

Maternal outcomes

Relief of symptoms: It is uncertain whether ice packs reduce perineal pain, perineal oedema and perineal bruising within 4–6 hours after birth when compared with cold gel pads (very low-certainty evidence). It is uncertain whether ice packs reduce perineal pain, redness, oedema, bruising, discharge or wound gaping within 24 hours, or 24–48 hours after birth, when compared with cold gel pads (very low-certainty evidence).

Health service use: It is uncertain whether ice packs affect additional prescription or non-prescription analgesia for relief of perineal pain within 24 hours, or 24–48 hours after birth, when compared with cold gel pads (very low-certainty evidence).

Maternal functioning/well-being: It is uncertain whether ice packs reduce pain associated with sitting within 24 hours of birth, or 24–48 hours after vaginal birth, when compared with cold gel pads (very low-certainty evidence). It is uncertain whether ice packs reduce pain associated with walking within 24 hours of birth, or 24–48 hours after birth, when compared with cold gel pads (very low-certainty evidence). It is uncertain whether ice packs reduce pain associated with feeding the baby within 24 hours, or 24–48 hours after birth, when compared with cold gel pads (very low-certainty evidence).

Experience of postnatal care: It is uncertain whether ice packs effect women's opinion on treatment affects (good to excellent) at day five, when compared with cold gel pads (very low-certainty evidence). It is uncertain whether ice packs effect women being satisfied with overall perineal care (good to excellent) at day 10, when compared with cold gel pads (very low-certainty evidence).

Adverse effects directly related to the use of cooling techniques (e.g. cold burn) were not reported in the systematic review.

Newborn outcomes

Breastfeeding status: It is uncertain whether ice packs affect the number of women providing any breastmilk to the baby 48 hours after birth, when compared with cold gel pads (very low-certainty evidence).

Additional considerations

Whether the effects of the interventions differed by type of perineal trauma (episiotomy versus tear) is unknown, as no such subgroup analyses were included in the systematic review (included subgroup analyses were parity and mode of birth).

Values

See Box 3.1 in section 3.A.2: Interventions for common physiological signs and symptoms.

Resources

No economic evaluations of non-pharmacological interventions for relieving perineal pain were identified.

Additional considerations

Non-pharmacological interventions such as local cooling are relatively inexpensive where the necessary infrastructure and facilities already exist.

Equity

No direct evidence was identified on the impact on health equity of non-pharmacological interventions for relieving postpartum perineal pain. Non-pharmacological interventions based on local cooling for relieving postpartum perineal pain may decrease equity, as many of these interventions require access to clean water, refrigeration, ice and cold storage, which is limited in many low-income countries. Access to gel pads for local cooling may also be limited in these settings. However, in settings where women have access to clean water, refrigeration, ice and cold storage, non-pharmacological interventions based on local cooling may increase equity.

Acceptability

See Box 3.2 in section 3.A.2: Interventions for common physiological signs and symptoms.

Feasibility

See Box 3.3 in section 3.A.2: Interventions for common physiological signs and symptoms.

Additional considerations

Access to clean water, refrigeration, ice, cold storage and gel pads is limited in many low-income countries.

Table 3.1 Main resource requirements for local cooling for perineal pain relief

Resource	Description
**Staff**	• Midwives/nurses
**Training**	• Practice-based training for health workers
**Supplies**	• Varies depending on method:<br>– ice pack or crushed ice in a bag (gloves may also be used), perineal pad, sterile wet gauze, cotton or other skin barrier<br>– gel pad, sterile wet gauze, cotton or other skin barrier<br>– fresh, clean water and portable sitz bath or similar
**Equipment and infrastructure**	• Refrigeration, freezing and cold storage facilities (including electricity)
**Time**	• Varies depending on the intervention; cooling treatments are generally applied for up to 20 minutes per application, commencing shortly after birth and at specified intervals or as needed for up to several days postpartum
**Supervision and monitoring**	• Same as for usual care

Table 3.2 Summary of judgements: Local cooling compared with no pain relief or usual care

Domain	Judgement
Desirable effects	Trivial
Undesirable effects	Don't know
Certainty of the evidence	Very low
Values	Probably no important uncertainty or variability
Balance of effects	Don't know
Resources required	Negligible costs or savings
Certainty of the evidence on required resources	No included studies
Cost-effectiveness	Don't know
Equity	Varies
Acceptability	Probably yes
Feasibility	Varies

Table 3.3 Summary of judgements: Local cooling compared with other forms of non-pharmacological perineal pain relief

Domain	Judgement
Desirable effects	Small
Undesirable effects	Don't know
Certainty of the evidence	Very low
Values	Probably no important uncertainty or variability
Balance of effects	Don't know
Resources required	Negligible costs or savings
Certainty of the evidence on required resources	No included studies
Cost-effectiveness	Don't know
Equity	Varies
Acceptability	Probably yes
Feasibility	Varies

A.2.2 Oral analgesia for perineal pain relief

RECOMMENDATION 5

Oral paracetamol is recommended as first-line choice when oral analgesia is required for the relief of postpartum perineal pain. *(Recommended)*

Remarks

- In making this recommendation, the Guideline Development Group (GDG) agreed perineal pain relief should be individualized, considering the presence of perineal trauma, intensity of the pain, multiple sources of postpartum pain (e.g. perineal, uterine, breast pain), the use of the lowest effective dose for the shortest period of time, and adverse effects and contraindications, including breastfeeding. The use of single-dose paracetamol given to the woman in the immediate postnatal period is unlikely to pose any significant risk to the newborn as the amount likely to be excreted in breastmilk would be very little and the volume of breastmilk consumed by the infant in the first days after birth is likely to be small.
- Aspirin is contraindicated during breastfeeding based on evidence of potentially harmful effects on breastfeeding babies due to salicylate and salicylate metabolites excreted in breastmilk.
- All women should be advised about the use of local cooling as a non-pharmacological option to relieve acute pain from perineal trauma sustained during childbirth, based on availability and a woman's preferences (see Recommendation 4 in this guideline).
- In acknowledgement of the limited evidence on the comparative effectiveness of different oral analgesics, the GDG suggested that when local perineal cooling or paracetamol is not effective in relieving perineal pain, women should be advised of other pharmacological pain relief options based on safety profile (e.g. allergies, adverse effects, contraindications), availability, experience with a particular analgesic and cost.
- All women should be asked about perineal pain and other perineal conditions (e.g. perineal trauma healing and haemorrhoids) during their postpartum stay in health facilities and at each postnatal care contact. Women should be advised on danger signs and symptoms, including any exacerbation of perineal pain as a manifestation of postpartum complications such as haematomas, haemorrhoids and infection.

Summary of evidence and considerations

Effects of the interventions (EB Table A.2.2)

Evidence was derived from three Cochrane reviews addressing the effect of a single dose of paracetamol/acetaminophen *(51)*, acetylsalicylic acid (hereafter aspirin) *(52)* and of NSAIDs *(53)* compared with placebo or no intervention, or with another pharmacological agent, to reduce acute perineal pain in the early postnatal period. The effect of different doses of the same drugs were also assessed.

Paracetamol

Evidence was derived from 10 trials with 1367 women *(51)*. Five trials were published in the 1970s, four in the 1980s and the most recent in 1992. Trials were conducted in Canada (1), France (1), the United States of America (USA) (7) and the Bolivarian Republic of Venezuela (1). No trials evaluated perineal pain relief after vaginal birth with intact perineum, and no distinctions were made between episiotomy and spontaneous lacerations. All trials were multi-arm including comparisons of paracetamol with other analgesics alone, or in combination, or with placebo. Authors of the review extracted only data from the paracetamol versus placebo arms. Two different doses of paracetamol were included in the trials: 500–650 mg and 1000 mg, versus placebo.

Aspirin

Evidence was derived from 17 trials with 1132 women *(52)*.[29] Trials were conducted in Belgium (1), Canada (1), India (1), the USA (11) and the Bolivarian Republic of Venezuela (3) between 1967 and 1997. No trials evaluated perineal pain relief after vaginal birth with intact perineum, and trials only included women with episiotomy. Most trials clearly specified that breastfeeding was an exclusion criterion, and all excluded women with known sensitivity or allergy to aspirin, and women who had previously received analgesia. Fifteen trials had multiple arms (between three and five) and, in addition to aspirin, assessed

29 One trial did not report the number of women recruited.

a number of other agents for perineal pain. The review analysed only the aspirin and placebo arms of the included trials. Doses of aspirin varies between trials, from 500 mg to 1200 mg. Three trials included two or more aspirin arms (in addition to a placebo arm); two trials compared 600 mg and 1200 mg aspirin (one of these included 49 women and in the other trial the number of included women was not reported). The other trial compared 300 mg, 600 mg and 1200 mg aspirin.

Oral non-steroidal anti-inflammatory drugs (NSAIDs)

Evidence was derived from 28 trials with 4181 women *(53)*. Trials were conducted in a mix of LMICs and high-income countries (HICs) between 1967 and 2013 (most conducted in the 1980s). Twenty-seven trials (3853 women) examined the effect of NSAIDs for relief of post-episiotomy pain, and one trial (328 women) included women with any perineal trauma requiring repair but excluded women with third or fourth degree tears. All trials excluded women who were breastfeeding. Thirteen different NSAIDs were evaluated. Data from trials reporting on indoprofen, zomepirac and fluproquazone were removed from the systematic review analyses as these NSAIDs are presently withdrawn from the market due to adverse effects. As the data on aspirin versus placebo or no treatment were more comprehensive in the aspirin systematic review, and to avoid double-reporting of the same data, the data were deliberately extracted only from the aspirin review to inform the current evidence summary. Trials compared any NSAID with placebo, paracetamol, or aspirin (as an alternative NSAID).

Three comparisons are presented below: (1) single-dose oral analgesic (any dose) compared with placebo, (2) single-dose oral analgesic compared with a higher single dose of the same analgesic, and (3) single-dose oral analgesic compared with a single dose of an alternative oral analgesic. The evidence and judgements related to the effects of interventions (desirable effects, undesirable effects, and certainty of the evidence) are presented separately for each comparison by type of control group. The remaining domains (values, resources, equity, acceptability and feasibility) were considered to be similar across the different comparisons and by type of control group (placebo or other forms of pharmacological perineal pain relief).

Comparison 1: Single-dose oral analgesic (any dose) compared with placebo

Comparison 1a: Single-dose paracetamol compared with placebo

Maternal outcomes

Relief of symptoms: It is uncertain whether a single dose of paracetamol provides adequate pain relief as reported by women when compared with placebo (very low-certainty evidence). Subgroup analyses according to the dose of paracetamol showed the following.

- *Paracetamol 500–650 mg:* It is uncertain whether paracetamol 500–650 mg provides adequate pain relief when compared with placebo (very low-certainty evidence).
- *Paracetamol 1000 mg:* Low-certainty evidence suggests paracetamol 1000 mg may provide adequate pain relief when compared with placebo (6 trials, 797 women; RR 2.42, 95% CI 1.53 to 3.81).

Health service use: Low-certainty evidence suggests a single dose of paracetamol may reduce the need for additional pain relief when compared with placebo (8 trials, 1132 women; RR 0.34, 95% CI 0.21 to 0.55). Subgroup analyses according to the dose of paracetamol showed the following.

- *Paracetamol 500–650 mg:* Low-certainty evidence suggests a single dose of 500–650 mg of paracetamol may reduce the need for additional pain relief when compared with placebo (3 trials, 317 women; RR 0.30, 95% CI 0.17 to 0.53).
- *Paracetamol 1000 mg:* Low-certainty evidence suggests a single dose of 1000 mg of paracetamol may reduce the need for additional pain relief when compared with placebo (6 trials, 815 women; RR 0.36, 95% CI 0.19 to 0.67).

Adverse effects: Low-certainty evidence suggests a single dose of paracetamol 1000 mg may have little or no effect on the incidence of maternal nausea or maternal sleepiness when compared with placebo (1 trial, 232 women; RR 0.18, 95% CI 0.01 to 3.66 and 1 trial, 232 women; RR 0.89, 95% CI 0.18 to 4.30, respectively). It is uncertain whether a single dose of paracetamol 1000 mg affects maternal bowel movements or maternal gastric discomfort when compared with placebo (very low-certainty evidence).

Maternal functioning/well-being and *experience of postnatal care* were not reported in the included trials.

Newborn outcomes

Breastfeeding status[30] and *adverse effects* were not reported in the included trials.

Comparison 1b: Single-dose aspirin compared with placebo

Maternal outcomes

Relief of symptoms: Low-certainty evidence suggests a single dose of aspirin may provide adequate pain relief as reported by women when compared with placebo (13 trials, 1001 women; RR 2.03, 95% CI 1.69 to 2.42). Subgroup analyses were done according to the dose of aspirin used, as follows.

- *Aspirin 500–650 mg:* Low-certainty evidence suggests a single dose of aspirin 500–650 mg may adequately relieve perineal pain when compared with placebo (11 trials, 800 women; RR 1.98, 95% CI 1.64 to 2.39).
- *Aspirin 300, 900, and 1200 mg:* It is uncertain whether a single dose of 300 mg, 900 mg or 1200 mg of aspirin adequately relieves perineal pain when compared with placebo (very low-certainty evidence).

Health service use: Low-certainty evidence suggests a single dose of aspirin may reduce the need for additional pain relief in the 4–8 hours after drug administration when compared with placebo (10 trials, 744 women; RR 0.25, 95% CI 0.17 to 0.37). Certainty of the evidence was very low for subgroups analyses according to dose (300 mg, 500–650 mg, 900 mg and 1200 mg).

Adverse effects: It is uncertain whether a single dose of aspirin affects the risk of adverse effects overall when compared with placebo (very low-certainty evidence). Certainty of the evidence was very low for subgroup analyses according to dose (300 mg, 500–650 mg, 900 mg and 1200 mg).

Maternal functioning/well-being and *experience of postnatal care* were not reported in the trials included in any of the three systematic reviews.

Newborn outcomes

Breastfeeding status and *adverse effects* were not reported in the included trials.

30 Breastfeeding was an exclusion criterion in many of the trials in the aspirin review and in all trials in the NSAIDs review.

Comparison 1c: Single-dose NSAID compared with placebo

Maternal outcomes

Relief of symptoms: It is uncertain whether a single dose of NSAID provides adequate pain relief at 4 hours after administration when compared with placebo (very low-certainty evidence). Subgroup analyses according to the type and dose of NSAID showed the following.

- *Diclofenac 100 mg:* Low-certainty evidence suggests a single dose of diclofenac 100 mg may provide adequate pain relief at 4 hours after administration when compared with placebo (1 trial, 64 women; RR 2.36, 95% CI 1.03 to 5.42).
- *Meclofenamate sodium 100 mg:* Low-certainty evidence suggests a single dose of meclofenamate sodium 100 mg may provide adequate pain relief at 4 hours after administration when compared with placebo (3 trials, 260 women; RR 1.42, 95% CI 1.10 to 1.82).
- *Meclofenamate sodium 200 mg:* Low-certainty evidence suggests a single dose of meclofenamate sodium 200 mg may provide adequate pain relief at 4 hours after administration when compared with placebo (3 trials, 262 women; RR 1.42, 95% CI 1.10 to 1.83).
- It is uncertain whether a single dose of ibuprofen 300–400 mg or 800 mg, diclofenac 25 mg or 100 mg, ketoprofen 25 mg, diflunisal 125 mg, 500 mg, 250 mg or 500 mg, ketoprofen 50 mg, or flurbiprofen 25 mg, 50 mg or 100 mg provides adequate pain relief at 4 hours after administration when compared with placebo (very low-certainty evidence).

Low-certainty evidence suggests a single dose of NSAID (any dose) may provide adequate pain relief at 6 hours after administration when compared with placebo (17 trials, 2079 women; RR 1.92, 95% CI 1.69 to 2.17). Subgroup analyses according to the type and dose of NSAID showed the following.

- *Ibuprofen 300–400 mg:* Low-certainty evidence suggests a single dose of ibuprofen 300–400 mg may provide adequate pain relief at 6 hours after administration when compared with placebo (2 trials, 124 women; RR 2.08, 95% CI 1.30 to 3.32).
- *Meclofenamate sodium 100 mg:* Low-certainty evidence suggests a single dose of meclofenamate sodium 100 mg may provide adequate pain relief at 6 hours after administration when compared

with placebo (3 trials, 260 women; RR 1.36, 95% CI 1.05 to 1.76).

- *Meclofenamate sodium 200 mg:* Low-certainty evidence suggests a single dose of meclofenamate sodium 200 mg may provide adequate pain relief at 6 hours after administration when compared with placebo (3 trials, 262 women; RR 1.40, 95% CI 1.07 to 1.83).
- *Dipyrone 500 mg:* Low-certainty evidence suggests a single dose of dipyrone 500 mg may provide adequate pain relief at 6 hours after administration when compared with placebo (1 trial, 133 women; RR 2.21, 95% CI 1.44 to 3.39).
- It is uncertain whether a single dose of ibuprofen 900 mg, ketoprofen 25 mg or 50 mg, diflunisal 125 mg, 250 mg or 500 mg, aceclofenac 50 mg, 100 mg or 150 mg, etodolac 25 mg or 100 mg, antrafenine 300 mg, flurbiprofen 25 mg, 50 mg or 100 mg, or fenoprofen 12.5 mg, 25 mg, 50 mg, 100 mg, 200 mg or 300 mg provides adequate pain relief at 6 hours after administration when compared with placebo (very low-certainty evidence).

Health service use: Low-certainty evidence suggests a single dose of NSAID (any dose) may reduce the need for additional pain relief at 4 hours after administration when compared with placebo (4 trials, 486 women; RR 0.39, 95% CI 0.26 to 0.58). Subgroup analyses according to the type and dose of NSAID showed the following.

- *Ibuprofen 300–400 mg:* Low-certainty evidence suggests a single dose of ibuprofen 300–400 mg reduces the need for additional pain relief at 4 hours after administration when compared with placebo (3 trials, 240 women; RR 0.32, 95% CI 0.18 to 0.56).
- *Ibuprofen 800 mg:* It is uncertain whether a single dose of ibuprofen 800 mg reduces the need for additional pain relief at 4 hours after administration when compared with placebo (very low-certainty evidence).

It is uncertain whether a single dose of NSAID (any dose) reduces the need for additional analgesia at 6 hours after administration when compared with placebo (very low-certainty evidence). Subgroup analyses according to the type and dose of NSAID showed the following.

- *Ibuprofen 300–400 mg:* Low-certainty evidence suggests a single dose of ibuprofen 300–400 mg may reduce the need for additional analgesia at 6 hours after administration when compared with placebo (3 trials, 186 women; RR 0.33, 95% CI 0.20 to 0.54).
- *Meclofenamate sodium 100 mg:* Low-certainty evidence suggests a single dose of meclofenamate sodium 100 mg may reduce the need for additional analgesia at 6 hours after administration when compared with placebo (3 trials, 299 women; RR 0.34, 95% CI 0.21 to 0.53).
- *Meclofenamate sodium 200 mg:* Low-certainty evidence suggests a single dose of meclofenamate sodium 200 mg may reduce the need for additional analgesia at 6 hours after administration when compared with placebo (2 trials, 142 women; RR 0.45, 95% CI 0.29 to 0.70).
- *Flurbiprofen 25 mg:* Low-certainty evidence suggests a single dose of flurbiprofen 25 mg may reduce the need for additional analgesia at 6 hours after administration when compared with placebo (1 trial, 40 women; RR 0.06, 95% CI 0.01 to 0.49).
- *Flurbiprofen 50 mg:* Low-certainty evidence suggests a single dose of flurbiprofen 50 mg may reduce the need for additional analgesia at 6 hours after administration when compared with placebo (1 trial, 37 women; RR 0.03, 95% CI 0.00 to 0.56).
- *Flurbiprofen 100 mg:* Low-certainty evidence suggests a single dose of flurbiprofen 100 mg may reduce the need for additional analgesia at 6 hours after administration when compared with placebo (1 trial, 39 women; RR 0.03, 95% CI 0.00 to 0.53).
- It is uncertain whether a single dose of ibuprofen 900 mg or antrafenine 300 mg reduces the need for additional analgesia at 6 hours after administration when compared with placebo (very low-certainty evidence).

Adverse effects: It is uncertain whether a single dose of NSAID (any dose) affects the risk of adverse effects at 4 hours after administration, when compared with placebo (very low-certainty evidence). Low-certainty evidence suggests a single dose of NSAID may make little or no difference to adverse effects at 6 hours after administration, when compared with placebo (13 trials, 1388 women; RR 1.38, 95% CI 0.71 to 2.70). Subgroup analyses according to the type and dose of NSAID showed the following.

- *Dipyrone 500 mg:* Low-certainty evidence suggests a single dose of dipyrone 500 mg may make little or no difference to adverse effects at 6 hours after administration when compared with placebo (2 trials, 335 women; RR 2.48, 95% CI 0.49 to 12.46).
- It is uncertain whether a single dose of ibuprofen 300–400 mg or 900 mg, ketoprofen 25 mg or 50 mg, aceclofenac 50 mg, 100 mg or 150 mg,

diflunisal 125 mg, 250 mg or 500 mg, antrafenine 300 mg, or flurbiprofen 25 mg, 50 mg or 100 mg affects the risk of adverse effects at 6 hours after administration, when compared with placebo (very low-certainty evidence).

Maternal functioning/well-being and *experience of postnatal care* were not reported in the trials included in any of the three systematic reviews.

Newborn outcomes
Breastfeeding status and *adverse effects* were not reported in the included trials.

Comparison 2: Single-dose oral analgesic compared with a higher single dose of the same analgesic

Comparison 2a: Single-dose aspirin compared with a higher single dose of aspirin

Maternal outcomes
Relief of symptoms: It is uncertain whether any dose of aspirin (300 mg or 600 mg) improves pain relief as reported by women when compared with a higher dose of aspirin (600 mg or 1200 mg) (very low-certainty evidence).

Health service use: It is uncertain whether any dose of aspirin (300 mg or 600 mg) reduces the need for additional perineal pain relief when compared with a higher dose of aspirin (600 mg or 1200 mg) (very low-certainty evidence).

Adverse effects: It is uncertain whether any dose of aspirin affects the risk of adverse effects when compared with a higher dose of aspirin (very low-certainty evidence).

Maternal functioning/well-being and *experience of postnatal care* were not reported in the included trials.

Newborn outcomes
Breastfeeding status and *adverse effects* were not reported in the included trials.

Comparison 2b: Single-dose NSAID compared with a higher single dose of the same NSAID

Maternal outcomes
Relief of symptoms: It is uncertain whether any dose of the following NSAIDs improves pain relief at four and/or at 6 hours after administration when compared with a higher dose of the same NSAID (very low-certainty evidence):
- ibuprofen 300–400 mg versus 800 mg at four and at 6 hours after administration;
- diclofenac 25 mg versus 50 mg or 100 mg at 4 and at 6 hours after administration;
- diflunisal (125 mg or 250 mg) versus diflunisal (250 mg or 500 mg) at 4 hours after administration;
- ketoprofen 25 mg versus 50 mg at 4 and at 6 hours after administration;
- aceclofenac (50 mg or 100 mg) versus aceclofenac (150 mg) at 4 and at 6 hours after administration;
- etodolac 25 mg versus 100 mg at 6 hours after administration;
- flurbiprofen (25 mg or 50 mg) versus flurbiprofen (50 mg or 100 mg) at 4 and at 6 hours after administration; and
- fenoprofen (any dose) versus fenoprofen (any higher dose) at 6 hours after administration.

Moderate-certainty evidence suggests meclofenamate sodium 100 mg probably makes little or no difference to adequate pain relief at 4 hours and at 6 hours after administration when compared with meclofenamate sodium 200 mg (3 trials, 348 women; RR 1.00, 95% CI 0.85 to 1.17; and RR 1.00, 95% CI 0.84 to 1.18, respectively).

Health service use: It is uncertain whether any dose of the following NSAIDs reduces the need for additional pain relief at 4 and/or at 6 hours after administration when compared with a higher dose of the same NSAID (very low-certainty evidence):
- ibuprofen 300–400 mg versus 800 mg at 4 and at 6 hours after administration;
- meclofenamate sodium 100 mg versus 200 mg at 6 hours after administration;
- flurbiprofen (any dose) versus flurbiprofen (any higher dose) at 6 hours after administration.

Adverse effects: It is uncertain whether any dose of the following NSAIDs affects the risk of adverse effects at 4 and/or at 6 hours after administration

when compared with a higher dose of the same NSAID (very low-certainty evidence):

- ibuprofen 300–400 mg versus 800 mg at 4 and at 6 hours after administration;
- diflunisal (125 mg or 250 mg) versus diflunisal (250 mg or 500 mg) at 4 and at 6 hours after administration;
- ketoprofen 25 mg versus 50 mg at 6 hours after administration;
- aceclofenac (50 mg or 100 mg) versus aceclofenac (100 mg or 150 mg) at 6 hours after administration; and
- flurbiprofen (any dose) versus flurbiprofen (any higher dose) at 6 hours after administration.

Maternal functioning/well-being and *experience of postnatal care* were not reported in the included trials.

Newborn outcomes

Breastfeeding status and *adverse effects* were not reported in the included trials.

Comparison 3: Single-dose oral analgesic compared with a single dose of an alternative oral analgesic

Comparison 3a: Single-dose NSAID compared with single-dose paracetamol

Maternal outcomes

Relief of symptoms: Moderate-certainty evidence suggests NSAIDs (single-dose, any dose) probably provide adequate pain relief at 4 hours after administration when compared with paracetamol (3 trials, 342 women; RR 1.54, 95% CI 1.07 to 2.22). Subgroup analyses according to the type and dose of analgesic showed the following.

- *Ibuprofen 300–400 mg versus paracetamol 1000 mg:* Low-certainty evidence suggests ibuprofen 300–400 mg may make little or no difference to adequate pain relief at 4 hours after administration when compared with paracetamol 1000 mg (1 trial, 72 women; RR 1.68, 95% CI 0.93 to 3.04).
- *Ibuprofen 300–400 mg versus paracetamol 500 mg:* Low-certainty evidence suggests ibuprofen 300–400 mg may make little or no difference to adequate pain relief at 4 hours after administration when compared with paracetamol 500 mg (1 trial, 210 women; RR 1.40, 95% CI 0.86 to 2.28).
- *Aceclofenac 100 mg versus paracetamol 650 mg:* It is uncertain whether aceclofenac 100 mg provides adequate pain relief at 4 hours after administration when compared with paracetamol 650 mg (very low-certainty evidence).

It is uncertain whether aceclofenac 100 mg provides adequate pain relief at 6 hours after administration when compared with paracetamol 650 mg (very low-certainty evidence).

Health service use: Low-certainty evidence suggests a single dose of ibuprofen 300–400 mg may make little or no difference to the need for additional analgesia at 4 hours after administration when compared with paracetamol 1000 mg (1 trial, 72 women; RR 0.55, 95% CI 0.27 to 1.13). Low-certainty evidence suggests a single dose of ibuprofen 300–400 mg may reduce the need for additional analgesia at 6 hours after administration when compared with paracetamol 1000 mg (1 trial, 59 women; RR 0.28, 95% CI 0.12 to 0.67).

Adverse effects: It is uncertain whether a single dose of ibuprofen 300–400 mg affects the risk of adverse effects at 4 hours after administration when compared with paracetamol 500 mg (very low-certainty evidence).

Low-certainty evidence suggests NSAIDs (single-dose, any dose) may make little or no difference to adverse effects at 6 hours after administration when compared with paracetamol (3 trials, 300 women; RR 0.74, 95% CI 0.27 to 2.08). Subgroup analyses according to the type and dose of analgesic showed the following.

- *Dipyrone 500 mg versus paracetamol 500 mg:* Low-certainty evidence suggests a single dose of dipyrone 500 mg may make little or no difference to the risk of adverse effects at 6 hours after administration when compared with paracetamol 500 mg (1 trial, 201 women; RR 0.71, 95% CI 0.23 to 2.15).
- *Aceclofenac 100 mg versus paracetamol 650 mg:* It is uncertain whether a single dose of aceclofenac 100 mg affects the risk of adverse effects at 6 hours after administration when compared with paracetamol 650 mg.

Newborn outcomes

Breastfeeding status and *adverse effects* were not reported in the included trials.

Comparison 3b: Single-dose NSAID (aspirin) compared with a single dose of another NSAID[31]

Maternal outcomes

Relief of symptoms: Moderate-certainty evidence suggests aspirin probably makes little or no difference to adequate pain relief at 4 hours after administration when compared with a different NSAID (4 trials, 731 women; RR 0.95, 95% CI 0.83 to 1.09). Subgroup analyses according to the type and dose of analgesic used showed the following.

- *Aspirin 900 mg versus ibuprofen:* It is uncertain whether aspirin 900 mg provides adequate pain relief at 6 hours after administration when compared with ibuprofen 300–400 mg or 900 mg (very low-certainty evidence).
- *Aspirin 500–650 mg versus dipyrone 500 mg:* Low-certainty evidence suggests aspirin 500-650 mg may make little or no difference to adequate pain relief at 6 hours after administration when compared with dipyrone 500 mg (1 trial, 179 women; RR 0.90, 95% CI 0.75 to 1.08).
- It is uncertain whether aspirin 500-650 mg provides adequate pain relief at 4 and at 6 hours after administration when compared with diflunisal 125 mg, 250 mg, or 500 mg, ibuprofen 300–400 mg, diclofenac 25 mg, 50 mg, or 100 mg, or flurbiprofen 25 mg, 50 mg or 100 mg (very low-certainty evidence).

Health service use: It is uncertain whether aspirin 500–650 mg makes any difference to the need for additional analgesia at 4 hours after administration when compared with ibuprofen 300–400 mg (very low-certainty evidence). It is uncertain whether aspirin 500–650 mg or 900 mg makes any difference to the need for additional analgesia at 6 hours after administration when compared with a different NSAID (ibuprofen 300–400 mg or 900 mg; flurbiprofen 25 mg, 50 mg or 100 mg) (very low-certainty evidence).

Adverse effects: It is uncertain whether aspirin 500–650 mg or 900 mg[32] increases the risk of adverse effects at 4 and at 6 hours after administration when compared with a different NSAID (dipyrone 500 mg; flurbiprofen 25 mg, 50 mg or 100 mg; diflunisal 125 mg, 250 mg or 500 mg; ibuprofen 300–400 mg or 900 mg) (very low-certainty evidence).

Maternal functioning/well-being and experience of postnatal care were not reported in the included trials.

Newborn outcomes

Breastfeeding status and adverse effects were not reported in the included trials.

Additional considerations

Other forms of pharmacological perineal pain relief were not considered, including rectal analgesics *(54, 55)* and topically applied anaesthetics for treating perineal pain after childbirth *(56)*.

Values

See Box 3.1 in section 3.A.2: Interventions for common physiological signs and symptoms.

Resources

No economic evaluations of pharmacological treatments for relieving perineal pain were identified.

Equity

No direct evidence was identified on the impact on health equity of pharmacological interventions for relieving postpartum perineal pain. Pharmacological interventions for relieving postpartum perineal pain may increase equity, as many of these interventions are widely available without a prescription and at low cost. However, pharmacological interventions may decrease equity if women are expected to pay for analgesics themselves.

Acceptability

See Box 3.2 in section 3.A.2: Interventions for common physiological signs and symptoms.

Additional considerations

Some women may decline paracetamol due to fears of harmful effects on the baby through breastmilk. Women who use paracetamol need clear information about the dose of paracetamol in any concurrent medications, to help avoid inadvertent overdose or toxicity *(51)*. Generally, it is anticipated that women will accept single-dose, oral analgesics with clear information about safety.

31 The direction of this comparison (comparing aspirin with other NSAIDs) differs from the others presented in this evidence summary due to the reporting in the Cochrane review, which has been reproduced faithfully.

32 Aspirin 900 mg assessed at 6 hours after administration only.

Table 3.4 Main resource requirements of oral analgesia for perineal pain relief

Resource	Description
**Staff**	• Doctors/midwives/nurses
**Training**	• Practice-based training for health workers
**Supplies**	• Analgesic drugs – all oral preparation, price per tablet/capsule *(57)*:<br>– paracetamol 500 mg = US$ 0.004<br>– NSAIDs:<br>· acetylsalicylic acid (aspirin) 500 mg = US$ 0.005<br>· ibuprofen 400 mg = US$ 0.01<br>· diclofenac sodium 50 mg = US$ 0.005
**Equipment and infrastructure**	• On-site pharmacy and/or medicine stock management system that is managed by a trained pharmacist or dispenser
**Time**	• Dispensing time estimated to be 2–5 minutes
**Supervision and monitoring**	• Same as for usual care

Feasibility

See Box 3.3 in section 3.A.2: Interventions for common physiological signs and symptoms.

Additional considerations

Generally, oral analgesics are widely-available at low cost. Aspirin is contraindicated during breastfeeding, but may be considered for use in non-breastfeeding women. The only non-opioid and non-steroidal anti-inflammatory medicines listed in the WHO Model List of Essential Medicines are acetylsalicylic acid (aspirin) (tablet: 100 mg to 500 mg; suppository: 50 mg to 150 mg), ibuprofen (tablet: 200 mg, 400 mg, 600 mg), and paracetamol (tablet: 100 mg to 500 mg; suppository: 100 mg) *(58)*.

Table 3.5 Summary of judgements: Single-dose oral analgesic (any dose) compared with placebo

Domain	Judgement
Desirable effects	Trivial
Undesirable effects	Varies
Certainty of the evidence	Very low
Values	Probably no important uncertainty or variability
Balance of effects	Don't know
Resources required	Negligible costs or savings
Certainty of the evidence on required resources	No included studies
Cost-effectiveness	Don't know
Equity	Varies
Acceptability	Probably yes
Feasibility	Probably yes

Table 3.6 Summary of judgements: Single-dose oral analgesic compared with a higher single dose of the same analgesic

Domain	Judgement
Desirable effects	Trivial
Undesirable effects	Varies
Certainty of the evidence	Very low
Values	Probably no important uncertainty or variability
Balance of effects	Don't know
Resources required	Negligible costs or savings
Certainty of the evidence on required resources	No included studies
Cost-effectiveness	Don't know
Equity	Varies
Acceptability	Probably yes
Feasibility	Probably yes

Table 3.7 Summary of judgements: Single-dose oral analgesic compared with a single dose of an alternative oral analgesic

Domain	Judgement
Desirable effects	Trivial
Undesirable effects	Varies
Certainty of the evidence	Very low
Values	Probably no important uncertainty or variability
Balance of effects	Don't know
Resources required	Negligible costs or savings
Certainty of the evidence on required resources	No included studies
Cost-effectiveness	Don't know
Equity	Varies
Acceptability	Probably yes
Feasibility	Probably yes

A.2.3 Pharmacological relief of pain due to uterine cramping/involution

RECOMMENDATION 6

Oral non-steroidal anti-inflammatory drugs (NSAIDs) can be used when analgesia is required for the relief of postpartum pain due to uterine cramping after childbirth, based on a woman's preferences, the clinician's experience with analgesics and availability. *(Recommended)*

Remarks

- In making this recommendation, the Guideline Development Group (GDG) agreed uterine pain relief should be individualized, considering the intensity of the pain, multiple sources of postpartum pain (e.g. perineal, uterine, breast pain), the use of the lowest effective dose for the shortest period of time, and adverse effects and contraindications, including breastfeeding.
- Aspirin is contraindicated during breastfeeding based on evidence of potentially harmful effects on breastfeeding babies due to salicylate and salicylate metabolites excreted in breastmilk.
- In acknowledgement of the limited evidence on the comparative effectiveness of different pharmacological and non-pharmacological interventions for postpartum uterine pain relief, the GDG suggested that women should be advised of different options based on safety profile (e.g. allergy, adverse effects, contraindications), availability, experience with a particular analgesic and cost.
- The GDG noted that use of opioids for the relief of pain due to uterine cramping should be discouraged as opioids showed no advantage over NSAIDs, are associated with maternal adverse effects, are contraindicated during breastfeeding and are associated with a risk of developing psychological and physical dependence.
- All women should be informed about uterine involution and changes in lochia postpartum. They should be asked about abdominal pain and vaginal discharge during their postpartum stay in health facilities and at each postnatal care contact. Women should be advised of danger signs and symptoms, including any exacerbation of uterine pain as a manifestation of postpartum complications such as endometritis.

Summary of evidence and considerations: Pharmacological interventions compared with placebo

Effects of the interventions (EB Table A.2.3a)

Evidence was derived from an updated Cochrane systematic review on the relief of pain due to uterine cramping/involution after vaginal birth *(46)*. The review included 28 trials involving 2749 women, of which 25 trials (2600 women) provided data for analyses. Twelve trials had two comparison arms. The rest were multi-arm trials (from three to seven groups). All trials were small, with the number of women allocated to the individual comparison arms ranging from 7 to 63. Trials were conducted in the Islamic Republic of Iran (8), Norway (2), the USA (13) and the Bolivarian Republic of Venezuela (2). Most trials were published prior to the year 2000. All included trials compared any type of analgesia with another type of analgesia, placebo or no treatment. Pharmacological agents were compared with placebo in 17 trials (1800 women), of which 15 trials (1411 women) contributed data.

Comparison 1: Paracetamol (oral, single-dose) compared with placebo

Maternal outcomes

Relief of symptoms: It is uncertain whether paracetamol (650 mg) provides adequate pain relief for uterine cramping/involution when compared with placebo (very low-certainty evidence).

Health service use: It is uncertain whether paracetamol (1000 mg) affects the need for additional pain relief for uterine cramping/involution when compared with placebo (very low-certainty evidence).

Adverse effects: It is uncertain whether paracetamol has any effect on adverse effects when compared with placebo (very low-certainty evidence). Subgroup analyses according to dose of paracetamol (650 mg or 1000 mg) showed the same level of uncertainty.

Maternal functioning/well-being and *experience of postnatal care* were not reported in the included trials.

Newborn outcomes

Breastfeeding status and *adverse effects* were not reported in the included trials.

Additional considerations

Paracetamol is considered safe during breastfeeding *(59)*. Although a single case of a rash on the upper trunk of a breastfeeding infant has been described, the American Academy of Pediatrics considers paracetamol compatible with breastfeeding. No other adverse effects of paracetamol exposure through breastmilk have been reported. Following the mother's treatment with 1000 mg of paracetamol, it has been estimated that the maximum dose her infant is exposed to is less than 2% of the maternal dose *(59)*.

Values

See Box 3.1 in section 3.A.2: Interventions for common physiological signs and symptoms.

Resources

No economic evaluations of pharmacological treatments for relieving pain due to uterine cramping/involution were identified.

Equity

No direct evidence was identified on the impact on health equity of pharmacological methods for relieving pain due to uterine cramping/involution. Pharmacological interventions for relieving pain due to uterine cramping/involution may increase equity in some settings, as many of these interventions are widely available without a prescription and at low cost. However, pharmacological interventions may decrease equity if women are expected to pay for analgesics themselves.

Additional considerations

Women should be informed of the effects (desirable and undesirable) of the respective available pharmacological options to relieve pain due to uterine cramping/involution.

Acceptability

See Box 3.2 in section 3.A.2: Interventions for common physiological signs and symptoms.

Additional considerations

Some women may decline paracetamol due to fears of harmful effects on the baby through breastmilk. Women who use paracetamol need clear information about the dose of paracetamol in any concurrent medications to help avoid inadvertent overdose or toxicity *(51)*. Generally, it is anticipated that women will accept single-dose, oral analgesics with clear information about safety.

Feasibility

See Box 3.3 in section 3.A.2: Interventions for common physiological signs and symptoms.

Additional considerations

Generally, paracetamol is widely available at low cost. Paracetamol (tablet: 100 mg to 500 mg; suppository: 100 mg) is listed in the WHO Model List of Essential Medicines *(58)*.

Table 3.8 Main resource requirements for paracetamol (oral, single-dose)

Resource	Description
**Staff**	• Doctors/midwives/nurses
**Training**	• Practice-based training for health workers
**Supplies**	• Paracetamol 500 mg (oral preparation) = US$ 0.004 per tablet/capsule *(57)*
**Equipment**	• On-site pharmacy and/or medicine stock management system that is managed by a trained pharmacist or dispenser
**Infrastructure**	• Dispensing time estimated to be 2–5 minutes
**Supervision and monitoring**	• Same as for usual care

Table 3.9 Summary of judgements: Paracetamol (oral, single-dose) compared with placebo

Domain	Judgement
Desirable effects	Don't know
Undesirable effects	Small
Certainty of the evidence	Very low
Values	Probably no important uncertainty or variability
Balance of effects	Don't know
Resources required	Negligible costs or savings
Certainty of the evidence on required resources	No included studies
Cost-effectiveness	Don't know
Equity	Varies
Acceptability	Varies
Feasibility	Probably yes

Comparison 2: NSAIDs compared with placebo

Maternal outcomes

Relief of symptoms: Low-certainty evidence suggests NSAIDs may provide adequate pain relief for uterine cramping/involution when compared with placebo (11 trials, 946 women; RR 1.66, 95% CI 1.45 to 1.91). Subgroup analyses according to the type of NSAIDs showed:

- *Aspirin 650 mg:* Low-certainty evidence suggests aspirin 650 mg may provide adequate pain relief for uterine cramping/involution when compared with placebo (6 trials, 282 women; RR 1.33, 95% CI 1.09 to 1.61).
- *Other NSAIDs:* It is uncertain whether naproxen 275 mg, 300 mg, 550 mg or 600mg, flurbiprofen 50 mg, ketorolac 5 mg or 10 mg, or fenoprofen 12.5 mg, 25 mg, 50 mg, 100 mg, 200 mg or 300 mg provides adequate pain relief for uterine cramping/involution when compared with placebo (very low-certainty evidence).

Health service use: It is uncertain whether NSAIDs reduce the need for additional pain relief for uterine cramping/involution when compared with placebo (very low-certainty evidence). Subgroup analyses according to type of NSAIDs (aspirin 650 mg; ketorolac 5 mg or 10 mg; naproxen 275 mg, 300 mg, 550 mg and 600 mg) showed the same level of uncertainty.

Adverse effects: It is uncertain whether NSAIDs affect adverse effects when compared with placebo (very low-certainty evidence). Subgroup analyses according to type of NSAIDs (aspirin 650 mg; fenoprofen 200 mg; flurbiprofen 50 mg; ketorolac 5 mg or 10 mg; naproxen 275 mg, 300 mg, 550 mg and 600 mg) showed the same level of uncertainty.

Maternal functioning/well-being and *experience of postnatal care* were not reported in the included trials.

Newborn outcomes

Breastfeeding status and *adverse effects* were not reported in the included trials.

Table 3.10 Main resource requirements for NSAIDs

Resource	Description
**Staff**	• Doctors/midwives/nurses
**Training**	• Practice-based training for health workers, or else none required
**Supplies**	• NSAIDs (oral preparation, price per tablet/capsule) *(57):*<br>– acetylsalicylic acid (aspirin) 500 mg = US$ 0.005<br>– ibuprofen 400 mg = US$ 0.01<br>– diclofenac sodium 50 mg = US$ 0.005
**Equipment and infrastructure**	• On-site pharmacy and/or medicine stock management system that is managed by a trained pharmacist or dispenser
**Time**	• Dispensing time estimated to be 2–5 minutes
**Supervision and monitoring**	• Same as for usual care

Values

See Box 3.1 in section 3.A.2: Interventions for common physiological signs and symptoms.

Resources

No economic evaluations of pharmacological treatments for relieving pain due to uterine cramping/involution were identified.

Equity

No direct evidence was identified on the impact on health equity of pharmacological methods for the relief of pain due to uterine cramping/involution. Other evidence around equity is the same as for *Comparison 1: Paracetamol (oral, single-dose) compared with placebo*.

Additional considerations

Additional considerations around equity are the same as for *Comparison 1: Paracetamol (oral, single-dose) compared with placebo*.

Acceptability

See Box 3.2 in section 3.A.2: Interventions for common physiological signs and symptoms.

Additional considerations

Some women may decline to use medications due to fears of harmful effects on the baby through breastmilk. Women who use medications to relieve pain need clear information about dosage and contraindications. Generally, it is anticipated that women will accept single-dose, oral analgesics with clear information about safety.

Feasibility

See Box 3.3 in section 3.A.2: Interventions for common physiological signs and symptoms.

Additional considerations

Generally, NSAIDs are widely available at low cost.

The only non-steroidal anti-inflammatory medicines listed in the WHO Model List of Essential Medicines are acetylsalicylic acid (aspirin) (tablet: 100 mg to 500 mg; suppository: 50 mg to 150 mg) and ibuprofen (tablet: 200 mg; 400 mg; 600 mg) *(58)*.

Table 3.11 Summary of judgements: NSAIDs compared with placebo

Domain	Judgement
Desirable effects	Small
Undesirable effects	Varies
Certainty of the evidence	Very low
Values	Probably no important uncertainty or variability
Balance of effects	Probably favours NSAIDs
Resources required	Negligible costs or savings
Certainty of the evidence on required resources	No included studies
Cost-effectiveness	Don't know
Equity	Varies
Acceptability	Varies
Feasibility	Probably yes

Comparison 3: Opioids compared with placebo

Maternal outcomes

Relief of symptoms: It is uncertain whether opioids provide adequate pain relief for uterine cramping/involution when compared with placebo (very low-certainty evidence). Subgroup analyses according to dose of opioid (codeine 60 mg or 120 mg) showed the same level of uncertainty.

Health service use: It is uncertain whether opioids affect the need for additional pain relief for uterine cramping/involution when compared with placebo (very low-certainty evidence). Subgroup analyses according to type and dose of opioid (codeine 60 mg or 120 mg, and nalbuphine 15 mg) showed the same level of uncertainty.

Adverse effects: It is uncertain whether opioids affect adverse effects when compared with placebo (very low-certainty evidence). Subgroup analyses according to dose of opioid (codeine 60 mg or 120 mg) showed the same level of uncertainty.

Maternal functioning/well-being and *experience of postnatal care* were not reported in the included trials.

Newborn outcomes

Breastfeeding status and *adverse effects* were not reported in the included trials.

Table 3.12 Main resource requirements for opioids

Resource	Description
**Staff**	• A physician is usually needed to prescribe opioids (this is not the case in all countries; in some settings midwives can also prescribe opioids)
**Training**	• Training to administer opioids is required as per practice-based training for health workers and training to monitor and manage adverse effects and complications of opioid use
**Supplies**	• Codeine 30 mg (oral preparation) = US$ 0.09 per tablet/capsule *(57)*<br>• Nalbuphine Chorhydrate 10 mg/ml = US$ 1.44/ml *(57)*
**Equipment and infrastructure**	• On-site pharmacy and/or medicine stock management system that is managed by a trained pharmacist or dispenser
**Time**	• Dispensing time estimated to be 2–5 minutes
**Supervision and monitoring**	• Supervision of administration and monitoring for adverse effects<br>• Secure method of storing opioids and recording opioid use to avoid abuse

Values

See Box 3.1 in section 3.A.2: Interventions for common physiological signs and symptoms.

Resources

No economic evaluations of pharmacological treatments for relieving pain due to uterine cramping/involution were identified.

Additional considerations

While in some high-resource settings oral opioid drugs are considered relatively inexpensive, these drugs may not be accessible in all settings and may not be affordable in some LMICs.

Equity

No direct evidence was identified on the impact on health equity of pharmacological methods for relieving pain due to uterine cramping/involution. Use of expensive opioid alternatives might have a negative impact on equity if these are preferentially used in high-resource settings and advantaged populations. Other evidence around equity is the same as for the previous comparisons.

Additional considerations

Additional considerations around equity are the same as in the previous comparisons.

Acceptability

See Box 3.2 in section 3.A.2: Interventions for common physiological signs and symptoms.

Additional considerations

Some women may decline opioids due to fears of adverse effects and harmful effects on the baby through breastmilk. Some women may decline opioids due to the need to rely on health workers to administer the medication.

Feasibility

See Box 3.3 in section 3.A.2: Interventions for common physiological signs and symptoms.

Table 3.13 Summary of judgements: Opioids compared with placebo

Domain	Judgement
Desirable effects	Don't know
Undesirable effects	Varies
Certainty of the evidence	Very low
Values	Probably no important uncertainty or variability
Balance of effects	Don't know
Resources required	Moderate costs
Certainty of the evidence on required resources	No included studies
Cost-effectiveness	Don't know
Equity	Probably reduced
Acceptability	Varies
Feasibility	Varies

Additional considerations

In lower-resource settings, where opioids are not widely available and used, there are likely to be financial implications as well as additional training requirements for their administration and the management of potential maternal and neonatal adverse effects. It is likely that the type of opioid used in different settings and countries would be influenced by the cost of the medication.

Codeine phosphate (tablet: 30 mg) is listed in the WHO Model List of Essential Medicines *(58)*.

Summary of evidence and considerations: Pharmacological interventions compared with other pharmacological interventions

Effects of the interventions (EB Table A.2.3b)

The evidence for this summary is derived from an updated Cochrane systematic review on the relief of pain due to uterine cramping/involution after vaginal birth *(46)*. The review includes 28 trials involving 2749 women, of which 25 trials (2600 women) contributed data. Twelve trials had two comparison arms. The rest were multi-arm trials (from three to seven groups). All trials were small, with the number of women allocated to the individual comparison arms ranging from 7 to 63. Trials were conducted in the Islamic Republic of Iran (8), Norway (2), the USA (13) and the Bolivarian Republic of Venezuela (2). Most trials were published prior to the year 2000.

All included trials compared one type of analgesia with another type of analgesia. Pharmacological agents were compared with other classes of pharmacological agents, or with drugs of the same class at different doses, in 20 trials (2262 women), of which 19 trials (1969 women) contributed data. Herbal medicines were compared in eight trials (736 women), given as pills or capsules. In six trials, some of the alternative comparisons included medications that are no longer in use due to severe adverse effects; only arms with currently used medications were included in the review.

Eight trials (1051 women) evaluating opioids and different NSAIDs clearly specified that breastfeeding was an exclusion criterion. One trial evaluating herbal medicines versus NSAIDs (126 women) included lactating women. The remaining 11 trials (1085 women) did not specify the breastfeeding status of women.

Comparison 1: Lower dose of an oral analgesic compared with a higher dose of the same analgesic

Comparison 1a: Naproxen (lower dose compared with a higher dose)

Maternal outcomes

Relief of symptoms: It is uncertain whether naproxen 300 mg provides adequate pain relief as reported by women when compared with a higher dose of naproxen (600 mg) (very low-certainty evidence).

Adverse effects: It is uncertain whether naproxen 300 mg affects the risk of adverse effects when compared with a higher dose of naproxen (600 mg) (very low-certainty evidence).

Health service use, maternal functioning/well-being and *experience of postnatal* care were not reported in the included trials.

Newborn outcomes

Breastfeeding status and *adverse effects* were not reported in the included trials.

Comparison 1b: Ketorolac (lower dose compared with a higher dose)

Maternal outcomes

Relief of symptoms: It is uncertain whether ketorolac 5 mg provides adequate pain relief as reported by women when compared with a higher dose of ketorolac (10 mg) (very low-certainty evidence).

Health service use: It is uncertain whether ketorolac 5 mg affects the need for additional pain relief when compared with a higher dose of ketorolac (10 mg) (very low-certainty evidence).

Adverse effects: It is uncertain whether ketorolac 5 mg affects the risk of adverse effects when compared with a higher dose of ketorolac (10 mg) (very low-certainty evidence).

Maternal functioning/well-being and *experience of postnatal care* were not reported in the included trials.

Newborn outcomes

Breastfeeding status and *adverse effects* were not reported in the included trials.

Comparison 1c: Codeine (lower dose compared with a higher dose)

Maternal outcomes

Relief of symptoms: It is uncertain whether codeine 60 mg provides adequate pain relief as reported by women when compared with a higher dose of codeine (120 mg) (very low-certainty evidence).

Health service use: It is uncertain whether codeine 60 mg affects the need for additional pain relief when compared with a higher dose of codeine (120 mg) (very low-certainty evidence).

Adverse effects: It is uncertain whether codeine 60 mg affects the risk of adverse effects when compared with a higher dose of codeine (120 mg) (very low-certainty evidence).

Maternal functioning/well-being and *experience of postnatal care* were not reported in the included trials.

Newborn outcomes

Breastfeeding status and *adverse effects* were not reported in the included trials.

Values

See Box 3.1 in section 3.A.2: Interventions for common physiological signs and symptoms.

Resources

No economic evaluations of pharmacological treatments for relieving pain due to uterine cramping/involution were identified.

Additional considerations

There is no large variation in cost based on dosage of the same class of analgesic.

Equity

No direct evidence was identified on the impact on health equity of pharmacological methods for the relief of pain due to uterine cramping/involution. Pharmacological interventions for relieving pain due to uterine cramping/involution may increase equity in some settings, as many of these interventions are widely available without a prescription and at low cost. However, pharmacological interventions may decrease equity if women are expected to pay for analgesics. Impacts on equity may be similar for lower or higher doses of the same class of analgesic.

Additional considerations

Women should be informed of the effects (desirable and undesirable) of the respective available pharmacological options to relieve pain due to uterine cramping/involution.

Table 3.14 Main resource requirements for the oral analgesics

Resource	Description
**Staff**	• Doctors/midwives/nurses<br>• Opioids: a physician is usually needed to prescribe opioids (this is not the case in all countries; in some settings midwives can also prescribe opioids)
**Training**	• Training to administer opioids as per practice-based training for health workers, and training to monitor and manage adverse effects and complications of opioid use
**Supplies**	• Paracetamol 500 mg (oral preparation) = US$ 0.004 per tablet/capsule *(57)*<br>• Codeine 30 mg tablets: US$ 0.0904 to US$ 0.25 per tablet/capsule *(57)*
**Equipment and infrastructure**	• On-site pharmacy and/or medicine stock management system that is managed by a trained pharmacist or dispenser
**Time**	• Dispensing time estimated to be 2–5 minutes
**Supervision and monitoring**	• Paracetamol: same as for usual care<br>• Opioids: supervision of administration and monitoring for adverse effects; secure method of storing opioids and recording opioid use to avoid abuse

Acceptability

See Box 3.2 in section 3.A.2: Interventions for common physiological signs and symptoms.

Additional considerations

Some women may decline medications due to fears of harmful effects on the baby through breastmilk. Women who use medications to relieve pain need clear information about dosage and contraindications. Generally, it is anticipated that women will accept single-dose, oral analgesics with clear information about safety. Women may prefer to use the lowest dose possible to relief pain due to uterine cramping/involution.

Feasibility

See Box 3.3 in section 3.A.2: Interventions for common physiological signs and symptoms.

Additional considerations

Health workers would generally prefer to prescribe analgesics using the lowest dose possible, and to prescribe those associated with fewer adverse effects.

In lower-resource settings, where opioids are not widely available and used, there are likely to be financial implications as well as additional training requirements for their administration and the management of potential maternal and neonatal adverse effects. It is likely that the type of opioid used in different settings and countries would be influenced by the cost of the medication.

The only non-opioids and non-steroidal anti-inflammatory medicines listed in the WHO Model List of Essential Medicines are acetylsalicylic acid (aspirin) (tablet: 100 mg to 500 mg; suppository: 50 mg to 150 mg), ibuprofen (tablet: 200 mg; 400 mg; 600 mg), and paracetamol (tablet: 100 mg to 500 mg; suppository: 100 mg) *(58)*.

The opioid codeine phosphate (tablet: 30 mg) is listed in the WHO Model List of Essential Medicines *(58)*.

Table 3.15 Summary of judgements: Lower dose of an oral analgesic compared with a higher dose of the same analgesic

Domain	Judgement
Desirable effects	Don't know
Undesirable effects	Don't know
Certainty of the evidence	Very low
Values	Probably no important uncertainty or variability
Balance of effects	Don't know
Resources required	Negligible costs or savings
Certainty of the evidence on required resources	No included studies
Cost-effectiveness	Don't know
Equity	Varies
Acceptability	Varies
Feasibility	Probably yes

Comparison 2: An oral analgesic compared with an alternative oral analgesic of the same class

Comparison 2a: Aspirin compared with naproxen

Maternal outcomes

Relief of symptoms: It is uncertain whether aspirin (650 mg) provides adequate pain relief for uterine cramping/involution as reported by women when compared with naproxen (275 mg) (very low-certainty evidence).

Adverse effects: It is uncertain whether aspirin (650 mg) has any effect on adverse effects when compared with naproxen (275 mg) (very low-certainty evidence).

Health service use, maternal functioning/well-being and *experience of postnatal care* were not reported in the included trials.

Newborn outcomes

Breastfeeding status and *adverse effects* were not reported in the included trials.

Comparison 2b: Aspirin compared with flurbiprofen

Maternal outcomes

Relief of symptoms: It is uncertain whether aspirin (650 mg) provides adequate pain relief for uterine cramping/involution as reported by women when compared with flurbiprofen (50 mg) (very low-certainty evidence).

Health service use: It is uncertain whether aspirin (650 mg) affects the need for additional pain relief when compared with flurbiprofen (50 mg) (very low-certainty evidence).

Adverse effects: It is uncertain whether aspirin (650 mg) has any effect on adverse effects when compared with flurbiprofen (50 mg) (very low-certainty evidence).

Maternal functioning/well-being and *experience of postnatal care* were not reported in the included trials.

Newborn outcomes

Breastfeeding status and *adverse effects* were not reported in the included trials.

Comparison 2c: Aspirin compared with ketorolac

Maternal outcomes

Relief of symptoms: It is uncertain whether any dose of aspirin provides adequate pain relief for uterine cramping/involution as reported by women when compared with any dose of ketorolac (very low-certainty evidence). Subgroup analyses according to dose of analgesic (ketorolac 5 mg or 10 mg) showed the same level of uncertainty.

Health service use: It is uncertain whether any dose of aspirin affects the need for additional pain relief when compared with any dose of ketorolac (very low-certainty evidence). Subgroup analyses according to dose of analgesic (ketorolac 5 mg or 10 mg) showed the same level of uncertainty.

Adverse effects: It is uncertain whether aspirin has any effect on adverse effects when compared with any dose of ketorolac (very low-certainty evidence). Subgroup analyses according to dose of analgesic (ketorolac 5 mg or 10 mg) showed the same level of uncertainty.

Maternal functioning/well-being and *experience of postnatal care* were not reported in the included trials.

Newborn outcomes

Breastfeeding status and *adverse effects* were not reported in the included trials.

Comparison 2d: Codeine compared with nalbuphine

Maternal outcomes

Health service use: It is uncertain whether codeine 60 mg affects the need for additional pain relief when compared with nalbuphine 15 mg (very low-certainty evidence).

Relief of symptoms, health service use, maternal functioning/well-being and *experience of postnatal care* were not reported in the included trials.

Newborn outcomes

Breastfeeding status and *adverse effects* were not reported in the included trials.

Additional considerations

Additional considerations around the effects of the medications on pain due to uterine cramping/involution are the same as for *Comparison 1: Lower dose of an oral analgesic compared with a higher dose of the same analgesic*.

Values

See Box 3.1 in section 3.A.2: Interventions for common physiological signs and symptoms.

Resources

Evidence and resources required are the same as for *Comparison 1: Lower dose of an oral analgesic compared with a higher dose of the same analgesic*.

Equity

No direct evidence was identified on the impact on health equity of pharmacological treatments for the relief of pain due to uterine cramping/involution. Other evidence around equity of use of the medications is the same as for *Comparison 1: Lower dose of an oral analgesic compared with a higher dose of the same analgesic*.

Additional considerations

Additional considerations around equity are the same as for *Comparison 1: Lower dose of an oral analgesic compared with a higher dose of the same analgesic*.

Acceptability

See Box 3.2 in section 3.A.2: Interventions for common physiological signs and symptoms.

Additional considerations

Additional considerations around acceptability are the same as for *Comparison 1: Lower dose of an oral analgesic compared with a higher dose of the same analgesic*.

Feasibility

See Box 3.3 in section 3.A.2: Interventions for common physiological signs and symptoms.

Additional considerations

Additional considerations around feasibility are the same as for *Comparison 1: Lower dose of an oral analgesic compared with a higher dose of the same analgesic*.

Table 3.16 Summary of judgements: An oral analgesic compared with an alternative oral analgesic of the same class

Domain	Judgement
Desirable effects	Don't know
Undesirable effects	Varies
Certainty of the evidence	Very low
Values	Probably no important uncertainty or variability
Balance of effects	Don't know
Resources required	Negligible costs or savings
Certainty of the evidence on required resources	No included studies
Cost-effectiveness	Don't know
Equity	Varies
Acceptability	Varies
Feasibility	Probably yes

Comparison 3: An oral analgesic compared with an alternative oral analgesic from a different class

Comparison 3a: Paracetamol compared with NSAIDs

Maternal outcomes

Relief of symptoms: It is uncertain whether paracetamol 650 mg provides adequate pain relief for uterine cramping/involution when compared with NSAIDs (aspirin 650 mg) (very low-certainty evidence).

Adverse effects: It is uncertain whether paracetamol has any effect on adverse effects when compared with NSAIDs (very low-certainty evidence). Subgroup analyses according to type and dose of analgesic (paracetamol 650 mg versus aspirin 650 mg; or paracetamol 1000 mg versus naproxen 500 mg) showed the same level of uncertainty.

Health service use, maternal functioning/well-being and *experience of postnatal care* were not reported in the included trials.

Newborn outcomes

Breastfeeding status and *adverse effects* were not reported in the included trials.

Comparison 3b: NSAIDs compared with opioids

Maternal outcomes

Relief of symptoms: Low-certainty evidence suggests NSAIDs may provide adequate pain relief for uterine cramping/involution when compared with opioids (5 trials, 560 women; RR 1.33, 95% CI 1.13 to 1.57). Subgroup analyses according of type and dose of analgesic showed the following.

- *Aspirin versus codeine:* It is uncertain whether aspirin (650 mg) provides adequate pain relief for uterine cramping/involution when compared with codeine (at doses of 60 mg or 120 mg) (very low-certainty evidence).
- *Fenoprofen versus codeine:* It is uncertain whether fenoprofen (at doses of 12.5 mg, 25 mg, 50 mg, 100 mg, 200 mg or 300 mg) provides adequate pain relief for uterine cramping/involution when compared with codeine 60 mg (very low-certainty evidence).
- *Flurbiprofen versus codeine:* It is uncertain whether flurbiprofen (50 mg) provides adequate pain relief for uterine cramping/involution when compared with codeine (at doses of 60 mg or 120 mg) (very low-certainty evidence).

- *Naproxen versus codeine:* It is uncertain whether naproxen (at doses of 300 mg or 600 mg) provides adequate pain relief for uterine cramping/involution when compared with codeine (60 mg) (very low-certainty evidence).

Health service use: It is uncertain whether NSAIDs reduce the need for additional pain relief for uterine cramping/involution when compared with opioids (very low-certainty evidence). Subgroup analyses according to type and dose of analgesic (aspirin 650 mg versus codeine 60 mg or 120 mg; flurbiprofen 50 mg versus codeine 60 mg or 120 mg; and naproxen 300 mg or 600 mg versus codeine 60 mg) showed the same level of uncertainty.

Adverse effects: It is uncertain whether NSAIDs affect adverse effects when compared with opioids (very low-certainty evidence). Subgroup analyses according to type and dose of analgesic (aspirin 650 mg versus codeine 60 mg or 120 mg; flurbiprofen 50 mg versus codeine 60 mg or 120 mg; naproxen 300 mg or 600 mg versus codeine 60 mg; and fenoprofen 200 mg versus codeine 60 mg) showed the same level of uncertainty.

Health service use, maternal functioning/well-being and *experience of postnatal care* were not reported in the included trials.

Newborn outcomes
Breastfeeding status and *adverse effects* were not reported in the included trials.

Comparison 3c: NSAIDs compared with herbal analgesia

Maternal outcomes
Relief of symptoms: Moderate-certainty evidence suggests NSAIDs probably provide adequate pain relief for uterine cramping/involution when compared with herbal analgesia (4 trials, 394 women; RR 0.96, 95% CI 0.78 to 1.18). Subgroup analyses according to the type and dose of analgesic showed the following.

- It is uncertain whether mefenamic acid 250 mg provides adequate pain relief for uterine cramping/involution when compared with pimpinella anisum, apium graveolens and crocus sativus 500 mg, Melissa officinalis 395 mg, or fennel 300 mg (very low-certainty evidence).
- It is uncertain whether ibuprofen 400 mg provides adequate pain relief for uterine cramping/involution when compared with fennel essence 20% (very low-certainty evidence).

Health service use: It is uncertain whether NSAIDs affect the need for additional pain relief for uterine cramping/involution when compared with herbal analgesia (very low-certainty evidence). Subgroup analyses according to type and dose of analgesic (ibuprofen 400 mg versus fennel essence 20%) showed the same level of uncertainty

Adverse effects: It is uncertain whether NSAIDs have any effect on adverse effects when compared with herbal analgesia (very low-certainty evidence). Subgroup analyses according to type and dose of analgesic (mefenamic acid 250 mg versus pimpinella anisum, apium graveolens and crocus sativus 500 mg) showed the same level of uncertainty.

Health service use, maternal functioning/well-being and *experience of postnatal care* were not reported in the included trials.

Newborn outcomes
Breastfeeding status and *adverse effects* were not reported in the included trials.

Values

See Box 3.1 in section 3.A.2: Interventions for common physiological signs and symptoms.

Resources

No economic evaluations of pharmacological treatments for relieving pain due to uterine cramping/involution were identified.

Table 3.17 Main resource requirements for the oral analgesics assessed

Resource	Description
**Staff**	• Doctors/midwives/nurses<br>• Opioids: A physician is usually needed to prescribe opioids (this is not the case in all countries; in some settings midwives can also prescribe opioids)
**Training**	• Training to administer opioids is required as per practice-based training for health workers and training to monitor and manage adverse effects and complications of opioid use
**Supplies**	• Analgesic drugs – all oral preparation, price per tablet/capsule *(57)*:<br>– codeine 30 mg = US$ 0.09<br>– paracetamol 500 mg = US$ 0.004<br>– NSAIDs:<br>· acetylsalicylic acid (aspirin) 500 mg = US$ 0.005<br>· ibuprofen 400 mg = US$ 0.01<br>· diclofenac sodium 50 mg = US$ 0.005
**Equipment and infrastructure**	• On-site pharmacy and/or medicine stock management system that is managed by a trained pharmacist or dispenser
**Time**	• Dispensing time estimated to be 2–5 minutes
**Supervision and monitoring**	• Paracetamol and NSAIDs: same as for usual care<br>• Opioids: supervision of administration and monitoring for adverse effects; secure method of storing opioids and recording opioid use to avoid abuse

Equity

No direct evidence was identified on the impact on health equity of pharmacological treatments for the relief of pain due to uterine cramping/involution. Other evidence around equity is the same as for the previous comparisons.

Additional considerations

Additional considerations around equity are the same as for the previous comparisons.

Acceptability

See Box 3.2 in section 3.A.2: Interventions for common physiological signs and symptoms.

Additional considerations

Additional considerations around acceptability are the same as for the previous comparisons.

Feasibility

See Box 3.3 in section 3.A.2: Interventions for common physiological signs and symptoms.

Additional considerations

Additional considerations around feasibility are the same as for the previous comparisons.

Table 3.18 Summary of judgements: An oral analgesic compared with an alternative oral analgesic from a different class

Domain	Judgement
Desirable effects	Don't know
Undesirable effects	Varies
Certainty of the evidence	Very low
Values	Probably no important uncertainty or variability
Balance of effects	Don't know
Resources required	Negligible costs or savings
Certainty of the evidence on required resources	No included studies
Cost-effectiveness	Don't know
Equity	Varies
Acceptability	Varies
Feasibility	Probably yes

A.2.4 Postnatal pelvic floor muscle training (PFMT) for pelvic floor strengthening

RECOMMENDATION 7

For postpartum women, starting routine pelvic floor muscle training (PFMT) after childbirth for the prevention of postpartum urinary and faecal incontinence is not recommended. *(Not recommended)*

Remarks

- In this context, PFMT refers to the performance of repeated voluntary contractions of the pelvic floor muscles, according to a protocol that outlines the frequency (one or more sets of exercises per day), intensity and progression of exercises, as well as the duration of the training period (e.g. at least several days of the week, for at least eight weeks) and may include maintenance pelvic floor muscle exercises after initial training.
- While PFMT started after childbirth is not recommended as a preventive measure, women with involuntary loss of small volumes of urine (urinary stress incontinence) after childbirth should be advised of the potential benefits of PFMT for treatment of urinary incontinence. For these women, in the absence of stronger evidence, the Guideline Development Group (GDG) agreed that unsupervised pelvic floor exercises performed at home may be beneficial and are unlikely to cause harmful effects. Pelvic floor muscle exercises may also positively affect sexual function in the postnatal period and promote self-care.
- All women should be informed during pregnancy and postnatally about potential pelvic floor problems, including urinary or faecal incontinence after childbirth.
- The GDG recognized that the effects of PFMT started in early pregnancy for pregnant women who do not have incontinence were not evaluated during the guideline process.

Summary of evidence and considerations

Effects of the interventions (EB Table A.2.4)

Evidence was derived from an updated Cochrane systematic review of PFMT for preventing and treating urinary and faecal incontinence in antenatal and postnatal women *(48)*. For the purpose of this guideline, only evidence from the trials evaluating PFMT initiated in the postpartum period were included. The data were derived from 19 RCTs with 5452 women, conducted largely in HICs. One multicentre trial was conducted across New Zealand and the United Kingdom.

Fourteen trials (4293 women) reported postnatal PFMT for mixed prevention and treatment of incontinence. For women who were continent at enrolment, PFMT was intended as a preventive treatment, while for women who had symptoms of incontinence at enrolment, PFMT was intended as a therapeutic treatment. Women were randomized to postnatal PFMT versus usual care versus no PFMT. From these, nine trials (3651 women) reported on the outcomes of interest. Due to the mixed population of women recruited in these trials, the relative effects of PFMT as a preventive treatment versus PFMT as a therapeutic treatment could not be disentangled.

Five trials (1159 women) included postpartum women who reported leakage of urine, faeces or both. One trial recruited women within six weeks post-birth, one between 10 and 16 weeks, and three trials recruited women at or beyond three months post-birth. Women were randomly allocated to supervised PFMT (as a treatment for incontinence) or to controls (women not receiving PFMT or receiving usual care). Only four trials (1061 women) reported on the outcomes of interest.

Comparison 1: Postnatal PFMT compared with no intervention or usual care for (mixed) prevention or treatment of incontinence

Relief of symptoms: Low-certainty evidence suggests PFMT may improve urinary incontinence in the early postnatal period (0–3 months) when compared with no PFMT (2 trials, 321 women; RR 0.54, 95% CI 0.44 to 0.66). It is uncertain whether PFMT affects urinary incontinence in the mid-postnatal period (> 3–6 months) when compared with usual care (very low-certainty evidence). Low-certainty evidence suggests PFMT may have little or no effect on urinary incontinence in the late postnatal period (> 6–12 months) when compared with no PFMT or usual care (3 trials, 826 women; RR 0.88, 95%

CI 0.71 to 1.09). It is uncertain whether PFMT affects faecal incontinence in the early postnatal period (0–3 months) or faecal incontinence in the late postnatal period (> 6–12 months) when compared with no PFMT (very low-certainty evidence).

Maternal functioning/well-being: It is uncertain whether PFMT affects postnatal quality of life (related to urinary incontinence) when compared with no PFMT (very low-certainty evidence).

Long-term maternal morbidity and *adverse effects* were not reported in the included trials. *Experience of postnatal care* was not reported in the systematic review.

Comparison 2: Postnatal PFMT compared with no intervention or usual care for treatment of incontinence

Relief of symptoms: It is uncertain whether PFMT affects urinary incontinence in the late postnatal period (> 6–12 months) following childbirth compared with control (very low-certainty evidence). It is uncertain whether PFMT affects faecal incontinence in the late postnatal period (> 6–12 months).

Long-term maternal morbidity: Low-certainty evidence suggests PFMT may make little or no difference to urinary incontinence in the long term (> 5–10 years) when compared with usual care (1 trial, 516 women; RR 0.96, 95% CI 0.88 to 1.05). Low-certainty evidence suggests PFMT may make little or no difference to urinary incontinence in the very long term (> 10 years) when compared with usual care (1 trial, 471 women; RR 1.03, 95% CI 0.94 to 1.12). It is uncertain whether PFMT affects faecal incontinence in the long term (> 5–10 years), or faecal incontinence in the very long term (> 10 years), when compared with usual care (very low-certainty evidence).

Maternal functioning/well-being: It is uncertain whether PFMT affects urinary incontinence-specific quality of life when compared with usual care (very low-certainty evidence).

Adverse effects were not reported in the included trials. *Experience of postnatal care* was not reported in the systematic review.

Additional considerations

Additional evidence from the Cochrane systematic review *(48)* suggests beginning PFMT early in pregnancy probably prevents urinary incontinence in late pregnancy and reduces the risk of incontinence during the postpartum period, in particular at three to six months postpartum. There is insufficient evidence on the effects beyond six months postpartum.

Values

See Box 3.1 in section 3.A.2: Interventions for common physiological signs and symptoms.

Resources

The Cochrane systematic review included a systematic search of full economic evaluations (cost-effectiveness analyses, cost–utility analyses and cost–benefit analyses), conducted as part of a single empirical study such as a randomized controlled trial, a model based on a single such study, or a model based on several such studies. No economic studies were identified.

Additional considerations

The Cochrane systematic review identified one protocol for an ongoing economic evaluation conducted alongside a RCT (240 pregnant and/or postpartum women with stress urinary incontinence; anticipated completion December 2020) *(60)*. For PFMT programmes with a supervised component (either as part of initial training or for the full duration on the programme), PFMT provided as part of a group may be more cost-effective than providing individual sessions *(60)*.

Table 3.19 Main resource requirements for postnatal PFMT for pelvic floor strengthening

Resource	Description
**Staff**	• Physiotherapist, midwife, nurse or other health worker
**Training**	• Certification in physiotherapy and/or postnatal exercise<br>• Training in postpartum pelvic floor muscle exercises (for midwives and nurses)
**Supplies**	• For home-based or unsupervised training, information (written and/or pictorial, e.g. leaflets)
**Equipment and infrastructure**	• Varies depending on programme; some may require no equipment, others may incorporate a chair for sitting, yoga mat (sufficient floor space needed) and/or exercise equipment (e.g. exercise ball), and group classes require a designated room
**Time**	• *Time to train:* varies, depending on the programme<br>• *Time to perform:* varies depending on the programme<br>• Many exercises can be performed unsupervised and do not require ongoing supervision/coaching
**Supervision and monitoring**	• Not required

Equity

No direct evidence on the impact on health equity of PFMT was identified. PFMT may decrease equity, as it can be more difficult for women to access it due to limited service availability and potential out-of-pocket costs, in particular if PFMT is provided by specialist personnel. PFMT may have no effect on or may increase equity if PFMT and exercises can be delivered or supervised by midwives or nurses, or performed unsupervised (with instruction).

Additional considerations

An interpretive synthesis of individual, professional and service issues associated with the implementation of PFMT for childbearing women *(61)* reported PFMT may not be easily accessible to non-English speaking women or women with low health literacy. Some women may feel a sense of shame because of symptoms of urinary incontinence and may refrain from disclosing the issue to their care providers *(61)*.

Acceptability

See Box 3.2 in section 3.A.2: Interventions for common physiological signs and symptoms.

In addition, a qualitative systematic review exploring PFMT adherence (incorporating several studies with postnatal women) found that individuals experienced substantial difficulties with capability (particularly knowledge and skills), motivation (especially associated with the considerable cognitive demands of PFMT) and opportunity (as external factors generate competing priorities) when adopting and maintaining a PFMT programme *(62)*.

Feasibility

A qualitative evidence synthesis of women's experiences of postnatal care found no direct evidence relating to women's views on the feasibility of using PFMT for the prevention or treatment of urinary or faecal incontinence *(28)*. Indirect evidence indicates that women may find it difficult to accommodate a PFMT programme as they struggle with competing demands on their time and prioritize the needs of their baby (high confidence in the evidence).

A qualitative evidence synthesis of health workers' experiences of postnatal care found no direct evidence relating to views on the feasibility of using PFMT for the prevention or treatment of urinary or faecal incontinence *(29)*. However, indirect evidence suggests that lack of personnel, resources and training may limit the offer PFMT, provision of information, and counselling on potential pelvic floor problems in the postnatal period (moderate confidence in the evidence).

An interpretive synthesis of individual, professional and service issues associated with the implementation of PFMT for childbearing women *(61)* reported women lack knowledge of urinary incontinence and PFMT that may diminish their likelihood of engaging with such training. As there may be some taboo and fatalism surrounding urinary incontinence following childbirth, some women may not feel comfortable discussing symptoms or treatment with their care providers *(61)*. Likewise, health workers may be reluctant to raise the issue with women as part of routine care *(61)*. The synthesis noted a lack of training and support for midwives to complete requisite training to enable delivery of PFMT as part of maternity care, and limited access to specialist physiotherapists in some regions *(61)*. The synthesis also reported that postnatal women performing PFMT would value assessment to confirm whether they are carrying out pelvic floor muscle contractions correctly. However, objective assessment may not be a part of regular service provision, and the acceptability of such an assessment to midwives and women is unknown *(61)*.

Table 3.20 Summary of judgements: Postnatal PFMT compared with usual care or no intervention

Domain	Judgement
Desirable effects	Don't know
Undesirable effects	Don't know
Certainty of the evidence	Very low
Values	Probably no important uncertainty or variability
Balance of effects	Don't know
Resources required	Varies
Certainty of the evidence on required resources	No included studies
Cost-effectiveness	Don't know
Equity	Varies
Acceptability	Probably yes
Feasibility	Varies

A.2.5 Non-pharmacological interventions to treat postpartum breast engorgement

RECOMMENDATION 8

For treatment of breast engorgement in the postpartum period, women should be counselled and supported to practice responsive breastfeeding, good positioning and attachment of the baby to the breast, expression of breastmilk, and the use of warm or cold compresses, based on a woman's preferences. *(Recommended)*

Remarks

- In making this recommendation, the Guideline Development Group acknowledged that the evidence was insufficient to conclude on the added value of cabbage-leaf cream, cold cabbage leaves, cold gel packs, warm herbal compress and breast massage over usual breastfeeding counselling and support for the treatment of breast engorgement during breastfeeding, which were often incorporated into the control arms of the trials evaluated.
- Some women may find that the non-pharmacological interventions evaluated relieve breast pain and hardness and may choose to use these methods. Women should be informed that it is unclear whether these treatment options for breast engorgement have adverse effects, due to a paucity of data.
- In this context, responsive breastfeeding *(63, 64)* refers to the mother responding to her baby's cues, as well as her own desire to breastfeed. Responsive feeding is distinct from demand feeding, as it recognizes the reciprocal mother–baby relationship and benefits of breastfeeding beyond the alleviation of hunger.
- All women should be advised of common breast conditions associated with lactation, such as sore or cracked nipples, engorgement and mastitis, and encouraged to report any signs and symptoms to their care providers.

Summary of evidence and considerations

Effects of the interventions (EB Table A.2.5)

Evidence was derived from an updated Cochrane systematic review of 21 trials involving 2170 women *(49)*. Of these, 18 trials (1996 women) evaluated non-pharmacological interventions for treatment of breast engorgement during lactation. Fourteen trials were RCTs and four were quasi-RCTs. Trials were conducted in a mix of LMICs and HICs.

Non-pharmacological treatments included cool, warm or room-temperature cabbage leaves directly applied to the breast; cabbage leaf extract creams; hot and/or cold compresses (alone or combined with herbal compounds); cold or chilled gel packs; Ghua-Sha (scraping) therapy; and different massage techniques.

This evidence summary includes only results from trials comparing an intervention to no intervention, or to placebo or to usual care. Four trials (353 women) assessed the use of cabbage leaves directly applied to the breast versus a control group including another intervention or usual care. Two trials (62 women) were excluded from the review, as the results were reported per individual breast rather than per woman. One trial was excluded from this summary as it compared the use of room-temperature cabbage leaves applied directly to the breast versus hot water bags. Four trials reported that women in both groups received information and advice on breastfeeding, while another two reported that only women in the control arm received information and advice on breastfeeding. In addition, usual care in some trials included the use of warm compresses (3 trials).

The review authors were unable to pool the results from the trials in a meta-analysis because of heterogeneity of interventions, assessment and reporting of outcomes.

Comparison 1: Cabbage leaf extract cream compared with placebo

One trial (39 women) compared a 1% cabbage leaf extract cream versus a placebo cream.

Maternal outcomes

Relief of symptoms: It is uncertain whether the use of a cream with 1% cabbage leaf extract reduces breast pain or breast engorgement (measured using a six-point, self-rated scale developed by Hill and Humenick)[33] when compared with placebo cream (very low-certainty evidence).

Adverse effects and *maternal functioning/well-being* were not reported in the included trial. *Short-term maternal morbidity, health service use* and *experience of postnatal care* were not reported in the systematic review.

Newborn/infant outcomes

Breastfeeding status was not reported in the included trial. *Adverse effects* were not reported in the systematic review.

Values

See Box 3.1 in section 3.A.2: Interventions for common physiological signs and symptoms.

Additional considerations

Health workers would generally place high value on promoting breastfeeding and on interventions that may help them to provide better advice and support to breastfeeding women.

Resources

No economic evaluations of non-pharmacological interventions for treating breast engorgement following childbirth were identified.

33 Hill PD, Humenick SS. The occurrence of breast engorgement. J Hum Lact. 1994;10(2):79-86. doi: 10.1177/089033449401000212.

Table 3.21 Main resource requirements for cabbage leaf extract cream

Resource	Description
**Staff**	• Midwife/nurse, or else none required where self-administered
**Training**	• Practice-based midwifery or nursing training, or else none required
**Supplies**	• Cabbage leaf cream = approximately US$ 30.00 per 60 ml tube
**Equipment and infrastructure**	• Calm, safe room conducive to privacy (curtain, door, wall)
**Time**	• Applied liberally to both breasts and left on for 2 hours
**Supervision and monitoring**	• Same as for usual care

Equity

No direct evidence was identified on the impact on health equity of non-pharmacological interventions for treating breast engorgement following childbirth. Cabbage leaf extract cream may be prohibitively expensive for some women and may decrease equity. It is unlikely cabbage leaf extract cream will be supplied by the health service. However, if it can be supplied by the health service, it may have no effect on or may increase equity.

Additional considerations

Even though the health, emotional, psychosocial and societal benefits of breastfeeding to women and children is recognized, breastfeeding rates worldwide are suboptimal, especially among low-income women. Increasing breastfeeding initiation and duration among low-income women, including prevention of breast problems that may affect breastfeeding continuation, would not only offer improved health benefits to the mother and infant, but would lessen the economic burden on this group within the community *(65, 66)*.

Acceptability

A qualitative evidence synthesis of women's experiences of postnatal care found no direct evidence relating to women's views on non-pharmacological treatments for breast engorgement *(28)*. However, indirect evidence from this review suggests that women would appreciate any techniques or treatments that provide relief from breast engorgement (high confidence in the evidence) as they are likely to enhance the development of the mother–infant relationship, improve self-perception of body image and increase psychosocial well-being (high confidence in the evidence). Findings from the same review also indicate that, in some contexts, women may prefer to use traditional practices including diet, medicinal plants (cabbage leaves or other), massage or spiritual healing to treat problems associated with breastfeeding (moderate confidence in the evidence).

Additional considerations

Most women would probably accept a simple intervention involving application of cream to the breasts to reduce engorgement and potentially prevent complications such as mastitis. However, some breastfeeding women may be reluctant to apply a cream directly to the breast, if they are concerned about their baby ingesting the cream while feeding and/or if they have to remove the cream before breastfeeding.

Feasibility

See Box 3.3 in section 3.A.2: Interventions for common physiological signs and symptoms.

Additional considerations

Application of cream is a simple and easy intervention that is expected to be feasibly implemented in most settings.

Table 3.22 Summary of judgements: Cabbage leaf extract cream compared with placebo

Domain	Judgement
Desirable effects	Don't know
Undesirable effects	Don't know
Certainty of the evidence	Very low
Values	Probably no important uncertainty or variability
Balance of effects	Don't know
Resources required	Moderate costs
Certainty of the evidence on required resources	No included studies
Cost-effectiveness	Don't know
Equity	Varies
Acceptability	Varies
Feasibility	Probably yes

Comparison 2: Cold cabbage leaves applied directly to the breast compared with usual care

One three-arm trial (228 women) compared the use of cold cabbage leaves applied directly to the breast with either usual care (including daily, in-house postnatal classes during rounds conducted by lactation consultants, and brochures in the hospital) or cold gel packs. The cold cabbage leaves versus standard care arm is considered in this comparison.

Maternal outcomes

Relief of symptoms: Moderate-certainty evidence suggests cold cabbage leaves probably reduce breast pain when compared with usual care (1 trial, 152 women; MD 1.03 lower, 95% CI 1.53 lower to 0.53 lower). Moderate-certainty evidence suggests cold cabbage leaves probably reduce breast hardness (measured using a breast engorgement assessment scale) when compared with usual care (1 trial, 152 women; MD 0.58 lower, 95% CI 0.82 lower to 0.34 lower).

Maternal functioning/well-being: Moderate-certainty evidence suggests cold cabbage leaves probably increase women's satisfaction (satisfied or very satisfied) when compared with usual care (1 trial 152 women; RR 1.42, 95% CI 1.22 to 1.64).

Adverse effects were not reported in the included trial. *Short-term maternal morbidity, health service use* and *experience of postnatal care* were not reported in the systematic review.

Newborn/infant outcomes

Breastfeeding duration: Low-certainty evidence suggests cold cabbage leaves may make little or no difference to the risk of cessation of breastfeeding before six months when compared with usual care (1 trial, 108 women; RR 1.75, 95% CI 0.93 to 3.30).

Adverse effects were not reported in the systematic review.

Values

See Box 3.1 in section 3.A.2: Interventions for common physiological signs and symptoms.

Additional considerations

Additional considerations around the values of health workers are the same as for *Comparison 1: Cabbage leaf extract cream compared with placebo*.

Resources

No economic evaluations of non-pharmacological interventions for treating breast engorgement following childbirth were identified.

Table 3.23 Main resource requirements for cold cabbage leaves applied directly to the breast

Resource	Description
**Staff**	• Midwife/nurse, or else none required where self-administered
**Training**	• Practice-based midwifery or nursing training, or else none required
**Supplies**	• Chilled cabbage leaves from the common green cabbage<br>• Availability and cost vary by region; indicative cost = US$ 0.5–1.3 per cabbage head<br>• Kitchen utensils or other sharp implement to prepare leaves
**Equipment and infrastructure**	• Refrigeration and cold storage facilities (including electricity)<br>• Access to clean water to wash and prepare leaves<br>• Calm, safe room conducive to privacy (curtain, door, wall)
**Time**	• Application time 2–8 hours (with fresh leaves introduced every 2 hours)
**Supervision and monitoring**	• Same as for usual care

Equity

No direct evidence was identified on the impact on health equity of non-pharmacological interventions for treating breast engorgement following childbirth. The impact of cold cabbage leaves for treating breast engorgement following childbirth is likely to vary by setting. This intervention requires access to clean water, refrigeration and cold storage, which is limited in many low-income countries. Fresh cabbage is commonly available in many areas, but may be limited where there is restricted access to fresh produce. Availability of cabbage may vary by region. Where the necessary supplies and facilities are readily available, cold cabbage leaves may have no effect on or may increase equity.

Additional considerations

Additional considerations around equity are the same as for *Comparison 1: Cabbage leaf extract cream compared with placebo*.

Acceptability

Evidence around acceptability is the same as for *Comparison 1: Cabbage leaf extract cream compared with placebo*.

Additional considerations

Most women would probably accept a simple intervention such as application of cold cabbage leaves to the breasts to reduce engorgement and potentially prevent complications such as mastitis.

Feasibility

See Box 3.3 in section 3.A.2: Interventions for common physiological signs and symptoms.

Additional considerations

Access to refrigeration and clean water is limited in many low-income settings. Access to fresh produce may also be limited in some settings.

Table 3.24 Summary of judgements: Cold cabbage leaves compared with usual care

Domain	Judgement
Desirable effects	Moderate
Undesirable effects	Don't know
Certainty of the evidence	Moderate
Values	Probably no important uncertainty or variability
Balance of effects	Probably favours cold cabbage leaves
Resources required	Negligible costs or savings
Certainty of the evidence on required resources	No included studies
Cost-effectiveness	Don't know
Equity	Varies
Acceptability	Probably yes
Feasibility	Varies

Comparison 3: Cold gel packs applied directly to the breast compared with usual care

One three-arm trial (228 women) compared the use of cold cabbage leaves directly applied to the breast with either standard care or cold gel packs. The cold gel packs (chilled gel pack applied to each breast for 2 hours followed by half hour break before repeat application) plus usual care, versus usual care alone (including daily in-house postnatal classes during rounds conducted by lactation consultants, brochures in the hospital) is considered in this comparison. Another trial (88 women) compared breast-shaped cold gel packs (worn 15–20 minutes after two consecutive feeds) with usual care. Results of this trial were not included in the summary tables of the review due to high rates of crossover among groups.

Maternal outcomes

Relief of symptoms: Low-certainty evidence suggests cold gel packs may make little or no difference to breast pain when compared with usual care (1 trial, 151 women; MD 0.4 lower, 95% CI 0.91 lower to 0.11 higher). Moderate-certainty evidence suggests cold gel packs probably reduce the number of women with breast hardness when compared with usual care (1 trial, 151 women; MD 0.34 lower, 95% CI 0.6 lower to 0.08 lower).

Maternal functioning/well-being: Low-certainty evidence suggests cold gel packs may make little or no difference to women's satisfaction (the number of women who were satisfied or very satisfied) when compared with usual care (1 trial, 151 women; RR 1.17, 95% CI 0.97 to 1.40).

Adverse effects were not reported in the included trial. *Short-term maternal morbidity, health service use* and *experience of postnatal care* were not reported in the systematic review.

Newborn/infant outcomes

Breastfeeding status: Low-certainty evidence suggests cold gel packs may make little or no difference to cessation of breastfeeding before six months when compared with usual care (1 trial, 109 women; RR 1.03, 95% CI 0.50 to 2.14).

Adverse effects were not reported in the systematic review.

Values

See Box 3.1 in section 3.A.2: Interventions for common physiological signs and symptoms.

Additional considerations

Additional considerations around the values of health workers are the same as for the previous comparisons.

Resources

No economic evaluations of non-pharmacological interventions for treating breast engorgement following childbirth were identified.

Equity

No direct evidence was identified on the impact on health equity of pharmacological interventions for treating breast engorgement following childbirth. The impact of cold gel packs on health equity is likely to vary across settings. These treatments require access to refrigeration and cold storage, which is limited in

Table 3.25 Main resource requirements for cold gel packs applied directly to the breasts

Resource	Description
**Staff**	• Midwife/nurse, or else none required where self-administered
**Training**	• Practice-based midwifery or nursing training, or else none required
**Supplies**	• Warm and/or cool reusable breast packs or similar (approximately US$ 20.00 per pack of two)<br>• Towels or other skin barrier
**Equipment and infrastructure**	• Refrigeration and cold storage facilities (including electricity)<br>• Calm, safe room conducive to privacy (curtain, door, wall)
**Time**	• Application time varies from 15 minutes to up to 4 hours (with breaks in application)
**Supervision and monitoring**	• Same as for usual care

many low-income countries. Availability of gel pads may also be limited in these settings.

Additional considerations

Additional considerations around equity are the same as for the previous comparisons.

Acceptability

Evidence around acceptability is the same as for the previous comparisons.

Additional considerations

Most women would probably accept a simple intervention such as application of gel packs to the breasts to reduce engorgement and potentially prevent complications such as mastitis.

Feasibility

See Box 3.3 in section 3.A.2: Interventions for common physiological signs and symptoms.

Additional considerations

Access to refrigeration and gel pads is limited in many low-income countries.

Table 3.26 Summary of judgements: Cold gel packs applied directly to the breasts compared with usual care

Domain	Judgement
Desirable effects	Small
Undesirable effects	Don't know
Certainty of the evidence	Low
Values	Probably no important uncertainty or variability
Balance of effects	Does not favour either
Resources required	Moderate costs
Certainty of the evidence on required resources	No included studies
Cost-effectiveness	Don't know
Equity	Varies
Acceptability	Probably yes
Feasibility	Varies

Comparison 4: Warm herbal compresses compared with usual care (including warm compresses without herbs)

Three trials (610 women) compared warm or hot herbal compresses with usual care (including warm compresses without herbs). One trial compared hot herbal compress balls (with Cassumunar ginger, turmeric and camphor) with compress balls without herbs (500 women). Another trial compared warm compresses followed by hollyhock leaf compresses with warm compresses alone (40 women). The last trial compared warm ginger compresses with routine care (76 women), but results were reported per individual breast and therefore were not included in the systematic review.

Maternal outcomes

Relief of symptoms: Moderate-certainty evidence suggests herbal compress balls probably reduce breast pain when compared with usual care (including warm compress balls without herbs) (1 trial, 500 women; MD 1.8 lower, 95% CI 2.07 lower to 1.53 lower). It is uncertain whether hollyhock leaf compresses have any effect on breast engorgement when compared with usual care (including warm compresses without herbs).

Adverse effects: It is uncertain whether herbal compress balls have any effect on adverse effects when compared with usual care (very low-certainty evidence).

Maternal functioning/well-being was not reported in the included trials. *Short-term maternal morbidity, health service use* and *experience of postnatal care* were not reported in the systematic review.

Newborn/infant outcomes

Breastfeeding status was not reported in the included trial. *Adverse effects* were not reported in the systematic review.

Additional considerations

The trial reported that two women in the herbal compress balls group experienced skin irritation compared with none in the control group (2/250 and 0/250, respectively).

Values

See Box 3.1 in section 3.A.2: Interventions for common physiological signs and symptoms.

Additional considerations

Additional considerations around the values of health workers are the same as for the previous comparisons.

Resources

No economic evaluations of non-pharmacological interventions for treating breast engorgement following childbirth were identified.

Additional considerations

A herbalist may be required to prepare hollyhock solution. In some settings, women might be able to prepare this and other herbal solutions themselves at home.

Equity

No direct evidence was identified on the impact on health equity of non-pharmacological interventions for treating breast engorgement following childbirth. The impact of warm herbal compresses for treating breast engorgement following childbirth is likely to vary by setting. Treatment with herbal compresses may decrease equity, as they might be difficult for women to access due to limited availability and potential out-of-pocket costs, in particular if the materials are provided by a herbalist or similar and cannot be accessed locally and/or prepared at home. Growth of herbs such as hollyhock may vary by region.

Additional considerations

Additional considerations around equity are the same as for the previous comparisons.

Acceptability

Evidence around acceptability is the same as for the previous comparisons.

Additional considerations

Most women would probably accept a simple intervention such as application of a warm herbal compress applied to the breasts to reduce engorgement and potentially prevent complications such as mastitis.

Feasibility

See Box 3.3 in section 3.A.2: Interventions for common physiological signs and symptoms.

Additional considerations

Feasibility may be limited in settings where the materials can only be provided by a herbalist or similar and cannot be accessed locally and/or prepared at home.

Table 3.27 Main resource requirements for warm compresses (with or without herbs)

Resource	Description
**Staff**	• Midwife/nurse, or else none required where self-administered<br>• A herbalist may be required to prepare hollyhock solution
**Training**	• Practice-based midwifery or nursing training, or else none required<br>• Training in herbal medicine as appropriate
**Supplies**	• Herbal or other solution (e.g. hollyhock or ginger) and packaging (ball, compress, pad)<br>• Towels or other skin barrier
**Equipment and infrastructure**	• Heating facilities, water boiling equipment and facilities<br>• Facilities to prepare herbal solutions (e.g. hollyhock leaves and stem are dried, milled and exposed to ultraviolet light) *(67)*<br>• For application, calm, safe room conducive to privacy (curtain, door, wall)
**Time**	• Application time varies from 10–20 minutes for a single application to up to 1.5 hours three times per day for two days
**Supervision and monitoring**	• Same as for usual care

Table 3.28 Summary of judgements: Warm compresses (with or without herbs) compared with usual care

Domain	Judgement
Desirable effects	Small
Undesirable effects	Don't know
Certainty of the evidence	Low
Values	Probably no important uncertainty or variability
Balance of effects	Don't know
Resources required	Varies
Certainty of the evidence on required resources	No included studies
Cost-effectiveness	Don't know
Equity	Varies
Acceptability	Probably yes
Feasibility	Varies

Comparison 5: Breast massage compared with usual care (without breast massage)

One trial compared Oketani breast massage[34] versus usual care (education on proper breastfeeding techniques, frequent breastfeeding and hot compress). The trial was excluded from the analyses as the results were reported per individual breast rather than per woman.

Another three-arm trial (200 women) compared breast massage alternating with cactus and aloe cold compresses, with cactus and aloe cold compresses alone, as well as with breast massage alone. Review authors only presented in this comparison the arms evaluating massage therapy plus cactus and aloe compresses versus cactus and aloe compresses alone; the arms comparing breast massage only versus cactus and aloe compresses alone were not presented in the review.

Additional considerations

The Cochrane systematic review included two trials considering ultrasound therapy and one on electromechanical massage. These trials were not included in this framework as ultrasound therapy was considered technically demanding and not feasible at the global level.

Values

See Box 3.1 in section 3.A.2: Interventions for common physiological signs and symptoms.

Additional considerations

Additional considerations around the values of health workers are the same as for the previous comparisons.

Resources

No economic evaluations of non-pharmacological interventions for treating breast engorgement following childbirth were identified.

Additional considerations

The cost of breast massage provided by professional massage therapists could be relatively high, depending on location and setting.

34 Connective tissue massage developed by midwife Sotomi Oketani.

Table 3.29 Main resource requirements for breast massage

Resource	Description
**Staff**	• Midwife/nurse, or specialist massage therapist (who is permitted to perform breast massage)
**Training**	• Training in postnatal breast massage (for midwives or nurses) or else certification in massage therapy with licence to offer breast massage
**Supplies**	• Varies by specific technique but includes:<br>– information (written and/or pictorial, e.g. leaflets) (where self-administered)<br>– warmed towels and natural massage lubricant<br>– chair, bed or massage table
**Equipment and infrastructure**	• Heating facilities (according to region)<br>• Calm, safe room conducive to privacy (curtain, door, wall)<br>• Chair, bed or massage table
**Time**	• Varies by technique (e.g. the Oketani method is for 30 minutes once per day for two consecutive days) *(68)*
**Supervision and monitoring**	• Same as for usual care

Equity

No direct evidence was identified on the impact on health equity of non-pharmacological interventions for treating breast engorgement following childbirth. The impact of breast massage for treating breast engorgement following childbirth is likely to vary by setting. Breast massage may decrease equity, as it can be difficult for women to access due to limited service availability and potential out-of-pocket costs, in particular if the service is provided by specialist personnel. Where breast massage can be performed by midwives or other health personnel with specialist training, or can be self-administered with instruction, it may have no effect on or may increase equity.

Additional considerations

Additional considerations around equity are the same as for the previous comparisons.

Acceptability

Evidence around acceptability is the same as for the previous comparisons.

Feasibility

See Box 3.3 in section 3.A.2: Interventions for common physiological signs and symptoms.

Additional considerations

Feasibility may be limited in settings where breast massage can only be performed by a specialist massage therapist. Where maternity staff can access training to provide breast massage, or massage is self-administered, the intervention may be feasible.

Table 3.30 Summary of judgements: Breast massage compared with usual care

Domain	Judgement
Desirable effects	Don't know
Undesirable effects	Don't know
Certainty of the evidence	No included studies
Values	Probably no important uncertainty or variability
Balance of effects	Don't know
Resources required	Varies
Certainty of the evidence on required resources	No included studies
Cost-effectiveness	Don't know
Equity	Varies
Acceptability	Probably yes
Feasibility	Varies

A.2.6 Pharmacological interventions to treat postpartum breast engorgement

RECOMMENDATION 9

The use of pharmacological interventions such as subcutaneous oxytocin and proteolytic enzyme therapy for the treatment of breast engorgement in the postpartum period is not recommended.
(Not recommended)

Remarks

- In making this recommendation, the Guideline Development Group emphasized breastfeeding counselling and support as the treatment of choice for breast engorgement after childbirth (see Recommendation 8 in this guideline).
- All women should receive continued breastfeeding advice and support and decide on breast engorgement treatment options based on their individual preferences.

Summary of evidence and considerations

Effects of the interventions (EB Table A.2.6)

Evidence was derived from an updated Cochrane systematic review of 21 trials involving 2170 women *(49)*. Of these, three trials (174 women) evaluated pharmacological interventions for the treatment of breast engorgement during lactation.

One of the included trials was an RCT and two were quasi-RCTs. Trials were conducted in Japan, Singapore and Sweden. Most of the trials recruited women with swollen, hard, painful breasts (with or without difficulty with breastfeeding). Pharmacological treatments included subcutaneous oxytocin, oral protease complex tablets and oral serrapeptase (anti-inflammatory proteolytic enzymes). The duration of the interventions varied from a single application to treatments given for up to three days. All trials reported that women in both groups received information and advice on breastfeeding. Follow-up varied from 15 minutes to six months after the intervention. Most trials followed women for two to seven days, or until improvement of symptoms.

The review authors were unable to pool the results from the trials in a meta-analysis because of heterogeneity of interventions, assessment and reporting of outcomes.

Comparison 1: Subcutaneous oxytocin compared with placebo

One trial (45 women) compared daily oxytocin 2.5 IU given subcutaneously until breasts became soft versus placebo.

Maternal outcomes

Relief of symptoms: It is uncertain whether the use of subcutaneous oxytocin has any effect on breast engorgement at three days of treatment when compared with placebo (very low-certainty evidence).

Adverse effects were not reported in the included trial. *Short-term maternal morbidity, health service use, maternal functioning/well-being*, and *experience of postnatal care* were not reported in the systematic review.

Newborn/infant outcomes

Breastfeeding status and *adverse effects* were not reported in the systematic review.

Additional considerations

No trials assessed the effects and safety of other forms of oxytocin (oral or nasal spray) on breast engorgement.

Values

See Box 3.1 in section 3.A.2: Interventions for common physiological signs and symptoms.

Additional considerations

Health workers would generally place high value on promoting breastfeeding and on interventions that may help them to provide better advice and support to breastfeeding women.

Resources

No economic evaluations of pharmacological interventions for treating breast engorgement were identified.

Table 3.31 Main resource requirements for subcutaneous oxytocin

Resource	Description
**Staff**	• Oxytocin requires subcutaneous administration by skilled health workers (doctors/ midwives/nurses)
**Training**	• Practice-based training for health workers to administer injections and monitor and manage expected and unexpected adverse effects is required, as per standard maternity staff training<br>• Some additional training may be required if subcutaneous route of administration of oxytocin is introduced in settings where it has not previously been available
**Supplies**	• Oxytocin indicative cost:<br>– 5 IU (injectable) = US$ 0.1885 per ml *(57)*<br>– 10 IU: US$ 0.22–1.19 per ml *(69, 70)*<br>• Needles and syringes<br>• Alcohol swabs<br>• Sharps container
**Equipment and infrastructure**	• On-site pharmacy and/or medicine stock management system that is managed by a trained pharmacist or dispenser, and bed or massage table<br>• Cold chain storage and transport
**Time**	• Dispensing time estimated to be 2–5 minutes
**Supervision and monitoring**	• Supervision and monitoring to ensure appropriate use, stock availability and quality

Equity

No direct evidence was identified on the impact on health equity of pharmacological interventions for treating breast engorgement following childbirth. The impact on health equity of subcutaneous oxytocin is likely to vary by geographical region and context. Oxytocin is relatively inexpensive and widely available in a range of settings. However, inconsistent stock levels and heat sensitivity may limit use in under-resourced LMICs, particularly in isolated rural areas.

Additional considerations

Even though the health, emotional, psychosocial and societal benefits of breastfeeding to women and children are recognized, breastfeeding rates worldwide are suboptimal, especially among low-income women. Increasing breastfeeding initiation and duration among low-income women, including prevention of breast problems that may affect breastfeeding continuation, would not only offer improved health benefits to the mother and infant, but would lessen the economic burden on this group within the community *(65, 66)*.

Acceptability

A qualitative evidence synthesis of women's experiences of postnatal care found no direct evidence relating to women's views on pharmacological interventions for relieving the symptoms of breast engorgement *(21)*. Indirect evidence from this review suggests that women would appreciate any interventions that provide relief from breast engorgement (high confidence in the evidence) as they are likely to enhance the development of the mother–infant relationship, improve self-perception of body image and increase psychosocial well-being (high confidence in the evidence). However, findings from the same review also indicate that, in some contexts, women may prefer to use traditional practices including diet, medicinal plants (cabbage leaves or other), massage or spiritual healing to treat problems associated with breastfeeding (moderate confidence in the evidence).

Additional considerations

An invasive and painful procedure involving a daily injection may not be acceptable to many

women, especially given limited evidence on its effects, and where other pharmacological and non-pharmacological treatment options are available.

Feasibility

See Box 3.3 in section 3.A.2: Interventions for common physiological signs and symptoms.

Additional considerations

Resource constraints may influence effective use of oxytocin in LMICs. Inconsistent supplies and reservations about oxytocin storage in areas with limited/inconsistent electricity hinder utilization. However, injectable oxytocin may be available in health facilities as it is already widely used globally for other indications (e.g. the prevention and treatment of postpartum haemorrhage, and induction of labour).

Oxytocin (10 IU in 1 ml for injection) is listed in the WHO Model List of Essential Medicines *(58)*.

Table 3.32 Summary of judgements: Subcutaneous oxytocin compared with placebo

Domain	Judgement
Desirable effects	Don't know
Undesirable effects	Don't know
Certainty of the evidence	Very low
Values	Probably no important uncertainty or variability
Balance of effects	Don't know
Resources required	Moderate costs
Certainty of the evidence on required resources	No included studies
Cost-effectiveness	Don't know
Equity	Varies
Acceptability	Probably no
Feasibility	Varies

Comparison 2: Proteolytic enzymes compared with placebo

Comparison 2a: Oral protease complex compared with placebo

One trial (59 women) published in 1965 compared oral administration of protease complex (enteric-coated tablet consisting of bromelain and trypsin) with placebo. It was unclear whether all women included in the trial were breastfeeding.

Maternal outcomes

Relief of symptoms: It is uncertain whether the use of oral protease complex has any effect on breast pain, or breast swelling, when compared with placebo (very low-certainty evidence).

Adverse effects: It is uncertain whether oral protease has any effect on adverse effects when compared with placebo, as narrative evidence was assessed as very low-certainty.

Short-term maternal morbidity, health service use, maternal functioning/well-being and *experience of postnatal care* were not reported in the systematic review.

Newborn/infant outcomes

Breastfeeding status and *adverse effects* were not reported in the systematic review.

Comparison 2b: Oral serrapeptase compared with placebo

One trial (70 women) compared oral serrapeptase (Danzen), an anti-inflammatory proteolytic enzyme drug derived from *Serratia E15* (isolated from the silkworm intestine) versus placebo. The authors gave cumulative percentages in the results section, which the review authors corrected. The trial authors reported that breastfeeding was encouraged during the study but only four women in the treatment group and eight in the placebo group breastfed their babies during the trial period.

Maternal outcomes

Relief of symptoms: It is uncertain whether oral serrapeptase has any effect on breast pain or breast swelling when compared with placebo (very low-certainty evidence). Low-certainty evidence suggests oral serrapeptase may reduce breast engorgement when compared with placebo (1 trial, 70 women; RR 0.36, 95% CI 0.14 to 0.88).

Adverse effects: It is uncertain whether oral serrapeptase has any effect on adverse effects compared with placebo, as narrative evidence was assessed as very low-certainty.

Short-term maternal morbidity, health service use, maternal functioning/well-being, and *experience of postnatal care* were not reported in the systematic review.

Newborn/infant outcomes

Breastfeeding status and *adverse effects* were not reported in the systematic review.

Additional considerations

Included trials reported no adverse effects in either the protease, serrapeptase, or placebo group.

Bromelain and serrapeptase are also marketed as dietary/nutritional supplements and are widely available for purchase without a prescription. A systematic review of the evidence around use of serratiopeptidase concluded there is insufficient evidence to support its use as an analgesic and health supplement *(71)*.

Values

See Box 3.1 in section 3.A.2: Interventions for common physiological signs and symptoms.

Resources

No economic evaluations of pharmacological interventions for treating breast engorgement were identified.

Equity

No direct evidence was identified on the impact on health equity of pharmacological interventions for treating breast engorgement following childbirth. Proteolytic enzymes may be prohibitively expensive for some women, and therefore may decrease equity. Their availability is also likely to vary by region. However, proteolytic enzymes may have no effect on or may increase equity where the treatments are provided by health facilities.

Table 3.33 Main resource requirements for proteolytic enzymes

Resource	Description
**Staff**	• Doctors/midwives/nurses, or else none required (where purchased privately as a dietary/nutritional supplement by the woman)
**Training**	• Practice-based training for health workers, or else none required
**Supplies**	• Proteolytic enzymes (e.g. bromelain, serrapeptase, oral administration) = approximately US$ 20–30 for 90 capsules (US$ 0.22–0.33 per tablet/capsule)
**Equipment and infrastructure**	• On-site pharmacy and/or medicine stock management system that is managed by a trained pharmacist or dispenser
**Time**	• Dispensing time estimated to be 2–5 minutes<br>• Multiple tablets taken multiple times daily
**Supervision and monitoring**	• Same as for usual care

Additional considerations

Additional considerations around equity are the same as for *Comparison 1: Subcutaneous oxytocin compared with placebo*.

Acceptability

Evidence around acceptability is the same as for *Comparison 1: Subcutaneous oxytocin compared with placebo*.

Additional considerations

It is anticipated that swallowing tablets containing ingredients to assist the body in breaking down protein would be an acceptable intervention for most women. However, with limited information about the safety profile of proteolytic enzymes, such as the long-term safety of serrapeptase *(71)*, it is unlikely that breastfeeding women will accept this intervention.

Feasibility

See Box 3.3 in section 3.A.2: Interventions for common physiological signs and symptoms.

Additional considerations

Proteolytic enzymes are available in some settings, over-the-counter or online, as a dietary supplement. Indicative prices suggest these supplements might be prohibitively expensive for some women.

No proteolytic enzymes are listed in the WHO Model List of Essential Medicines *(58)*.

Table 3.34 Summary of judgements: Proteolytic enzymes compared with placebo

Domain	Judgement
Desirable effects	Don't know
Undesirable effects	Don't know
Certainty of the evidence	Very low
Values	Probably no important uncertainty or variability
Balance of effects	Don't know
Resources required	Moderate costs
Certainty of the evidence on required resources	No included studies
Cost-effectiveness	Don't know
Equity	Varies
Acceptability	Probably no
Feasibility	Probably no

A.3 PREVENTIVE MEASURES

Background

The GDG considered the evidence and other relevant information to inform recommendations on the prevention of the following conditions.

Mastitis

Mastitis is an inflammatory condition of the breast, presenting with breast pain, redness and swelling, which may or may not be accompanied by infection *(50)*. Approximately one in four women breastfeeding during the first 26 weeks postpartum experience mastitis *(72)*. Non-infective mastitis may result from milk stasis, blocked ducts, engorgement, or nipple or breast tissue damage often associated with poor positioning and attachment of the infant at the breast and incomplete emptying of the breasts *(50)*. Infective mastitis may result from cracked or traumatized nipples and may lead to abscess formation *(50)*. Prevention of mastitis usually involves effective removal of milk, ensuring good infant positioning and attachment, massaging the breast during feeding, as well as supportive measures such as rest, adequate fluids, the application of warm compresses and oral analgesia.

Postpartum constipation

Constipation refers to infrequent, hard, dry or bulky stools that are difficult or painful to pass, a feeling of incomplete evacuation or obstruction, or the need for manual manoeuvres to complete the evacuation *(73)*. The prevalence of self-reported postpartum constipation is 15–62% *(74)*. Causes of postpartum constipation include hormonal changes during pregnancy and the puerperium, pelvic floor disorders (including perineal pain after childbirth and perineal trauma), fear of perineal wound breakdown, haemorrhoids, and adverse effects of iron supplementation or drugs received during pregnancy and childbirth (e.g. analgesics, opiates, magnesium sulphate or enemas) *(74)*. Disrupted eating during active labour and in the immediate days postpartum may negatively affect bowel movements. In the later postpartum period, cultural practices and diet restrictions, disrupted food and water consumption, and emotional concerns may also affect bowel movements *(74)*. Strategies for preventing constipation include pharmacological interventions (e.g. laxatives) and non-pharmacological interventions (e.g. dietary and lifestyle modification and advice on positioning during bowel movements) *(74)*.

In addition to the GDG recommendation on the above, this section of the guideline includes four sets of recommendations on preventive measures that have been integrated from WHO guidelines on preventing maternal infections that are relevant to routine postnatal care.

Box 3.4 Values

Findings from a qualitative evidence synthesis exploring what women want from postnatal care *(21)* indicate that women want a positive experience in which they are able to adapt to their new self-identity and develop a sense of confidence and competence as a mother. They also want to adjust to changes in their intimate and family relationships (including their relationship to their baby), navigate ordinary physical and emotional challenges, and experience the dynamic achievement of personal growth as they adjust to their new normal, both as parents and as individuals in their own cultural context.

A.3.1 Non-pharmacological interventions to prevent postpartum mastitis

RECOMMENDATION 10

For the prevention of mastitis in the postpartum period, women should be counselled and supported to practise responsive breastfeeding, good positioning and attachment of the baby to the breast, hand expression of breastmilk, and the use of warm or cold compresses, based on a woman's preferences. *(Recommended)*

Remarks

- In making this recommendation, the Guideline Development Group acknowledged that the evidence was insufficient to conclude on the added value of probiotics, anti-secretory factor-inducing foods, acupoint massage, and specialist breastfeeding education over usual breastfeeding advice and support for the prevention of mastitis during breastfeeding, interventions that were often incorporated into the control arms of the trials evaluated.
- In this context, responsive breastfeeding *(63, 64)* refers to the mother responding to her baby's cues, as well as her own desire to breastfeed. Responsive feeding is distinct from demand feeding, as it recognizes the reciprocal mother–baby relationship and benefits of breastfeeding beyond alleviation of hunger.
- All women should be advised of common breast conditions associated with lactation, such as sore or cracked nipples, engorgement and mastitis, and encouraged to report any signs and symptoms to their care providers.
- Providers should support women to continue breastfeeding with breast engorgement if they wish to, as per the 2017 WHO guideline *Protecting, promoting and supporting breastfeeding in facilities providing maternity and newborn services (75)*.
- All women should receive breastfeeding counselling in accordance with the 2018 WHO guideline *Counselling of women to improve breastfeeding practices (76)*.

Summary of evidence and considerations

Effects of the interventions (EB Table A.3.1)

Evidence was derived from an updated Cochrane systematic review on interventions for preventing mastitis after childbirth *(50)*, which includes 10 trials with 3034 women. This review included both pharmacological and non-pharmacological interventions, which are each addressed separately for the purposes of this guideline. The current summary includes six trials (2215 women) evaluating non-pharmacological interventions, which were conducted in Australia (1 trial), Brazil (1), China (2), Spain (1) and Sweden (1), and published between 2004 and 2018.

Two trials (of which only one provided data) compared probiotics with placebo. The other three trials evaluated hydrothermally processed cereals, in-hospital specialist breastfeeding education and breast acupoint massage.

All the trials included women who did not have mastitis at enrolment.

Comparison 1: Probiotics compared with placebo

Probiotics were given daily in the form of capsules containing *Lactobacillus fermentum 3* in one trial (625 women). Results of the largest trial conducted in Australia (639 women) are unavailable due to a contractual agreement between the probiotics supplier and the trialists.

Maternal outcomes

Prevention of symptoms: It is uncertain whether probiotics reduce the number of women with nipple damage within six months postpartum, or breast pain (very low-certainty evidence).

Short-term maternal morbidity: Low-certainty evidence suggests probiotics may make little or no difference to the risk of mastitis when compared with placebo (1 trial, 291 women; RR 0.58, 95% CI 0.33 to 1.02).

Maternal functioning/well-being and *adverse effects* were not reported in the included trials. *Health service use* and *experience of postnatal care* was not reported in the systematic review.

Newborn/infant outcomes

Breastfeeding status was not reported in the included trials and *adverse effects* were not reported in the systematic review.

Additional considerations

The largest trial evaluating probiotics (639 women), which compared probiotics with placebo, was the trial for which no data were available due to restrictions imposed on the trial authors by the intervention manufacturer. With only one other trial included in this comparison, it is likely that the inclusion of these data would substantially impact the overall results.

Another Cochrane systematic review on the effectiveness and safety of treatments for breast engorgement during lactation – including 21 trials (2170 women) – was updated in 2020 *(49)*. While mastitis was a pre-specified outcome for the review, trials did not include mastitis as an outcome.

Values

See Box 3.4 in section 3.A.3: Preventive measures.

In addition, findings from a qualitative evidence synthesis exploring what women want from postnatal care *(21)* highlight the importance women place on breastfeeding as a medium for establishing a relationship with their baby (moderate confidence in the evidence) and the unanticipated challenges they sometimes experience when breastfeeding is difficult or painful (moderate confidence in the evidence). Findings also suggest that women would welcome any additional support, information and, where appropriate, treatment (pharmacological or non-pharmacological) to facilitate successful breastfeeding (high confidence in the evidence).

Additional considerations

Health workers would generally place high value on promoting breastfeeding and on interventions that may help them to provide better advice and support to breastfeeding women.

Resources

No economic evaluations of non-pharmacological interventions for preventing mastitis following childbirth were identified.

Equity

No direct evidence was identified on the impact on health equity of non-pharmacological interventions for preventing mastitis following childbirth. Probiotics may be prohibitively expensive for some women and may decrease equity. Cold storage is required for some probiotics, which may not be available in under-resourced settings. It is unlikely probiotics will be supplied by a health service. However, if probiotics can be supplied by a health service, they may have no effect on or may increase equity.

Additional considerations

Even though the health, emotional, psychosocial and societal benefits to women and children of breastfeeding are recognized, breastfeeding rates worldwide are suboptimal, especially among low-income women. Increasing breastfeeding initiation and duration among low-income women, including

Table 3.35 Main resource requirements for probiotics

Resource	Description
**Staff**	• Doctors/midwives/nurses, or else none required
**Training**	• Practice-based training for health workers, or else none required
**Supplies**	• Probiotics sachets or tablets/capsules (approximately US$ 1 per tablet/capsule or US$ 1.50 per sachet, intended for daily use)
**Equipment and infrastructure**	• Some probiotics may require refrigeration or must be stored below a certain temperature (e.g. below 25 °C)
**Time**	• Intended for daily use
**Supervision and monitoring**	• Same as for usual care

prevention of breast problems that may affect breastfeeding continuation, would not only offer improved health benefits to the mother and infant, but would lessen the economic burden experienced by this group within the community *(65, 66)*.

Acceptability

A qualitative evidence synthesis of women's experiences of postnatal care found no direct evidence relating to women's views on non-pharmacological treatments for preventing mastitis *(28)*. However, indirect evidence from this review suggests that women often feel unprepared for the potential challenges associated with breastfeeding (moderate confidence in the evidence) and are likely to welcome more information and support (including instruction from appropriately trained staff) to enable informed decision-making with regard to breastfeeding techniques and/or possible treatments for painful or uncomfortable breasts (high confidence in the evidence). Women would appreciate any techniques or treatments that provide relief from breast engorgement (high confidence in the evidence) as they are likely to enhance the development of the mother–infant relationship, improve self-perception of body image and increase psychosocial well-being (high confidence). Findings from the same review also indicate that, in some contexts, women may prefer to use traditional practices including diet, medicinal plants, massage and spiritual healing to enhance breastfeeding and treat any associated problems (moderate confidence in the evidence).

Additional considerations

It is anticipated that probiotics as sachets, tablets or capsules would be an acceptable intervention to most women.

Feasibility

A qualitative evidence synthesis of women's experiences of postnatal care found no direct evidence relating to women's views on the feasibility of using non-pharmacological interventions to prevent mastitis *(28)*. Indirect evidence from the same review indicates that some women in LMICs may be less likely to seek help for this type of problem if they perceive that health facilities lack the resources (that is, appropriately trained staff or suitable treatments) or if they believe that the preventive strategy will incur additional costs (moderate confidence in the evidence).

A qualitative evidence synthesis of health workers' experiences of postnatal care found no direct evidence relating to views on the feasibility of interventions to treat breast engorgement during lactation *(29)*. However, indirect evidence suggest that lack of personnel, resources and training may limit the offer of non-pharmacological interventions, provision of information and counselling on interventions to prevent mastitis in the postnatal period (moderate confidence in the evidence). The lack of continuity of care and absence of common policies or guidelines across different cadres and levels of maternal health services may limit the offer of consistent information and breastfeeding counselling (moderate confidence in the evidence).

Additional considerations

Probiotics may be prohibitively expensive for some women. Some probiotics may require refrigeration or must be stored below a certain temperature (e.g. below 25 °C), which may not be feasible in some settings. The shelf-life of probiotics must also be considered.

Table 3.36 Summary of judgements: Probiotics compared with placebo

Domain	Judgement
Desirable effects	Trivial
Undesirable effects	Don't know
Certainty of the evidence	Very low
Values	Probably no important uncertainty or variability
Balance of effects	Don't know
Resources required	Moderate costs
Certainty of the evidence on required resources	No included studies
Cost-effectiveness	Don't know
Equity	Varies
Acceptability	Probably yes
Feasibility	Varies

Comparison 2: Hydrothermally processed cereal with anti-secretory factor-inducing properties compared with standard cereal (serving as a placebo)

The trial (40 women) contributing to this comparison considered hydrothermally processed cereals (which induce the production of anti-secretory factor [AF] in human milk) versus non-treated cereal (as a placebo).

Maternal outcomes

Short-term maternal morbidity: It is uncertain whether hydrothermally processed cereal reduces the incidence of mastitis within six months postpartum, or within 12 months postpartum, when compared with standard cereal (very low-certainty evidence).

Maternal functioning/well-being was not reported in the included trial. Prevention of symptoms, health service use, experience of postnatal care, and adverse effects were not reported in the systematic review.

Newborn/infant outcomes

Breastfeeding status was not reported in the included trial and *adverse effects* were not reported in the systematic review.

Values

Evidence around values is the same as for *Comparison 1: Probiotics compared with placebo*.

Additional considerations

Additional considerations around the values of health workers are the same as for *Comparison 1: Probiotics compared with placebo*.

Resources

No economic evaluations of non-pharmacological interventions for preventing mastitis following childbirth were identified.

Equity

No direct evidence was identified on the impact on health equity of non-pharmacological interventions for preventing mastitis following childbirth. The impact on equity of AF-inducing foods is likely to vary by their regional availability and cost. However, AF-inducing foods may decrease equity as they are unlikely to be supplied by health facilities and may be prohibitively expensive for many women.

Other considerations around equity are the same as for *Comparison 1: Probiotics compared with placebo*.

Table 3.37 Main resource requirements for hydrothermally processed cereal with AF-inducing properties

Resource	Description
**Staff**	• None required
**Training**	• None required
**Supplies**	• AF-inducing foods (e.g. treated cereal) = approximately US$ 22.00 per 450 g packet (to be consumed with dairy products such as yoghurt or milk, cooked as porridge, or used in baking)
**Equipment and infrastructure**	• Dry and cool storage of products<br>• Kitchen facilities and utensils<br>• Other facilities based on chosen preparation method (e.g. refrigeration, cooking facilities)
**Time**	• As daily consumption
**Supervision and monitoring**	• Same as for usual care

AF: anti-secretory factor

Additional considerations

Additional considerations around equity are the same as for *Comparison 1: Probiotics compared with placebo*.

Acceptability

Evidence around acceptability is the same as for *Comparison 1: Probiotics compared with placebo*.

Additional considerations

It is anticipated that AF-inducing foods such as treated cereals would be an acceptable intervention for most women.

Feasibility

Evidence around feasibility is the same as for *Comparison 1: Probiotics compared with placebo*.

Additional considerations

AF-inducing foods such as treated cereals may be prohibitively expensive for many women. Their regional availability is likely to vary.

Table 3.38 Summary of judgements: Hydrothermally processed cereal with AF-inducing properties compared with standard cereal (serving as a placebo)

Domain	Judgement
Desirable effects	Don't know
Undesirable effects	Don't know
Certainty of the evidence	Very low
Values	Probably no important uncertainty or variability
Balance of effects	Don't know
Resources required	Moderate costs
Certainty of the evidence on required resources	No included studies
Cost-effectiveness	Don't know
Equity	Probably reduced
Acceptability	Probably yes
Feasibility	Varies

Comparison 3: Specialist breastfeeding education compared with usual care

The trial (211 women) contributing to this comparison considered in-hospital specialist breastfeeding education (a 30-minute personal session with a lactation consultant and a nurse) versus usual care (early breastfeeding, advice on breastfeeding techniques and support in case of difficult breastfeeding).

Maternal outcomes

Prevention of symptoms: It is uncertain whether specialist breastfeeding education reduces the risk of breast pain (defined in the trial as sore nipples) at hospital discharge, at 7 days and at 30 days, when compared with usual care (very low-certainty evidence). It is uncertain whether specialist breastfeeding education reduces the risk of breast engorgement at any of these time points when compared with usual care (very low-certainty evidence).

Short-term maternal morbidity: It is uncertain whether specialist breastfeeding education reduces the risk of women developing mastitis at hospital discharge, at 7 days and at 30 days, when compared with usual care (very low-certainty evidence).

Maternal functioning/well-being was not reported in the included trial and *health service use, experience of postnatal care*, and *adverse effects* were not reported in the systematic review.

Newborn/infant outcomes

Breastfeeding status: Low-certainty evidence suggests specialist breastfeeding education may make little or no difference to exclusive breastfeeding at seven days (1 trial, 169 women; RR 1.03, 95% CI 0.90 to 1.18). It is uncertain whether specialist breastfeeding education affects exclusive breastfeeding at 30 days (very low-certainty evidence).

Adverse effects were not reported in the systematic review.

Additional considerations

For the purpose of the comparisons in this evidence summary, early breastfeeding and breastfeeding advice and/or support (not including specialist advice) was deemed usual care. Such advice should be the minimum standard for quality postnatal care in all facilities.

Another Cochrane systematic review of 21 trials (2170 women) on the effectiveness and safety of treatments for breast engorgement during lactation was updated in 2020 *(49)*. While mastitis was a pre-specified outcome for the review, trials did not include mastitis as an outcome.

Values

Evidence around values is the same as for the previous comparisons.

Additional considerations

Qualitative evidence on breastfeeding counselling has shown that both women and health workers highly value breastfeeding counselling *(76)*. It has also shown that women wanted more counselling and stressed the importance of follow-up. When provided or proactively sought out by women, counselling is highly valued and increased satisfaction.

Resources

No economic evaluations of non-pharmacological interventions for preventing mastitis following childbirth were identified.

Additional considerations

The cost of lactation specialists could be relatively high, depending on location and setting. Where midwives and nurses can gain the necessary training to provide specialist lactation support, costs may be lowered. However, the cost of training itself may also be considerable.

Equity

No direct evidence was identified on the impact on health equity of non-pharmacological interventions for preventing mastitis following childbirth. Specialist breastfeeding advice and support may decrease equity, as it can be difficult for women to access due to limited service availability and potential out-of-pocket costs, in particular in settings where the service is only available from specialist personnel such as lactation consultants. Where specialist breastfeeding education can be performed by midwives and nurses with lactation training, it may have no effect on or may increase equity.

Additional considerations

Additional considerations around equity are the same as for the previous comparisons.

Acceptability

Evidence around acceptability is the same as for the previous comparisons.

Additional considerations

Another systematic review of qualitative studies on breastfeeding counselling found that breastfeeding counselling was highly valued by women and health workers. It was acknowledged that health workers may be reticent to counsel if not properly trained and allocated sufficient time for counselling *(76)*. Variability in acceptability was judged as minor.

Table 3.39 Main resource requirements for specialist breastfeeding education

Resource	Description
**Staff**	• Varies depending on the specific programme; lactation consultant, breastfeeding nurse, midwife or other specialist provider
**Training**	• Certification in lactation
**Supplies**	• Information (written and/or pictorial, e.g. leaflets)
**Equipment and infrastructure**	• Calm, safe room conducive to privacy (curtain, door, wall)<br>• Ability to conduct home visits where needed
**Time**	• *Time to train:* varies depending on specific programme<br>• *Time to perform:* varies depending on specific programme; ideally over multiple sessions of at least 30 minutes duration
**Supervision and monitoring**	• Same as for usual care

Feasibility

Evidence around feasibility is the same as for the previous comparisons.

Additional considerations

To enable quality counselling, the provision of appropriate training, coaching, and support for health workers and lay/non-lay counsellors by skilled trainers is essential. Trained counsellors need sufficient time for counselling. Health workers would prefer to have more time and resources, in order to provide better quality counselling *(76)*. The feasibility of specialist breastfeeding education is therefore likely to vary based on access to personnel, resources and training.

Table 3.40 Summary of judgements: Specialist breastfeeding education compared with usual care

Domain	Judgement
Desirable effects	Trivial
Undesirable effects	Don't know
Certainty of the evidence	Very low
Values	Probably no important uncertainty or variability
Balance of effects	Don't know
Resources required	Moderate costs
Certainty of the evidence on required resources	No included studies
Cost-effectiveness	Don't know
Equity	Varies
Acceptability	Probably yes
Feasibility	Varies

Comparison 4: Acupoint massage compared with usual care

The trial (400 women) contributing to this comparison considered breast acupoint massage[35] with usual care (early breastfeeding, breastfeeding advice on positioning and attachment and breastfeeding on-demand).

35 Digital pressure massage from proximal to distal, along the direction of the breast ducts, followed by massage around the 'root' of the breast in a clockwise direction, for 30 seconds, three times per day.

Maternal outcomes

Prevention of symptoms: Moderate-certainty evidence suggests acupoint massage probably reduces the risk of breast pain when compared with usual care (1 trial, 400 women; RR 0.13, 95% CI 0.07 to 0.23). Moderate-certainty evidence suggests acupoint massage probably reduces the risk of breast engorgement when compared with usual care (1 trial, 400 women; RR 0.49, 95% CI 0.37 to 0.65).

Short-term maternal morbidity: Moderate-certainty evidence suggests acupoint massage probably reduces the incidence of mastitis within six months postpartum when compared with usual care (1 trial, 400 women; RR 0.38, 95% CI 0.19 to 0.78).

Maternal functioning/well-being: Moderate-certainty evidence suggests acupoint massage probably improves women's perception of milk supply (moderate or better) when compared with usual care (1 trial, 400 women; RR 1.26, 95% CI 1.13 to 1.40).

Health service use, experience of postnatal care, and *adverse effects* were not reported in the systematic review.

Newborn/infant outcomes

Breastfeeding status: Moderate-certainty evidence suggests that acupoint massage probably increases exclusive breastfeeding (at 42 days postpartum) when compared with usual care (1 trial, 400 women; RR 1.90, 95% CI 1.58 to 2.29).

Adverse effects were not reported in the systematic review.

Values

Evidence around values is the same as for the previous comparisons.

Resources

No economic evaluations of non-pharmacological interventions for preventing mastitis following childbirth were identified.

Additional considerations

The cost of acupoint massage provided by professional massage therapists could be relatively high, depending on location and setting.

Equity

No direct evidence was identified on the impact on health equity of non-pharmacological interventions for preventing mastitis following childbirth. Acupoint

Table 3.41 Main resource requirements for acupoint massage

Resource	Description
**Staff**	• Midwife, nurse or massage therapist (who is permitted to perform breast massage)
**Training**	• Training in postnatal acupoint breast massage (for midwives or nurses) or else certification in massage therapy with licence to offer breast massage
**Supplies**	• Information (written and/or pictorial, e.g. leaflets) (where self-administered)<br>• Warmed towels and natural massage lubricant
**Equipment and infrastructure**	• Calm, safe room conducive to privacy (curtain, door, wall)<br>• Ability to conduct home visits where needed<br>• Heating facilities required to warm towels<br>• Chair, bed or massage table
**Time**	• Varies depending on the specific method<br>• Acupoints are pressed for short durations (e.g. 30 seconds) multiple times daily, with additional acupoint massage if breast is painful
**Supervision and monitoring**	• Same as for usual care

breast massage may decrease equity, as it can be difficult for women to access due to limited service availability and potential out-of-pocket costs, in particular if the service is provided by specialist personnel. Where acupoint breast massage can be performed by midwives or other health personnel, or self-administered with instruction, it may have no effect on or may increase equity.

Additional considerations

Additional considerations around equity are the same as for the previous comparisons.

Acceptability

Evidence around acceptability is the same as for the previous comparisons.

Additional considerations

It is anticipated that acupoint massage would be acceptable for most women. Although pressure is applied to the breast, the amount of pressure applied should never induce pain.

Feasibility

Evidence around feasibility is the same as for the previous comparisons.

Additional considerations

Accupoint breast massage may be feasible in settings where midwives or other health personnel can access the necessary training and provide it, but this will vary across health facilities. Accupoint massage may also be self-administered following instructions from trained personnel.

Table 3.42 Summary of judgements: Acupoint massage compared with usual care

Domain	Judgement
Desirable effects	Moderate
Undesirable effects	Don't know
Certainty of the evidence	Moderate
Values	Probably no important uncertainty or variability
Balance of effects	Probably favours acupoint massage
Resources required	Varies
Certainty of the evidence on required resources	No included studies
Cost-effectiveness	Don't know
Equity	Varies
Acceptability	Probably yes
Feasibility	Varies

A.3.2 Pharmacological interventions to prevent postpartum mastitis

RECOMMENDATION 11

Routine oral or topical antibiotic prophylaxis for the prevention of mastitis in the postpartum period is not recommended. *(Not recommended)*

Remarks

- In making this recommendation, the Guideline Development Group (GDG) emphasized the risk of adverse effects of antibiotics for the woman and the newborn, and the negative public health impact of routine antibiotic administration on the global efforts to contain antimicrobial resistance.
- The GDG agreed that further investigation on the effects of antibiotics for the prevention of mastitis is not a research priority.

Summary of evidence and considerations

Effects of the interventions (EB Table A.3.2)

Evidence was derived from a Cochrane systematic review on interventions for preventing mastitis after childbirth, which includes 10 trials with 3034 women *(50)*. Three trials compared the use of antibiotics versus placebo, a different antibiotic, or usual care. One of these trials (Sebitloane et al., 2008) was not considered as neither the intervention (antibiotics given during the intrapartum period) nor the population (women with HIV planning a vaginal birth) were in the scope of this guideline.

Only the comparisons evaluating antibiotics versus placebo or no intervention, including usual care – and not those comparing antibiotics with other antibiotics – have been extracted for this evidence summary. Only one priority outcome, the incidence of mastitis within six months postpartum, was reported in the two included trials.

Two comparisons are presented below: *(1) Oral prophylactic antibiotics compared with placebo or usual care, and (2) Topical prophylactic antibiotics versus placebo or no intervention*. The evidence and judgements related to the effects of interventions (desirable effects, undesirable effects, and certainty of the evidence) are presented separately for each comparison. The remaining domains (values, resources, equity, acceptability and feasibility) were considered to be similar across both interventions.

Comparison 1: Oral prophylactic antibiotics compared with placebo or usual care

Comparison 1a: Oral antibiotics (flucloxacillin) compared with placebo

This comparison includes one trial (10 women), conducted in Australia and published in 2004, which included lactating women with cracked nipples colonized with *Staphylococcus aureus.* The trial compared oral flucloxacillin (taken for seven days) versus placebo capsules for the same duration. The trial was interrupted early due to poor intervention compliance and lack of eligible participants.

Maternal outcomes

Short-term maternal morbidity: Low-certainty evidence suggests that the use of oral antibiotics (flucloxacillin) may make little or no difference to the risk of mastitis within six months postpartum when compared with placebo (1 trial, 10 women; RR 0.33, 95% CI 0.02 to 6.55).

Maternal functioning/well-being and *adverse effects* were not reported in the included trial. Health service use and experience of postnatal care were not reported in the systematic review.

Newborn/infant outcomes

Breastfeeding status was not reported in the included trial, and *adverse effects* were not reported in the systematic review.

Comparison 1b: Oral antibiotics (cloxacillin/erythromycin) compared with usual care (breastfeeding advice)

This comparison includes data from a four-arm trial (84 women) conducted in Canada and published in 1999, which included women attending a breastfeeding clinic for breastfeeding problems, cracked/sore nipples, or positive *S. aureus* results. The four arms of the trial compared: topical 2% mupirocin ointment applied to the nipples (n = 25 women); topical fusidic acid ointment applied to the nipples (n = 17); and oral antibiotics – cloxacillin/Erythromycin (regimen not reported) (n = 19) with breastfeeding advice (n = 23). The trial was interrupted early because trial authors perceived that women who did not receive antibiotic had a higher rate of mastitis (no further information provided). The oral antibiotics versus breastfeeding advice arms of this trial are considered in this comparison.

Maternal outcomes
Short-term maternal morbidity: Low-certainty evidence suggest that the use of oral antibiotics (cloxacillin/erythromycin) may make little or no difference to the risk of mastitis within six months postpartum when compared with usual care (1 trial, 42 women; RR 0.17, 95% CI 0.02 to 1.28).

Maternal functioning/well-being and *adverse effects* were not reported in the included trial. Health service use and experience of postnatal care were not reported in the systematic review.

Newborn/infant outcomes
Breastfeeding status was not reported in the included trial, and *adverse effects* were not reported in the systematic review.

Comparison 2: Topical prophylactic antibiotics compared with usual care (breastfeeding advice)

This comparison includes data from arm three of the four-arm trial (84 women) described above.

Maternal outcomes
Short-term maternal morbidity: Low-certainty evidence suggests that topical fusidic acid ointment (1 trial, 40 women; RR 0.77, 95% CI 0.27 to 2.22) may make little or no difference to the incidence of mastitis within six months postpartum when compared with usual care. Low-certainty evidence suggests that topical mupirocin ointment (1 trial, 48 women; RR 0.39, 95% CI 0.12 to 1.35) may make little or no difference to the incidence of mastitis within six months postpartum when compared with usual care.

Maternal functioning/well-being and *adverse effects* were not reported in the included trial. *Health service use* and *experience of postnatal care* were not reported in the systematic review.

Newborn/infant outcomes
Breastfeeding status was not reported in the included trial and *adverse effects* were not reported in the systematic review.

Additional considerations
Another Cochrane systematic review on the effectiveness and safety of treatments for breast engorgement during lactation including 21 trials (2170 women) was updated in 2020 *(49)*. While mastitis was a pre-specified outcome for the review, trials did not include mastitis as an outcome.

A 2013 Cochrane systematic review on the effectiveness of antibiotic therapies for relieving symptoms for breastfeeding women with mastitis with or without laboratory investigation found insufficient evidence to confirm or refute the effectiveness of antibiotic therapy for the treatment of lactational mastitis *(77)*.

Values
See Box 3.4 in section 3.A.3: Preventive measures.

In addition, findings from a qualitative evidence synthesis exploring what women want from postnatal care *(21)* highlights the importance some women place on breastfeeding as a medium for establishing a relationship with their baby (moderate confidence in the evidence) and the unanticipated challenges they sometimes experience when breastfeeding is difficult or painful (moderate confidence in the evidence). Findings further suggest that women may welcome any additional support, information and, where appropriate, treatment to facilitate successful breastfeeding (high confidence in the evidence).

Additional considerations
Most women may prefer to avoid the inconvenience and adverse effects of antibiotic use.

Table 3.43 Main resource requirements for pharmacological interventions to prevent mastitis

Resource	Description
**Staff**	• Doctors/midwives/nurses
**Training**	• Practice-based training for health workers
**Supplies**	• Oral antibiotics,[36] price per tablet/capsule *(57)*:<br>– amoxicillin/clavulanic acid 500 mg/125 mg = US$ 0.16<br>– cephalexin 250 mg = US$ 0.04<br>– cloxacillin sodium 500 mg = US$ 0.04<br>• Topical antibiotics, price per gram *(57)*:<br>– fusidic acid 2% cream = US$ 0.08<br>– mupirocin 2% cream = US$ 0.19
**Equipment and infrastructure**	• On-site pharmacy and/or medicine stock management system that is managed by a trained pharmacist or dispenser
**Time**	• Dispensing time estimated to be 2–5 minutes
**Supervision and monitoring**	• Same as for usual care

Health workers and policy-makers are likely to place a high value on the potential impact of antibiotic use on antibiotic resistance. There may be no variation in this value across settings.

Resources

No economic evaluations of pharmacological interventions for preventing mastitis following childbirth were identified.

Equity

No direct evidence was identified on the impact on health equity of pharmacological interventions for preventing mastitis following childbirth. Prophylactic antibiotics for preventing mastitis following childbirth may increase equity, as these medications are widely available at low cost. However, prophylactic antibiotics may decrease equity if women are expected to pay for the antibiotics themselves.

Additional considerations

Even though the recognized health, emotional, psychosocial and societal benefits of breastfeeding to women and children, breastfeeding rates worldwide are sub-optimal, especially among low-income women. Increasing breastfeeding initiation and duration among low-income women, including prevention of breast problems that may affect breastfeeding continuation, would not only offer improved health benefits to the mother and infant, but would lessen the economic burden experienced by this group within the community *(65, 66)*.

Acceptability

A qualitative evidence synthesis of women's experiences of postnatal care found no direct evidence relating to women's views on pharmacological treatments for preventing mastitis *(28)*. However, indirect evidence from this review suggests that women often feel unprepared for the potential challenges associated with breastfeeding (moderate confidence in the evidence) and are likely to welcome more information and support to enable informed decision-making with regard to breastfeeding techniques and/or possible treatments for painful or uncomfortable breasts (high confidence in the evidence). Findings from the same review also indicate that women would appreciate any techniques or treatments that provide relief from breast engorgement (high confidence in the evidence) as they are likely to enhance the development of the mother–infant relationship, improve self-perception of body image and increase psychosocial well-being (high confidence in the evidence). In some contexts, women may prefer to use traditional practices (diet, medicinal plants and spiritual healing) to enhance breastfeeding practices and treat any problems associated with breastfeeding (moderate confidence in the evidence).

36 Includes the antibiotics listed under the WHO Model List of Essential Medicines "access group antibiotics" (those that have wide activity against pathogens and lower resistance potential), which are suitable for skin and soft tissue infections.

Additional considerations

One trial in the Cochrane systematic review was ceased prematurely due to difficulties in recruitment arising from the demands of motherhood and reluctance to take antibiotics among some women *(50)*.

Given concerns about adverse effects and antibiotic resistance, it is possible that many women and health workers will be reluctant to take/prescribe antibiotics in the absence of a confirmed infection, and without clear evidence of benefit.

Some breastfeeding women may be reluctant to apply a cream directly to the breast, if they are concerned about their baby ingesting the cream while feeding and/or if they have to remove the cream before breastfeeding.

Feasibility

A qualitative evidence synthesis of women's experiences of postnatal care found no direct evidence relating to women's views on the feasibility of using prophylactic antibiotics to treat mastitis *(28)*. Indirect evidence from the same review indicates that some women in LMICs may be less likely to seek help for this type of problem if they perceive that health facilities lack the resources to offer appropriate treatments or if they believe the preventive strategy will incur additional costs (moderate confidence in the evidence).

Additional considerations

The antibiotics listed under the WHO Model List of Essential Medicines "access group antibiotics" (those that have wide activity against pathogens and lower resistance potential) that are suitable for skin and soft tissue infections are amoxicillin plus clavulanic acid (as a first-choice treatment), cloxacillin (as a first-choice treatment) and cephalexin (as a second-choice treatment) *(58)*. Oral administration is preferred for cloxacillin due to better bioavailability *(58)*.

Table 3.44 Summary of judgements: Oral prophylactic antibiotics compared with placebo or usual care

Domain	Judgement
Desirable effects	Trivial
Undesirable effects	Don't know
Certainty of the evidence	Low
Values	Probably no important uncertainty or variability
Balance of effects	Probably favours no intervention
Resources required	Negligible costs or savings
Certainty of the evidence on required resources	No included studies
Cost-effectiveness	Don't know
Equity	Varies
Acceptability	Varies
Feasibility	Varies

Table 3.45 Summary of judgements: Topical prophylactic antibiotics versus usual care (breastfeeding advice)

Domain	Judgement
Desirable effects	Trivial
Undesirable effects	Don't know
Certainty of the evidence	Low
Values	Probably no important uncertainty or variability
Balance of effects	Probably favours no intervention
Resources required	Negligible costs or savings
Certainty of the evidence on required resources	No included studies
Cost-effectiveness	Don't know
Equity	Varies
Acceptability	Varies
Feasibility	Varies

A.3.3 Prevention of postpartum constipation

RECOMMENDATION 12

Dietary advice and information on factors associated with constipation should be offered to women for the prevention of postpartum constipation. *(Recommended)*

Remarks

- Dietary advice and information to prevent constipation during the postnatal period should include promoting a healthy balanced diet with adequate intake of water and dietary fibre (found in vegetables, fruit, nuts and whole grains) *(78)*. Information should include factors related to constipation, as well as advice on toileting habits (e.g. responding to the urge to have a bowel movement, and complete evacuation) and engaging in low impact, physical activity (e.g. walking) for at least 150 minutes throughout the week (see Recommendation 22 in this guideline). Advice and information should be culturally sensitive, and tailored to a woman's needs (e.g. considering mode of birth or birth complications) and to specific contexts.
- Constipation during the postpartum period could potentially result from a range of antepartum, intrapartum and postpartum-related events and circumstances, including haematinics used in pregnancy and postpartum, disrupted drinking and eating during active labour, enemas, narcotic drugs administered during labour or post caesarean birth, and perineal pain related to trauma, haemorrhoids, irregular and altered dietary patterns in the postnatal period, and psychosocial and situational factors. Prevention of constipation should include measures to address these common underlying factors.
- The Guideline Development Group (GDG) recognized the need to ensure that health workers adhere to existing WHO recommendations as part of the strategies to prevent postpartum constipation (2014 *WHO recommendations for augmentation of labour [79]* and 2018 *WHO recommendations on intrapartum care for a positive childbirth experience [17]*).
  - For women at low risk, WHO recommends oral fluid and food intake and the adoption of mobility during labour.
  - Administration of an enema for reducing the use of labour augmentation is not recommended.
- All women should be asked about their bowel movements during their postpartum stay in health facilities, and at each postnatal care contact.
- In making this recommendation, the GDG took into account a stepwise approach for the prevention and treatment of constipation in the adult population, where the use of laxatives is applied only if dietary modifications or fibre supplementation fail to relieve the constipation, particularly given concerns about maternal and neonatal adverse effects of laxatives. The GDG suggested that this approach be applied in the immediate postpartum to stimulate first maternal bowel movements after childbirth and through the entire postnatal period, after both vaginal and caesarean birth.
- Women with a history of constipation before or during pregnancy may benefit from continuing with treatments to relieve postpartum constipation.

RECOMMENDATION 13

Routine use of laxatives for the prevention of postpartum constipation is not recommended. *(Not recommended)*

Remarks

- The Guideline Development Group (GDG) highlighted that the current recommendation is applicable in the context of prevention of functional postpartum constipation, defined as infrequent, hard, dry or bulky stools that are difficult or painful to pass, or a feeling of incomplete evacuation or obstruction. This recommendation does not apply to chronic constipation and acute constipation associated with other organ dysfunctions (i.e. acute gastrointestinal dysfunction).
- In making this recommendation, the GDG considered a stepwise approach for the prevention and treatment of constipation in the adult population, where the use of laxatives is only applied if dietary modifications or fibre supplementation fail to relieve the constipation. The GDG suggested that this approach be applied in the immediate postpartum to stimulate first maternal bowel movements after childbirth and through the entire postnatal period, after both vaginal and caesarean birth.
- All women should be asked about their bowel movements during their postpartum stay in health facilities, and at each postnatal care contact, and should receive dietary advice and information on factors associated with constipation as per Recommendation 12 in this guideline.
- The GDG agreed that further investigation on the effects of routine use of laxatives for preventing constipation in postpartum women is not a research priority.

Summary of evidence and considerations

Effects of the interventions (EB Table A.3.3)

Evidence was derived from an updated Cochrane systematic review of interventions for preventing postpartum constipation *(74)*. Data for this evidence summary were derived from four trials involving 1061 women, of which two were RCTs and two were quasi-RCTs. Trial were conducted in Ireland (1), South Africa (1) and the USA (2). All four trials were published 40 or more years ago. A fifth trial comparing laxative plus a bulking agent versus laxative alone in women who had undergone surgical repair following anal sphincter injuries during vaginal birth was not considered in this evidence summary.

All trials compared use of laxatives versus no intervention or placebo. The laxatives evaluated were bisoxatin acetate, active senna, dorbanex, and dioctyl-sodium succinate plus senna. bisoxatin acetate is now contraindicated for breastfeeding women, while dorbanex is no longer available as it is "reasonably anticipated to be human carcinogen" based on animal studies *(74)*. Therefore, only data from the trials including available treatments and treatments indicated in postpartum women are described below (two trials). In both of the included trials, laxative treatment was commenced immediately after birth.

Comparison: Laxatives compared with placebo

Two eligible trials (755 women) examined the effectiveness and safety of a laxative versus placebo. These trials were published in 1960 and 1980, and compared active senna or dioctyl-sodium succinate plus active senna versus placebo.

Maternal outcomes

Symptoms of constipation – time to first bowel movements (days): Low-certainty evidence suggests laxatives may result in more women having their first bowel movement less than 24 hours after birth when compared with placebo (1 trial, 471 women; RR 2.90, 95% CI 2.24 to 3.75). It is uncertain whether laxatives affect the number of women having their first bowel movement on day 1 after birth (very low-certainty evidence). Low-certainty evidence suggests laxatives may result in fewer women having their first bowel movement on day 2 after birth (1 trial, 471 women; RR 0.23, 95% CI 0.11 to 0.45). It is uncertain whether laxatives affect the number of women having their first bowel movement on day 3 or on day 4 after birth (both very low-certainty evidence). None of the trials reported on other symptoms of constipation, such as pain or straining during defecation, incidence of postpartum constipation as per self-report or stool consistency.

Health service use: It is uncertain whether laxatives affect the number of postpartum enemas given (very low-certainty evidence).

Adverse effects: It is uncertain whether laxatives affect the incidence of abdominal cramps (very low-certainty evidence).

Maternal functioning/well-being was not reported in the included trials and *experience of postnatal care* was not reported in the systematic review.

Newborn/infant outcomes
Adverse effects: It is uncertain whether laxatives affect the incidence of neonatal loose stools and diarrhoea (very low-certainty evidence).

Additional considerations

None of the trials assessed non-pharmacological interventions, such as dietary advice and modification, promotion of healthy physical activities, correct bodily positioning for defecation, use of herbs, or traditional and complementary medicine.

There is some concern around the use of dioctyl-sodium succinate, which was evaluated together with senna in one of the eligible trials. The drug is reported to be ineffective and potentially unsuitable for use during breastfeeding *(74)*. Some laxatives may interfere with the body's absorption of vitamins and mineral supplements *(16)*.

Values

See Box 3.4 in section 3.A.3: Preventive measures.

Resources

No economic evaluations of pharmacological and non-pharmacological interventions for preventing postpartum constipation were identified.

Equity

No direct evidence was identified on the impact on health equity of pharmacological and non-pharmacological interventions for preventing postpartum constipation. The impact of pharmacological and non-pharmacological interventions for preventing postpartum constipation on health equity is likely to vary across interventions. As pharmacological interventions and dietary supplements are widely available at relatively low cost and without a prescription, these may increase equity. Non-pharmacological interventions such as broader dietary modification and physical activity may present challenges for some women.

Acceptability

A qualitative evidence synthesis of women's experiences of postnatal care found no direct evidence relating to women's views on pharmacological or non-pharmacological treatments for preventing/relieving postpartum constipation *(28)*. Indirect evidence from this review suggests that women may appreciate any techniques or treatments that provide relief from

Table 3.46 Main resource requirements for prevention of postpartum constipation

Resource	Description
**Staff**	• Varies depending on intervention; some require no staff<br>• Dietary information and general advice may be provided by a midwife or nutrition and dietetics service
**Training**	• For dietary interventions and general advice, postnatal midwifery training or certification in nutrition and dietetics; or else none required
**Supplies**	• Varies depending on intervention:<br>– senna = US$ 0.01 per tablet/capsule; < US$ 4 for a six-month supply *(57)*<br>– cereal fibre supplements = US$ 1.79 per 227 g bag of wheat bran<br>– herbs, supplements or other alternative treatments (varies by treatment and region)<br>– information (written and/or pictorial, e.g. leaflets)
**Equipment and infrastructure**	• Varies depending on intervention; a stool to assist bodily positioning during defecation may be helpful<br>• Appropriate physical environment, with adequate water, sanitation and hand hygiene and disposal facilities: toilets, washing and bathing facilities that are adequate, safe, clean, well-maintained and conducive to privacy (curtain, door, wall)
**Time**	• Varies depending on the intervention
**Supervision and monitoring**	• Not required

constipation (high confidence in the evidence) as they are likely to aid comfort, mobility and psychosocial well-being (high confidence in the evidence). However, findings from the same review also indicate that, in specific contexts, women may be reluctant to use either pharmacological or non-pharmacological interventions as they adhere to strict dietary routines associated with traditional postnatal practices (low confidence in the evidence).

Additional considerations

Indirect evidence from a qualitative evidence synthesis exploring uptake of antenatal care *(80)* indicates that women in a variety of LMICs are more likely to turn to traditional healers, herbal remedies, or traditional birth attendants to treat constipation (moderate confidence).

Feasibility

A qualitative evidence synthesis of women's experiences of postnatal care found no direct evidence relating to women's views on the feasibility of using pharmacological or non-pharmacological interventions for preventing constipation *(28)*.

A qualitative evidence synthesis of health workers' experiences of postnatal care found no direct evidence relating to views on the feasibility of using pharmacological or non-pharmacological interventions for preventing constipation *(29)*. However, indirect evidence suggests a lack of personnel, resources and training may limit the offer of dietary advice and pharmacological treatments to address this problem (moderate confidence in the evidence).

Additional considerations

The only laxative drug listed in the WHO Model List of Essential Medicines is senna *(58)*, recommended for use only if dietary interventions have been ineffective *(81)*.

It is anticipated that moderate changes to diet would be feasible for most women.

Table 3.47 Summary of judgements: Laxatives compared with placebo

Domain	Judgement
Desirable effects	Don't know
Undesirable effects	Don't know
Certainty of the evidence	Very low
Values	Probably no important uncertainty or variability
Balance of effects	Don't know
Resources required	Varies
Certainty of the evidence on required resources	No included studies
Cost-effectiveness	Don't know
Equity	Varies
Acceptability	Probably yes
Feasibility	Probably yes

Table 3.48 Summary of judgements: Non-pharmacological intervention (diet and lifestyle advice) compared with no intervention

Domain	Judgement
Desirable effects	Don't know
Undesirable effects	Don't know
Certainty of the evidence	No included studies
Values	Probably no important uncertainty or variability
Balance of effects	Probably favours non-pharmacological intervention
Resources required	Varies
Certainty of the evidence on required resources	No included studies
Cost-effectiveness	Don't know
Equity	Varies
Acceptability	Probably yes
Feasibility	Probably yes

A.3.4 Prevention of maternal peripartum infection after uncomplicated vaginal birth

RECOMMENDATION 14

Routine antibiotic prophylaxis for women with uncomplicated vaginal birth is not recommended. *(Not recommended)*

Remarks

- This recommendation has been integrated from the 2015 *WHO recommendations for prevention and treatment of maternal peripartum infections (82),* where it was considered a strong recommendation based on very low-certainty evidence.
- The following remarks were made by the Guideline Development Group (GDG) responsible for the original recommendation.
  - The GDG was concerned about the potential public health implications of the high rate of routine use of antibiotics following vaginal birth without any specific risk factors in some settings. The group places emphasis on the negative impact of such routine use on the global efforts to contain antimicrobial resistance and, therefore, made a strong recommendation against routine antibiotic prophylaxis.
  - In this context, "uncomplicated vaginal birth" refers to vaginal birth in the absence of any specific risk factor for, or clinical signs of, maternal peripartum infection.
  - Careful monitoring of all women after birth is essential to promptly identify any sign of endometritis and institute appropriate antibiotic treatment.
  - Recommendations on antibiotic use for common intrapartum conditions or interventions that often raise concerns about increased risk of infection are available in the original WHO guideline *(82)*.

A.3.5 Preventive anthelminthic treatment

RECOMMENDATION 15

Preventive chemotherapy (deworming), using annual or biannual[a] single-dose albendazole (400 mg) or mebendazole (500 mg), is recommended as a public health intervention for all non-pregnant adolescent girls and women of reproductive age, including postpartum and/or lactating women, living in areas where the baseline prevalence of any soil-transmitted helminth infection is 20% or more among adolescent girls and women of reproductive age, in order to reduce the worm burden of soil-transmitted helminths.
(Context-specific recommendation)

Remarks

- This recommendation has been adapted and integrated from the 2017 WHO guideline *Preventive chemotherapy to control soil-transmitted helminth infections in at-risk population groups (83)*, where it was considered a strong recommendation based on moderate-certainty evidence.
- Although the original recommendation was formulated for non-pregnant adolescent girls and women of reproductive age, it also applies for lactating women as studies reviewed found there is no harm in its use (low concentration in breastmilk was considered unlikely to be harmful for the breastfed infant).
- During the deliberations, the Guideline Development Group (GDG) responsible for the original recommendation took into particular consideration the following evidence that resulted in a strong recommendation:
  - non-pregnant adolescent girls and women of reproductive age benefit significantly from anthelminthic treatment in terms of a reduction in worm burden;
  - the morbidity caused by the different soil-transmitted helminth species in heavily infected individuals is well documented and severe;
  - albendazole and mebendazole are well tolerated among non-pregnant adolescent girls and non-pregnant women, with only minor and transient adverse effects reported;
  - preventive chemotherapy is generally well accepted among women, health workers and policy-makers, though uncertainty exists around the feasibility of providing this intervention among adolescent girls, as existing infrastructure may vary by country and context;
  - logistical difficulties and additional costs of alternative methods to identify and treat infected individuals can be prohibitive; and
  - soil-transmitted helminth-endemic areas with at least 20% soil-transmitted helminth prevalence were considered the priority for large-scale programmes due to the presence of infections of moderate and heavy intensity and, therefore, soil-transmitted helminth-related morbidity.
- The postnatal care GDG agreed that, in endemic areas, preventive anthelminthic treatment could also be provided to pregnant women after the first trimester as part of worm infection reduction programmes, as per the 2017 WHO guideline *Preventive chemotherapy to control soil-transmitted helminth infections in at-risk population groups (83)* and the 2016 *WHO recommendations on antenatal care for a positive pregnancy experience (16)*.

[a] Biannual administration is recommended where the baseline prevalence exceeds 50%.

A.3.6 Preventive schistosomiasis treatment

RECOMMENDATION 16a

In endemic communities with *Schistosoma* spp. prevalence of 10% or higher, WHO recommends annual preventive chemotherapy with praziquantel in a single dose for ≥ 75% up to 100% of pregnant women after the first trimester, and non-pregnant adolescent girls and women of reproductive age, including postpartum and/or lactating women, to control schistosomiasis morbidity and move towards eliminating the disease as a public health problem. *(Context-specific recommendation)*

RECOMMENDATION 16b

In endemic communities with *Schistosoma* spp. prevalence of less than 10%, WHO suggests one of two approaches based on the programmes' objectives and resources: (i) where there has been a programme of regular preventive chemotherapy, continuing preventive chemotherapy at the same or a reduced frequency towards interruption of transmission; and (ii) where there has not been a programme of regular preventive chemotherapy, a clinical approach of test-and-treat, instead of preventive chemotherapy targeting a population. *(Context-specific recommendation)*

Remarks

- These recommendations have been adapted and integrated from the 2022 *WHO guideline on control and elimination of human schistosomiasis (84)*. Recommendation 16a for settings with prevalence above 10% was considered a strong recommendation based on moderate-certainty evidence. Recommendation 16b was considered a conditional recommendation based on low to very low-certainty evidence.
- The source guideline notes that, in endemic communities with *Schistosoma* spp. baseline prevalence of 10% or higher that demonstrate a lack of appropriate response to annual preventive chemotherapy despite adequate coverage (≥ 75%), WHO suggests biannual instead of annual preventive chemotherapy, in coordination with the interventions stated in *Recommendation 3: Conditional recommendation* (very low-certainty evidence).

A.3.7 Oral pre-exposure prophylaxis (PrEP) for HIV prevention

RECOMMENDATION 17

Oral pre-exposure prophylaxis (PrEP) containing tenofovir disoproxil fumarate (TDF) should be started or continued as an additional prevention choice for postpartum and/or lactating women at substantial risk[a] of HIV infection as part of combination HIV prevention approaches. *(Context-specific recommendation)*

Remarks

- This recommendation has been adapted and integrated from the WHO 2016 *Consolidated guidelines on the use of antiretroviral drugs for treating and preventing HIV infection: recommendations for a public health approach, second edition (42)*, where it was considered a strong recommendation based on high-certainty evidence.
- Pregnant and lactating women living in settings where HIV incidence is greater than 3 per 100 person-years, particularly in sub-Saharan Africa, often remain at substantial and increased risk of HIV acquisition during pregnancy and breastfeeding. Biological factors increase susceptibility, and social and behavioural factors may increase exposure to HIV infection.
- The source guideline states that there is no safety-related rationale for disallowing or discontinuing PrEP use during pregnancy and breastfeeding for HIV-negative women who are receiving PrEP and remain at risk of HIV acquisition. The Guideline Development Group responsible for the original recommendation concluded that in such situations the risk of HIV acquisition and accompanying increased risk of mother-to-child HIV transmission outweigh any potential risks of PrEP, including any risks of fetal and infant exposure to TDF in PrEP regimens.
- As countries roll out PrEP to postpartum and/or lactating women, the source guideline notes the need for active surveillance of this population, including for adverse maternal and infant outcomes.

[a] Substantial risk is provisionally defined as HIV incidence greater than 3 per 100 person-years in the absence of PrEP.

A.4 MENTAL HEALTH INTERVENTIONS

Background

The GDG considered the evidence and other relevant information to inform recommendations on the screening and prevention of common mental disorders in the postpartum period (depression and anxiety).

Depression and anxiety during the postpartum period are leading causes of disability in women around the world. Their prevalence is estimated at 13% in HICs and 19.8% in LMICs *(85)*. Despite a high burden of illness, less than 20% of affected women report their symptoms to health workers, probably due to stigma and poor help-seeking practices inherently associated with these disorders *(86)*. In addition, depression and anxiety have a profound impact on the parent-infant relationship, which is the foundation of the future emotional, relational and social development of the child. Different tools, such as the Edinburgh Postnatal Depression Scale (EPDS) and the Patient Health Questionnaire-9 (PHQ-9), have been used to screen for postpartum depression in routine primary health care or for population-based screening at the community level. Post-screening management may involve various treatments and interventions depending on the specific context.

Box 3.5 Values

Findings from a systematic qualitative review exploring what women want from postnatal care *(21)* indicate that some women experience periods of low mood, depression and loneliness during the postnatal period (moderate confidence in the evidence) and struggle with extended periods of tiredness or exhaustion during the transition to motherhood (moderate confidence in the evidence). For first-time mothers in particular, these feelings may be exacerbated by anxieties and insecurities about their new role and their ability to adapt to an idealized perception of a so-called good mother (moderate confidence in the evidence). To cope with these challenges, women appreciate the support they receive from family members, peers and health workers (high confidence in the evidence) and are therefore likely to value interventions that help them to overcome any mental health concerns. Findings from the same review also indicate that women want a positive experience in which they are able to adapt to their new self-identity and develop a sense of confidence and competence as a mother. They also want to adjust to changes in their intimate and family relationships (including their relationship to their baby), navigate ordinary physical and emotional challenges, and experience the dynamic achievement of personal growth as they adjust to their new normal, both as parents and as individuals in their own cultural context.

A.4.1 Screening for postpartum depression and anxiety

RECOMMENDATION 18

Screening for postpartum depression and anxiety using a validated instrument is recommended and should be accompanied by diagnostic and management services for women who screen positive. *(Recommended)*

Remarks

- Screening for common mental health conditions in the postnatal period should be performed using a validated instrument, such as the Edinburgh Postnatal Depression Scale (EPDS) or Patient Health Questionnaire-9 (PHQ-9). All women should be asked about their emotional well-being at each postnatal care contact.
- The Guideline Development Group noted that trials showing a reduction in postpartum depression and anxiety included universal screening for mental health conditions by trained health workers, coupled with confirmatory diagnosis and treatment strategies.
- Systems for referral, diagnosis and management of women should be established or strengthened to ensure adequate follow-up and management for those who screen positive, in accordance with principles of screening programmes *(87)*. Women identified at risk of postpartum depression or anxiety based on screening results should be offered psychosocial and/or psychological interventions to prevent these conditions as per Recommendation 19 in this guideline.

Summary of evidence and considerations

Effects of the interventions (EB Table A.4.1)

Evidence was derived from a systematic review of screening for common mental disorders among pregnant and postpartum women *(88)*, including six studies involving 13 728 women. One was an RCT (4621 women), three were cluster-RCTs (7930 women) and two were quasi-RCTs (5336 women). Trials were conducted in China, Hong Kong Special Administrative Region (1), the Netherlands (1), Norway (1), the United Kingdom (2) and the USA (1) between 1997 and 2014. The scope of all trials was universal screening for women.

Five trials employed the EPDS for assessing rates of depression, of which three trials also employed Schedule for Clinical Assessment in Neuropsychiatry (SCAN) interviews and clinical assessments by physicians and public health nurses as a confirmatory test for postpartum depression. One trial implemented a two-step approach with a self-administered EPDS for screening and a physician evaluation using the PHQ-9 for those screening positive. All screening was coupled with treatment strategies, including non-directive counselling, psychoeducation and pharmacological therapy. Assessment of postpartum depression was done in-person, online or via postal mail at varying time points from 3 to 12 weeks postpartum.

The results in this evidence summary are stratified by study design (RCTs and quasi-RCTs).

Comparison: Screening for common mental disorders (depression, anxiety) in the postpartum period compared with no screening or usual care

Maternal outcomes

Mental health condition

Postpartum depression

- Evidence from RCTs: Low-certainty evidence suggests screening for common mental disorders in postpartum women may reduce the rate of postpartum depression when compared with usual care (4 trials, 3164 women; OR 0.53, 95% CI 0.45 to 0.62).
- Evidence from quasi-RCTs: Low-certainty evidence suggests screening for common mental disorders in postpartum women may reduce the rate of postpartum depression when compared with usual care (2 trials, 5010 women; OR 0.30, 95% CI 0.24 to 0.48).

Postpartum anxiety

- Evidence from RCTs: High-certainty evidence suggests screening for common mental disorders in postpartum women reduces postpartum anxiety when compared with usual care (1 trial, 565 women; MD 0.28 fewer, 95% CI 0.44 fewer to 0.11 fewer).
- Evidence from quasi-RCTs: It is uncertain whether screening for common mental disorders in

postpartum women affects the postpartum anxiety rate when compared with usual care (very low-certainty evidence).

Maternal functioning/well-being

- Evidence from RCTs: Low-certainty evidence suggests screening for common mental disorders in postpartum women may improve quality of life when compared with usual care (2 trials, 2068 women; MD 0.24, 95% CI 0.11 to 0.38). Moderate-certainty evidence suggests screening for common mental disorders in postpartum women probably makes little or no difference to marital satisfaction when compared with usual care (2 trials, 1017 women; OR 0.56 lower, 95% CI 0.21 to 1.53).
- Evidence from quasi-RCTs: It is uncertain whether screening for common mental disorders in postpartum women affects quality of life when compared with usual care (very low-certainty evidence).

Short-term maternal morbidity

- Evidence from RCTs: High-certainty evidence suggests screening for common postpartum mental disorders reduces parental stress when compared with usual care (3 trials, 1582 women; OR 0.57, 95% CI 0.45 to 0.74).
- Evidence from quasi-RCTs: It is uncertain whether screening for common mental disorders in postpartum women affects parental stress when compared with usual care (very low-certainty evidence).

Health service use

- Evidence from RCTs: Low-certainty evidence suggests screening for common postpartum mental disorders may increase treatment seeking practices when compared with usual care (2 trials, 1017 women; OR 3.45, 95% CI 2.52 to 4.70).

Adverse effects: The review authors were not able to pool results for adverse effects reported in two studies (4546 women). One trial (462 women) reported no adverse effects. The other trial (4084 women) reported no hospital or psychiatric admissions due to adverse events. Contacts with other mental health or social workers were rare in the screening group.

Experience of postnatal care was not reported in the included trials, and subgroup analyses were not possible due to the limited number of studies.

Additional considerations

The systematic review reported additional neonatal/infant outcomes that were not included in this evidence summary. Infant outcomes were reported in three studies, showing a small improvement in child socioemotional development in the intervention group (SMD = −0.10, 95% CI −0.16 to −0.04, 4050 infants, I^2 = 0%). No improvement was perceived in physical development of the infants (SMD = 0.09, 95% CI −0.02 to 0.19, 1486 infants, I^2 = 0%). One study reported an improvement in parent-child interaction (SMD = 0.32, 95% CI 0.13 to 0.52, n = 565, I^2 = 26.52%). The number of doctor visits for the child increased among the intervention group (SMD = 0.19, 95% CI 0.01 to 0.34, n = 462). However, no differences were noted in number of hospitalizations (SMD = 0.06, 95% CI −0.13 to 0.24, n = 462).

The primary publication of this systematic review included three trials (914 women) where screening for common postpartum mental disorders was conducted during pregnancy. It revealed a reduction in rates of depressive symptoms among women in the postpartum period (OR 0.68, 95% CI 0.48 to 0.96).

Values

See Box 3.5 in section 3.A.4: Mental health interventions.

Resources

The systematic review identified one study (high quality) evaluating cost-effectiveness of screening programmes *(89)*. General screening for postpartum depression was done either face-to-face or through postal questionnaires. The women who screened positive for postpartum depression were either offered treatment based on cognitive behavioural approaches or non-directive counselling. In the case of severe symptoms such as suicidality, women were referred for psychiatric support. The intervention group reported a greater number of quality-adjusted life-year (QALY) (+0.003, 95% CI 0.001 to 0.006) and fewer total costs (−36.035, 95% CI −68.423 to −3.646). The trial arm receiving cognitive behavioural approaches reported a higher rise in QALYs, was more cost-effective and also reported fewer contacts with health services. When QALYs were considered to range between £20 000 and £30 000, the probability for cost-effectiveness was over 70% for this group.

Table 3.49 Main resource requirements for screening for common mental disorders (depression, anxiety) in the postpartum period

Resource	Description
**Staff**	• Midwives (community/public health), nurses, health visitors, social workers and/or mental health professionals (e.g. counsellors, psychotherapists, clinical psychologists, psychiatrists, community psychiatric nurses)
**Training**	• Special training and support for the staff administering the screening and assessment of women (e.g. midwives, nurses, health visitors, social workers)
**Supplies**	• Screening measures/tools/questionnaires
**Equipment and infrastructure**	• Might require redesigning or enhancing organization of postnatal care, for example by establishing and integrating networks of public health nurses, health visitors, social workers and/or mental health professionals in the delivery of postnatal care
**Time**	• Time associated with the screening<br>– EPDS: 10 minutes face-to-face, 5 minutes self-administered<br>– PHQ: 3–10 minutes depending on the version
**Supervision and monitoring**	• Ongoing support and supervision from physicians and psychiatric supervising teams

Equity

No direct evidence was identified on the impact on health equity of screening for common mental disorders (depression, anxiety) in the postpartum period. In general, screening would increase equity, especially if programmes are in place to provide management of conditions if they are detected. If screening tools are not universal and culturally adapted, then illiterate women and those from minority groups may be left out, especially if these are self-administered tools. This would potentially lead to inequity.

Additional considerations

Women affected by social inequalities have an increased risk of many common postpartum mental disorders and their adverse consequences *(90)*.

Acceptability

Evidence from a qualitative evidence synthesis exploring women's experiences of postnatal care *(28)* highlights an often unvoiced and unmet desire among women for acknowledgement of their own care needs, especially their mental and emotional well-being (high confidence in the evidence). Women expect and appreciate that postnatal health-care resources should primarily be focused on the well-being of their baby and sometimes feel awkward or guilty about asking for help with their own needs (moderate confidence in the evidence). However, most women acknowledge that they need lots of support during the transition to motherhood and appreciate professional help with emotional and psychosocial concerns (high confidence in the evidence).

A qualitative evidence synthesis of health workers' views and experience on provision of postnatal care *(29)* suggests that women may hesitate to acknowledge mental health issues and seek treatment due to concerns about stigma and being judged as an unfit parent (low confidence in the evidence).

Findings from a synthesis on the perspectives of women, men, and health workers on discharge preparedness, derived from a scoping review on discharge preparation and discharge readiness *(91)*, indicate that care for women is often seen to be overlooked during the postnatal care period, with predominant emphasis put on the care of the baby (moderate confidence in the evidence) and assumptions that women receive the information they need during antenatal care (low confidence in the evidence). The discharge process is often viewed as rushed by both women and health workers (low confidence in the evidence). Furthermore, women are often unprepared for the pain and discomfort they experience and lack practical education on taking care of themselves and their newborns and support with postpartum depression (low confidence in the evidence).

Additional considerations

Several qualitative studies exploring women's perceptions of screening tools for postnatal depression *(92)* suggest that tools like the EPDS are useful to women as long as they are not used as an end point, simply to confirm or rule out depression, but rather as a starting point for a genuine conversation about psychosocial well-being. For some women there was a perception that mental health screening might be viewed as a test for so called normality, leading to subsequent concerns about feeling stigmatized. Other women felt that screening could be intrusive and insensitive and highlighted the importance of kind, well trained staff in the screening process.

Feasibility

A qualitative evidence synthesis of women's experiences of postnatal care *(28)* found no direct evidence relating to feasibility of screening for common maternal mental disorders. However, indirect evidence indicates that some women value the time they spend with health workers and do not like to feel hurried through postnatal assessments without having the opportunity to discuss assessment related concerns (low confidence in the evidence). The availability of adequately staffed health facilities with sensitive, well-trained personnel is an important consideration for women (moderate confidence in the evidence).

Buist et al. *(93)*, in their evaluation of screening programmes for postpartum depression in Australia, provided health workers' perceptions on use of EPDS. The majority of nurses (83%), midwives (76%) and general practitioners (71%) reported that EPDS was easy to use.

A qualitative evidence synthesis of health workers' views and experience on the provision of postnatal care *(29)* suggests that lack of personnel and resources may limit the offer of postnatal screening for common mental disorders in the postpartum period (moderate confidence in the evidence). Health workers reported limited knowledge, training and experience in screening women to identify postpartum mental health issues (moderate confidence in the evidence), including the provision of culturally sensitive care and support for women (low confidence in the evidence), and knowledge about referrals and available services for women with mental health conditions.

Additional considerations

Screening for postpartum depression can be embedded in routine health-care information systems or in population-based screening at community level through household surveys.

After screening for common postpartum mental disorders, provision of diagnosis and treatment should be a priority for health systems. The preferred choice and acceptability for delivery agents should also be explored. In some settings, seeking treatment from general practitioners for mental health conditions may not be accepted due to the belief that general practitioners are more suitable for treating physical conditions *(89)*.

Table 3.50 Summary of judgements: Screening for common mental disorders (depression, anxiety) in the postpartum period compared with no screening or usual care

Domain	Judgement
Desirable effects	Moderate
Undesirable effects	Trivial
Certainty of the evidence	Low
Values	Probably no important uncertainty or variability
Balance of effects	Probably favours screening
Resources required	Moderate costs
Certainty of the evidence on required resources	Moderate
Cost-effectiveness	Probably favours screening
Equity	Probably increased
Acceptability	Probably yes
Feasibility	Probably yes

A.4.2 Prevention of postpartum depression and anxiety

RECOMMENDATION 19

Psychosocial and/or psychological interventions during the antenatal and postnatal period are recommended to prevent postpartum depression and anxiety. *(Recommended)*

Remarks

- All women during antenatal and postnatal care would benefit from psychosocial interventions such as psychoeducation[37] to develop coping strategies, manage stress and build supportive networks, where feasible and with availability of resources. The Guideline Development Group agreed that psychosocial interventions to support maternal mental health are an important component of early childhood health and development (see Recommendation 41 in this guideline).
- Women with clinically significant symptoms or risk factors should be offered psychological interventions (e.g. cognitive behavioural therapy or interpersonal therapy).
- The provision of these interventions should be decided in a collaborative manner based on the woman's preference and the care provider's ability to deliver the intervention in terms of training, expertise and experience.
- Women at risk for postpartum depression and/or anxiety are women who either (i) have exhibited depressive symptoms but scored below the cut-off for depressive disorder on screening tests, (ii) had previous episodes of depression, or (iii) have social risk factors such as low income, intimate partner violence or being an adolescent.

Summary of evidence and considerations

Effects of the interventions (EB Table A.4.2)

Evidence was derived from a systematic review of psychological and psychosocial interventions for prevention of common postpartum mental disorders *(94)*. The data were derived from 44 studies (23 542 women), with the earliest study published in 1995. Most studies (38) were conducted in HICs. These interventions were focused on either universal populations (21) or targeted populations (23), including women with social stressors, with sub-threshold depressive symptoms at baseline, or specific populations such as adolescents.

The majority of interventions were psychosocial in nature (28); the rest (16) had a psychological theoretical underpinning. Psychosocial interventions included psychoeducational strategies, home visits by allied health professionals or lay health workers, or social support interventions. Psychological interventions included cognitive behavioural therapy, interpersonal psychotherapy, mindfulness, mind–body interventions and mood monitoring. Interventions were employed either by mental health specialists or non-specialists in mental health including health professionals trained in specific therapies (e.g. general practitioners, midwives, nurses and allied health workers, peers and partners). All interventions were delivered face-to-face except one, which was delivered using an online medium. Heterogeneous tools and psychometric scales were used for assessment of depression and anxiety in the included studies.

All interventions, irrespective of their time point of delivery (antenatal period, postnatal period, or both), aimed to prevent either postpartum depression or anxiety. Therefore, all outcomes reported in the systematic review were measured during the postpartum period. This report provides separate meta-analyses for interventions according to the timing of intervention delivery.

37 Psychoeducation is an evidence-based intervention with systematic, structured information sharing about symptoms of depression/anxiety and their prevention and treatment. It may also integrate emotional and motivational aspects of the conditions and teach problem-solving and communication skills, providing education and resources in an empathetic and supportive environment.

Comparison: Interventions to prevent common mental disorders (depression, anxiety) in the postpartum period, delivered at any time, compared with no intervention or usual care

Maternal outcomes

Mental health condition

Postpartum depression

Moderate-certainty evidence suggests interventions to prevent common postpartum mental disorders, with components delivered at any time point, probably reduce the rate of postpartum depression when compared with no interventions or usual care (9 trials, 1831 women; OR 0.61, 95% CI 0.38 to 0.99). Low-certainty evidence suggests interventions to prevent common postpartum mental disorders, with components delivered at any time point, may reduce postpartum depression severity when compared with no interventions or usual care (38 trials, 20 569 women; SMD 0.29 lower, 95% CI 0.44 lower to 0.15 lower).

Postpartum anxiety

Moderate-certainty evidence suggests interventions to prevent common postpartum mental disorders, with components delivered at any time point, probably reduce the rate of postpartum anxiety (4 trials; OR 0.20, 95% CI 0.04 to 0.89), and postpartum anxiety severity (9 trials, 1796 women; SMD 0.79 lower, 95% CI 1.30 lower to 0.28 lower), when compared with no interventions or usual care.

Maternal functioning/well-being: Moderate-certainty evidence suggests interventions to prevent mental disorders, with components delivered at any time point, probably reduce marital discord (7 trials, 1563 women; SMD 0.33 lower, 95% CI 0.54 lower to 0.12 lower), and probably improve maternal infant attachment (6 trials, 2078 women; SMD 0.11 lower, 95% CI 0.20 lower to 0.02 lower), when compared with no intervention or usual care. Low-certainty evidence suggests interventions to prevent mental disorders, with components delivered any time point, may have little or no effect on perceived social support (9 trials, 8416 women; SMD 0.002 higher, 95% CI 0.05 lower to 0.05 higher), exclusive breastfeeding (1 trial, 2438 women; OR 1.02, 95% CI 0.81 to 1.27), or breastfeeding initiation practices (2 trials, 1210 women; OR 1.10, 95% CI 0.90 to 1.33), when compared with no intervention or usual care.

Short-term maternal morbidity: Low-certainty evidence suggests interventions to prevent mental disorders with components delivered at any time point may have little or no effect on paternal stress when compared with no intervention or usual care (4 trials, 592 women; SMD 0.07 higher, 95% CI 0.21 lower to 0.34 higher).

Experience of postnatal care: Low-certainty evidence suggests interventions to prevent mental disorders, with components delivered at any time point, may reduce maternal dissatisfaction when compared with no intervention or usual care (8 trials, 4007 women; SMD 0.36 lower, 95% CI 0.60 lower to 0.12 lower).

Health service use: It is uncertain whether interventions to prevent maternal mental disorders, with components delivered antenatally, have an effect on treatment seeking practices when compared with no interventions or usual care (very low-certainty evidence).

Adverse effects were not reported in the included trials.

Outcomes by time of intervention (antenatal only, antenatal and postpartum, postpartum only)

Mental health condition

Postpartum depression

- Interventions delivered in the antenatal period only: It is uncertain whether interventions to prevent common postpartum mental disorders with components delivered antenatally have an effect on the rate of postpartum depression when compared with no intervention or usual care (very low-certainty evidence).
- Interventions with components delivered in both, antenatal and postpartum periods: Low-certainty evidence suggests interventions to prevent mental disorders with components delivered both antenatally and postnatally may have little or no effect on the rate of postpartum depression when compared with no intervention or usual care (5 trials 594 women; OR 0.57, 95% CI 0.27 to 1.18).
- Interventions delivered in the postpartum period only: Moderate-certainty evidence suggests interventions to prevent common postpartum mental disorders with components delivered postnatally probably have little or no effect on the rate of postpartum depression when compared with no intervention or usual care (2 trials, 1012 women; OR 0.82, 95% CI 0.48 to 1.41).

Postpartum depression severity

- Interventions delivered in the antenatal period only: Low-certainty evidence suggests interventions to prevent common postpartum mental disorders with components delivered

antenatally may reduce postpartum depression severity when compared with no intervention or usual care (9 trials, 3006 women; MD 0.70 lower, 95% CI 1.17 lower to 0.24 lower).

- Interventions with components delivered in both antenatal and postpartum periods: Moderate-certainty evidence suggests interventions to prevent common postpartum mental disorders with components delivered both antenatally and postnatally probably reduce postpartum depression severity when compared with no intervention or usual care (14 trials, 3485 women; MD 0.10 lower, 95% CI 0.20 lower to 0.01 lower).
- Interventions delivered in the postpartum period only: It is uncertain whether interventions to prevent common postpartum mental disorders with components delivered postnatally have an effect on postpartum depression severity when compared with no intervention or usual care (very low-certainty evidence).

Postpartum anxiety severity

- Interventions delivered in the antenatal period only: It is uncertain whether interventions to prevent common postpartum mental disorders with components delivered in the antenatal period have an effect on severity of postpartum anxiety when compared with no intervention or usual care (very low-certainty evidence).
- Interventions with components delivered in both antenatal and postpartum periods: Low-certainty evidence suggests that interventions to prevent common postpartum mental disorders with components delivered both antenatally and postnatally may have little or no effect on postpartum anxiety severity when compared with no intervention or usual care (3 trials, 171 women, MD 0.20 lower, 95% CI 0.50 lower to 0.11 higher).
- Interventions with components delivered in the postpartum period only: Moderate-certainty evidence suggests that interventions to prevent common postpartum mental disorders with components delivered postnatally probably reduce the severity of postpartum anxiety when compared with no interventions or usual care (4 trials, 1193 women; MD 0.45 lower, 95% CI 0.88 lower to 0.02 lower).

Additional considerations

Additional analyses were performed to evaluate the effectiveness of included interventions in improving postpartum depressive symptoms, by type of intervention and population focus. Twenty-one studies targeted populations with risk factors or prodromal symptoms of depression, and 20 of these focused on universal populations. Interventions designed for universal populations had a greater effect on postpartum depressive symptoms (SMD = −0.33, 95% CI −0.57 to −0.10) than their counterparts (SMD = −0.21, 95% CI −0.33 to −0.09). However, this subgroup analysis did not achieve statistical significance ($P = 0.64$). Twenty-six studies reported on the effectiveness of psychosocial interventions for depressive symptoms while 15 were underpinned by a psychological theory. Psychological interventions had a greater effect on postpartum depressive symptoms (SMD = −0.44, 95% CI −0.77 to −0.10) than their counterparts (SMD = −0.22, 95% CI −0.40 to −0.05). However, this subgroup analysis did not achieve statistical significance ($P = 0.20$).

Values

See Box 3.5 in section 3.A.4: Mental health interventions.

Resources

The systematic review identified two studies evaluating cost-effectiveness of prevention interventions for common postpartum mental disorders. One study (high quality) presented a cost-effectiveness analysis for a volunteer-based programme for the prevention of postpartum depression among high risk women in Canada, and reported a mean cost per woman of Can$ 4497 in the peer support group and Can$ 3380 in the usual care group (difference of Can$ 1117, $P < 0.0001$) *(95)*. There was a 95% probability that the programme would cost less than Can$ 20 196 per case of postpartum depression averted. Although this was a volunteer-based programme, it resulted in a net cost to the health system and society. However, this cost was within the range for other accepted interventions for this population *(95)*. Another study (moderate quality) evaluated the psychoeducational intervention for postpartum women "What Were We Thinking", reporting no difference in costs between the intervention recipients and their control counterparts *(96)*. The incremental cost-effectiveness ratios were AU$ 36 451 per QALY gained and AU$ 152 per percentage point reduction in 30-day prevalence of depression, anxiety, and adjustment disorders. The estimate lies under the unofficial cost-effectiveness threshold of AU$ 55 000 per QALY; however, there was considerable variability surrounding the results, with a 55% probability that the programme would be considered cost-effective at that threshold *(96)*.

Table 3.51 Main resource requirements for interventions to prevent common mental disorders (depression, anxiety) in the postpartum period

Resource	Description
**Staff**	• Midwives, health visitors, facilitators, pregnancy outreach workers, multidisciplinary teams of nurses and graduates, or mental health professionals (e.g. social workers, clinical and health psychologists, multidisciplinary teams of reproductive health and mental health nurses)
**Training**	• Special training and support for the staff (e.g. midwives, nurses, health visitors, social workers) in delivering the intervention
**Supplies**	• Online media, self-help apps, workshop/training/educational materials, booklets<br>• Tools to evaluate maternal mental health disorders
**Equipment and infrastructure**	• Might require redesigning or enhancing organization of postnatal care, for example by way of establishing and integrating networks of health visitors, social workers and/or mental health professionals in the delivery of postpartum care
**Time**	• Time associated with the delivery of the intervention and follow-up care for women
**Supervision and monitoring**	• Ongoing support and supervision from physicians and psychiatric supervising teams

Equity

No direct evidence was identified on the impact on health equity of interventions to prevent common mental disorders (depression, anxiety) in the postpartum period. In general, interventions to prevent common postpartum mental disorders can increase equity. However, if they are not universal and culturally adapted then minorities and illiterate women may be left out. This would potentially lead to inequity.

Additional considerations

Women affected by social inequalities have increased risk of many common postpartum mental disorders and their adverse consequences *(90)*.

Acceptability

Evidence from a qualitative evidence synthesis exploring women's experiences of postnatal care *(28)* highlights an often unvoiced and unmet desire among women for acknowledgement of their own health-care needs, especially their mental and emotional well-being (high confidence in the evidence). Findings also indicate that women may experience low mood, depression, fatigue, insecurity and anxiety during the transition to motherhood (high confidence in the evidence) and welcome psychosocial support with these issues, provided the support is delivered by suitably trained health workers who are sensitive to their needs and life circumstances (high confidence in the evidence). In addition, women appreciate continuity (of care and carer) so any intervention that is delivered during both the antenatal and postnatal phases should, ideally, be provided by the same person (high confidence in the evidence).

Findings from a synthesis on the perspectives of women, men and health workers on discharge preparedness, derived from a scoping review on discharge preparation and discharge readiness *(91)*, indicate that care for women is often seen to be overlooked during the postnatal care period, with predominant emphasis put on the care of the baby (moderate confidence in the evidence) and assumptions that women receive the information they need during antenatal care (low confidence in the evidence). The discharge process is often viewed as rushed by both women and health workers, including provision of too much information, women wanting to get home early, time limitations and health workforce shortage (low confidence in the evidence). Furthermore, women are often unprepared for the pain and discomfort they experience and lack practical education on taking care of themselves and their newborns and support about postpartum depression (low confidence in the evidence).

Additional considerations

Several qualitative studies *(97–99)* exploring women's postpartum mental health indicate that women sometimes struggle to identify mental health conditions and/or don't know who to

approach or how to access support. They may feel overwhelmed or inadequate and develop coping strategies or engage in self-denial to disguise mental health concerns. Findings also indicate that women appreciate regular home visits from health workers provided the support offered is empowering rather than undermining or patronizing.

Feasibility

A qualitative evidence synthesis of women's experiences of postnatal care *(28)* found no direct evidence relating to women's views on the feasibility of interventions to prevent common mental disorders (depression, anxiety) in the postpartum period. However indirect evidence from this review suggests that personal qualities relating to empathy, sensitivity and discretion are important to women, so health professionals offering therapeutic interventions may require additional training in these skills (moderate confidence in the evidence).

A qualitative evidence synthesis of health workers' experiences of postnatal care *(29)* found no direct evidence relating to views on the feasibility of interventions to prevent common mental disorders (depression, anxiety) in the postpartum period. However, indirect evidence suggests that a lack of personnel, resources and training may limit provision of information, counselling and preventive interventions in the postnatal period (moderate confidence in the evidence).

Additional considerations

Integrating psychosocial and psychological interventions for prevention of common postpartum mental disorders into routine maternal and child health service will enhance feasibility. Delivery of interventions by trained non-specialists also enhances feasibility.

Table 3.52 Summary of judgements: Interventions to prevent common mental disorders (depression, anxiety) in the postpartum period, delivered at any time, compared with no intervention or usual care

Domain	Judgement
Desirable effects	Small
Undesirable effects	Don't know
Certainty of the evidence	Low
Values	Probably no important uncertainty or variability
Balance of effects	Probably favours intervention
Resources required	Moderate costs
Certainty of the evidence on required resources	Moderate
Cost-effectiveness	Probably favours intervention
Equity	Probably increased
Acceptability	Probably yes
Feasibility	Varies

A.5 NUTRITIONAL INTERVENTIONS AND PHYSICAL ACTIVITY

Background

This section of the guideline includes three sets of recommendations that have been integrated from WHO guidelines on postpartum nutritional supplementation and physical activity and that are relevant to routine postnatal care.

A.5.1 Postpartum oral iron and folate supplementation

RECOMMENDATION 20

Oral iron supplementation, either alone or in combination with folic acid supplementation, may be provided to postpartum women for 6–12 weeks following childbirth for reducing the risk of anaemia in settings where gestational anaemia is of public health concern.[a] *(Context-specific recommendation)*

Remarks

- This recommendation has been integrated from the 2016 WHO publication *Iron supplementation in postpartum women (100)*, where it was considered a conditional recommendation based on low-certainty evidence.
- The following remarks were among those made by the Guideline Development Group responsible for the original recommendation.
  - This recommendation is applicable to all postpartum women, irrespective of their lactation status.
  - For ease of implementation and continuity of care, postpartum supplementation should begin as early as possible after birth, and the iron supplementation regimen (that is, the dose and whether the supplement is consumed daily or weekly) should follow that used during pregnancy *(16)* or alternatively should start with that planned for non-pregnant adult women and adolescent girls *(101, 102)*.
  - In cases in which a woman is diagnosed with anaemia in a clinical setting *(103)*, she should be treated in accordance with the country's policy, or the WHO recommendation of daily iron supplements (120 mg of elemental iron plus 400 µg folic acid), until her haemoglobin concentration rises to normal *(103, 104)*.
  - Postpartum and lactating women should be encouraged to receive adequate nutrition, which is best achieved through consumption of a balanced, healthy diet *(78, 105)*.

[a] WHO considers a 20% or higher population prevalence of gestational anaemia to be a moderate public health problem.

A.5.2 Postpartum vitamin A supplementation

RECOMMENDATION 21

Vitamin A supplementation in postpartum women for the prevention of maternal and infant morbidity and mortality is not recommended. *(Not recommended)*

Remarks

- This recommendation has been integrated from the 2011 WHO publication *Vitamin A supplementation in postpartum women (106)*, where it was considered a strong recommendation based on very low-certainty evidence.
- The Guideline Development Group responsible for the original recommendation agreed that postpartum and/or lactating women should be encouraged to receive adequate nutrition, which is best achieved through consumption of a balanced, healthy diet *(78, 105)*.

A.5.3 Physical activity and sedentary behaviour

RECOMMENDATION 22

All postpartum women without contraindication should:

- **undertake regular physical activity throughout the postpartum period;**
- **do at least 150 minutes of physical activity throughout the week for substantial health benefits; and**
- **incorporate a variety of physical and muscle-strengthening activities; adding gentle stretching may also be beneficial.** *(Recommended)*

RECOMMENDATION 23

Postpartum women should limit the amount of time spent being sedentary. Replacing sedentary time with physical activity of any intensity (including light intensity) provides health benefits. *(Recommended)*

Remarks

- These recommendations have been adapted and integrated from the 2020 *WHO guidelines on physical activity and sedentary behaviour (107)*, where they were considered strong recommendations based on moderate and low-certainty evidence, respectively.
- The postnatal care Guideline Development Group noted the following based on the original guidelines.
  - For postpartum women, physical and muscle-strengthening activities can be undertaken as part of recreation and leisure (e.g. play, games, leisure sports or planned exercise), transportation (e.g. walking), work and household tasks, in the context of daily occupational, educational, home and/or community settings. Postpartum women should try to meet these recommendations where possible, as able, and without contraindication, and with the support of their partners and families. Clinical guidance should be sought for women with complications associated with pregnancy or childbirth.
  - 150 minutes of physical activity per week is equivalent of approximately 20–25 minutes of walking per day. The 150 minutes does not need to be continuous physical activity, but rather can accumulate over the course of the day.
- Good practice statements are as follows.
  - If postpartum women are not meeting the level of physical activity in the recommendations, doing some physical activity will benefit their health.
  - Postpartum women should start by doing small amounts of physical activity, and gradually increase frequency, intensity and duration over time.
- Additional safety considerations are as follows.
  - Postpartum women should:
    - be informed by their health-care provider of the danger signs alerting them as to when to stop, or to limit physical activity and consult a qualified health worker immediately should they occur;
    - return to physical activity gradually after childbirth, and in consultation with a health worker, in particular in the case of caesarean birth; and
    - be advised by their provider on special considerations given their history and any contraindications to participating in physical activity during the postpartum period.
- Related recommendations from this guideline include the following.
  - Women who, before pregnancy, habitually engaged in vigorous-intensity aerobic activity, or who were physically active, can continue these activities during pregnancy and the postpartum period.
- Sedentary behaviour is defined as time spent sitting or lying with low energy expenditure while awake, in the context of occupational, educational, home and community settings and transportation.

A.6 CONTRACEPTION

Background

This section of the guideline includes one recommendation, which has been integrated from a WHO guideline on the provision of contraceptive information and services, and which is relevant to routine postnatal care.

A.6.1 Postpartum contraception

RECOMMENDATION 24

Provision of comprehensive contraceptive information and services during postnatal care is recommended. *(Recommended)*

Remarks

- This recommendation has been adapted and integrated from the 2014 WHO document *Ensuring human rights in the provision of contraceptive information and services: guidance and recommendations (108)*. The current *WHO recommendations on contraceptive methods for postpartum and/or lactating women* are specified in Box 3.6, based on the 2015 WHO publication *Medical eligibility criteria for contraceptive use (109)*. WHO recommendations for postpartum contraception should be checked regularly for any updates.
- The postnatal care Guideline Development Group noted the following based on existing WHO documents.
  - All postpartum women and couples should be offered evidence-based, comprehensive contraceptive information, education and counselling to ensure informed choice for their own use of modern contraception without discrimination. Privacy of individuals should be respected throughout the provision of contraceptive information and services, including confidentiality of medical and other personal information *(108)*.
  - The mode of birth does not restrict a postpartum woman's contraceptive choice.
  - Breastfeeding women ≥ 6 months postpartum can use progestogen-only pills (POPs), progestogen-only injectable contraceptives (POIs), levonorgestrel (LNG) and etonogestrel (ETG) implants without restriction (medical eligibility criteria [MEC] Category 1) and can generally use combined hormonal contraception (CHC) (MEC Category 2).[a]
  - A woman's risk of HIV infections does not restrict her contraceptive choice, and women are eligible to use all progestogen-only contraceptive methods, copper-bearing intrauterine devices (Cu-IUDs) and levonorgestrel-releasing intrauterine device (LNG-IUDs) without restriction as per the WHO guidance statement *Contraceptive eligibility for women at high risk of HIV (110)*.
  - Self-administered injectable contraception should be made available as an additional approach to deliver injectable contraception for individuals of reproductive age, as per the 2019 *WHO consolidated guideline on self-care interventions for health: sexual and reproductive health and rights (111)*, and based on eligibility according to the WHO MEC for contraceptive use.
  - The WHO guidance on MEC includes a range of other contraceptive methods that are self-administered, including the combined contraceptive patch, the combined contraceptive vaginal ring, the progesterone-releasing vaginal ring (PVR) and barrier methods, including condoms (male latex, male polyurethane and female condoms), the diaphragm (with spermicide) and the cervical cap. Women who are breastfeeding can also choose to use contraceptive methods together with the lactational amenorrhoea method during the first six months postpartum.
  - Ongoing competency-based training and supervision of health workers on the delivery of contraceptive education, information and services, should be based on existing WHO guidelines.

a MEC: medical eligibility criteria; MEC categories for contraceptive eligibility are: MEC Category 1 – a condition for which there is no restriction for the use of the contraceptive method; MEC Category 2 – a condition where the advantages of using the method generally outweigh the theoretical or proven risks; MEC Category 3 – a condition where the theoretical or proven risks usually outweigh the advantages of using the method; MEC Category 4 – a condition which represents an unacceptable health risk if the contraceptive method is used.

Box 3.6 Recommendations for contraceptive methods for postpartum women, including lactating women *(109)*

**Less than six weeks postpartum**
Postpartum women who are < 48 hours postpartum can use Cu-IUDs (MEC Category 1).
Breastfeeding women who are < 48 hours postpartum can generally use LNG-IUDs (MEC Category 2).
Breastfeeding women who are < 6 weeks postpartum can generally use POPs and LNG and ETG implants (MEC Category 2).
Breastfeeding women who are ≥ 4 weeks postpartum can use the PVR without restrictions (MEC Category 1).
Postpartum women who are ≥ 4 weeks postpartum can use Cu-IUDs and LNG-IUDs without restriction (MEC Category 1).
Postpartum women who are ≥ 21 days to 42 days postpartum without other risk factors for venous thromboembolism (VTE) can generally use CHCs (MEC Category 3).
**Six weeks to less than six months postpartum**
Breastfeeding women who are ≥ 6 weeks to < 6 months postpartum can use POPs, POIs, and LNG and ETG implants without restriction (MEC Category 1).
Postpartum women who are > 42 days postpartum can use CHCs without restriction (MEC Category 1).
**Recommendations against some contraceptive methods**
Postpartum women who are ≥ 48 hours to < 4 weeks postpartum generally should not have an LNG-IUD inserted (MEC Category 3).
Breastfeeding women who are < 6 weeks postpartum generally should not use POIs (depot medroxyprogesterone acetate or norethisterone enanthate) (MEC Category 3).
Breastfeeding women < 6 weeks postpartum should not use CHCs (MEC Category 4).
Breastfeeding women ≥ 6 weeks to < 6 months postpartum generally should not use CHCs (MEC Category 3).
Postpartum women who are < 21 days postpartum and do not have other risk factors for VTE generally should not use CHCs (MEC Category 4).
Postpartum women who are < 21 days postpartum with other risk factors for VTE should not use CHCs (MEC Category 2).
Postpartum women who are ≥ 21 days to 42 days postpartum with other risk factors for VTE generally should not use (MEC Category 3).
**Recommendations on emergency contraception**
Breastfeeding women can use combined oral contraceptive pills or LNG for emergency contraceptive pills (ECPs) without restriction (MEC Category 1).
Women who are breastfeeding can generally use ulipristal acetate for ECPs (MEC Category 2).

B. Newborn care

B.1 NEWBORN ASSESSMENT

Background

Early detection of conditions that may adversely affect the health and development of the newborn is an important component of quality routine postnatal care.

For guidance on routine assessment of the newborn, health workers should refer to WHO's operational manual *(39)*, in which detailed guidance on assessment of the newborn is provided.

In this section of the guideline, the GDG considered the evidence and other relevant information to inform recommendations on universal screening for the following conditions.

Abnormalities of the eye

An estimated 1.14 million children aged 0–15 years are blind or severely visually impaired from eye conditions *(112)*. The major causes of blindness in children are congenital and developmental catarsact, corneal scarring, congenital eye anomalies, retinal dystrophies, glaucoma and retinopathy of prematurity. Conditions which are present at birth can be detected by screening during the neonatal period, and affected newborns can be referred for treatment, such as surgery for cataract, glaucoma and retinoblastoma (a malignant tumour), longer term follow-up, or referral for a general health assessment or vision rehabilitation *(113)*. Newborn eye screening is usually undertaken using a torch light to examine the external structures of the eye, and red reflex testing, which requires a direct ophthalmoscope or a suitable alternative.

Hearing impairment

The prevalence of severe to profound permanent bilateral hearing loss (PBHL) in newborns is reported to be 1–1.5 per 1000 newborns in HICs *(114, 115)*. PBHL is defined as bilateral permanent conductive (bone, ear canal or ear drum problems) or sensorineural (nerve or brain damage) hearing loss of 35 dB or greater in the better ear *(116)*. It occurs during the intrauterine or congenital period (due to infections such as TORCH [toxoplasmosis, rubella, cytomegalovirus, herpes simplex, syphilis], genetic and craniofacial problems) and also during the postnatal period (e.g. due to intracranial insults, meningitis, jaundice, ototoxic antibiotics). Two tests that can detect hearing loss in newborns and infants under 6 months of age are otoacoustic emissions (OAE) and automated auditory brainstem response (AABR), used alone or in combination.

Hyperbilirubinaemia

Neonatal unconjugated (indirect) hyperbilirubinaemia is a common condition that affects approximately 60–80% of otherwise healthy newborns *(117, 118)*. It manifests in the first days after birth as jaundice. Biochemically, it is defined by an increase in total serum bilirubin (TSB) as a result of an elevated indirect serum bilirubin. Although most newborns present with physiological jaundice, which is frequently normal and benign, a subset of newborns will develop severe disease warranting treatment and necessitating hospitalization in the first weeks after birth *(117, 118)*. Severe jaundice, if not diagnosed and treated in time, can lead to acute bilirubin encephalopathy, bilirubin-induced neurological dysfunction (BIND) or, in the most severe cases, kernicterus and/or jaundice-related death. Clinically, jaundice is recognized by visual inspection, and the serum bilirubin levels are estimated non-invasively through the skin using a transcutaneous bilirubinometer (TcB) and/or confirmed invasively by blood sample and laboratory estimation of TSB. Visual inspection (with or without risk factor assessment) is a commonly used screening method, especially in resource-constrained settings. TSB is the most accurate method of serum bilirubin estimation, but it requires a heel prick and laboratory assessment.

In addition to the GDG recommendations and guidance on the above, this section of the guideline includes one recommendation on assessment of the newborn for danger signs that has been integrated from the 2014 *WHO recommendations on postnatal care of the mother and newborn (15)*.

Box 3.7 Values

Findings from a qualitative evidence synthesis exploring what women want from postnatal care *(21)* indicate that women want a positive experience in which they are able to adapt to their new self-identity and develop a sense of confidence and competence as a mother. They also want to adjust to changes in their intimate and family relationships (including their relationship to their baby), navigate ordinary physical and emotional challenges, and experience the dynamic achievement of personal growth as they adjust to their new normal, both as parents and as individuals in their own cultural context. Findings from the same review also indicate that women tend to prioritize the needs of their baby and are therefore likely to value information relating to, and practices and procedures that optimize, neonatal development (low confidence in the evidence).

Box 3.8 Acceptability of interventions

A qualitative evidence synthesis exploring what women want from postnatal care *(21)* found no direct evidence relating to women's views on the newborn assessments evaluated. Indirect evidence from the review indicates that women want clear and accurate information about their infant's well-being (high confidence in the evidence), provided it is delivered by knowledgeable health workers who are sensitive to their needs (moderate confidence in the evidence). Indirect evidence from a qualitative evidence synthesis of women's experiences of postnatal care suggests most women appreciate advice and information from health workers about neonatal development (high confidence in the evidence) *(28)*. Women also tend to prioritize the needs of their infant so are likely to appreciate procedures and techniques that optimize infant well-being (low confidence in the evidence).

Box 3.9 Feasibility of interventions

A qualitative evidence synthesis of women's experiences of postnatal care found no direct evidence relating to women's views on the feasibility of newborn screening *(28)*. However indirect evidence from this review indicates that some women value the time they spend with health workers and do not like to feel hurried through assessments without having the opportunity to discuss screening-related concerns (low confidence in the evidence). With this in mind, the availability of adequately equipped health facilities with sensitive, well-trained health workers are important considerations for some women (moderate confidence in the evidence).

A qualitative evidence synthesis of health workers' experiences of postnatal care found no direct evidence relating to views on the feasibility of newborn screening *(29)*. However, indirect evidence suggests that lack of personnel, resources and training may limit the provision of information and counselling on newborn screening and a complete physical examination of the newborn (moderate confidence in the evidence). Health workers across different settings reported differences in the extent to which they felt adequately trained to provide postnatal care, in particular newborn examinations (moderate confidence in the evidence).

B.1.1 Assessment of the newborn for danger signs

RECOMMENDATION 25

The following signs should be assessed during each postnatal care contact, and the newborn should be referred for further evaluation if any of the signs is present:

- **not feeding well**
- **history of convulsions**
- **fast breathing (breathing rate > 60 per minute)**
- **severe chest in-drawing**
- **no spontaneous movement**
- **fever (temperature > 37.5 °C)**
- **low body temperature (temperature < 35.5 °C)**
- **any jaundice in first 24 hours after birth, or yellow palms and soles at any age.**

The parents and family should be encouraged to seek health care early if they identify any of the above danger signs between postnatal care visits. *(Recommended)*

Remarks

- This recommendation has been adapted and integrated from the 2014 *WHO recommendations on postnatal care of the mother and newborn* (15), where it was considered a strong recommendation based on low-certainty evidence.
- No remarks were noted by the Guideline Development Group responsible for the original recommendation.

B.1.2 Universal screening for abnormalities of the eye

RECOMMENDATION 26

Universal newborn screening for abnormalities of the eye is recommended and should be accompanied by diagnostic and management services for children identified with an abnormality. *(Recommended)*

Remarks

- Universal newborn screening for abnormalities of the eye should be done prior to discharge after a health-facility birth or at the first postnatal care contact in an outpatient setting after a home birth. Ideally, the screening should be done within the first six weeks after birth.
- An external examination of the eye and red reflex test should be done using standard equipment (e.g. a direct ophthalmoscope) by a trained health worker.
- The Guideline Development Group (GDG) acknowledged the evidence reviewed related to screening for a single condition (congenital cataract). However, since the red reflex test can detect a wide range of conditions, the GDG expanded the recommendation to cover all abnormalities of the eye that may be detected on a screening examination.
- The recommendation is based on evidence from studies in all newborns, irrespective of gestation or presence/absence of high-risk factors. However, evidence from studies conducted only in high-risk populations such as preterm newborns or those with congenital anomalies was not considered.
- The extension of the recommendation to include diagnostics and management was made to incorporate the principles of screening *(87)*.
- Systems for screening, referral, diagnosis and management should be established or strengthened to ensure adequate follow-up and management for those who screen positive.

Summary of evidence and considerations

Effects of the interventions (EB Table B.1.2)

Evidence was derived from a systematic review of universal newborn eye screening *(113)*. The review identified 25 non-randomized studies involving 2 289 431 infants, of which three studies were included in this evidence summary.

Two studies were conducted in Sweden (724 523 newborns) using data collected from a paediatric, cataract-specific register. The first study compared two regions using different screening locations and another region where there was no screening, from 1992 to 1998 (total population of included regions covered almost 400 000 newborns). One region established red reflex testing with an external eye examination in the maternity ward during the first few days after birth, a second used the same screening tests performed in well-baby clinics at around 6 weeks of age, and no screening was carried out in the third. The second study added national data from 2007 to 2009 to the first study with eye screening established as routine in 90% of maternity wards (total population 394 438 newborns). Screening was via red reflex testing and an external eye examination by doctors or nurses. The third study was a before-and-after study conducted in Israel (18 872 newborns), evaluating the effect of introducing red reflex testing between 2008 and 2009, and between 2010 and 2011.

Comparison: Universal newborn screening for abnormalities of the eye compared with no screening

Newborn/infant outcomes

Severe neonatal/infant morbidity: Low-certainty evidence suggests universal screening for abnormal eye conditions in maternity wards may increase the proportion of newborns with congenital cataract referred from maternity wards or well-baby clinics in the first year after birth compared with no screening (1 study, 394 438 infants; RR 9.83, 95% CI 1.36 to 71.20). It is uncertain whether universal screening for abnormal eye conditions in well-baby clinics has any effect on the proportion of newborns with congenital cataract referred from maternity wards or

well-baby clinics in the first year after birth compared with no screening (very low-certainty evidence). It is uncertain whether universal screening for abnormal eye conditions has any effect on the proportion of newborns with congenital cataract referred from any health facility (maternity ward, well-baby clinic, by a paediatrician, or other) in the first year after birth compared with no screening, when screening is done in either maternity wards or well-baby clinics (very low-certainty evidence). Low-certainty evidence suggests universal screening for abnormal eye conditions in maternity wards may increase the proportion of newborns with congenital cataract referred within 42 days of birth when compared with no screening (1 study, 394 438; RR 4.61, 95% CI 1.12 to 19.01). It is uncertain whether universal screening for abnormal eye conditions in well-baby clinics has any effect on the proportion of newborns with congenital cataract referred within 42 days of birth when compared with no screening (very low-certainty evidence).

Health service use: Low-certainty evidence suggests universal screening for abnormalities of the eye in maternity wards may increase the proportion of newborns with congenital cataract operated on within 42 days of birth compared with no screening (1 study, 394 438 infants; RR 8.23, 95% CI 1.13 to 59.80). It is uncertain whether universal screening for abnormalities of the eye in well-baby clinics has any effect on the proportion of newborns with congenital cataract operated on within 42 days of birth compared with no screening (very low-certainty evidence).

Adverse effects: Low-certainty evidence suggests that universal screening for abnormal eye conditions using red reflex testing may increase the occurrence of clinical conjunctivitis compared with no screening (1 study, 18 870; OR 1.22, 95% CI 1.01 to 1.47). It is uncertain whether universal screening for abnormal eye conditions using red reflex testing increases the occurrence of bacterial conjunctivitis compared with no screening (very low-certainty evidence).

Neonatal/infant mortality and *neurodevelopment* were not reported in the included studies.

Maternal outcomes

Experience of postnatal care and *cost* were not reported in the included studies.

Data analyses for the pre-specified subgroups were not possible as the data were not available.

Additional considerations

One study (7641 healthy newborns) demonstrated high specificity (96.0%, 95% CI 95.6 to 96.5%) of red reflex testing using a direct ophthalmoscope for anterior segment and posterior segment conditions, and all conditions combined, when compared with the gold standard of a comprehensive eye examination (including digital imaging and indirect ophthalmoscopy or ultrasound as required) *(119)*, but sensitivity was 0% for posterior segment conditions and 66.7% (with wide confidence intervals) for anterior segment conditions.

A comparative study based on data from the Paediatric Cataract Register (PECARE) found that, among all the congenital cataract cases operated in the first year after birth, 13 per 100 000 children (561 743 newborns) were referred within 42 days (that is, by eight weeks) of birth when screened by early red reflex testing compared with 1.3 per 100 000 (population 308 181 newborns) who were screened using torch light examination *(120)*.

Values

See Box 3.7 in section 3.B.1: Newborn assessment.

Resources

No economic evaluations of universal screening for abnormalities of the eye for term, healthy newborns compared with no screening were identified.

Additional considerations

One study included in the systematic review assessed the cost-effectiveness of universal eye screening using red reflex testing in well-baby clinics and maternity units, compared with maternity unit screening only *(121)*. As universal screening was not compared with no screening, the study did not provide data relevant to this evidence summary.

Table 3.53 Main resource requirements for universal newborn screening for abnormalities of the eye

Resource	Description
**Staff**	• Neonatologists/paediatricians, midwives/nurses
**Training**	• Practice-based training in torch examination for newborn health workers; no specialist training is required for newborn torch light eye examination<br>• Training to perform red reflex testing is provided by a paediatric ophthalmologist *(122)*
**Supplies**	• Information (written and/or pictorial, e.g. leaflets) for parents<br>• Batteries (replaceable or rechargeable dry-cell alkaline batteries, depending on the specific device; Arclight™ can be charged via solar or USB)[38]
**Equipment and infrastructure**	• Medical torch/flashlight/penlight = US$ 2.60 *(123)*<br>• Ophthalmoscope set = US$ 51.41 *(123)*; US$ 7.50 for Arclight™ (cost for bulk purchase) *(124)*<br>• Darkened room/space
**Time**	• Screening can be performed prior to discharge after birth and/or as part of subsequent routine postnatal visits<br>• *Time to perform:* 1–2 minutes
**Supervision and monitoring**	• Referral to a paediatric ophthalmologist is required where there are abnormal results; otherwise, same as for usual care

Equity

No direct evidence was identified on the impact on health equity of universal screening for abnormalities of the eye for term, healthy newborns. Universal eye screening among newborns could help to address health inequalities by improving detection of eye abnormalities in low- to middle-income settings, where childhood blindness is more prevalent *(125)*. However, the ultimate impact on health equity is likely to vary by the capacity of services to provide appropriate referral, as well as access to the necessary treatment.

Additional considerations

Evidence from a systematic review suggests that girls with bilateral cataract are less likely to access cataract surgery than boys in some regions, particularly in South Asia and East Asia, and the Pacific *(126)*. There is also variation in age and stage at presentation of retinoblastoma by country-income groups, with children in HICs presenting at lower median age and with lower rates of advanced stage VI disease compared with LMICs *(127)*. Early screening, identification and counselling will raise awareness among parents and may help to increase access to services.

Acceptability

See Box 3.8 in section 3.B.1: Newborn assessment.

Additional considerations

A pilot study in the United Republic of Tanzania showed that including an eye care module in the child health training programme for 60 primary child health-care workers on red reflex testing using the Arclight™ increased health workers' knowledge, was acceptable and that they were able to implement it within routine care *(128)*.

In another study in the United Republic of Tanzania, 24 nurses were trained to screen the eyes of children aged 0–5 years using Arclight™ red reflex testing and reported that that parents were happy and willing to let their children be screened *(129)*.

38 For more information, see the Arclight Project: https://med.st-andrews.ac.uk/arclight/

Feasibility

See Box 3.9 in section 3.B.1: Newborn assessment.

Additional considerations

A study in the United Republic of Tanzania assessed the feasibility of Arclight red reflex screening for children aged 0–5 years in primary health-care settings by trained nurses *(129)*. Most nurses reported they could differentiate a normal from an abnormal red reflex very easily or easily, that examining newborns less than four weeks old was more difficult and that the examination took less than 3 minutes. However, screening interfered with their day-to-day activities because of staff shortages, large numbers of children and multiple responsibilities. Care is needed in extrapolating some these findings to newborns who are more difficult to screen using the red reflex test than older children.

Table 3.54 Summary of judgements: Universal eye screening compared with no screening

Domain	Judgement
Desirable effects	Small
Undesirable effects	Trivial
Certainty of the evidence	Very low
Values	Probably no important uncertainty or variability
Balance of effects	Probably favours universal eye screening
Resources required	Negligible costs or savings
Certainty of the evidence on required resources	No included studies
Cost-effectiveness	Don't know
Equity	Varies
Acceptability	Probably yes
Feasibility	Probably yes

B.1.3 Universal screening for hearing impairment

RECOMMENDATION 27

Universal newborn hearing screening (UNHS) with otoacoustic emissions (OAE) or automated auditory brainstem response (AABR) is recommended for early identification of permanent bilateral hearing loss (PBHL). UNHS should be accompanied by diagnostic and management services for children identified with hearing loss. *(Recommended)*

Remarks

- In making this recommendation, regardless of gestation or risk factors, the Guideline Development Group agreed that although evidence on the effects originated from high-income countries, the evidence on resources, cost effectiveness, values, equity, acceptability and feasibility demonstrate that UNHS could be successfully implemented in low- and middle-income countries.
- PBHL is defined as bilateral permanent conductive or sensorineural hearing loss of 35 dB or greater in the better ear.
- If UNHS indicates possible PBHL, a follow-up definitive test must be done as soon as possible after screening. This involves testing by an audiologist with a more detailed diagnostic auditory brainstem response in a highly-controlled environment. It takes 30–60 minutes to complete the diagnostic test.
- The principles for screening programmes *(87)* must be implemented throughout UNHS introduction and scale-up. In settings where principles for screening are not met, implementation of universal screening may be considered unethical.
- Parents and caregivers of all children should be informed about age-appropriate hearing and language development and communication skills regardless of the screening results.

Summary of evidence and considerations

Effects of the interventions (EB Table B.1.3)

Evidence was derived from a systematic review on universal newborn hearing screening programmes to detect children with PBHL *(130)*. The review included 30 non-randomized studies, of which five studies (1 023 610 newborns) that reported comparative effects of universal newborn hearing screening (UNHS) versus no UNHS were included in this evidence summary. Studies were conducted in Australia (1), the Netherlands (1), the United Kingdom (1) and the USA (2).

The screening tests used in the intervention group were OAE, AABR, or both. Where described, screening was performed in the first 24–48 hours after birth or by 2 weeks of age. The comparison group received either no screening at any time, no screening in the first eight months after birth followed by distraction screening at eight months or later, or risk factor screening. "Any hearing loss requiring amplification" was used to define PBHL in one study. In the other four studies, threshold levels in the better ear of > 40 dB, > 35 dB or > 25 dB were used.

Four studies (1 171 737 newborns) were large population-based studies of UNHS programmes. Those failing the screening received a definitive hearing assessment from an audiologist promptly following screening. Children with PBHL were followed up to ascertain developmental outcomes. The remaining study (63 children) used a retrospective cohort design, and reviewed medical records of children with PBHL at 5 years of age to ascertain if they had received newborn screening, definitive hearing testing, amplification devices (hearing aids) and the timing of these procedures.

Comparison: UNHS compared with no screening or selective screening (no UNHS)

Newborn/infant outcomes

Severe neonatal/infant morbidity: Low-certainty evidence suggests UNHS may make little or no difference to the proportion of infants identified with PBHL when compared with no UNHS (3 studies, 1 023 497 newborns; RR 1.01, 95% CI 0.89 to 1.14). Low-certainty evidence suggests UNHS may improve the proportion of infants identified with PBHL early (before 9 months of age) when compared with no

UNHS (1 study, 156 733 newborns; RR 3.28, 95% CI 1.84 to 5.85). It is uncertain whether UNHS in newborns with bilateral hearing loss improves the mean age of identification of PBHL when compared with no UNHS (very low-certainty evidence).

Neurodevelopment: It is uncertain whether UNHS in newborns with PBHL has any effect on mean receptive language at 3–8 years of age (z score and development quotient), mean expressive language at 3–8 years of age (z score and development quotient), mean literacy at 5–11 years of age (z score) and mean literacy at 13–19 years of age (z score) when compared with no UNHS (very low-certainty evidence).

Adverse effects were not reported in the systematic review.

Maternal outcomes
Experience of postnatal care and *cost* were not reported in the systematic review.

Additional considerations

Evidence reviews indicated that infants with PBHL identified through UNHS have significantly earlier referral, diagnosis and treatment, and improve communication outcomes than those identified through means other than UNHS *(131, 132)*.

A systematic review of 32 study populations in HICs (1 799 863 screened infants) found high sensitivity, specificity, positive and negative predictive values for AABR and OAE, used alone or in combination. Sensitivity and specificity ranged from 89% to 100% and from 92% to 100%, respectively, positive predictive values ranged from 2% to 84%, with all negative predictive values 100% *(115)*.

Values

See Box 3.7 in section 3.B.1: Newborn assessment.

Resources

WHO conservatively estimated a return on investment from newborn hearing screening in lower-middle and high-income settings. Results, based on actual costs, estimated that in a lower-middle-income setting (taken as an example) there would be a possible return of 1.67 International dollars for every one dollar invested in newborn hearing screening *(116)*.

A systematic review of economic evaluations identified 12 economic evaluations *(133)* estimating the incremental cost per detected true positive case or incremental cost per DALY averted of UNHS compared with selective screening or no screening. Eleven studies used a model-based approach. The only within-study evaluation (moderate quality) compared targeted and universal hearing screening in community-based or hospital-based programme in Nigeria between 2005 and 2006 *(134)*. The analysis found that cost per baby screened under selective screening or cost per child detected with permanent congenital and early-onset hearing loss (PCEHL) were higher both in the hospital and in the community compared with UNHS. The incremental cost per true positive case detected of community-selective screening was US$ 1221 compared with community-UNHS *(133)*. If a no screening strategy with $0 cost and nil effect was considered (as reference), the community-based UNHS would have an incremental cost-effectiveness ratio (ICER) of US$ 26 809 *(133)*.

In different studies the cost of newborn screening using OAE ranges from US$ 1.60 to US$ 2.20 per baby *(135)* and the cost for AABR ranges from US$ 2.30 to approximately US$ 4.00 per baby *(135, 136)*.

Additional considerations

The systematic review of economic evaluations described above identified eleven modelling evaluations that concluded UNHS was cost-effective compared with no screening or selective screening in Australia, China, Chinese Taipei, Germany, Philippines, Spain, the United Kingdom and the USA *(133)*.

One additional modelling evaluation published after the publication of the above-mentioned systematic review found that a two-stage screening protocol (OAE-AABR maternity) was cost-effective for implementation in Albania *(137)*.

Table 3.55 Main resource requirements for universal newborn hearing screening (UNHS)[39]

Resource	Description
**Staff**	• Doctors/midwives/nurses<br>• Specialist staff are not required for screening due to the simplicity of operating the equipment and automation of results
**Training**	• Training to administer screening tests (OAE and/or AABR)<br>• One or both screening tests may be used depending on local protocols
**Supplies**	• Information (written and/or pictorial, e.g. leaflets) for parents<br>• For OAE screening:<br>– Ear tips = indicative cost US$ 160 for a pack of 250 reusable pieces, equating to around US$ 0.10 per baby *(134)*<br>• For AABR screening:<br>– Disposable earphones and sensors/electrodes = US$ 12 per baby *(134)*<br>• Referral letters where required (paper, printing)
**Equipment and infrastructure**	• OAE device and software including small outer-ear probe with earphone and microphone (US$ 4127–5777) *(135)*<br>• AABR device and software including ear couplers (US$ 5503–7153) *(135)*<br>• Computer or other device compatible with testing software to assess results<br>• A quiet, calm space to carry out the test<br>• Access to electricity for computer use, printing and charging the screening device battery
**Time**	• Time required for each screening test is approximately 20 minutes, including settling the baby, performing the test, recording the results and discussing the results with parents; screening may take longer if the baby is restless or has difficulty settling *(138)*<br>• The test itself takes approximately 5 minutes<br>• Servicing and recalibration of screening equipment according to manufacturer specifications<br>• Screening is usually completed prior to discharge or can be completed at a postnatal visit
**Supervision and monitoring**	• Referral to audiology services is required for newborns who do not meet the pass mark for hearing screening; otherwise, same as for usual care

AABR: automated auditory brainstem response; OAE: otoacoustic emissions

Equity

No direct evidence was identified on the impact on health equity of UNHS for term, healthy newborns. UNHS could help to address health inequalities by improving detection of auditory problems and prompting early intervention from families and health workers to minimize developmental disadvantage in low- to middle-income settings, where childhood hearing impairment is more prevalent *(139)*. However, the ultimate impact on health equity is likely depend on access to the necessary definitive diagnostic test, and provision of appropriate referral and treatment for those who screen positive.

Additional considerations

Some studies have reported that risk factors for infant hearing loss or impairment include low maternal education *(140)*, minority race *(141)* and living in poorer urban neighbourhoods *(141)*. Low socioeconomic status has also shown to be related to non-participation in newborn hearing screening programmes *(142)*.

Acceptability

See Box 3.8 in section 3.B.1: Newborn assessment.

Additional considerations

Several individual qualitative research studies exploring parental experiences of UNHS programmes indicate that parents may experience anxiety related to newborn hearing screening, often feel unprepared for the positive and/or inconclusive results of a newborn hearing test and may need additional support and information from knowledgeable, sensitive health workers *(143–145)*. A cross-sectional observational study among 48 mothers in Nigeria

39 These costs relate exclusively to screening; additional costs would be incurred for any diagnostic testing and treatment, where required.

reported poor awareness of newborn hearing screening. However, the majority of mothers (92%) expressed a willingness to accept hearing screening for their child. Willingness to accept screening was associated with socioeconomic status and maternal education *(146)*.

Feasibility

See Box 3.9 in section 3.B.1: Newborn assessment.

Additional considerations

Implementation of selective hearing screening programmes (e.g. risk factor and opportunistic screening) requires training of health workers in the important risk factors for hearing loss and the behaviour of babies who have hearing loss. These programmes also require a personal level of awareness from busy health workers on a day-to-day basis.

A national evaluation of the UNHS programme in the USA from 2005 to 2006 *(147)* found that the screening programme was effective but barriers to follow-up including a lack of service-system capacity, lack of health workers knowledge and information gaps limited overall success in a number of states. Low follow-up presents a challenge to UNHS programmes, particularly in low- to middle-income settings.

Table 3.56 Summary of judgements: UNHS compared with no screening or selective screening (no UNHS)

Domain	Judgement
Desirable effects	Small
Undesirable effects	Don't know
Certainty of the evidence	Low
Values	Probably no important uncertainty or variability
Balance of effects	Probably favours UNHS
Resources required	Large costs
Certainty of the evidence on required resources	Moderate
Cost-effectiveness	Probably favours UNHS
Equity	Varies
Acceptability	Probably yes
Feasibility	Varies

B.1.4 Universal screening for neonatal hyperbilirubinaemia

RECOMMENDATION 28

Universal screening for neonatal hyperbilirubinaemia by transcutaneous bilirubinometer (TcB) is recommended at health facility discharge. *(Recommended)*

RECOMMENDATION 29

There is insufficient evidence to recommend for or against universal screening by total serum bilirubin (TSB) at health facility discharge. *(No recommendation issued)*

Remarks

- The postnatal age for universal TcB screening at discharge should be guided by the timing of health facility discharge. The Guideline Development Group (GDG) considered that all healthy newborns should receive facility care for at least 24 hours after birth. The GDG considered that transcutaneous bilirubin screening at discharge should be followed up with serum bilirubin measurement, appropriate treatment, and follow-up as indicated by age-appropriate nomograms.[40]
- The GDG emphasized that the existing WHO recommendations on routine assessment of the newborn for danger signs, including jaundice and yellow palms and soles, still apply (See Recommendation 25 in this guideline). During health facility stay, clinicians should ensure that all newborns are routinely monitored for the development of jaundice and that serum bilirubin should be measured in those at risk; that is, in all babies if jaundice appears on day 1, and in all babies if palms and soles are yellow at any age *(148)*.
- The GDG decided not to formulate a recommendation on universal screening for neonatal hyperbilirubinaemia using TSB due to the lack of evidence comparing universal TSB with universal TcB measurement. There was uncertainty around the benefits of universal TSB screening compared with clinical screening for important clinical outcomes. Additionally, the GDG considered that the costs were large, and feasibility and acceptability varied markedly.

Summary of evidence and considerations: Universal TcB screening for neonatal hyperbilirubinaemia

Effects of the interventions (EB Table B.1.4a)

Evidence was derived from a systematic review of universal screening for hyperbilirubinaemia in term healthy newborns at discharge *(149)*. The systematic review included five studies that were included in this evidence summary (377 814 newborns), of which four studies were conducted in the USA and one in South Africa.

One study was an RCT (1858 newborns) and four studies used a retrospective cohort design (375 956 newborns). All studies included late preterm or term newborns. Universal TcB screening was done for all newborns being discharged from the well-baby nurseries, irrespective of whether they had a clinical risk factor for the development of hyperbilirubinaemia. Most studies conducted TcB screening in hospital prior to discharge and on the day of discharge (24–96 hours after birth depending on mode of birth). In the comparison group, all studies used clinical screening (visual inspection and/or assessment of risk factors; 329 394 newborns) except one, which used “random” TcB (285 newborns). In the TcB group, confirmatory TSB was done in all studies, but the protocol varied according to risk status.

40 A nomogram is a chart that provides hour-specific TcB/TSB values. It helps to determine when to obtain serum bilirubin and decide the need for phototherapy or exchange transfusion based on the infant’s postnatal age and clinical risk factors.

Comparison: Universal screening for identification of neonatal hyperbilirubinaemia by TcB at discharge compared with clinical screening (visual inspection and/or assessment of risk factors), followed by TcB or TSB if required

Newborn/infant outcomes

Severe neonatal morbidity

Severe hyperbilirubinaemia

- Evidence from RCTs: Low-certainty evidence suggests universal screening by TcB for hyperbilirubinaemia may lead to a reduction in proportion of newborns with severe hyperbilirubinaemia when compared with clinical screening (1 trial, 1858 newborns; RR 0.27, 95% CI 0.08 to 0.97).
- Evidence from non-RCTs: Low-certainty evidence suggests universal screening by TcB for hyperbilirubinaemia may lead to a reduction in the proportion of newborns with severe hyperbilirubinaemia when compared with clinical screening (1 study, 358 086 newborns; RR 0.25, 95% CI 0.12 to 0.52).

Jaundice requiring exchange transfusion

- Evidence from RCTs: It is uncertain whether universal screening by TcB for hyperbilirubinaemia has any effect on the proportion of newborns with jaundice requiring exchange transfusion when compared with clinical screening (very low-certainty evidence).
- Evidence from non-RCTs: Low-certainty evidence suggests universal screening by TcB for hyperbilirubinaemia may reduce the proportion of newborns with jaundice requiring exchange transfusion when compared with clinical screening (1 study, 358 086 newborns; OR 0.28, 95% CI 0.19 to 0.42).

Kernicterus/BIND

- It is uncertain whether universal screening by TcB for hyperbilirubinaemia has any effect on the proportion of cases of BIND/kernicterus when compared with clinical screening (RCT; very low-certainty evidence).

Health service use

Readmission for jaundice

- Evidence from RCTs: Moderate-certainty evidence suggests universal screening by TcB for hyperbilirubinaemia probably reduces readmission for jaundice when compared with clinical screening (1 trial, 1858 newborns; OR 0.24, 95% CI 0.13 to 0.46).
- Evidence from non-RCTs: It is uncertain whether universal screening by TcB for hyperbilirubinaemia has any effect on readmission for jaundice when compared with clinical screening (very low-certainty evidence).

Neonatal mortality, neurodevelopment and *adverse effects* were not reported in the systematic review.

Maternal outcomes

Experience of postnatal care and *cost* were not reported in the systematic review.

Additional considerations

One before-and-after study in a well-baby nursery (28 908 newborns) identified as part of the systematic review above compared universal TcB screening (daily TcB in hospital and post discharge in the community) with clinical screening (visual inspection by a public health nurse). The study reported that universal TcB screening decreased severe hyperbilirubinaemia (OR 0.45, 95% CI 0.31 to 0.65) and readmission for jaundice (OR 0.91, 95% CI 0.81 to 1.04) in healthy newborns ≥ 35 weeks' gestation, while the mean length of pre-discharge hospital stay was unchanged (40.8±22.3 hours in the universal TcB screening group compared with 40.3±21.5 hours in the visual inspection group; MD 0.5 higher, 95% CI 0 to 1 higher) *(150)*.

There are concerns that TcB screening may overestimate TSB levels in newborns with dark skin colour/tone *(151, 152)*, but evidence in this regard is conflicting *(153)*. The same applies to factors such as gestational age, postnatal age and TSB levels.

Values

See Box 3.7 in section 3.B.1: Newborn assessment.

Resources

No economic evaluations of universal TcB screening compared with clinical screening in term newborns without complications were identified.

Additional considerations

In a before-and-after study evaluating the implementation of universal TcB screening in a tertiary centre in Canada, McLean and colleagues estimated the cost per TcB screen to be Can$ 3.54 and Can$ 3.76 in hospital and community settings, respectively *(154)*.

Table 3.57 Main resource requirements of universal screening for neonatal hyperbilirubinaemia by TcB

Resource	Description
**Staff**	• Midwives/nurses
**Training**	• Practice-based training including training to use TcB in accordance with local protocols
**Supplies**	• Alcohol wipes for sterilization of meter probe<br>• Reusable probe tips (depending on device)<br>• Printed nomogram for plotting results (if not done electronically)
**Equipment and infrastructure**	• TcB = capital outlay approximately US$ 500–2000 each, plus calibration costs<br>• Access to electricity for charging meter via docking station<br>• Computer (if using electronic data transfer from TcB)
**Time**	• Daily operational check of TcB prior to use<br>• Calibration prior to each measurement (depending on device)<br>• Time to perform test estimated to be 2 minutes; results are instantaneous
**Supervision and monitoring**	• Ongoing device maintenance and calibration as per manufacturer specifications

Equity

No direct evidence was identified on the impact on health equity of universal screening for hyperbilirubinaemia by TcB among term newborns. Universal TcB screening of newborns could help to improve detection of neonatal hyperbilirubinaemia and prompt early intervention to prevent severe hyperbilirubinaemia in LMICs, where jaundice-related death and disability is more prevalent *(155)*. None of the included studies assessed TcB in community settings. Given pre-discharge TcB screening (and follow-up testing and management, where required) would be equally accessible to all facility-born newborns, it is anticipated that this intervention would probably have no impact on health equity.

Acceptability

See Box 3.8 in section 3.B.1: Newborn assessment.

Additional considerations

The authors of a qualitative study on parents' decision-making in newborn screening *(156)* found that parents supported newborn screening for treatable disorders but suggested optional screening for other disorders. They also suggested that the variable influences on parents' decision-making could provide critical perspectives and help screening programmes to address parents' preferences and meet relevant information needs, if parents were included in the decision-making regarding screening policies.

Feasibility

See Box 3.9 in section 3.B.1: Newborn assessment.

Additional considerations

Indirect evidence from a qualitative evidence synthesis exploring provision and uptake of routine antenatal services *(80)* suggests the initial cost and maintenance of screening equipment as well as the lack of suitably trained health workers may be prohibitive factors in some resource-limited settings (high confidence in the evidence).

As TcBs are portable, easy to use and generate instantaneous results *(157)*, universal screening with this method was considered probably feasible, where funding for capital outlay is available.

Table 3.58 Summary of judgements: Universal TcB compared with clinical screening

Domain	Judgement
Desirable effects	Large
Undesirable effects	Don't know
Certainty of the evidence	Very low
Values	Probably no important uncertainty or variability
Balance of effects	Probably favours universal TcB
Resources required	Moderate costs
Certainty of the evidence on required resources	No included studies
Cost-effectiveness	Don't know
Equity	Probably no impact
Acceptability	Probably yes
Feasibility	Probably yes

Summary of evidence and considerations: Universal TSB screening for neonatal hyperbilirubinaemia

Effects of the interventions (EB Table B.1.4b)

Evidence was derived from a systematic review of universal screening for hyperbilirubinaemia in term healthy newborns at discharge *(149)*. The review included two retrospective cohort studies and one observational study (490 426 newborns), all conducted in the USA. All studies included newborns with gestational age ≥35 weeks (490 426 newborns). All studies evaluated universal TSB screening compared with clinical screening (visual inspection and/or assessment of risk factors), but the intervention and comparison groups varied slightly across studies. Risk factors were variably defined, as were definitions of severe hyperbilirubinaemia and protocols for post-discharge follow-up.

Comparison: Universal screening of TSB before discharge compared with clinical screening (visual inspection and/or risk factor assessment)

Newborn/infant outcomes

Severe neonatal morbidity: It is uncertain whether universal pre-discharge TSB screening for neonatal hyperbilirubinaemia has any effect on the number of neonates with severe hyperbilirubinaemia or jaundice requiring exchange transfusion when compared with clinical screening.

Health service use: It is uncertain whether universal pre-discharge TSB screening for neonatal hyperbilirubinaemia has any effect on readmissions for jaundice when compared with clinical screening.

Neonatal mortality, neurodevelopment and *adverse effects of screening* were not reported in the systematic review.

Maternal outcomes

Experience of postnatal care and *cost* were not reported in the systematic review.

Values

See Box 3.7 in section 3.B.1: Newborn assessment.

Resources

No direct economic evaluations were identified on universal TSB screening for neonatal hyperbilirubinaemia compared with clinical screening.

Additional considerations

Estimated costs per TSB screen performed in health facilities in HICs varied. McLean and colleagues estimated the cost per TSB screen to be Can$ 15.82 in hospital settings, and Can$ 50.21 and Can$ 65.03 in urban and rural community settings, respectively *(154)*. No information on the costs of TSB screening in LMICs was identified.

A 2012 modelling study evaluated the incremental cost-effectiveness of a system-based approach involving universal TSB screening and the management of neonatal jaundice and prevention of kernicterus in infants > 35 weeks' gestation. It considered the traditional practice of visual inspection and selective TSB screening, and found that increased costs associated with universal TSB screening were partially offset by reduced emergency room visits, readmissions, and cases of kernicterus *(158)*.

Equity

No direct evidence was identified on the impact on health equity of universal screening for neonatal hyperbilirubinaemia by TSB before discharge from health facilities among term newborns. Universal TSB screening of newborns could improve detection of neonatal hyperbilirubinaemia and prompt early intervention to prevent severe hyperbilirubinaemia in

Table 3.59 Main resource requirements for universal screening for neonatal hyperbilirubinaemia by TSB

Resource	Description
**Staff**	• Midwives/nurses
**Training**	• Practice-based training including training to perform newborn heel prick test
**Supplies**	• Heel warmers<br>• Gloves<br>• Alcohol wipes<br>• Lancets and bullets<br>• Filter paper/cards<br>• Post-injection plasters/adhesive bandages<br>• Estimated cost per TSB screen in health facility = Can$ 15.82 in hospital setting, Can$ 50.21 and Can$ 65.03 in urban and rural community settings, respectively *(154)*<br>• Cost per newborn for universal TSB screening = Can$ 176 *(158)*
**Equipment and infrastructure**	• On-site pathology facility or access to laboratory services including blood/chemistry analyser
**Time**	• Time to perform test estimated to be 2–5 minutes<br>• Laboratory time to process results varies by facility from less than 30 minutes to several hours
**Supervision and monitoring**	• Accurate identification of serum bilirubin levels and appropriate management as per local hyperbilirubinaemia protocols, where required

LMICs, where jaundice-related death and disability is more prevalent *(155)*, but more evidence is needed. The PICO question guiding this evidence summary was expressly concerned with universal TSB screening before discharge from health facilities. Therefore, given pre-discharge TSB screening (and follow-up testing and management, where required) would be equally accessible to all facility-born newborns, it is anticipated that this intervention would probably have no impact on health equity.

Acceptability

See Box 3.8 in section 3.B.1: Newborn assessment.

Additional considerations

TSB screening requires a blood draw via heel prick, which is an invasive, painful procedure for newborns that may not be acceptable to parents. In settings where TSB can be included in a routine metabolic screen, thus not requiring an additional standalone heel prick, parents may find universal TSB more acceptable.

Feasibility

See Box 3.9 in section 3.B.1: Newborn assessment.

Additional considerations

TSB is not available in all facilities, and capacity to procure TSB analysis via external laboratories varies across facilities and settings. Where TSB can be included in a routine metabolic screen, it may be more feasible.

Table 3.60 Summary of judgements: Universal TSB compared with clinical screening

Domain	Judgement
Desirable effects	Don't know
Undesirable effects	Don't know
Certainty of the evidence	Very low
Values	Probably no important uncertainty or variability
Balance of effects	Don't know
Resources required	Large costs
Certainty of the evidence on required resources	No included studies
Cost-effectiveness	Don't know
Equity	Probably no impact
Acceptability	Varies
Feasibility	Varies

B.2 PREVENTIVE MEASURES

Background

The GDG considered the evidence and other relevant information to inform recommendations on the prevention of the following adverse outcomes during the neonatal period and/or infancy.

Hypothermia and its sequelae

Neonatal hypothermia is a common condition, affecting between 32% and 85% of hospital-born newborns *(159)*. The newborn's first bath is intended to eliminate blood and meconium from the skin, leaving the vernix intact *(160)*. However, bathing can be a stressful procedure for a newborn, and early bathing can trigger hypothermia and its related consequences, including hypoglycaemia, hypoxia and pulmonary haemorrhage *(161)*. Bathing of neonates is carried out using various methods including tub bathing, sponge bathing, swaddled bathing and bathing under running water.

Atopic dermatitis and other skin conditions

The skin of a neonate is a dynamic and complex organ that is under the process of maturation. Neonates and infants have thin, highly permeable skin with a larger body surface area. Topical emollients may protect the stratum corneum, increase its hydration, decrease water loss across the skin and can enhance epidermal barrier function *(162)*. These emollients include creams, ointments, lotions, oils, gels, sprays and emulsions, and are used for bathing, cleansing or as leave-on emollients. While applying emollients prior to the development of atopic dermatitis may help in primary prevention of the condition, the plausible harm is the potential destruction of the acid mantle, which is key to epidermis function *(163)*. Further, absorption of the excipients of emollients may result in contact sensitivity and epidermal injury *(163)*.

Neonatal infection

Severe neonatal infection is a leading cause of global neonatal mortality *(164)*. During the first days after birth, the healing umbilical cord stump is a possible entry point for infection, including through colonization of the stump with potentially pathogenic microorganisms *(165)*. In LMICs, traditional practices to seal the umbilical cord stump involve the application of potentially harmful substances to the area (e.g. dirt, dust, clay, mud and animal dung) *(166)*, which further increase the risk of systematic infection and tetanus. Chlorhexidine is a broad-spectrum antiseptic that is applied topically to the umbilical cord stump and base for the prevention of neonatal infection.

Sudden infant death syndrome (SIDS)

SIDS is defined as "the sudden unexpected death of an infant less than 1 year of age, with onset of the fatal episode apparently occurring during sleep, that remains unexplained after a thorough investigation, including performance of a complete autopsy and review of the circumstances of death and the clinical history" *(167)*. The umbrella terms "sudden unexpected death in infancy" (SUDI) and "sudden unexpected infant death" (SUID) refer to all unexplained deaths in infancy, as well as deaths due to known causes (e.g. suffocation, malformations, arrhythmias) *(168)*. According to the triple risk model *(169)*, an infant is susceptible to SIDS where all of three independent factors are present: (i) exogenous stressors (e.g. prone position, overheating, infection, head covering); (ii) critical development period in homeostatic control; and (iii) infant vulnerability (e.g. due to prematurity, low birthweight or nicotine exposure). Prone (lying with chest down or back up) sleeping position is considered one such exogenous stressor, which may disrupt infant cardiovascular functioning, increasing the risk of hypoxia, bradycardia, hypotension, metabolic acidosis, gasping and death *(168)*.

Box 3.10 Values

Findings from a qualitative evidence synthesis exploring what women want from postnatal care *(21)* indicate that women want a positive experience in which they are able to adapt to their new self-identity and develop a sense of confidence and competence as a mother. They also want to adjust to changes in their intimate and family relationships (including their relationship to their baby), navigate ordinary physical and emotional challenges, and experience the dynamic achievement of personal growth as they adjust to their new normal, both as parents and as individuals in their own cultural context. Findings from the same review also indicate that women tend to prioritize the needs of their baby and are highly likely to value any strategy that fosters infant development and enhances breastfeeding and/or their baby's general well-being (high confidence in the evidence).

In addition to the GDG recommendations on the above, this section of the guideline includes one recommendation that has been integrated from WHO guidelines on immunization for prevention of infections that is relevant to routine postnatal care. Guidance around specific newborn immunization is provided in Box 3.11 (section 3.2.5), based on existing WHO position papers.

B.2.1 Timing of first bath to prevent hypothermia and its sequelae

RECOMMENDATION 30

The first bath of a term, healthy newborn should be delayed for at least 24 hours after birth. *(Recommended)*

Remarks

- The Guideline Development Group (GDG) noted that there is no evidence to support an early first bath after birth for any special reason, such as meconium staining or for preventing the risk of transmission of infection from the mother.
- The GDG suggested that all measures should be taken to minimize heat loss during bathing, which include maintaining a neutral thermal environment, immediate drying, appropriate clothing of the newborn for the ambient temperature (this means 1–2 layers of clothes more than adults and use of hats/caps), and allowing the mother and baby to remain together at all times.

Summary of evidence and considerations

Effects of the interventions (EB Table B.2.1)

Evidence was derived from a systematic review of the timing of first baths in term, healthy newborns *(170)*, including 13 studies with 37 995 newborns. The systematic review included nine before-and-after intervention studies, two case-control studies, one non-randomized trial, and one cross-sectional study. Studies were conducted in Canada (1), Ethiopia (1), Nepal (1), Pakistan (1), the United Kingdom (1) and the USA (8).

Most of the studies considered early bathing in the pre-implementation phase as bathing received earlier than the cut-off time point that defined the delayed bath in the relevant setting. Two studies defined early bathing as at or before 24 hours from birth and delayed bathing as after 24 hours from birth. Nine studies defined early bathing as at or before 6 hours from birth; however, the definition for delayed bathing varied between the studies, from after 6 hours from birth to after 24 hours from birth. One study defined early bathing as at or before 12 hours and delayed bathing as after 12 hours, while one study classified the data on timing of first bath into six categories: > 24 hours, 12–23.9 hours, 6–11.9 hours, 3–5.9 hours, 12.9 hours and < 1 hour.

Comparison 1: Delayed first bath (after 24 hours) compared with early first bath (at or before 24 hours)

Newborn/infant outcomes

Neonatal/infant mortality: Low-certainty evidence suggests a delayed first bath (after 24 hours) may reduce infant mortality (all-cause) when compared with an early bath (at or before 24 hours) (1 study, 789 newborns; RR 0.46, 95% CI 0.28 to 0.76).

Short-term neonatal morbidity: Low-certainty evidence suggests that a delayed first bath (after 24 hours) may reduce neonatal hypothermia when compared with an early bath (at or before 24 hours) (1 study, 660 newborns; RR 0.50, 95% CI 0.28 to 0.88).

Breastfeeding status: It is uncertain whether a delayed first bath (after 24 hours) has any effect on exclusive breastfeeding at discharge when compared with an early bath (at or before 24 hours) (very low-certainty evidence).

Severe neonatal/infant morbidity was not reported in the systematic review.

Maternal outcomes

Experience of postnatal care was not reported in the systematic review.

Comparison 2: Delayed first bath (after 6 hours; i.e. at or after 9, 12 or 24 hours) compared with early first bath (at or before 6 hours).

Newborn/infant outcomes

Neonatal/infant mortality: It is uncertain whether a delayed first bath (after 6 hours) has any effect on neonatal mortality when compared with an early bath (at or before 6 hours) (very low-certainty evidence).

Short-term neonatal morbidity: Low-certainty evidence suggests a delayed first bath (after 6 hours; that is, at or after 9, 12 or 24 hours) may reduce neonatal hypothermia when compared with an early bath (at or before 6 hours) (4 studies, 2711 newborns; OR 0.47, 95% CI 0.36 to 0.61). Low-certainty evidence suggests a delayed first bath (after 6 hours; that is, after 12 or 24 hours) may reduce neonatal hypoglycaemia when compared with an early bath (at or before 6 hours) (3 studies, 2775 newborns; RR 0.39, 95% CI 0.23 to 0.66).

Breastfeeding status: Low-certainty evidence suggests a delayed first bath (after 6 hours; that is, after 9, 12 or 24 hours) may increase exclusive breastfeeding at discharge when compared with an early bath (at or before 6 hours) (6 studies, 6768 newborns; OR 1.20, 95% CI 1.08 to 1.34).

Severe neonatal/infant morbidity was not reported in the systematic review.

Maternal outcomes

Experience of postnatal care was not reported in the systematic review.

Values

See Box 3.10 in section 3.B.2: Preventive measures.

Resources

No economic evaluations of late first neonatal bathing were identified.

Equity

A before-and-after evaluation of Malawi's Community-Based Maternal and Newborn Care programme using household surveys indicated increases in the coverage of delayed bathing (defined as first bath at 6 or more hours after birth) at endline compared with baseline across wealth quartiles. A small but statistically significant increase in equity was observed between baseline and endline for

Table 3.61 Main resource requirements for delayed first bath compared with early first bath

Resource	Description
**Staff**	• Midwives/nurses, as for usual care
**Training**	• In-service training to implement the new facility protocol and provide the necessary information and support to parents and caregivers for delayed first neonatal bathing (e.g. counselling families, managing blood or meconium on newborns without bathing, bathing safety)<br>• Additional training, support and clinical champions, especially for settings in which early bathing is culturally normative and where staff may be resistant to change
**Supplies**	• Posters, flyers and information leaflets for staff<br>• Information (written and/or pictorial, e.g. leaflets) for parents<br>• Appropriate clothing for the baby according to ambient temperature (1–2 layers of clothing plus hats/caps)<br>• Bathing supplies including clean water, clean towels (as for usual care)
**Equipment and infrastructure**	• Same as for usual care
**Time**	• A policy of delayed bathing may mean that some babies' first bath will occur after discharge; in most cases the primary bath provider is therefore likely to be the mother, parents or a family member<br>• Additional time may be required before discharge to provide information and support to parents about first bath and bathing practices at home
**Supervision and monitoring**	• Ongoing chart audit by clinical leaders to monitor bathing times and address any barriers to implementation

delayed bathing *(171)*. Another community-based newborn care package in Nepal demonstrated an increase in coverage of delayed bathing (defined as first bath at 6 or more hours after birth) across wealth quartiles at endline compared with baseline, though there was no statistically significant increase in equity for this specific component of the care package *(172)*.

Interventions that improve newborn and infant outcomes, including rates of breastfeeding, could assist to address health equity. Delaying the timing of first neonatal bath is a simple intervention with no additional ongoing cost implications for facilities or families. Provided the necessary training and support is available to alter current practice, this intervention would probably increase health equity.

Acceptability

A qualitative evidence synthesis of women's experiences of postnatal care found no direct evidence relating to women's views on the timing of bathing for their babies *(28)*. Indirect evidence from this review suggests most women appreciate any advice, information or technique (e.g. a delayed first bath) that might enhance their baby's comfort and well-being (high confidence in the evidence). Findings also suggest that women are likely to value intimate moments in the development of mother–infant attachment, such as their baby's first bath (moderate confidence in the evidence).

Additional considerations

In one study included in the systematic review *(170)* assessing the effect of delaying newborn bathing for 24 hours, nurses felt more supported by their peers to delay the first bath by 24 hours during post-implementation surveys than before the intervention *(173)*. However, the acceptability among families of delayed bathing may vary across populations, particularly where early bathing is culturally normative *(174)*.

Feasibility

A qualitative evidence synthesis of women's experiences of postnatal care found no direct evidence relating to women's views on the feasibility of a delayed first bath as opposed to early bathing *(28)*. Low quality, indirect evidence from some LMICs indicates that women are aware of resource constraints, including inconsistent water supplies that might affect the timing of infant bathing (moderate confidence in the evidence). A qualitative evidence synthesis of health workers' experiences of postnatal care found no direct evidence relating to views on the feasibility of delayed first neonatal bathing in term newborns *(29)*. However, indirect evidence suggests that lack of personnel, resources and training may limit provision of information and counselling on delayed first neonatal bathing in term newborns in the postnatal period (moderate confidence in the evidence).

Additional considerations

In one study included in the systematic review *(173)* assessing the effect of delaying newborn bathing for 24 hours, more nurses reported feeling comfortable identifying the benefits of delayed bathing, its contraindications, and encouraging families and their nursing peers to delay the first bath by 24 hours during post-implementation surveys than before the intervention. There was no increase in workload.

Table 3.62 Summary of judgements: Delayed first bath compared with early first bath

Domain	Judgement
Desirable effects	Moderate
Undesirable effects	Trivial
Certainty of the evidence	Low
Values	Probably no important uncertainty or variability
Balance of effects	Probably favours delayed first bath
Resources required	Negligible costs or savings
Certainty of the evidence on required resources	No included studies
Cost-effectiveness	Don't know
Equity	Probably increased
Acceptability	Varies
Feasibility	Probably yes

B.2.2 Use of emollients for the prevention of skin conditions

RECOMMENDATION 31

Routine application of topical emollients in term, healthy newborns for the prevention of skin conditions is not recommended. *(Not recommended)*

Remarks

- By topical emollients, the recommendation refers to creams, ointments, lotions, oils, gels, sprays and emulsions for skin care, applied routinely to whole or part of the body, without additional massage.
- In making this recommendation, the Guideline Development Group (GDG) agreed there was insufficient evidence on the benefits and harms, if any, of routine application of topical emollients in term, healthy newborns for either preventing skin conditions (including atopic dermatitis, skin dryness and others) or atopic sensitization to allergens (food or inhalation).
- The recommendation is based on evidence from studies of term, healthy newborns. Evidence from studies conducted in high-risk populations, such as newborns with family history of allergic disease, preterm and small for gestational age newborns were not considered.
- The recommendation does not preclude further research on emollient use in term, healthy newborns given the lack of evidence on key neonatal outcomes.
- The studies included in the evidence base for this recommendation rarely reported harm. However, the GDG raised concerns about the potential risk of harm with certain types of emollients from pilot randomized controlled trials and observational studies in term newborns and adults, and in studies of preterm newborns and animals.

Summary of evidence and considerations

Effects of the interventions (EB Table B.2.2)

Evidence was derived from a systematic review of the effect of routine use of topical emollients in term, healthy newborns to improve neonatal outcomes *(175)*. The review included 12 RCTs (5215 newborns) of which five trials were considered in this evidence summary. Three trials included only term newborns (728 newborns) and two trials included newborns ≥ 35 weeks of gestation (2624 newborns).

Trials were conducted between 2006 and 2017 in Germany (1), Japan (2) and the United Kingdom (1). One trial was a multicountry trial conducted in Sweden and Norway. Trials compared various forms of emollients such as creams or nut, seed, and vegetable oils, with other emollients and/or no emollients in two-, three- and four-arm study designs. The newborns allocated to "emollient only" and control groups were considered for the analysis. Where stated, emollients were applied to the face, left forearm, or left thigh and abdomen, and application commenced at varying times from birth through to two weeks after birth. Duration and frequency of emollient use varied from four weeks to up to eight months, applied from two times per week to 2–3 times per day.

Comparison: Topical emollients compared with no intervention or skin care without emollients

Newborn/infant outcomes

Short-term neonatal/infant morbidity: Low-certainty evidence suggests skin care with topical emollients may have little or no effect on atopic dermatitis in term, healthy newborns when compared with no intervention or skin care without emollients (2 trials, 1408 newborns; RR 1.29, 95% CI 0.96 to 1.72). It is uncertain whether skin care with emollients has any effect on food allergies, allergic sensitization to food allergens, allergic sensitization to inhalation allergens, dryness or skin problems in healthy newborns when compared with no intervention or skin care without emollients (very low-certainty evidence).

Adverse effects: Only two trials reported provided information on adverse effects. One trial (115 newborns) reported that oils may impede the development of lamellar lipid structures of the

permeability barrier from birth. The other trial (2397 newborns) reported one slippage of the baby in the emollient group compared with none in the control.

Maternal outcomes

Maternal functioning/well-being was not reported in the systematic review.

Additional considerations

The systematic review included seven trials (894 newborns, one trial did not report the number of participants) including newborns at risk of atopic dermatitis, defined in most studies as having at least one first-degree relative with parent-reported or physician-diagnosed eczema, allergic rhinitis or asthma. The results suggest that the use of emollients reduces the risk of atopic dermatitis with little or no effect on food allergy, allergic sensitization with food allergens, allergic sensitization with inhaled allergens, skin dryness and skin problems. In one trial (1394 newborns), parents reported an increase in doctor-diagnosed skin infections. There was no difference in infant slippages within 1 hour of applying emollients.

Values

See Box 3.10 in section 3.B.2: Preventive measures.

Resources

No economic evaluations of routine skin care with topical emollients for term, healthy newborns were identified.

Additional considerations

One trial *(176)* evaluating emollient application during the first year for preventing atopic eczema in high-risk infants will report cost-effectiveness and cost-utility at 24 months.

Equity

No direct evidence was identified on the impact on health equity of routine skin care with topical emollients for term, healthy newborns. If families are expected to pay for skin care products, especially across the entire postnatal period, the intervention may decrease equity.

Acceptability

A qualitative evidence synthesis of women's experiences of postnatal care found no direct evidence relating to women's views on the use of emollients for their babies *(28)*. Indirect evidence from this review suggests that most women appreciate any advice, information and, where appropriate, treatment that might enhance their baby's comfort and well-being (high confidence in the evidence). However, in some LMIC contexts women may prefer to use traditional newborn care practices, including the use of specific oils, native plants and herbal compresses to address infant skin concerns (moderate confidence in the evidence).

Feasibility

A qualitative evidence synthesis of women's experiences of postnatal care found no direct evidence relating to women's views on the feasibility of using emollients for skin care in term newborns *(28)*. Indirect evidence from the same review indicates some women in LMICs may be less likely to use these products if they perceive that health facilities lack the resources to offer advice and

Table 3.63 Main resource requirements for topical emollients for skin care in term, healthy newborns

Resource	Description
**Staff**	• None required
**Training**	• None required
**Supplies**	• Suitable emollient product for newborn skin (e.g. plant-based oil or commercially available moisturizing cream); price varies by location and supplier
**Equipment and infrastructure**	• Access to soap and clean water for proper hand hygiene prior to application
**Time**	• Application frequency varies by product and specific regimen; generally daily application but may be up to several times per day<br>• Duration of the intervention varies, from four weeks to 12 months
**Supervision and monitoring**	• None required

support or if they believe that utilization will incur unnecessary additional costs (moderate confidence in the evidence).

A qualitative evidence synthesis of health workers' experiences of postnatal care found no direct evidence relating to views on the feasibility of using emollients for skin care in term newborns *(29)*. However, indirect evidence suggests that lack of personnel, resources and training may limit the provision of information and counselling on the use of emollients for skin care in term newborns during the postnatal period (moderate confidence in the evidence).

Additional considerations

A study included in the systematic review on effectiveness evaluated whether emollient use from birth is feasible for reducing the atopic dermatitis in high-risk neonates. All participating families found the intervention acceptable, and the majority reported applying emollients at least five days per week at six months *(162)*.

Emollient use is a simple intervention, but application to an infant's whole body daily or several times per week, across an extended period of time, may be demanding for some families.

Table 3.64 Summary of judgements: Topical emollients compared with no emollients

Domain	Judgement
Desirable effects	Trivial
Undesirable effects	Small
Certainty of the evidence	Very low
Values	Probably no important uncertainty or variability
Balance of effects	Probably favours no emollients
Resources required	Moderate costs
Certainty of the evidence on required resources	No included studies
Cost-effectiveness	Don't know
Equity	Varies
Acceptability	Probably yes
Feasibility	Probably yes

B.2.3 Application of chlorhexidine to the umbilical cord stump for the prevention of neonatal infection

RECOMMENDATION 32a

Clean, dry umbilical cord care is recommended. *(Recommended)*

RECOMMENDATION 32b

Daily application of 4% chlorhexidine (7.1% chlorhexidine digluconate aqueous solution or gel, delivering 4% chlorhexidine) to the umbilical cord stump in the first week after birth is recommended only in settings where harmful traditional substances (e.g. animal dung) are commonly used on the umbilical cord. *(Context-specific recommendation)*

Remarks

- The Guideline Development Group conclusions are based on moderate-certainty evidence about the effects on neonatal mortality of applying 4% chlorhexidine in the first week after birth in newborns with non-hygienic (harmful) cord care. In newborns with non-hygienic cord care, chlorhexidine reduced mortality. In newborns without non-hygienic cord care, chlorhexidine did not reduce mortality.
- The recommendation is based on studies from Asia and Africa with high proportions of home births, low birthweight infants and neonatal mortality, conducted primarily between 2000 and 2010. The prevalence of omphalitis has decreased since that time.

Summary of evidence and considerations

Effects of the interventions (EB Table B.2.3)

Evidence was derived from an individual patient data (IPD) meta-analysis on the efficacy and safety of umbilical cord cleansing with chlorhexidine in neonates *(177)*, including five trials (129 391 newborns), analysed as intention-to-treat (ITT). Four trials were cluster-RCTs (92 480 newborns) and one was an individual RCT (36 911 newborns). Trials were conducted in Bangladesh, Nepal, Pakistan, the United Republic of Tanzania and Zambia. The baseline neonatal mortality rate in three trial sites (Bangladesh, Nepal, Pakistan) was 30 or more per 1000 live births. The baseline mortality rate in the other two trial sites (the United Republic of Tanzania, Zambia) was 15 per 1000 live births. About 80% of births in three trial sites occurred at home (Bangladesh, Nepal, Pakistan), and less than 50% of births in the other two trial sites occurred at home (the United Republic of Tanzania, Zambia). Use of non-hygienic applications (defined as using harmful substances on the cord such as dirt, dust, clay, mud and animal dung) at any time during the study ranged from < 1% to 90% across the five trials.

Use of chlorhexidine 4% was compared with dry cord care and/or soap and water, a placebo liquid or intensive hand washing. Chlorhexidine was applied daily, using single and/or multiple applications via a soaked cotton ball or dropper bottle, for up to 14 days after birth, on pre-specified days after birth or until 3 days after cord separation. Chlorhexidine was applied by study staff, a volunteer health worker, traditional birth attendant, clinical staff and/or the mother.

Comparison: Routine application of chlorhexidine to the umbilical cord stump compared with dry cord care or usual care

Newborn/infant outcomes

Neonatal mortality: Moderate-certainty evidence suggests routine application of chlorhexidine to the umbilical cord stump probably makes little or no difference to neonatal mortality when compared with dry cord care or usual care (5 trials, 136 829 newborns; OR 0.90, 95% CI 0.78 to 1.04).

Subgroup analysis by the mortality rate reported in the trial is as follows.

- It is uncertain whether routine application of chlorhexidine to the umbilical cord stump has any effect on neonatal mortality in settings with neonatal mortality rates ≥ 30% when compared with dry cord care or usual care (very low-certainty evidence).

- High-certainty evidence suggests routine application of chlorhexidine to the umbilical cord stump has little or no effect on neonatal mortality in settings with neonatal mortality rates < 30% when compared with dry cord care or usual care (2 trials, 74 762 newborns; OR 0.99, 95% CI 0.79 to 1.25).

Subgroup analysis by place of birth is as follows.

- It is uncertain whether routine application of chlorhexidine to the umbilical cord stump has any effect on neonatal mortality in babies born at home when compared with dry cord care or usual care (very low-certainty evidence).
- Moderate-certainty evidence suggests routine application of chlorhexidine to the umbilical cord stump probably makes little or no difference to neonatal mortality in babies born in facilities when compared with dry cord care or usual care (5 trials, 50 644 newborns; OR 0.95, 95% CI 0.81 to 1.10).

Subgroup analysis by non-hygienic applications is as follows.

- Moderate-certainty evidence suggests routine application of chlorhexidine to the umbilical cord stump probably reduces neonatal mortality when compared with usual care that includes non-hygienic applications (5 trials, 27 817 newborns; OR 0.63, 95% CI 0.50 to 0.79).
- Moderate-certainty evidence suggests routine application of chlorhexidine to the umbilical cord stump probably makes little or no difference to neonatal mortality when compared with dry cord care or cord care that does not include non-hygienic applications (5 trials, 136 320 newborns; OR 0.89, 95% CI 0.77 to 1.03).

Neonatal mortality after 24 hours of age: Moderate-certainty evidence suggests routine application of chlorhexidine to the umbilical cord stump probably makes little or no difference to mortality in infants over 24 hours of age when compared with dry cord care or usual care (5 trials, 135 237 newborns; OR 0.91, 95% CI 0.82 to 1.02).

Severe neonatal/infant morbidity:[41] Moderate-certainty evidence suggests routine application of chlorhexidine to the umbilical cord stump probably reduces the risk of moderate omphalitis when compared with dry cord care or usual care (5 trials, 137 942 newborns; OR 0.77, 95% CI 0.71 to 0.83). Low-certainty evidence suggests routine application of chlorhexidine to the umbilical cord stump may reduce the risk of severe omphalitis when compared with dry cord care or usual care (5 trials, 137 942 newborns; OR 0.55, 95% CI 0.39 to 0.76). Low-certainty evidence suggests routine application of chlorhexidine to the umbilical cord stump may have little or no effect on any possible serious bacterial infection (PSBI) when compared with dry cord care or usual care (5 trials, 137 942 newborns; OR 0.91, 95% CI 0.76 to 1.10). Low-certainty evidence suggests routine application of chlorhexidine to the umbilical cord stump may have little or no effect on more specific PSBI when compared with dry cord care or usual care (5 trials, 137 942 newborns; OR 0.91, 95% CI 0.75 to 1.11). Moderate-certainty evidence suggests routine application of chlorhexidine to the umbilical cord stump probably makes little or no difference to more severe PSBI (5 trials, 137 942 newborns; OR 0.93, 95% CI 0.83 to 1.10).

The IPD meta-analysis did not report on *health service use*. Only narrative information on the adverse effects of chlorhexidine was available, which is summarized below under Additional considerations.

Maternal outcomes

The IPD meta-analysis did not report on *experience of postnatal care*.

Additional considerations

The five studies were reviewed by the IPD authors for data on adverse effects. One study did not state whether adverse effects were assessed. One study reported eight cases of mild local skin irritation and one case where the chlorhexidine was accidently applied to the conjunctiva, resulting in mild ocular irritation. No newborns in the other three trials were reported to have adverse effects.

41 Data quality precluded the analysis of any omphalitis; over 30% of infants were reported to have omphalitis or an infection around the umbilical cord stump.

In 2019, the WHO released an alert regarding serious eye injury as a result of chlorhexidine misadministration, with reports of chlorhexidine being mistakenly applied to the eye (as eye drops or ointment) *(178)*. The alert urges all stakeholders involved in umbilical cord care to take steps to ensure correct use and administration of chlorhexidine. Reproductive health programmes and regulators were instructed to ensure clear and unique labelling of chlorhexidine products, and to provide parents and other caregivers with detailed, culturally appropriate written materials and counselling on chlorhexidine use *(178)*.

Two hospital trials were included in the 2014 *WHO recommendations on postnatal care of the mother and newborn (15)* but were not included in the current IPD analysis as individual patient data were not available. Both trials examined infants for omphalitis and reported similar effects to the IPD analysis (890 newborns, RR 0.48, 95% CI 0.28 to 0.84). One trial examined infants for neonatal mortality and reported a large, non-significant reduction (120 newborns, RR 0.11, 95% CI 0.01 to 2.04).

Values

See Box 3.10 in section 3.B.2: Preventive measures.

Resources

No economic evaluations of the application of chlorhexidine to the umbilical cord stump were identified.

Table 3.65 Main resource requirements for the application of chlorhexidine to the umbilical cord stump

Resource	Description
**Staff**	• Midwives/nurses, skilled birth attendants, or other trained health workers to apply chlorhexidine and/or to provide instruction for parents and other caregivers to apply chlorhexidine
**Training**	• Practice-based training for neonatal health workers including specific instruction on use of chlorhexidine for umbilical cord care (e.g. information leaflets, instructional videos)<br>• Training to recognize the signs and symptoms of umbilical cord infection
**Supplies**	• Information (written and/or pictorial, e.g. leaflets) for parents and other caregivers<br>• Chlorhexidine digluconate 7.1%, topical administration *(57)*:<br>– gel = US$ 0.36 for a 20 g tube ($0.0178 per gram)<br>– aqueous solution = US$ 0.0356 per ml (available in 10 ml, 15 ml and 100 ml bottles)<br>• Direct application with a clean finger or cotton balls
**Equipment and infrastructure**	• On-site pharmacy and/or medicine stock management system that is managed by a trained pharmacist or dispenser<br>• Access to soap and clean water and alcohol-based solutions for hand hygiene prior to application<br>• Safe storage of chlorhexidine tube/bottle away from sunlight
**Time**	• Application frequency varies, generally daily administration from birth for seven days
**Supervision and monitoring**	• Prompt referral and/or treatment following any signs or symptoms of umbilical cord infection or local reaction, as per usual care

Equity

No direct evidence was identified on the impact on health equity of application of chlorhexidine to the umbilical cord stump to reduce neonatal mortality and morbidity. Given the high-burden of sepsis-related neonatal morbidity and mortality in LMICs, use of chlorhexidine as an umbilical cord antiseptic could help to address health inequity in low- to middle-income settings. However, if families are expected to pay for chlorhexidine without any subsidization, the intervention may decrease equity.

Additional considerations

A 2010 household survey in rural Bangladesh found that most households were not willing to pay for chlorhexidine at the fixed price point, but indicated a willingness and capacity to borrow money to meet any shortfalls *(179)*. Another study in Bangladesh *(180)* found that if the unit price of multi-dose chlorhexidine was any higher than 25 Bangladesh Taka (US$ 0.35), at least some households would need to borrow money in order to pay for it, thus potentially requiring subsidization *(179)*.

Acceptability

A qualitative evidence synthesis of women's experiences of postnatal care found no direct evidence relating to women's views on the use of chlorhexidine to treat the umbilical cord stump (28). Indirect evidence from this review suggests that most women appreciate advice and information from health workers about treatments and techniques that optimize infant well-being (high confidence in the evidence). However, in some LMIC contexts, women may prefer to use traditional newborn care practices, including the use of specific oils, native plants and herbal compresses during the immediate postpartum period (moderate confidence in the evidence).

Feasibility

A qualitative evidence synthesis of women's experiences of postnatal care found no direct evidence relating to women's views on the feasibility of using chlorhexidine *(28)*. Indirect evidence from the same review indicates that some women in LMICs may be less likely to use chlorhexidine if they believe that treatment will incur additional or unnecessary costs (moderate confidence in the evidence). A qualitative evidence synthesis of health workers' experiences of postnatal care found no direct evidence relating to views on the feasibility of using chlorhexidine *(29)*. However, indirect evidence suggests that lack of personnel, resources and training may limit provision of information and counselling on cord care during the postnatal period (moderate confidence in the evidence).

Additional considerations

The WHO Model List of Essential Medicines for Children includes Chlorhexidine "solution or gel: 7.1% (digluconate) delivering 4% chlorhexidine (for umbilical cord care)" *(181)*.

Table 3.66 Summary of judgements: Routine application of chlorhexidine to the umbilical cord stump compared with dry or usual cord care

Domain	Judgement
Desirable effects	Small
Undesirable effects	Small
Certainty of the evidence	Low
Values	Probably no important uncertainty or variability
Balance of effects	Probably favours chlorhexidine
Resources required	Moderate costs
Certainty of the evidence on required resources	No included studies
Cost-effectiveness	Don't know
Equity	Varies
Acceptability	Varies
Feasibility	Varies

B.2.4 Sleeping position for the prevention of sudden infant death syndrome

RECOMMENDATION 33

Putting the baby to sleep in the supine position during the first year is recommended to prevent sudden infant death syndrome (SIDS) and sudden unexpected death in infancy (SUDI). *(Recommended)*

Remarks

- This recommendation is based on studies from high-income countries regardless of the gestational age of the baby. Other risk factors for SIDS (e.g. bed-sharing, sleeping place, parental smoking, etc.) were not considered.
- In making this recommendation, the Guideline Development Group also considered the evidence from ecological studies reporting the trends in post-neonatal mortality and SIDS rates from international vital statistics, epidemiologic studies of SIDS risk factors and outcomes of public health interventions that advocated non-prone sleeping to reduce the risk for SIDS.

Summary of evidence and considerations

Effects of the interventions (EB Table B.2.4)

Evidence was derived from a systematic review of the effect of sleep position on neonatal and infant outcomes in term, healthy newborns *(168)*. The review included 49 studies of which 32 observational studies provided data for this evidence summary. All but one study (conducted in Brazil) were conducted in HICs. For the studies reporting the outcomes of SIDS or SUDI, the exposure could be recorded any time up to 365 days of age, while for the remaining studies reporting the outcomes of apparent life-threatening event (ALTE), neurodevelopmental delay and positional plagiocephaly, exposure occurred at least once in the neonatal period. The highest incidence of SIDS was observed around a mean age of 2–4 months across all the studies.

Comparison: Supine (back) sleep position compared with non-supine (prone or side) sleep position

Newborn/infant outcomes

Infant mortality: It is uncertain whether sleeping in a supine position has any effect on deaths due to SIDS in infants < 1 year of age when compared with sleeping in a non-supine (prone or side) position (very low-certainty evidence). Low-certainty evidence suggests that sleeping in a supine position may decrease SUDI when compared with sleeping in a non-supine (prone or side) position (1 study, 384 infants; OR 0.39, 95% CI 0.23 to 0.65).

Severe neonatal/infant morbidity: It is uncertain whether sleeping in a supine position has any effect on unexplained SIDS or severe ALTE in the neonatal period when compared with sleeping in a non-supine (prone or side) position (very low-certainty evidence).

Neurodevelopment: Compared with sleeping in a prone position, moderate-certainty evidence suggests sleeping in a supine position probably worsens gross motor development at 6 months of age, measured as odds of being 0.5 SD below the mean on the gross motor scale of the Denver Developmental Screening Test (DDST) (1 study, 2097 infants; OR 1.67, 95% CI 1.22 to 2.27). Compared with sleeping in a side position, moderate-certainty evidence suggests sleeping in a supine position probably has little or no effect on gross motor development at 6 months of age, measured as odds of being 0.5 SD below the mean on the gross motor scale of the DDST (1 study, 8012 infants; OR 1.02, 95% CI 0.91 to 1.15). Compared with sleeping in a prone position, low-certainty evidence suggests sleeping in a supine position may have little or no effect on gross motor development at 18 months of age, measured as odds of being 0.5 SD below the mean on the gross motor scale of the DDST (1 study, 1919 newborns; OR 1.16, 95% CI 0.96 to 1.43). Compared with sleeping in a side position, low-certainty evidence suggests sleeping in a supine position may have little or no effect on gross motor development at 18 months of age, measured as odds of being 0.5 SD below the mean on the gross motor scale of the DDST (1 study, 7503 newborns; OR 1.12, 95% CI 0.86 to 1.45).

Health service use: It is uncertain whether sleeping in a supine position has any effect on hospital admissions

related to ALTE within 6 months of age[42] when compared with sleeping in a non-supine (prone or side) position (very low-certainty evidence).

Adverse effects: Low-certainty evidence suggests sleeping in a supine position may increase positional plagiocephaly within 28 weeks of age[43] when compared with sleeping in a non-supine position (prone or side) (2 studies, 471 newborns; OR 6.53, 95% CI 3.39 to 12.57).

Neonatal mortality was not reported in the included studies.

Maternal outcomes

Experience of postnatal care was not reported in the included studies.

Additional considerations

An analysis of trends in post-neonatal mortality and SIDS rates in Australia, Great Britain, the Netherlands, New Zealand, Norway, Sweden and the USA between 1980 and 1992 found that countries that experienced a rapid decline in prone sleeping also had reductions in SIDS rates of approximately 50% *(182)*. There were no significant changes in the proportion of parents who smoked cigarettes or in breastfeeding rates during this time. There was no apparent increase in adverse events such as deaths due to aspiration or ALTE, though these data were considered preliminary.

The Back to Sleep campaign, a large public health programme in the USA aiming to promote the supine sleeping position, was launched in 1994. This campaign remains ongoing (though has now been renamed as the Safe to Sleep campaign).[44] Significant increases in the supine sleep position from 13% to approximately 76% from 1992 to 2006, and a drop in the USA SIDS rate of about 50% over the same period, has subsequently been reported. The most dramatic declines occurred in the years immediately after the first non-prone sleep position recommendations *(183, 184)*. A study evaluating the impact of the Back to Sleep campaign from 1990 to 2012 in Colorado, USA, also reported significant decreases in SIDS incidence from 1.99/1000 live births in the pre-Back to Sleep era (1990-1993) to 0.57/1000 live births in the post-Back to Sleep era (1997–2012) ($P \leq 0.001$ for the trend) *(185)*.

Values

See Box 3.10 in section 3.B.2: Preventive measures.

Table 3.67 Main resource requirements for supine (back) sleep position

Resource	Description
**Staff**	• Midwives/nurses, other trained providers
**Training**	• In-service training to support supine sleeping, including counselling of families about supine sleeping and sleep safety post-discharge
**Supplies**	• Information (written and/or pictorial, e.g. leaflets) for parents/caregivers
**Equipment and infrastructure**	• Bassinet/crib/cot or other safe sleep surface
**Time**	• Additional time required for counselling of families regarding sleep position; otherwise same as for usual care
**Supervision and monitoring**	• *Pre-discharge:* Midwifery/nursing supervision to ensure safety of sleep position – Ongoing chart audit by clinical leaders to monitor sleeping position and address any barriers to supine sleeping<br>• *Post-discharge:* Discussion about sleeping position at postnatal visits and additional counselling on supine sleeping where needed

42 Assessed at 1, 3 and 6 months of age.

43 Assessed at 8–12 or 25–28 weeks of age.

44 The Back to Sleep campaign was initiated by the NICHD, AAP, Maternal and Child Health Bureau of the Health Resources and Services Administration, SIDS Alliance (now known as First Candle), and the Association of SIDS and Infant Mortality Programs. The focus of the campaign was to encourage parents to put their babies to sleep on their backs in order to reduce the risk of SIDS.

Resources

No economic evaluations of the supine sleep position in term newborns without complications were identified.

Equity

No direct evidence was identified on the impact on health equity of sleep position in term newborns without complications.

Additional considerations

The prevalence of SIDS is higher among socially and economically disadvantaged families *(186)*. There are also clear disparities in newborn sleeping practices according to racial, demographic, economic and other factors. For example, supine sleep positioning appears less prevalent among Hispanic and non-Hispanic black populations compared with non-Hispanic white populations *(187)*. Other studies have shown mothers with low levels of education are more likely to place their infant in a non-supine sleep position than mothers with higher levels of education *(188, 189)*. Lower caregiver income has also been associated with decreased likelihood of awareness of infant sleep position recommendations *(190)*.

Acceptability

A qualitative evidence synthesis of women's experiences of postnatal care found no direct evidence relating to women's views on infant sleeping positions *(28)*. Indirect evidence from this review suggests that most women appreciate advice and information from health workers about techniques that optimize infant well-being (high confidence in the evidence). However, in some LMIC contexts, women may prefer to adopt traditional newborn care practices during the immediate postpartum period (moderate confidence in the evidence).

Additional considerations

A qualitative evidence synthesis exploring decision-making for infant sleep environment among at-risk families *(191)* identified key issues that were prevalent among relatively deprived populations living in HICs. Parents were reluctant to accept health worker guidance on infant sleeping positions if they felt the advice was counter-intuitive or compromised their own experience (e.g. that placing infants in non-supine positions aid comfort, help with breathing or reduce the potential for choking). Parents wanted information and advice explained to them along with supporting evidence, rather than being told what to do in a didactic manner.

Feasibility

A qualitative evidence synthesis of women's experiences of postnatal care found no direct evidence relating to women's views on the feasibility of implementing the supine sleep position *(28)*. Indirect evidence from this review indicates that women want clear, consistent information from health workers about techniques or treatments that optimize infant well-being, including appropriate sleeping procedures (high confidence in the evidence). A qualitative evidence synthesis of health workers' experiences of postnatal care found no direct evidence relating to views on the feasibility of implementing the supine sleep position *(29)*. However, indirect evidence suggests that a lack of personnel, resources and training may limit the

provision of information and counselling on sleep position in term newborns during the postnatal period (moderate confidence in the evidence). Evidence suggests that availability of guidelines or policies on sleeping positions may prevent divergent opinions among health workers along the postnatal care pathway and help parents to feel secure about the information they receive (moderate confidence in the evidence).

Additional considerations

A qualitative synthesis exploring decision-making for infant sleep environment among at-risk families *(191)* identified several key issues prevalent among relatively deprived populations living in HICs. Findings indicate that parents in these communities may not trust advice on sleeping positions given by health workers, especially where it conflicts with information provided by trusted family members or community networks.

Table 3.68 Summary of judgements: Supine (back) sleep position compared with non-supine (prone or side) sleep position

Domain	Judgement
Desirable effects	Large
Undesirable effects	Trivial
Certainty of the evidence	Very low
Values	Probably no important uncertainty or variability
Balance of effects	Favours supine position
Resources required	Negligible costs or savings
Certainty of the evidence on required resources	No included studies
Cost-effectiveness	Don't know
Equity	Probably increased
Acceptability	Probably yes
Feasibility	Probably yes

B.2.5 Immunization for the prevention of infections

RECOMMENDATION 34

Newborn immunization should be promoted as per the latest existing WHO recommendations for routine immunization. *(Recommended)*

Remarks

- This recommendation has been adapted and integrated from the 2014 *WHO recommendations on postnatal care of the mother and newborn (15)*, based on Guideline Development Group consensus on existing WHO guidelines.
- The current WHO guidance on newborn immunizations (as of November 2021) are specified in Box 3.11, based on the latest WHO position papers recommending birth dose immunization for hepatitis B *(192)*, polio *(193)* and Bacille Calmette-Guérin (BCG) vaccines *(194)*.
- WHO recommends the following vaccines as early as 6 weeks of age: diphtheria–tetanus–pertussis-containing vaccine, haemophilus influenzae type b, pneumococcal conjugate vaccine and rotavirus *(195)*.
- WHO recommendations for routine immunization of children should be checked regularly for any updates *(195)*.

Box 3.11 WHO newborn immunization guidance and considerations (as of November 2021)

**Hepatitis B vaccine**
All infants should receive the first dose of the hepatitis B vaccine as soon as possible after birth, ideally within 24 hours. If administration within 24 hours is not feasible, all infants should receive the birth dose during the first contact with health facilities, up to the time of the first primary dose series.
If administration within 24 hours is not feasible, the birth dose can still be effective in preventing perinatal transmission if given within seven days, particularly within three days – although it will be somewhat less effective than if given within 24 hours, effectiveness declining with each passing day. Even after seven days, a late birth dose can be effective in preventing horizontal transmission and therefore remains beneficial.
The birth dose should be followed by two or three additional doses to complete the primary series. Vaccination schedules are described in the 2017 *Hepatitis B vaccines: WHO position paper (192).*
**Polio vaccine**
In polio-endemic countries and countries at high risk for importation and subsequent spread, WHO recommends a bivalent oral polio vaccination (bOPV) birth dose followed by a primary series of three bOPV doses and at least two inactivated poliovirus (IPV) dose.
**Bacille Calmette–Guérin (BCG) vaccine**
In countries or settings with a high incidence of TB disease and/or leprosy, a single dose of BCG vaccine should be given to neonates at birth, or as soon as possible thereafter, for prevention of TB and leprosy. If it cannot be given at birth, it should be given at the earliest opportunity thereafter and should not be delayed.
If the birth dose was missed, catch-up vaccination of unvaccinated older infants and children is recommended since evidence shows it is beneficial. Catch-up vaccination should be done at the earliest convenient encounter with the health system to minimize known or unknown exposure to TB- or leprosy-infected contacts.

B.3 NUTRITIONAL INTERVENTIONS

Background

Optimal nutrition is crucial for newborns to survive and thrive. The GDG considered the evidence and other relevant information to inform recommendations relating to supplementation of two important micronutrients, vitamin A and vitamin D.

Vitamin A serves important functions in vision, the immune system and general cellular functioning *(196)*. Infants and young children have increased vitamin A requirements due to their rapid growth and development. However, infants are born with low vitamin A stores and are dependent on external sources, most importantly breastmilk. In settings where vitamin A deficiency and/or undernutrition is common, infants are likely to receive inadequate amounts of vitamin A from breastmilk due to poor maternal nutritional status (197). Globally, vitamin A deficiency (serum retinol less than 0.70 µmol/L) is estimated to affect about 33%, or 190 million, children 6–59 months of age *(198, 199)*. The highest prevalence is found in South Asia (44%) and sub-Saharan Africa (48%) *(199)*. When severe, vitamin A deficiency can cause visual impairment (night blindness) and anaemia. It can also increase the risk of illness and death from common and preventable childhood infections, such as measles and those causing diarrhoea.

Vitamin D plays an important role in bone mineralization, muscle contraction, nerve conduction, general cellular functioning and the immune system. Acquisition of bone mineral content is greatest in the first year after birth *(200)*, and adequate bone mineralization during this period lays the foundation for strong bones later in life (201). Vitamin D has two active forms: vitamin D2 (ergocalciferol) and vitamin D3 (cholecalciferol). Vitamin D2 is naturally present only in fungi, while vitamin D3 can be obtained from dietary sources such as fatty fish and egg yolks, and/or through synthesis in the body following exposure to sunlight. Infants are generally considered to be at high risk of vitamin D deficiency as they have limited vitamin D stores at birth, infrequent exposure to sunlight, and relatively large vitamin D requirements due to their rapid growth D *(196)*. Severe vitamin D deficiency can lead to nutritional rickets; a skeletal disorder associated with impaired growth, limb and pelvic deformities, developmental delay, dental abnormalities and hypocalcemic seizures *(202)*. Although exclusive breastfeeding is recommended for infants up to 6 months of age, low levels of vitamin D (~50 IU/L) are often found in breastmilk *(203)*.

Box 3.12 Values

Findings from a qualitative evidence synthesis exploring what women want from postnatal care *(21)* indicate that women want a positive experience in which they are able to adapt to their new self-identity and develop a sense of confidence and competence as a mother. Findings from the same review also indicate that women tend to prioritize the needs of their infant (low confidence in the evidence) and, with this in mind, appreciate practical advice, support and information from health workers (moderate confidence in the evidence), provided this is delivered in a consistent manner and in a form they can understand (moderate confidence in the evidence).

B.3.1 Neonatal vitamin A supplementation

RECOMMENDATION 35a

Routine neonatal vitamin A supplementation is not recommended to reduce neonatal and infant mortality. *(Not recommended)*

RECOMMENDATION 35b

In settings with recent (within the last five years) and reliable data that indicate a high infant mortality rate (greater than 50 per 1000 live births) and a high prevalence of maternal vitamin A deficiency (> 10% of pregnant women with serum retinol concentrations < 0.70 µmol/L), providing newborns with a single oral dose of 50 000 IU of vitamin A within the first three days after birth may be considered to reduce infant mortality. *(Context-specific recommendation)*

Remarks

- In making this recommendation, the Guideline Development Group emphasized the need to avoid harm, given the uncertainty of the evidence and the conflicting results of research studies, as well as implementation costs.
- The proposed infant mortality rate of greater than 50 per 1000 live births was calculated based on several assumptions:
  - 50% of the total infant mortality rate are neonatal deaths;
  - 50% of neonatal mortality occurs within 24 hours after birth;
  - the post-neonatal mortality rate up to 6 months of age makes up two thirds (2/3) of the total infant mortality rate, and the mortality rate between 6 and 12 months of age makes up the remaining one third (1/3);
  - the rate of 30 deaths per 1000 used in the studies accounts for deaths between enrolment in the study up to 6 months of age;
  - dosing/enrolment almost always occurred within the first 24 hours after birth.

Summary of evidence and considerations

Effects of the interventions (EB Table B.3.1)

Evidence was derived from an updated Campbell systematic review of the effects of neonatal nutrition interventions on neonatal mortality and child health and development outcomes *(204)*, and from an individual participant data (IPD) meta-analysis on the effects of early neonatal vitamin A supplementation on infant mortality *(205)*. The IPD meta-analysis was used to further inform the evidence on the effects and safety of neonatal vitamin A supplementation, and to investigate the potential sources of heterogeneity. The Campbell review identified 16 trials (169 366 infants) that assessed the effect of neonatal vitamin A supplementation, of which 13 individually randomized or cluster RCTs (168 788 infants) contributed data to this evidence summary. All trials were conducted in LMICs. All 13 trials provided oral vitamin A as either a single dose of 50 000 IU, two doses of 24 000 IU (within 24 hours of one another), a single dose of 25 000 IU, or a single dose of 25 000 IU and 50 000 IU, compared with placebo.

The IPD meta-analysis included 11 trials assessing the effect of early neonatal vitamin A supplementation (25 000–50 000 IU intended to be given within 2–3 days after birth) compared with placebo, with infant follow-up through at least 6 months of age.

Comparison: Vitamin A supplementation compared with placebo or no vitamin A supplementation (Campbell systematic review)

Newborn/infant outcomes

Neonatal/infant mortality: High-certainty evidence shows early vitamin A supplementation has little or no effect on all-cause neonatal mortality (0–28 days after birth) when compared with placebo (6 trials, 126 548 infants; RR 0.99, 95% CI 0.90 to 1.08). Moderate-certainty evidence suggests early vitamin A supplementation probably has little or no effect on all-cause infant mortality at 6 months of age when

compared with placebo (12 trials, 154 940 infants; RR 0.98, 95% CI 0.89 to 1.07). High certainty evidence suggests that early vitamin A supplementation has little or no effect on all-cause infant mortality at 12 months of age when compared with placebo (8 trials, 118 376 infants; RR 1.04, 95% CI 0.94 to 1.14).

Adverse effects: Moderate-certainty evidence suggests vitamin A supplementation probably increases infant bulging fontanelle (within three days of dosing) when compared with placebo (6 trials, 100 256 infants; RR 1.53, 95% CI 1.12 to 2.09). High certainty evidence shows vitamin A supplementation has little or no effect on infants vomiting (within three days of dosing) when compared with placebo (5 trials, 99 582 infants; RR 1.00, 95% CI 0.93 to 1.07).

Severe neonatal morbidity was not reported in the included trials.

Maternal outcomes

Experience of postnatal care was not reported in the systematic review.

Comparison: Vitamin A supplementation compared with placebo or no vitamin A supplementation (IPD meta-analysis)[45]

Neonatal/infant mortality: Newborn vitamin A supplementation was associated with a variable effect from an 11% decrease to a 6% increase in the risk of death up to 6 months of age (11 trials; RR 0.97, 95% CI 0.89 to 1.06) and a variable effect from a 7% decrease to an 8% increase of death up to 12 months of age (10 trials; RR 1.00, 95% CI 0.93 to 1.08) when compared with placebo.

Univariate meta-regression and meta-analyses by study level characteristics

Meta-regression identified five variables as significantly associated with the effect of newborn vitamin A supplementation on mortality at six months, as presented below. Meta-analyses by study-level characteristics were subsequently conducted for these variables.

Geographic region: Until 6 months of age, newborn vitamin A supplementation was associated with a 13% lower risk of death in Asian countries (5 trials; RR 0.87, 95% CI 0.77 to 0.98), but in African countries the risk of death includes a variable effect from a 2% reduction to a 15% increase (6 trials; RR 1.06, 95% CI 0.98 to 1.15). Until 12 months of age, newborn vitamin A supplementation was associated with a variable effect from a 20% reduction to a 3% increase in the risk of death in Asian countries (4 trials; RR 0.91, 95% CI 0.80 to 1.03), and a variable effect from no effect to a 15% increase in African countries (6 trials; RR 1.07, 95% CI 1.00 to 1.15).

Maternal vitamin A deficiency: Until 6 months of age, and in study populations where the prevalence of maternal vitamin A deficiency was moderate or severe, newborn vitamin A supplementation was associated with a 13% lower risk of death (3 trials; RR 0.87, 95% CI 0.80 to 0.94). In study populations where there was no or mild maternal vitamin A deficiency, the risk of death includes a variable effect, from a 4% lower to a 15% higher risk of death (7 trials; RR 1.05, 95% CI 0.96 to 1.15). Until 12 months of age, and in study populations where the prevalence of maternal vitamin A deficiency was moderate or severe, newborn vitamin A supplementation was associated with a variable effect, from a 17% lower risk to no effect on the risk of death (2 trials; RR 0.91, 95% CI 0.83 to 1.00). In study populations where there was no or mild maternal vitamin A deficiency, the risk of death includes a variable effect, from a 2% lower risk to a 15% higher risk (7 trials; RR 1.06, 95% CI 0.98 to 1.15).

Early infant mortality: Newborn vitamin A supplementation was associated with a 9% lower risk of death up to 6 months of age in study populations where 6-month mortality in the control group was ≥ 30 per 1000 live births (6 trials; RR 0.91, 95% CI 0.85 to 0.98), but includes a variable effect from a 5% lower risk to a 24% higher risk of death in populations where 6-month mortality in the control group was under 30/1000 (5 trials; RR 1.08, 95% CI 0.95 to 1.24).

Ratio of 6-month to 12-month mortality in the control group: Newborn vitamin A supplementation was associated with a variable effect from a 16% lower to a 1% higher risk of death up to 6 months of age in study populations where 75% or more of infant mortality occurred in the first 6 months (6 trials; RR 0.92, 95% CI 0.84 to 1.01), but was associated with a variable effect from no effect to a 22% higher risk of mortality in populations where less than 75% of infant mortality occurred up to 6 months of age (4 trials; RR 1.11, 95% CI 1.00 to 1.22).

45 The number of infants for each outcome is not presented as these data were not reported in the meta-analysis. Certainty of the evidence could not be graded, as the necessary information to enable grading was not available in the original publication.

Maternal education: Newborn vitamin A supplementation was associated with a 12% lower risk of death up to 6 months of age in populations where ≥ 32% of mothers had never been to school (4 trials; RR 0.88, 95% CI 0.80 to 0.96), but was associated with a variable effect from an 8% lower to an 18% higher risk of death in study populations where < 32% of mothers had never been to school (5 trials; RR 1.04, 95% CI 0.92 to 1.18).

Subgroup analysis by neonatal risk status (high risk or low risk)

Vitamin A given to < 2500 g newborns: Vitamin A supplementation in newborns whose birthweight was < 2500 g was associated with a variable effect from an 11% decrease to a 5% increase in the risk of death up to 6 months of age (8 trials; RR 0.97, 95% CI 0.89 to 1.05) and a variable effect from an 8% decrease to an 8% increase in the risk of death up to 12 months of age (7 trials; RR 0.99, 95% CI 0.92 to 1.08) when compared with placebo.

Vitamin A given to ≥ 2500 g newborns: Vitamin A supplementation in newborns whose birthweight was ≥ 2500 g was associated with a variable effect from an 11% decrease to a 4% increase in the risk of death up to 6 months of age (8 trials; RR 0.96, 95% CI 0.89 to 1.04) and a variable effect from a 7% decrease to a 7% increase in the risk of death up to 12 months of age (7 trials; RR 1.00, 95% CI 0.93 to 1.07) when compared with placebo.

Subgroup analysis by maternal risk of vitamin A deficiency

Vitamin A given to newborns of mothers with night blindness during pregnancy: Vitamin A supplementation in newborns whose mothers had night blindness during pregnancy was associated with a variable effect from a 35% decrease to a 33% increase in the risk of death up to 6 months of age (3 trials; RR 0.93, 95% CI 0.65 to 1.33) and a variable effect from a 36% decrease to a 37% increase of death up to 12 months of age (2 trials; RR 0.94, 95% CI 0.64 to 1.37) when compared with placebo.

Vitamin A given to newborns of mothers without night blindness during pregnancy: Vitamin A supplementation in newborns whose mothers did not had night blinding during pregnancy was associated with a 14% decrease in the risk of death up to 6 months of age (3 trials; RR 0.86, 95% CI 0.77 to 0.96) but a variable effect from a 23% decrease to no effect in the risk of death up to 12 months of age (2 trials; RR 0.88, 95% CI 0.78 to 1.00) when compared with placebo.

Subgroup analysis by initiation of breastfeeding

Vitamin A given to newborns initiating breastfeeding during the first hour after birth: Vitamin A supplementation in newborns who initiated breastfeeding during the first hour after birth was associated with a variable effect from a 6% decrease to a 14% increase in the risk of death up to 6 months of age (6 trials; RR 1.03, 95% CI 0.94 to 1.14) and a variable effect from a 5% decrease to a 12% increase in the risk of death up to 12 months of age (6 trials; RR 1.03, 95% CI 0.95 to 1.12), when compared with placebo.

Vitamin A given to newborns initiating breastfeeding 2–23 hours after birth: Vitamin A supplementation in newborns who initiated breastfeeding 2–23 hours after birth was associated with a variable effect from a 13% reduction to a 3% increase in the risk of death up to 6 months of age (6 trials; RR 0.94, 95% CI 0.87 to 1.03) and a variable effect from a 10% decrease to a 5% increase in the risk of death up to 12 months of age (6 trials; RR 0.97, 95% CI 0.90 to 1.05), when compared with placebo.

Vitamin A given to newborns initiating breastfeeding ≥ 24 hours after birth: Newborn vitamin A supplementation in newborns who initiated breastfeeding ≥ 24 hours after birth was associated with a variable effect from a 26% decrease to a 13% increase in the risk of death up to 6 months of age (6 trials; RR 0.92, 95% CI 0.74 to 1.13) and a variable effect from a 26% decrease to an 8% increase in the risk of death up to 12 months of age (6 trials; RR 0.90, 95% CI 0.74 to 1.08), when compared with placebo.

Subgroup analysis by newborn sex

Vitamin A given to male newborns: Vitamin A supplementation in male newborns was associated with a variable effect from an 8% decrease to an 8% increase in the risk of death up to 6 months of age (11 trials; RR 0.99, 95% CI 0.92 to 1.08) and a variable effect from a 7% decrease to a 7% increase in the risk of death up to 12 months of age (10 trials; RR 1.00, 95% CI 0.93 to 1.07), when compared with placebo.

Vitamin A given to female newborns: Newborn vitamin A supplementation in female newborns was associated with a variable effect from a 14% decrease

to a 1% increase in the risk of death up to 6 months of age (11 trials; RR 0.93, 95% CI 0.86 to 1.01) and a variable effect from an 8% decrease to a 6% increase in the risk of death up to 12 months of age (10 trials; RR 1.01, 95% CI 0.92 to 1.06), when compared with placebo.

Additional considerations

The Campbell review reported non-pooled results on long term neurodevelopment outcomes. One trial (2067 infants) *(206)* suggested neonatal vitamin A supplementation had no adverse effect on development (assessed using Bayley Scales of Infant Development at 3 years of age, having had vitamin A supplementation as neonates) irrespective of the presence or absence of bulging fontanelle (assessed by palpation of the anterior fontanelle), or any other signs or symptoms (e.g. fever, irritability, vomiting or diarrhoea) or an increase in intracranial pressure, as reflected by the resistive index. Neonatal vitamin A supplementation had a positive effect on all developmental scores. Another trial (15 937 infants) *(207)* found no evidence of a difference on neurodevelopment (intelligence, memory and motor function) at eight years post vitamin A supplementation in neonates or pregnant women; however, when the neonates and their mothers were both supplemented with vitamin A versus placebo, it increased their performance in reading, spelling and maths calculations.

Values

See Box 3.12 in section 3.B.3: Nutritional interventions.

Additional considerations

In general, health workers, policy-makers and parents, caregivers and family members in all settings are likely to place a high value on the survival of infants and young children and on safe interventions that lead to improved survival without causing morbidity.

Resources

No economic evaluations of neonatal vitamin A supplementation were identified.

Additional considerations

Operational research may be needed to determine the resources required based on the country or regional contexts within a country, such as differences between and within populations, urban and rural settings, and health systems, particularly the delivery of quality maternal and newborn care.

Table 3.69 Main resource requirements for neonatal vitamin A supplementation

Resource	Description
**Staff**	• Midwives/nurses, skilled birth attendants or other trained provider
**Training**	• Practice-based training for neonatal health workers
**Supplies**	• Vitamin A (retinol) 50 000 IU/ml drops (oral administration) = US$ 1.70 per 30 ml bottle (US$ 0.06 per ml) *(57)*<br>• Vitamin A (retinol) soft gel capsules (as palmitate; oil formulation for oral administration) *(123)*:<br>– 100 000 IU soft gel caps = US$ 1.79 per pack of 100<br>– 100 000 IU soft gel caps = US$ 7.54 per pack of 500<br>– 200 000 IU soft gel caps = US$ 2.79 per pack of 100<br>– 200 000 IU soft gel caps = US$ 11.25 per pack of 500
**Equipment and infrastructure**	• On-site pharmacy and/or medicine stock management system that is managed by a trained pharmacist or dispenser<br>• Some vitamin A supplements need to be stored in cool conditions (e.g. below 25 °C); some may need to be refrigerated once opened, while others do not require refrigeration
**Time**	• Dispensing time estimated to be 2 minutes<br>• Administration as a single or daily dose directly into the baby's mouth (drop or squeezable capsule formulation)
**Supervision and monitoring**	• Medical and/or midwifery monitoring for any adverse effects of supplementation

Equity

No direct evidence was identified on the impact on health equity of neonatal vitamin A supplementation. Vitamin A deficiency is a public health problem in LMICs, especially in areas of high maternal vitamin A deficiency. Effective interventions to improve newborn nutrition in such populations could help to address health inequities. However, if caregivers and families are expected to pay for supplements, vitamin A supplementation may decrease equity.

Additional considerations

It is important to assess the availability of subnational data in order to target and reduce inequities within countries and, when available, to use the disaggregated data to identify areas in need of programme availability, strengthening and support, as well as areas of high coverage, which may offer important lessons, insights and good practices.

Acceptability

A qualitative evidence synthesis of women's experiences of postnatal care found no direct evidence relating to women's views on vitamin A supplementation for newborns *(28)*. Indirect evidence from this review indicates that women want more information and support from health workers, particularly around the feeding and nutritional needs of their infant (high confidence in the evidence) and are likely to welcome interventions that optimize the well-being of their baby (moderate confidence in the evidence). However, women in certain contexts (particularly LMIC settings) may be resistant to vitamin supplementation because of personal preferences or community expectations relating to traditional dietary and/or nutritional practices (moderate confidence in the evidence).

Additional considerations

From the NeoVita trials in Ghana, India and the United Republic of Tanzania *(208-210)*, neonatal vitamin A supplementation has been accepted for research purposes by families and health workers. However, in the Bangladesh mechanistic study, it was reported that 11% (72/636) of pregnant women eligible for the study refused participation, not wishing to give their newborns supplements *(211)*.

In a feasibility study conducted in Bangladesh and Nepal *(212)*, mothers viewed neonatal vitamin A supplementation favourably with few reports of negative experiences. The negative experiences were largely self-limiting and not clearly attributable to the intervention. Health workers viewed the intervention favourably.

The potential for benefit in some contexts and the potential for harm in other contexts may lead to differences in acceptability among various population groups. If restricted to specific settings in which the intervention has shown significant benefit, the intervention would likely be acceptable.

Feasibility

A qualitative evidence synthesis of women's experiences of postnatal care found no direct evidence relating to women's views on the feasibility of using Vitamin A supplementation for their newborns *(28)*. Indirect evidence from the same review indicates there may be feasibility challenges in some LMICs among women who don't understand and/or value neonatal supplementation strategies (moderate confidence in the evidence). In addition, women and families in low resource settings may be less motivated to engage with health workers if they think health facilities are under-resourced or if they believe treatment will incur additional costs (moderate confidence in the evidence).

A qualitative evidence synthesis of health workers' experiences of postnatal care found no direct evidence relating to views on vitamin supplementation for newborns *(29)*. However, indirect evidence suggests lack of personnel, resources and training may limit provision of information and counselling on vitamin supplementation for newborns (moderate confidence in the evidence).

Additional considerations

The pooled analysis of the NeoVita trials suggests the inclusion of neonatal vitamin A dosing in the interventions that can be implemented in the immediate postnatal period and the first few days after birth is feasible and, potentially, cost-

effective. Data from the published trials used in the pooled analysis suggest it is possible for vitamin A supplementation to be administered by health workers in community and hospital settings. In India, approximately 45% of births took place in the home, whereas in Ghana and the United Republic of Tanzania, 76–87% of births took place in a health facility. Pilot data from Nepal and Bangladesh also indicate that delivering neonatal vitamin A supplementation is feasible *(212)*.

Vitamin A (retinol) is listed in the WHO Model List of Essential Medicines for Children *(181)* as oral oily solution, tablets and water-miscible injection. Some vitamin A supplements need to be stored in cool conditions (e.g. below 25 °C) and some require refrigeration once opened.

Table 3.70 Summary of judgements: Neonatal vitamin A supplementation compared with placebo or no supplementation

Domain	Judgement
Desirable effects	Trivial
Undesirable effects	Small
Certainty of the evidence	High
Values	Probably no important uncertainty or variability
Balance of effects	Probably favours no supplementation
Resources required	Negligible costs or savings
Certainty of the evidence on required resources	No included studies
Cost-effectiveness	Don't know
Equity	Varies
Acceptability	Varies
Feasibility	Probably yes

B.3.2 Vitamin D supplementation for breastfed, term infants

RECOMMENDATION 36

Vitamin D supplementation in breastfed, term infants is recommended for improving infant health outcomes only in the context of rigorous research. *(Context-specific recommendation)*

Remarks

- The Guideline Development Group acknowledged that vitamin D supplementation is currently recommended within the first weeks after birth as part of national guidance in many countries to improve vitamin D status and prevent rickets; however, there was agreement that, at the present time, there is insufficient evidence on the benefits and harms, if any, of routine vitamin D supplementation on health outcomes of term, breastfed infants.
  - Vitamin D supplementation in infants was found to improve 25-hydroxyvitamin D (25(OH)D) concentrations and reduce the prevalence of serum 25(OH)D concentrations < 50 nmol/L.
  - However, there was no evidence that vitamin D supplementation in infants reduces the prevalence of serum 25(OH)D concentrations < 30 nmol/L, prevents rickets or improves bone health.
  - There was little evidence reported on adverse effects; however, adverse effects would not be expected with daily doses providing the safe and adequate intake level.
  - Evidence from non-breastfed infants was not considered by this guideline panel as standards for infant formula include fortification with vitamin D *(213)*.
- In addition to variable acceptability of the intervention across stakeholders, the provision of vitamin D supplements in infants is likely to incur some costs, which does not support its use for all breastfed, term infants.
- It is generally recommended that infants less than 6 months of age be protected from UV rays as much as possible, preferably being kept away from direct sunlight and having their skin protected by appropriate clothing and hats to reduce the risk of skin cancer and adverse effects of excessive sunlight exposure (e.g. sun burn). Phototherapy for the treatment of neonatal jaundice is an exception to this general recommendation.
- Research in this context includes adequately powered studies on the effect of neonatal vitamin D supplementation on mortality, morbidity, growth and development, including clinically relevant outcomes (both benefits and harms), assessment of vitamin D status and cost-effectiveness of this intervention in breastfed and non-breastfed infants.

Summary of evidence and considerations

Effects of the interventions (EB Table B.3.2)

Evidence was derived from an updated Cochrane systematic review of vitamin D supplementation for breastfed, term infants to prevent vitamin D deficiency and improve bone health *(214)*. Trials evaluating vitamin D supplementation given directly to the infant were included in this evidence summary. Eight trials (536 breastfed infants) were considered, one of which was a three-arm trial. Trials were conducted in Australia (1), India (1), Mexico (1), Norway (1), Spain (1) and the USA (3).

All infants were singleton, healthy, full-term infants, exclusively breastfed or whose mothers intended to exclusively breastfeed. Initiation of supplementation varied across studies, from at birth to six weeks after birth (where described). Doses and active forms of vitamin D also varied across trials: vitamin D2 (ergocalciferol) drops at a dose of 400 IU/day until seven weeks, and three and six months were used in three trials (88 infants); vitamin D3 (cholecalciferol) drops at a single dose of 50 000 IU were used in one trial (49 infants); and doses of 200 IU, 400 IU, 402 IU/day until 6, 9 and 12 months were used in four trials (448 infants). Subgroup analyses were performed for infants at high-risk of vitamin D deficiency, due to skin pigmentation, covering or avoidance of sun exposure, and/or latitude (that is, insufficient ultraviolet intensity most of the year) versus low-risk infants, vitamin D dose, time of initiation, and form of vitamin D (D2 or D3).

Comparison: Vitamin D supplementation for breastfed, term infants compared with placebo or no supplementation

Newborn/infant outcomes

Subclinical or clinical vitamin D deficiency: Low-certainty evidence suggests that infants receiving vitamin D supplementation may have a lower risk of vitamin D insufficiency (plasma/serum 25-hydroxyvitamin D [25(OH)D] concentrations < 50 nmol/L) (4 trials, 274 infants; RR 0.57, 95% CI 0.41 to 0.80) when compared with infants not receiving vitamin D supplementation or receiving placebo. Low-certainty evidence suggests that vitamin D supplementation may increase plasma/serum 25(OH)D concentrations at latest time reported up to 6 months of age (6 trials, 334 infants; MD 22.63 nmol/L higher, 95% CI 17.05 higher to 28.21 higher) when compared with no supplementation or receiving placebo. It is uncertain whether vitamin D supplementation affects vitamin D deficiency (25(OH)D concentrations < 30 nmol/L), nutritional rickets (biochemical), weight, length, head circumference at 6 months of age, or bone mineral content at the end of intervention (very low-certainty evidence).

Adverse effects: It is uncertain whether vitamin D supplementation causes adverse effects (hypercalcaemia or other) when compared with no vitamin D supplementation or placebo (very low-certainty evidence).

Neonatal mortality and severe *neonatal morbidity* were not reported in the systematic review.

Maternal outcomes

Experience of postnatal care was not reported in the systematic review.

Subgroup analysis by neonatal risk status (high risk or low risk)

Vitamin D supplementation given to high-risk infants compared with no vitamin D supplementation or placebo

Subclinical or clinical vitamin D deficiency: Low-certainty evidence suggests that vitamin D supplementation among high-risk infants may reduce vitamin D insufficiency (plasma/serum 25(OH)D concentrations < 50 nmol/L) (3 trials, 134 infants; RR 0.65, 95% CI 0.46 to 0.94) when compared with no vitamin D supplementation or placebo. Low-certainty evidence suggests that vitamin D supplementation among high-risk infants may increase plasma/serum 25(OH)D concentrations (nmol/L) at latest time reported (up to 6 months of age; 3 trials, 134 infants; MD 18.24 nmol/L higher, 95% CI 9.39 higher to 27.09 higher) when compared with no vitamin D supplementation or placebo. It is uncertain whether vitamin D supplementation in high risk infants affects vitamin D deficiency (plasma/serum 25(OH)D < 30 nmol/L) or nutritional rickets (biochemical) when compared with no vitamin D supplementation or placebo (very low-certainty evidence).

Comparison: Vitamin D supplementation given to low-risk infants compared with no vitamin D supplementation or placebo

Subclinical or clinical vitamin D deficiency: Low-certainty evidence suggests that vitamin D supplementation among low-risk infants may reduce vitamin D insufficiency (plasma/serum 25(OH)D concentrations < 50 nmol/L) (1 trial, 140 infants; RR 0.19, 95% CI 0.07 to 0.53) when compared with no vitamin D supplementation or placebo. Low-certainty evidence suggests that vitamin D supplementation among low-risk infants may increase plasma/serum 25(OH)D levels (nmol/L) at latest the point reported up to 6 months of age (3 trials, 200 infants; MD 25.53 higher, 95% CI 18.34 higher to 32.72 higher) when compared with no vitamin D supplementation or placebo. It is uncertain whether vitamin D supplementation in low-risk infants affects the risk of nutritional rickets (biochemical; vitamin D2 200 IU/day from birth to 6 months of age) when compared with no vitamin D supplementation or placebo (very low-certainty evidence). Low-certainty evidence suggests that vitamin D supplementation among low-risk infants (D2 400 IU/day from birth to 3 months of age) may improve baby bone mineral content at the end of intervention when compared with placebo or no treatment (1 trial, 18 infants; MD 15 higher, 95% CI 6.68 higher to 23.32 higher). Low-certainty evidence suggests that vitamin D supplementation among low-risk infants (D2 400 IU/day from birth to 6 months of age) may reduce bone mineral content at the end of the intervention when compared with placebo or no treatment (1 trial, 38 infants; MD 11.5 lower, 95% CI 21.32 lower to 1.68 lower).

Subgroup analysis by different active form (vitamin D2 or D3)

Vitamin D supplementation given as vitamin D2 or D3 compared with placebo

Subclinical or clinical vitamin D deficiency: Low-certainty evidence suggests that infants receiving vitamin D3 supplementation may have lower risk of vitamin D insufficiency (25(OH)D < 50 nmol/L) when compared with no vitamin D supplementation or placebo (3 trials, 262 infants; RR 0.58, 95% CI 0.40 to 0.82). It is uncertain whether vitamin D2 supplementation affects vitamin D insufficiency (25(OH)D < 50 nmol/L) or vitamin D deficiency (25(OH)D < 30 nmol/L) when compared with no vitamin D supplementation or placebo. It is uncertain whether vitamin D3 supplementation affects vitamin D deficiency (25(OH)D < 30 nmol/L) when compared with no vitamin D supplementation or placebo.

Subgroup analysis by dosage (single oral dose of 50 000 IU or 400 IU daily)

Vitamin D supplementation as a daily oral dose of 400 IU compared with placebo

Subclinical or clinical vitamin D deficiency: Low-certainty evidence suggests that infants receiving vitamin D supplementation as a daily oral dose of 400 IU may have lower risk of vitamin D insufficiency (25(OH)D < 50 nmol/L) when compared with placebo (3 trials, 253 infants; RR 0.56, 95% CI 0.39 to 0.81). It is uncertain whether vitamin D supplementation as a daily oral dose of 400 IU affects vitamin D deficiency (25(OH)D < 30 nmol/L) when compared with placebo.

Vitamin D supplementation as a single oral dose of 50 000 IU compared with placebo

Subclinical or clinical vitamin D deficiency: It is uncertain whether vitamin D supplementation as a single oral dose of 50 000 IU affects vitamin D insufficiency (25(OH)D < 50 nmol/L) or vitamin D deficiency (25(OH)D < 30 nmol/L) when compared with placebo.

Subgroup analysis by time of administration (from birth, from 1 month of age)

Vitamin D supplementation given from birth compared with placebo

Subclinical or clinical vitamin D deficiency: Low-certainty evidence suggests that infants receiving vitamin D supplementation from birth may have a reduced risk of vitamin D insufficiency (25(OH)D <50 nmol/L) when compared with placebo (3 trials, 134 infants; RR 0.65, 95% CI 0.46 to 0.94). It is uncertain whether vitamin D supplementation given from birth affects vitamin D deficiency (25(OH)D < 30 nmol/L) when compared with placebo.

Vitamin D supplementation given from 1 month of age compared with placebo

Subclinical or clinical vitamin D deficiency: Low-certainty evidence suggests that infants receiving vitamin D supplementation given from 1 month of age may have a reduced risk of vitamin D insufficiency (25(OH)D < 50 nmol/L) when compared with placebo (1 trial, 140 infants; RR 0.19, 95% CI 0.07 to 0.53).

Subgroup analysis by duration of supplementation (single, oral 50 000 IU at birth, 1-2 months or > 6 months)

Vitamin D supplementation given as a single, oral dose of 50 000 IU at birth compared with placebo

Subclinical or clinical vitamin D deficiency: It is uncertain whether vitamin D supplementation given at birth as a single, oral dose of 50 000 IU affects vitamin D insufficiency (25(OH)D < 50 nmol/L) or vitamin D deficiency (25(OH)D < 30 nmol/L) when compared with placebo.

Vitamin D supplementation given for 1–2 months after birth compared with placebo

Subclinical or clinical vitamin D deficiency: It is uncertain whether vitamin D supplementation given 1–2 months after birth affects vitamin D insufficiency (25(OH)D < 50 nmol/L) when compared with placebo.

Vitamin D supplementation given for > 6 months after birth compared with placebo

Subclinical or clinical vitamin D deficiency: Low-certainty evidence suggest that infants receiving vitamin D supplementation for more than six months after birth may have a reduced risk of vitamin D insufficiency (25(OH)D < 50 nmol/L) when compared with placebo (2 trials, 241 infants; RR 0.57, 95% CI 0.39 to 0.83). It is uncertain whether vitamin D supplementation given for more than six months after birth affects vitamin D deficiency (25(OH)D < 30 nmol/L) when compared with placebo.

Additional considerations

WHO recommends vitamin D supplementation in very low birthweight infants at a dose ranging from 400 IU to 1000 IU per day until 6 months of age *(215)*.

The systematic review did not report the priority outcomes of neonatal morbidity or mortality. Only one of the included trials evaluated neonatal morbidity *(216)* and reported that infants in the placebo group were more likely to report diarrhoea and respiratory illnesses than infants supplemented with vitamin D (median 13.0; IQR 7.0–28.5; $P < 0.05$). When differences between groups were assessed separately for diarrhoea or respiratory illness, there was no statistically significant difference. Admission to hospital was needed for 22 infants, 11 in each of the vitamin D supplementation and placebo groups.

Values

See Box 3.12 in section 3.B.3: Nutritional interventions.

Resources

No economic evaluations of vitamin D supplementation for breastfed, term infants were identified.

Additional considerations

A cost-effectiveness modelling study of the use of vitamin D supplementation in pregnant women and infants and children < 4 years of age in the United Kingdom, where rickets has an estimated annual incidence of 29.75 per 100 000 children < 4 years of age, found that vitamin D supplementation in dark skin tone populations was cost saving. In medium skin tone populations and light skin tone populations, the incremental cost-effectiveness ratio was £19 295 per QALY and £404 047 per QALY, respectively *(217)*. Overall, supplementation was cost-saving in participants with a dark skin tone, cost-effective in participants with a medium skin tone, but not cost-effective in participants with a light skin tone.

Supplements containing vitamin D are available on prescription or for sale without a prescription from pharmacies, shops and online in many countries. However, there is wide variation in the content and price, and some supplements may not be suitable for at-risk groups.

Table 3.71 Main resource requirements for vitamin D supplementation of breastfed, term infants

Resource	Description
**Staff**	• Doctors/midwives/nurses, or else none required (where purchased privately without a prescription)
**Training**	• Practice-based training for health workers, or else none required
**Supplies**	• *International medical products price guide:* Vitamin D3 (cholecalciferol) 10 000 IU/ml drops (oral administration) = US$ 2.15 per 30 ml bottle (US$ 0.07 per m) *(57)*<br>• *Private purchase:* Cost varies widely by manufacturer, supplier and dose; indicative range = US$ 0.10–1.50 per ml (oral administration of drops)
**Equipment and infrastructure**	• On-site pharmacy and/or medicine stock management system that is managed by a trained pharmacist or dispenser<br>• Some vitamin D supplements need to be stored in cool conditions (e.g. below 25 °C) and some may need refrigeration once opened, while others do not require refrigeration<br>• For breastfeeding infants < 6 months of age, drops are administrated directly into the baby's mouth or onto the mother's breast (or another clean surface)
**Time**	• Dispensing time estimated to be 2 minutes<br>• Daily administration is generally required; some regimens involve a single dose given at birth
**Supervision and monitoring**	• Same as for usual care

Equity

No direct evidence was identified on the impact on health equity of vitamin D supplementation for breastfed, term infants. Limited available data suggest that vitamin D deficiency is widespread at the global level and may be particularly prevalent in settings lacking vitamin D fortification programmes *(218)*. Effective interventions to improve newborn nutrition in such populations could help to address health inequities. However, if caregivers and families are expected to pay for supplements, vitamin D supplementation may decrease equity.

Additional considerations

In the USA, daily vitamin D supplementation has been recommended for breastfed infants since 2008 *(219)*. In an examination of trends in meeting vitamin D intake among infants in the USA since 2009 *(220)*, only 20.5% of breastfed infants were found to meet the intake requirements of 400 IU/day of vitamin D. Breastfed infants in families with a total income ≥ 400% of the federal poverty level, with a head of household being a college graduate, and with private health insurance, were more likely to meet the intake guidelines. The authors suggested that renewed considerations are needed for how best to meet recommended vitamin D intake for infants.

Acceptability

A qualitative evidence synthesis of women's experiences of postnatal care found no direct evidence relating to women's views on vitamin D supplementation for breastfed, term infants *(28)*. Indirect evidence from this review indicates that women want more information and support from health workers, particularly around the feeding and nutritional needs of their infant (high confidence in the evidence) and are likely to welcome interventions that optimize the well-being of their baby (moderate confidence in the evidence). However, women in certain contexts (particularly LMIC settings) may be resistant to vitamin supplementation because of personal preferences or community expectations relating to traditional dietary and/or nutritional practices (moderate confidence in the evidence).

Additional considerations

A survey of breastfeeding mothers showed just over half supplemented their young infants with vitamin D in the preceding week, with 42% receiving the recommended dose of 400 IU *(221)*. Most found administering vitamin D supplements easy, but 6% found it burdensome. The great majority (88%) of mothers preferred to supplement themselves rather than their infants. Reasons for not supplementing infants with vitamin D included lack of knowledge about supplementation, belief that breastmilk provided infants with adequate vitamin D, inconvenience, or their infant's apparent dislike of the supplement *(221)*.

An observational study of a primary care practice in the USA showed just over 36% of paediatricians recommended vitamin D supplementation for breastfed newborns *(222)*. Those who did not recommend supplementation were more likely to believe that the guidance to supplement was not evidence-based. Only 44.6% of parents of infants whose paediatrician recommended supplements actually provided them. About two thirds of parents of infants predominantly breastfed for at least six months believed breastmilk contained adequate amounts of nutrients for infants, and only 3% of their infants received vitamin D supplementation *(222)*.

There is some concern among health workers and breastfeeding advocates that recommendations to supplement breastmilk with vitamin D will imply to women that formula is superior to breastmilk *(223, 224)*. This implication may result in fewer women choosing to breastfeed and/or more women reducing or discontinuing breastfeeding.

Feasibility

A qualitative evidence synthesis of women's experiences of postnatal care found no direct evidence relating to women's views on the feasibility of using vitamin D supplementation in breastfed, term infants *(28)*. Indirect evidence from the same review indicates there may be challenges in some LMICs among women who do not understand and/or value postnatal supplementation strategies (moderate confidence in the evidence). In addition, women and families in low resource settings may be less motivated to engage with health workers if they think health facilities are under-resourced or if they believe treatment will incur additional costs (moderate confidence in the evidence).

A qualitative evidence synthesis of health workers' experiences of postnatal care found no direct evidence relating to views on vitamin supplementation for newborns *(29)*. However, indirect evidence suggests that lack of personnel, resources and training may limit provision of information and counselling on vitamin

supplementation for newborns (moderate confidence in the evidence).

Additional considerations

Surveys conducted in Canada *(225, 226)* and Ireland *(227)*, where there are national recommendations for vitamin D supplementation of young infants, report variable implementation, from about half to 92%. Women from British Columbia, Canada, who provided vitamin D supplementation to their breastfed infants recalled hearing the recommendations at least once from a public health nurse or physician, understood breastmilk had inadequate amounts of vitamin D, and believed supplementation provided health benefits to the infant *(225)*.

Poor adherence with long-term micronutrient supplementation regimens and costs to consumers have been cited as practical limitations for LMICs *(218)*. Adherence is often influenced by individual, social and environmental factors, including personal beliefs, the motivation of the mother or caregiver, social networks, health workers, others involved in the delivery of programmes and the local health system structure.

Vitamin D3 supplements (cholecalciferol) are listed in the WHO Model List of Essential Medicines for Children (oral liquid: 400 IU/ml; solid oral dosage form: 400 IU; 1000 IU) *(181)*. Vitamin D2 (ergocalciferol) is listed as a suitable alternative. Some vitamin D supplements need to be stored in cool conditions (e.g. below 25 °C) and some require refrigeration once opened.

Table 3.72 Summary of judgements: Vitamin D supplementation compared with placebo or no supplementation

Domain	Judgement
Desirable effects	Small
Undesirable effects	Don't know
Certainty of the evidence	Low
Values	Probably no important uncertainty or variability
Balance of effects	Does not favour either
Resources required	Moderate costs
Certainty of the evidence on required resources	No included studies
Cost-effectiveness	Varies
Equity	Varies
Acceptability	Varies
Feasibility	Probably yes

B.4 INFANT GROWTH AND DEVELOPMENT

Background

In this section of the guideline, the GDG considered the evidence and other relevant information to inform one recommendations on whole-body massage to promote newborn growth and development.

Body massage refers to systematic, tactile stimulation of the body using hands, involving rubbing and gentle, slow stroking of body parts. Massage can be done using different techniques, with or without the application of oils, such as nut, mineral and vegetable oils *(228)*. Body massage serves to improve circulation and soothe the peripheral and central nervous system *(229)*. Massage has been shown to increased vagal activity *(230)*, which leads to decreased cortisol and catecholamine levels *(231)*. This effect provides a rationale for the stress-reducing properties of massage. Increased vagal activity also increases secretion of insulin and gastrin, which promotes nutrient absorption, and increases bowel movements and hence stool frequency, which reduces bilirubin circulation. The tactile stimulation provided by the massage might contribute to a better neonatal experience that could help with overall development *(229)*. Massage has been found to promote soothing behaviour in infants and better parent–infant interactions *(232)*.

In addition to the GDG recommendation on the above, this section of the guideline includes four recommendations on improving early childhood development that have been integrated from existing WHO guidelines relevant to routine postnatal care.

B.4.1 Whole-body massage

RECOMMENDATION 37

Gentle whole-body massage may be considered for term, healthy newborns for its possible benefits to growth and development. *(Recommended)*

Remarks

- In this context, gentle whole-body massage refers to using hands for tactile stimulation, with rubbing and slow stroking of body parts or a passive range of motion across limb joints, with or without emollients.
- In making this recommendation, the Guideline Development Group (GDG) considered the effects of whole-body massage on length, weight and head circumference to be large, clinically meaningful and of critical importance for the newborn. The GDG acknowledged that evidence was of low to very low certainty and the biological mechanisms for the large effects are unclear.
- There is insufficient evidence on the effectiveness of the use of emollients for massage, type of provider, frequency and duration of sessions, length of intervention and techniques of massage. However, the GDG agreed that the use of emollients might facilitate massage.
- In most of the trials evaluated, the whole-body massage was given for 10 to 20 minutes per day for six to eight weeks by the mother after initial training.
- Babies' reactions to whole-body massage must be respected in line with the principles of responsive caregiving and respectful care. Massage should be used as an important opportunity to promote parent–infant interaction and stimulation for early childhood development.

Summary of evidence and considerations

Effects of the interventions (EB Table B.4.1)

Evidence was derived from a systematic review of the effect of body massage on growth and neurodevelopment in term, healthy newborns *(233)*. The review included 30 RCTs and quasi-RCTs involving 3826 newborns. Trials were conducted in Canada (1), China (19), the Islamic Republic of Iran (3), Israel (1), Japan (1), Turkey (2) and the USA (3). Most of the included trials used a two-arm design (3 558 newborns), one trial used a three-arm design (76 newborns), and two trials (192 newborns) used a four-arm design for studying the effects of multimodal and combined stimulation. Where reported, massage commenced from birth, within 24 hours of birth, within 48 hours of birth, after five days from birth, or after the second week from birth. All trials involved application of whole-body massage with stroking and passive range of motion across the limb joints, with or without the use of oil.

Comparison: Whole-body massage compared with no massage

Newborn/infant outcomes

Growth: It is uncertain whether newborn whole-body massage has any effect on newborn weight at the end of the intervention period, or weight at follow-up at 8–12 months, when compared with no massage (very low-certainty evidence). Low-certainty evidence suggests newborn whole-body massage may increase infant length at the end of intervention period when compared with no massage (8 trials, 1260 newborns; MD 1.53 cm longer, 1.37 cm to 1.70 cm longer). It is uncertain whether newborn whole-body massage has any effect on infant length at 12 months follow-up when compared with no massage (very low-certainty evidence). It is uncertain whether newborn whole-body massage has any effect on infant head circumference at the end of intervention period or head circumference at six months follow-up when compared with no massage (very low-certainty evidence).

Severe neonatal/infant morbidity: It is uncertain whether newborn whole-body massage has any effect on newborn bilirubin levels at four days after birth when compared with no massage (very low-certainty evidence).

Sleep characteristics: It is uncertain whether newborn massage has any effect on newborn sleep duration at the end of intervention period or at 24 months follow-up when compared with no intervention (very low-certainty evidence).

Neurodevelopment: It is uncertain whether newborn whole-body massage has any effect on infant Psychomotor Development Index scores at the end of intervention period or at 24 months follow-up when compared with no massage (very low-certainty evidence). It is uncertain whether newborn whole-body massage has any effect on infant Mental Development Index scores at the end of intervention or at 24 months follow-up when compared with no massage (very low-certainty evidence). It is uncertain whether newborn whole-body massage has any effect on infant development (gross motor, fine motor, language, personal social behaviour) at the end of the intervention period (measured using the Gesell development quotient or Capital Institute mental checklist), or at 12 months follow-up (measured using the Gesell development quotient), when compared with no massage (very low-certainty evidence).

Adverse effects were not reported in the included studies.

Maternal outcomes

Maternal functioning/well-being: It is uncertain whether newborn whole-body massage has any effect on the Maternal Attachment Inventory score when compared with routine care (very low-certainty evidence). It is uncertain whether newborn massage has any effect on crying or fussing time at the end of the intervention period, and at six months follow-up when compared with no intervention (very low-certainty evidence).

Additional considerations

A 2013 Cochrane Review, which included 34 RCTs, assessed the effect of massage on infants under 6 months of age *(234)*. The meta-analysis favoured the intervention in terms of better weight, length and

head circumference growth as well as developmental outcomes. However, the quality of evidence was rated very low for most outcomes. Twenty-four trials from the Cochrane systematic review overlap with the current review, while four new trials were conducted after the publication of this review in 2013.

Another systematic review and meta-analysis published in 2019 *(235)* evaluated the influence of massage on hyperbilirubinaemia. The review included six RCTs involving 357 full-term neonates and found significantly lower transcutaneous bilirubin after four days of massage therapy. Two trials from the current systematic review overlap with this review, while other trials from the 2019 review did not qualify for the present review as the population was jaundiced so not healthy newborns.

Values

Findings from a systematic qualitative review exploring what women want from postnatal care *(21)* indicate that women want a positive experience in which they are able to adapt to their new self-identity and develop a sense of confidence and competence as a mother. They also want to adjust to changes in their intimate and family relationships (including their relationship to their baby), navigate ordinary physical and emotional challenges, and experience the dynamic achievement of personal growth as they adjust to their new normal, both as parents and as individuals in their own cultural context.

Findings from the same review also indicate that women tend to prioritize the needs of their baby and are highly likely to value any form of therapy that enhances infant development, promotes infant sleep and reduces infant crying (high confidence in the evidence).

Resources

No economic evaluations of whole-body massage with/without oil in term newborns were identified.

Table 3.73 Main resource requirements for whole-body massage

Resource	Description
**Staff**	• Midwives/nurses to perform massage and/or to provide instruction for parents/caregivers to perform massage
**Training**	• For midwives, nurses and/or parents, training to perform infant massage from a trained massage therapist or paediatric massage consultant<br>• In most cases, the primary massage provider is likely to be the mother, parents or a family member (with instruction provided by midwives/nurses)
**Supplies**	• Massage oil (optional), such as a plant-based oil or commercially available baby oil; price varies by location and supplier<br>• Instructional brochures, videos or similar
**Equipment and infrastructure**	• Access to soap and clean water for proper hand hygiene prior to massage<br>• Baby manikin/baby care model for instruction
**Time**	• *Time to train:* varies by technique; for the Field (1986) technique, approximately 1 hour to provide instruction and observe and correct massage technique as needed *(236)*<br>• *Time to perform:* varies by technique; commonly 15–20 minute sessions 1–3 times daily for 4–5 days after birth up to several months.
**Supervision and monitoring**	• Same as for usual care

Equity

No direct evidence was identified on the impact on health equity of whole-body massage with/without oil in term newborns. Interventions among neonates and infants that promote healthy developmental outcomes could assist to address health equity. Newborn/infant massage is a relatively simply and accessible intervention across a range of settings. Provided the necessary training and support for health workers is available, this intervention may increase health equity.

Acceptability

A qualitative evidence synthesis of women's experiences of postnatal care found no direct evidence relating to women's views on the use of massage for their babies *(28)*. Indirect evidence from this review suggests that most women appreciate any advice, information and, where appropriate, therapy that might enhance their baby's comfort and well-being (high confidence in the evidence). Findings also suggest that women are likely to value the intimate moments that massage confers in the development of mother–infant attachment (moderate confidence in the evidence).

Additional considerations

Infant massage is a therapy that may be performed by parents and other caregivers. Findings from an American study in which fathers were encouraged to massage their infants for 15 minutes each evening over a three-week period highlight several benefits including, relieving infant stress (soothing), relieving maternal stress (caregiving), increased confidence, and increased contribution. In addition, fathers expressed a desire for further access and opportunities for interaction with their infants *(237)*.

Feasibility

A qualitative evidence synthesis of women's experiences of postnatal care found no direct evidence relating to women's views on the feasibility of using massage on term newborns *(28)*. Indirect evidence suggests women are likely to appreciate the practical, low-cost nature of this therapy (moderate confidence in the evidence) and the basic principles of massage are likely to be understood across a broad range of settings and contexts (moderate confidence in the evidence). A qualitative evidence synthesis of health workers' experiences of postnatal care found no direct evidence relating to views on the feasibility of using massage on term newborns *(29)*. However, indirect evidence suggests that lack of personnel, resources and training may limit provision of information and counselling on newborn massage in the postnatal period (moderate confidence in the evidence).

Table 3.74 Summary of judgements: Whole-body massage compared with no massage

Domain	Judgement
Desirable effects	Moderate
Undesirable effects	Don't know
Certainty of the evidence	Very low
Values	Probably no important uncertainty or variability
Balance of effects	Probably favours massage
Resources required	Negligible costs or savings
Certainty of the evidence on required resources	No included studies
Cost-effectiveness	Don't know
Equity	Probably increased
Acceptability	Probably yes
Feasibility	Probably yes

B.4.2 Improving early childhood development

RECOMMENDATION 38

All infants and children should receive responsive care between 0 and 3 years of age; parents and other caregivers should be supported to provide responsive care. *(Recommended)*

Remarks

- This recommendation has been adapted and integrated from the 2020 *Improving early childhood development: WHO guideline (238)*, where it was considered a strong recommendation based on moderate-certainty evidence for responsive care.
- The postnatal care Guideline Development Group noted the following based on the original guideline.
  - Responsive caregiving incorporates anticipatory guidance for safety, education, development and the establishment of a caring and understanding relationship with one's child. Parenting is not limited to biological parents but extends to guardians or caregivers providing consistent care for the child.
  - To provide responsive care for a newborn, parents and caregivers need to be aware of the newborn's signals, such as readiness for a feed, pain or stress, and be able to respond to these signals appropriately.
  - Interventions to support responsive caregiving during the postnatal period should focus on promoting positive caregiver–infant interactions and strengthening the parent–infant relationship. An emphasis should be placed on responsiveness between caregivers and the infant, and should target the caregiver–infant dyad rather than the caregivers or the child alone.
  - Health workers should encourage and support responsiveness (care that is prompt, consistent, contingent and appropriate to the child's cues, signals, behaviours and needs). Interventions that improve parents and caregivers' abilities to incorporate the child's signals and perspective can be undertaken in the context of, but not limited to, play, communication and feeding. For the newborn, they include, but are not limited to, facilitating the caregiver to be aware of, receptive and appropriately responsive to the baby's needs and wants, such as exclusive breastfeeding on demand.

RECOMMENDATION 39

All infants and children should have early learning activities with their parents and other caregivers between 0 and 3 years of age; parents and other caregivers should be supported to engage in early learning with their infants and children. *(Recommended)*

Remarks

- This recommendation has been adapted and integrated from the 2020 *Improving early childhood development: WHO guideline (238)*, where it was considered a strong recommendation based on moderate-certainty evidence for early learning.
- The postnatal care Guideline Development Group noted the following based on the original guideline.
  - Early learning refers to any opportunity for the baby, toddler or child to interact with a person, place or object in their environment, recognizing that every interaction (positive or negative, or absence of interaction) is contributing to the child's brain development and laying the foundation for later learning.
  - Activities that support early learning in the newborn period include, but are not limited to, making eye contact, smiling, talking, singing and gentle massage of the newborn infant (see Recommendation 37 in this guideline). Responding to the child's signals as discussed above also promotes early learning.
  - Health workers should enhance parents' and caregivers' knowledge, attitudes, practices or skills with respect to supporting early learning and development during the postnatal period. These interventions may either: (i) directly support caregivers in providing new early learning opportunities for their children; or (ii) build caregiver capacities more generally, providing information and guidance around healthy newborn/child development or a range of nurturing care topics.

RECOMMENDATION 40

Support for responsive care and early learning should be included as part of interventions for optimal nutrition of newborns, infants and young children. *(Recommended)*

Remarks

- This recommendation has been adapted and integrated from the 2020 *Improving early childhood development: WHO guideline* (238), where it was considered a strong recommendation based on moderate-certainty evidence.
- The postnatal care Guideline Development Group noted the following based on the original guideline.
  - Responsive feeding is a part of responsive caregiving and is essential to adequate nutrition. To thrive, nutrition interventions alone are not enough to improve child development, but they have an impact on young children's development, particularly when combined with responsive caregiving and opportunities for early learning. For the newborn, exclusive breastfeeding on demand is a form of responsive feeding.
  - Health workers should support mothers to exclusively breastfeed their infant on demand, while encouraging and supporting sensitivity and responsiveness (care that is prompt, consistent, contingent on, and appropriate to the child's cues, signals, behaviours and needs) and secure attachment.
  - In the postnatal period, interventions for optimal nutrition can be enhanced by including guidance on making eye contact, smiling, talking, singing and gentle massage of the newborn infant (see Recommendation 37 in this guideline), during feeding times and beyond.

RECOMMENDATION 41

Psychosocial interventions to support maternal mental health should be integrated into early childhood health and development services. *(Recommended)*

Remarks

- This recommendation has been integrated from the 2020 *Improving early childhood development: WHO guideline* (238), where it was considered a strong recommendation based on moderate-certainty evidence.
- The postnatal care Guideline Development Group noted the following based on the original guideline.
  - Psychosocial interventions for common mental disorders in the postpartum period (depression and anxiety) should be provided (see Recommendations 18 and 19 in this guideline). These include routine enquiry about the mother's mental health and social well-being, and psychosocial support as part of every postnatal consultation, combined with referral to a skilled provider for conditions that require more intensive support, through strategies such as psychoeducation, cognitive behavioural therapy and interpersonal psychotherapy. Early childhood learning and postnatal services are important avenues to provide interventions for the prevention and treatment of maternal mental health conditions.
  - In addition, fathers/partners/caregivers should also be included in such interventions in order to target relevant risk factors for maternal and child health (e.g. intimate partner violence and lack of involvement of fathers in parental care) (see Recommendation 52 in this guideline).

B.5 BREASTFEEDING

Background

This section of the guideline includes two sets of recommendations that have been integrated from WHO guidelines on breastfeeding that are relevant to routine postnatal care.

B.5.1 Exclusive breastfeeding

RECOMMENDATION 42

All babies should be exclusively breastfed from birth until 6 months of age. Mothers should be counselled and provided with support for exclusive breastfeeding at each postnatal contact. *(Recommended)*

Remarks

- This recommendation has been integrated from the 2014 *WHO recommendations on postnatal care of the mother and newborn (15)*, where it was considered a strong recommendation based on moderate-certainty evidence for neonatal outcomes.
- The postnatal care Guideline Development Group noted the following based on existing WHO documents.
  - Breastfeeding counselling should be provided in both the antenatal period and postnatally, as per existing WHO guidelines *(75, 76)*.
  - All mothers should be supported to initiate breastfeeding within the first hour after birth. Mothers should receive practical support to enable them to initiate and establish breastfeeding and manage common breastfeeding difficulties *(75)* (see Box 3.13).
  - Some exceptions to exclusive breastfeeding for term newborns are mentioned in the WHO document *Acceptable medical reasons for use of breast-milk substitutes (239)*. These exceptions include: infants with classic galactosaemia, infants with maple syrup urine disease and infants with phenylketonuria.

B.5.2 Protecting, promoting and supporting breastfeeding in facilities providing maternity and newborn services

RECOMMENDATION 43a

Facilities providing maternity and newborn services should have a clearly written breastfeeding policy that is routinely communicated to staff and parents. *(Recommended)*

RECOMMENDATION 43b

Health-facility staff who provide infant feeding services, including breastfeeding support, should have sufficient knowledge, competence and skills to support women to breastfeed. *(Recommended)*

Remarks

- These recommendations have been integrated from the 2017 WHO guideline, *Protecting, promoting and supporting breastfeeding in facilities providing maternity and newborn services (75)*, where the overall certainty of evidence was judged to be very low.
- The postnatal care Guideline Development Group noted the following based on existing WHO documents.
  - These recommendations provide an enabling environment for sustainable implementation of the *Ten steps to successful breastfeeding* within health facilities and should be accompanied by the establishment of ongoing monitoring and data-management systems *(240)*.
  - Facilities providing maternity and newborn services should fully comply with the *International code of marketing of breast-milk substitutes* and relevant World Health Assembly resolutions *(241)*.
  - Additional recommendations on key clinical practices for women and newborns to successfully establish and maintain breastfeeding are included in the 2017 WHO guideline *Protecting, promoting and supporting breastfeeding in facilities providing maternity and newborn services (75)* (see Box 3.13).

Box 3.13 Key clinical practices to successfully establish and maintain breastfeeding *(75)*

Discuss the importance and management of breastfeeding with pregnant women and their families.

Facilitate immediate and uninterrupted skin-to-skin contact, and support mothers in initiating breastfeeding as soon as possible after birth.

Support mothers to initiate and maintain breastfeeding and manage common difficulties.

Do not provide breastfed newborns with any food or fluids other than breastmilk, unless medically indicated.

Enable mothers and their infants to remain together and to practise rooming-in 24 hours a day.

Support mothers to recognize and respond to their infants' cues for feeding.

Counsel mothers on the use and risks of feeding bottles, teats and pacifiers.

Coordinate discharge so that parents and their infants have timely access to ongoing support and care.

C Health systems and health promotion interventions

Background

High-quality postnatal care comprises not only the discrete clinical interventions offered to women and newborns, but the broader health system structures, systems, processes, policies and innovations through which care is delivered. This section of the guideline describes health systems and health promotion interventions to improve the provision, utilization and experience of postnatal care. Specifically, the GDG considered the evidence and other relevant information to inform recommendations relating to length of stay and discharge from health facilities, frequency and place of postnatal care contacts, and involvement of men in care.

Schedules and place of postnatal care contacts

Routine postnatal care services provide a delivery platform for care of healthy women and their healthy newborns. It is provided at different time windows, often by different health workers in different places *(6)*. Despite its importance, coverage and quality of postnatal care for women and newborns tend to be relatively poor *(12)*. Routine postnatal care is provided in outpatient settings (office or health facilities) or at home. Its purpose is to assess the health and well-being of the women and newborns, to provide breastfeeding and hygiene counselling, parenting and child health counselling and general support to families, and to facilitate access to timely care. In addition, home visits provide an opportunity to assess the home environment. Different modalities of postnatal home visits have been introduced, mainly to ensure early postnatal care contacts for women and newborns, either after discharge from health facilities *(242, 243)*, to replace or complement outpatient postnatal care contacts, or as part of community-based packages, particularly in rural areas *(244)*.

Length of stay in, and discharge from, health facilities

Length of stay in health facilities after childbirth varies widely between countries, from a few hours to up to 6 days for singleton vaginal births, and from 2.5 to 9 days following caesarean births *(11)*. There has been a steady decline in the length of time woman–infant dyads spend in health facilities after birth. Shorter postnatal stays have been promoted in some settings to decrease medicalization of the immediate postnatal period and to increase maternal–infant bonding and satisfaction. However, concerns have been raised that early discharge from health facilities may delay identification and early management of maternal and newborn complications and lead to insufficient time to prepare women and families for the transition to the home, particularly if there is insufficient or absent support in the community *(242)*. Observational studies have assessed the relationship between length of postnatal hospital stay for healthy women and newborns and related outcomes, showing conflicting results on neonatal mortality, infant readmissions, and breastfeeding initiation and duration *(242)*.

Decision-making around readiness for discharge is complex and must consider not only the physical condition of the woman and newborn to identify health risks, but also the woman's birth experience, the woman and parents' learning needs, and their ability to support the care of the mother and newborn *(245, 246)*. Discharge readiness is linked to the quality of discharge preparation, which highlights the critical role that health workers play in the discharge preparation process *(247)*.

Involvement of men in postnatal care and maternal and newborn health

Involving men in supporting the care of their partners and newborn children, throughout pregnancy, childbirth and after birth, is promoted as an effective strategy to improve maternal and newborn health outcomes. Men's practical, emotional and financial support can help women and newborns to access essential health services, and provide support to the woman in caring for herself as well as improving family care practices *(248)*. Evidence further indicates that working with men in their roles as partners/husbands and fathers can provide an entry point for supporting gender-transformative change within

households and couple relationships *(249)*. Since the publication in 2015 of WHO recommendations on the involvement of men as an effective intervention to facilitate and support improved maternal, newborn and child health *(250)*, a number of systematic reviews and two major WHO-supported evidence mapping exercises have been conducted that have helped to identify and organize this growing evidence base *(251, 252)*.

In addition to the GDG recommendation on the above, this section of the guideline includes six sets of recommendations on other health systems and health promotion interventions that have been integrated from WHO guidelines that are relevant to routine postnatal care.

Box 3.14 Values

Findings from a qualitative evidence synthesis exploring what women want from postnatal care *(21)* indicate that women want a positive experience in which they are able to adapt to their new self-identity and develop a sense of confidence and competence as a mother. They also want to adjust to changes in their intimate and family relationships (including their relationship to their baby), navigate ordinary physical and emotional challenges, and experience the dynamic achievement of personal growth as they adjust to their new normal, both as parents and as individuals in their own cultural context (moderate to high confidence in the evidence).

C.1 Schedules for postnatal care contacts

RECOMMENDATION 44

A minimum of four postnatal care contacts is recommended.

If birth is in a health facility, healthy women and newborns should receive postnatal care in the facility for at least 24 hours after birth. If birth is at home, the first postnatal contact should be as early as possible within 24 hours of birth. At least three additional postnatal contacts are recommended for healthy women and newborns, between 48 and 72 hours, between 7 and 14 days, and during week six after birth. *(Recommended)*

Remarks

- The number, timing and content of postnatal care contacts should be tailored to each woman's and newborn's health outcomes and needs, and guided by the recommendations in this guideline, including:
  - the woman's and newborn's physical well-being and the woman's emotional well-being;
  - the skills and confidence of the woman to care for herself and the skills and confidence of parents/caregivers/family to care for the newborn;
  - the home environment and other factors that may influence the ability to provide care for the woman and newborn in the home, and care-seeking behaviour; and
  - the place of birth and the time of discharge from the health facility for a facility-based birth.
- In making this recommendation, the Guideline Development Group considered the following.
  - There was insufficient evidence from randomized controlled trials on the effects of more frequent postnatal care contacts compared with less frequent contacts.
  - Epidemiological data shows that most maternal and neonatal deaths occur in the first three days after birth, in particular on the day of birth, with another increase during the second week after birth.
  - Transition to well-woman and well-infant care will usually occur around week six after birth, including referrals to infant immunization and family planning services.
- Postnatal care contacts, in particular during the first week, can occur at home or in outpatient services (e.g. the health facility or health worker's office) by skilled health personnel or trained community health workers as per Recommendation 48 in this guideline, according to the preferences of women/parents/caregivers and the organization of services as per the health system.
- Continuity of care is valued by women and health workers to establish supportive, caring and trusting relationships and improve experience of care. In settings with well-functioning midwifery programmes, midwife-led continuity-of-care models provide an opportunity to ensure continuity of postnatal care, as per Recommendation 49 in this guideline.
- Routine postnatal care contacts may be complemented by phone-based follow-up or the use of digital targeted communication, as per Recommendation 54 in this guideline.

Summary of evidence and considerations

Effects of the interventions (EB Table C.1)

Evidence was derived from an updated Cochrane systematic review assessing maternal and newborn outcomes of different home visiting schedules during the early postpartum period *(243)*. The review included 16 RCTs involving 11 718 women, of which three trials were considered in this evidence summary; two RCTs (1562 women) and one quasi-RCT (200 women). Trials were conducted in Spain, the USA, and Zambia.

The trials evaluated fixed schedules of home visits, from one home visit within three or five days after birth up to four visits on a fixed schedule (3, 7, 28 and 42 days), compared with one midwife home visit at about 42 days (408 women), usual office-based visits for the baby (1154 women), or health centre check-ups (200 women) 5–14 days after birth, arranged by the hospital newborn nursery (1154 women). One trial included full-term woman–infant dyads without risk factors who had had a vaginal birth (408 women), and two trials included women after vaginal or caesarean births (1354 women). Parity,

gestational age or risk factor status was not reported in the other trials.

Comparison 1: Schedules involving four postnatal home visits (3, 7, 28 and 42 days after birth) compared with one postnatal home visit (at about 42 days after birth)

Maternal outcomes

Short-term maternal morbidity: It is uncertain whether schedules involving four home visits have any effect on maternal health problems (as identified by a doctor) when compared with one home visit (very low-certainty evidence).

Maternal mortality, health service use, maternal functioning/well-being, experience of postnatal care and *long-term maternal morbidity* were not reported in the included trial. Cost was not reported in the systematic review.

Newborn/infant outcomes

Neonatal/infant mortality: It is uncertain whether schedules involving four home visits have an effect on neonatal mortality when compared with one home visit (very low-certainty evidence).

Severe neonatal morbidity: It is uncertain whether schedules involving four home visits have any effect on infant respiratory tract infection within 42 days of birth when compared with one home visit (very low-certainty evidence).

Health service use: Low-certainty evidence suggests schedules involving four home visits may reduce infant referrals to paediatricians within 42 days of birth when compared with one home visit (1 trial, 352 newborns; RR 0.41, 95% CI 0.28 to 0.60).

Breastfeeding status: Low-certainty evidence suggests schedules involving four home visits may increase exclusive breastfeeding up to six weeks when compared with one home visit (1 trial, 352 newborns; RR 1.13, 95% CI 1.05 to 1.22).

Long-term neonatal morbidity and *growth* were not reported in the systematic review.

Comparison 2: Schedules involving two postnatal visits (3–5 and 10–14 days after birth) versus one outpatient visit (10–14 days after birth)

Maternal outcomes

Maternal mortality, short-term morbidity, health service use, maternal functioning/well-being and *long-term maternal morbidity* were not reported in the included trial. Experience of postnatal care was not reported separately for this trial in the systematic review subgroup analysis.

Newborn/infant outcomes

Breastfeeding status: It is uncertain whether schedules involving two postnatal care visits have any effect on discontinuation of breastfeeding (up to 30 days) when compared with one postnatal care visit (very low-certainty evidence). Moderate-certainty evidence suggests that schedules involving two postnatal care visits probably have little or no effect on any breastfeeding (last assessment up to six months) when compared with one postnatal care visit (1 trial, 1000 newborns; RR 1.09, 95% CI 1.00 to 1.18).

Neonatal/infant mortality, severe neonatal morbidity, health service use, long-term neonatal morbidity and *growth* were not reported in the included trial.

Additional considerations

A structured search identified no trials comparing more frequent versus less frequent outpatient postnatal care contacts (whether facility-based, clinic-based, or office-based).

Some studies have investigated optimal timing of the first postnatal care contact after birth discharge. One trial excluded from the Cochrane systematic review conducted in Australia (475 women) randomized women to postnatal check-up by a general practitioner of the woman's choice at one week after discharge ("early") or six weeks after birth ("standard") *(253)*. All women also received a home visit within a few days of discharge from hospital by a maternal and child health nurse. The trial did not find any statistically significant differences between early and standard general practitioner visits in full breastfeeding at three (46.3% vs 51.4%) or six months (29.3% vs 34.5%), mean EPDS score (7.38 [5.31] vs 7.48 [5.35]), moderate or severe depression defined as EPDS ≥ 13 (16.6% vs 13.6%) or additional visits to a general practitioner (mean 1.2 vs 1.1 visits). A review on optimal timing of first postnatal visits by skilled personnel in HICs identified two cross-

sectional studies, published from 2000, comparing postnatal routine neonatal examinations *(254)*. One study investigated visits at 6–48 hours versus 49–72 hours after birth, the other study investigated visits within three days (if length of stay was ≥ 48 hours) or five days (if length of stay was > 48 hours) of discharge versus neonatal examinations after three or five days. Studies found no clinically important difference in the neonatal readmission rate 28–30 days after birth (very low-certainty evidence). One study (79 720 newborns) found a clinically important reduction in readmission rate for jaundice (unspecified timeframe) between those receiving an early well-child visit within three or five days (depending on the length of stay after birth) versus a later visit (very low-certainty evidence).

Given limited evidence on optimal number and timing of postnatal care contacts, a systematic review on daily and weekly distribution of maternal and neonatal mortality in the postnatal period was conducted *(255)*. Distribution of postpartum maternal deaths was 48.9% on the day of birth (day 0), 24.5% between days 1 and 7, and 24.9% between days 8 and 42 after childbirth (26 studies, > 1 530 964 live births, 6142 postpartum maternal deaths). Neonatal deaths during the first week account for three fourths of all neonatal deaths 71.9%, 13.82% neonatal deaths occurred during the second week, 8.13% during the third week, and 5.07% neonatal deaths occurred during the fourth week up to 28 days (16 studies, > 5 628 926 live births, 22 840 neonatal deaths). Distribution of neonatal deaths within the first week was 38.8% on day 1, 12.3% on day 2, 8.99% on day 3, 5.7% on day 4, 3.9% on day 5, 2.91% on day 6 and 2.7% on day 7 (34 studies, > 6 539 342 live births, 42 276 neonatal deaths).

A modelling study estimated a proportion of neonatal deaths of 0.73 (uncertainty range 0.72–0.74) in the first week after birth, of which deaths on day 1 represented 0.36 (uncertainty range 0.34–0.38) in 2013 for 186 countries. The predicted proportions of neonatal deaths on the day of birth (day 0) and within one week were consistent across countries with different neonatal mortality rates and income, and in different regions *(256)*.

Another modelling study predicted the proportions of cause-specific neonatal deaths in the early (1–7 days of age) and late (8–28 days of age) neonatal periods, for 194 countries between 2000 and 2013 *(257)*. The leading causes of neonatal death are the same for the early and late neonatal periods, but their distribution differs between the early period (prematurity [40.8%] intrapartum complications [27.0%] infections [14.3%]) and the late period (infections [47.6%], prematurity [21.2%] and intrapartum complications [12.9%]). The distribution of neonatal deaths in the early and late neonatal periods is similar for congenital disorders (10.6% and 10.2%) or those classified as having other causes (7.3% and 8.1%).

Values

See Box 3.14 in section 3.C: Health systems and health promotion interventions.

In addition, evidence from a qualitative evidence synthesis exploring what women want from postnatal care *(21)* indicates that women may experience periods of low mood, loneliness, anxiety and fatigue during the postnatal period (moderate confidence in the evidence) and appreciate the advice, reassurance and support (practical and emotional) they receive from health workers and family members during this time (high confidence in the evidence). Some women may struggle with labour- and birth-induced trauma (physical and psychological) (high confidence in the evidence) and/or experience difficulties with breastfeeding or find it difficult to embrace their maternal identity (moderate confidence in the evidence), so are likely to value regular engagement with health workers to resolve these concerns. Further, women tend to prioritize the needs of their baby during the postnatal period (moderate confidence in the evidence), so are likely to value clinical and developmental outcomes associated with their infant.

Resources

No economic evaluations of more frequent compared with less frequent postnatal care contacts were identified.

Additional considerations

Schedules of postnatal care involving more contacts may increase the cost of services compared with a reduced number of contacts. Time spent by staff providing usual care and women accessing care will be shorter, with reduced contact schedules.

Table 3.75 Main resource requirements for more frequent compared with less frequent postnatal care contacts

Resource	Description
**Staff**	• Designated, trained, motivated staff for postnatal care of women and newborns in facilities/clinics/office or at home<br>• Additional personnel might be required to conduct postnatal home visits
**Training**	• Same as regular practice-based training for health workers<br>• Might require additional personnel and education or training sessions for staff conducting postnatal home visits
**Supplies**	• Same supplies regardless of number of postnatal care contacts<br>• Same medical supplies (e.g. painkillers, iron tablets, contraceptives, anthelminthics, mosquito nets, gloves)<br>• For home visits, community/household registers to record findings of the visits, and referral slips, counselling cards or flip chart<br>• Staff supplies (e.g. boots, umbrella and bag)<br>• Home-based records<br>• Information cards for women/parents/caregivers with home visitor contact information
**Equipment and infrastructure**	• Functional birth notification system for staff performing the home visits or scheduling outpatient contacts<br>• For outpatient contact, same basic and adequate equipment for postnatal care that is available in sufficient quantities at all times<br>• Clean, comfortable waiting room for women and their companions<br>• Clean, private examination room<br>• For postnatal home visits, same portable equipment to conduct home visits (e.g. weighing scales, thermometer, clock/timer, bag and mask)<br>• Access to transport to conduct postnatal home visits (e.g. bicycle or motor vehicle)
**Time**	• Additional staff time for more frequent postnatal care of women and newborns in facilities/clinics/office or at home<br>• Additional transport time to facility/office for outpatient contact or transport time to client's home for postnatal home visits
**Supervision and monitoring**	• Regular supportive supervision and review by supervisors and coordination meetings between health facilities/districts, outpatient services and staff conducting postnatal home visits<br>• Systems for follow-up after discharge from health facility (integration of midwives or nurses into home-based postnatal care, establishing networks of health workers, integration of facility and community postnatal care)<br>• Systems to report stock-outs of supplies for postnatal home visitors

Equity

No direct evidence was identified on the impact on health equity of more frequent compared with less frequent postnatal care contacts.

Schedules of postnatal care involving more contacts may increase equity, particularly if they ensure coverage among woman–infant dyads from low socioeconomic groups or in rural areas, who are less likely to receive postnatal care and have higher rates of preventable maternal, perinatal and neonatal mortality. However, these schedules may decrease equity if coverage is lower in low socioeconomic groups, in rural areas, or if women and families are expected to cover the cost of more frequent postnatal care contacts.

Acceptability

Evidence from a qualitative evidence synthesis exploring women's experiences of postnatal care *(28)* indicates that women appreciate and value the practical, psychosocial and emotional support they receive from health workers during the postnatal period (high confidence in the evidence). Some women, particularly in HICs, feel that they want more support from health workers during the postnatal period as well as flexible contact opportunities, including regular home visits, drop-in clinics, out-of-hours services and telephone availability (moderate confidence in the evidence). Where regular postnatal contact services are already in place, women appreciate being seen by the same care provider to establish trust and familiarity with

personal circumstances (moderate confidence in the evidence).

In some LMIC settings, women only engage with postnatal services when they feel there is something wrong with themselves or their babies (moderate confidence in the evidence). Evidence also indicates that some women, particularly in LMICs, are unlikely to welcome additional engagement with postnatal providers in circumstances where the additional costs outweigh the perceived benefits, where there is a lack of trust in formal health systems, where women perceive there to be a culture of mistreatment or where adherence to traditional postnatal practices may be disrupted (moderate confidence in the evidence).

Feasibility

A qualitative evidence synthesis exploring women's experiences of postnatal care *(28)* found no direct evidence relating to the feasibility of increasing the number of postnatal contacts. Indirect evidence indicates that the proximity of the health facility may encourage additional engagement with postnatal services, particularly in LMICs; that is, geographical closeness facilitates access for women and community engagement by health workers (low confidence in the evidence). Evidence also suggests the costs associated with extra visits to the health facility may limit access for some women, while the health system resources required (additional staff, travelling costs) to provide home visits may be prohibitive in some LMIC settings (low confidence in the evidence).

A qualitative evidence synthesis of health workers' experiences of postnatal care found no direct evidence relating to views on the feasibility of increasing the number of postnatal contacts *(29)*. However, indirect evidence suggest that lack of personnel, resources and training may limit the availability and quality of postnatal care services (moderate confidence in the evidence). Sometimes, health workers had to prioritize some services or care over others (low confidence in the evidence). Providers appreciated continuity of care to be able to establish trusting relationships, assess women's emotional well-being and improve their experience of care (moderate confidence in the evidence). By contrast, lack of continuity of care or common policies or guidelines across different cadres and levels of maternal health services may limit the offer of consistent information and counselling (moderate confidence in the evidence). Providers believed postnatal/parental education was sometimes superficial and provided too late (low confidence in the evidence).

Table 3.76 Summary of judgements: More frequent compared with less frequent postnatal care contacts

Domain	Judgement
Desirable effects	Small
Undesirable effects	Don't know
Certainty of the evidence	Very low
Values	Probably no important uncertainty or variability
Balance of effects	Probably favours more frequent contacts
Resources required	Moderate costs
Certainty of the evidence on required resources	No included studies
Cost-effectiveness	Don't know
Equity	Probably increased
Acceptability	Varies
Feasibility	Varies

C.2 Length of stay in health facilities after birth

RECOMMENDATION 45

Care for healthy women and newborns in the health facility is recommended for at least 24 hours after vaginal birth. *(Recommended)*

Remarks

- Despite insufficient evidence, the Guideline Development Group (GDG) acknowledged that it was important to establish a minimum time before discharge in light of the wide variation in length of stay after birth *(11)*, including lengths of stay that were considered too short for the delivery of health facility interventions recommended in this guideline.
- The GDG acknowledged that timing of discharge from the health facility should be guided by the following.
  - The time needed to complete the assessment of a comprehensive set of criteria to evaluate maternal and newborn well-being and needs, and the findings of these assessments, as per Recommendation 46 in this guideline.
  - The health system's capacity to organize postnatal care contacts after discharge through community-based services (e.g. home visits) or in outpatient services (e.g. in the health facility or provider's office). Most healthy women and newborns would be ready for discharge 24 hours after birth, provided functioning and accessible follow-up services are available.
  - Unnecessarily prolonged stays in health facilities after birth should be avoided considering the increased risk of healthcare-associated infections, costs to the health system and to service users, and women's and families' preferences.
- Given the paucity of evidence, the GDG was not able to recommend a minimum time of care in the health facility after caesarean birth, but noted that discharge within 24 hours after caesarean birth increased the risk of adverse maternal and neonatal outcomes and reduced breastfeeding at six weeks. The content of postnatal discharge criteria and discharge preparation would also need to consider post-operative outcomes and the needs of women and newborns after a caesarean birth.
- As part of birth preparedness and complication readiness during pregnancy, women/parents/caregivers should be informed that stay in the heath facility after birth is recommended for a minimum of 24 hours. However, length of stay in the health facility will depend on individual health outcomes and needs, particularly after a caesarean birth, and the availability of postnatal care services for follow-up after discharge.

Summary of evidence and considerations

Effects of the interventions (EB Table C.2)

Evidence was derived from an updated Cochrane systematic review on policies of early postnatal discharge from hospital for healthy women and term infants *(242)*, including 17 RCTs with 9409 women. Data on timing of discharge from 15 trials, reported by mode of birth (vaginal or caesarean birth), are included in this evidence summary.

The timing of discharge and intensity of antenatal, in-hospital (pre-discharge) and post-discharge interventions offered to the woman–infant dyads in the intervention groups differed considerably between trials. Standard discharge policies in the comparisons groups also varied greatly.

The evidence and judgements are presented separately by mode of birth, based on a subgroup analysis from the Cochrane systematic review.

Comparison 1: Early discharge following vaginal birth compared with usual discharge

Ten studies (3553 women), published between 1962 and 2005, included only women after vaginal births, all conducted in HICs. All trials' eligibility criteria were designed to limit the participation to women at low risk of complications. Six trials recruited women during the antenatal period, and four recruited women after childbirth. Women in the intervention arms were discharged up to 24 hours in three trials, between 24 and 48 hours in five trials, and after 48 hours in three trials. Usual hospital stay also varied according to the standard local practices, ranging

from 37 to 72 hours in four trials, four to five days in three trials, and six to nine days in two trials, or else not described. Eight trials reported co-interventions, of which three included antenatal activities.

Maternal outcomes

Short-term maternal morbidity: It is uncertain whether a policy of early discharge after vaginal birth has any effect on the number of women scoring above the cut-off score indicating probable postpartum depression within six months when compared with usual discharge (very low-certainty evidence).

Health service use: It is uncertain whether a policy of early discharge after vaginal birth has any effect on maternal readmission within six weeks when compared with usual discharge (very low-certainty evidence).

Experience of postnatal care: Low-certainty evidence suggests that a policy of early discharge (with two to five follow-up home visits) after vaginal birth may improve women's satisfaction with postnatal care (continuous data) when compared with usual discharge (2 trials, 306 women; SMD 0.74 higher, 95% CI 0.5 higher to 0.98 higher). It is uncertain whether a policy of early discharge after vaginal birth has any effect on the number of women who perceive their length of hospital stay as too short or too long when compared with usual discharge (very low-certainty evidence).

Maternal mortality and *maternal functioning/well-being* were not reported in the included trials. Cost outcomes are reported under Resources.

Newborn outcomes

Neonatal mortality: It is uncertain whether a policy of early discharge after vaginal birth has any effect on the risk of infant mortality within 28 days when compared with usual discharge (very low-certainty evidence). It is uncertain whether a policy of early discharge after vaginal birth has any effect on the risk of infant mortality within one year when compared with usual discharge (very low-certainty evidence).

Health service use: It is uncertain whether a policy of early discharge after vaginal birth has any effect on readmission for neonatal morbidity within seven days when compared with usual discharge (very low-certainty evidence). It is uncertain whether a policy of early discharge after vaginal birth has any effect on readmission for neonatal morbidity within 28 days when compared with usual discharge (very low-certainty evidence).

Breastfeeding status: It is uncertain whether a policy of early discharge after vaginal birth has any effect on the number of women breastfeeding (exclusively or partially) at six weeks postpartum when compared with usual discharge (very low-certainty evidence). Moderate-certainty evidence suggests a policy of early discharge (with one home visit) after vaginal birth probably increases the number of women breastfeeding (exclusively or partially) at 12 weeks postpartum when compared with usual discharge (1 trial, 430 participants; RR 1.21, 95% CI 1.03 to 1.41).

Severe neonatal morbidity was not reported in the included trials.

Additional considerations

Additional ad-hoc analyses were conducted to complement this evidence summary, to assess the effects of a policy of early discharge within 24 hours versus later discharge by mode of birth (within 48 hours of vaginal birth or within 72 hours of caesarean birth; see Web Supplement).[46]

A systematic review *(258)* identified five population-based interrupted time series analyses of changes in policies of timing of postnatal discharge introduced in the 1990s in Denmark and the USA. This review suggests that a postnatal hospital stay of < 48 hours after vaginal birth or < 96 hours after caesarean birth is associated with increased infant readmission to the hospital within 28 days of birth and unscheduled postnatal care contacts.

A systematic review on daily and weekly distribution of maternal and neonatal mortality in the postnatal period *(255)* reported the distribution of all maternal deaths (up to 42 days postpartum) was 48.9% on day 1, and 24.5% between days 2 and 7 (26 studies, > 1 530 964 live births, 6142 postpartum maternal deaths). Neonatal deaths during the first week account for three fourths (71.9%) of all neonatal deaths (16 studies, > 5 628 926 live births, 22 840 neonatal deaths). The distribution of neonatal deaths within the first week was 38.8% on day 1, 12.3% on day 2, 8.99% on day 3, 5.7% on day 4, 3.9% on

46 The Web Supplement is available at: https://www.who.int/publications/i/item/9789240045989

day 5, 2.91% on day 6, and 2.7% on day 7 (34 studies, > 6 539 342 live births, 42 276 neonatal deaths).

A modelling study estimated a proportion of neonatal deaths to be 0.73 (uncertainty range 0.72–0.74) in the first week after birth, of which deaths on day 1 represented 0.36 (uncertainty range 0.34–0.38) in 2013 for 186 countries. The predicted proportions of neonatal deaths on the day of birth (day 0) and within week one were consistent across countries with different neonatal mortality rates and income, and in different regions *(256)*.

Values

See Box 3.14 in section 3.C: Health systems and health promotion interventions.

In addition, findings from a qualitative evidence synthesis exploring what women want from postnatal care *(21)* indicate that women want to form an immediate relationship with their baby (moderate confidence in the evidence) and value the practical and emotional support offered by health workers to enable mother–baby bonding (high confidence in the evidence). Women appreciate tailored care and support during the immediate postnatal period, including an understanding that informational needs, as well as individual capacity to assimilate information, may vary from woman to woman (moderate confidence in the evidence). In some settings, perceived staff shortages and/or the disruptive nature of postnatal wards (lack of privacy, hospital visits, ward rounds) may lead to inadequate care and a perception among some women that they are discharged too early from a health facility feeling ill-prepared and lacking in confidence about their ability to cope at home (moderate confidence in the evidence).

Findings from a qualitative evidence synthesis on the perspectives of women, men and health workers related to postnatal hospital discharge *(91)* indicate that women value postnatal education for themselves and their partners and families to ensure parental confidence, and the opportunity to practice care with the support of a midwife (moderate confidence in the evidence). Both women and men value their autonomy in relation to the discharge process (moderate confidence in the evidence), including decisions regarding the timing of the discharge, adequate recognition by staff at the hospital, and availability of care without direct interference and intrusiveness.

Resources

No economic evaluations of policies of early discharge from health facilities after term, uncomplicated vaginal birth were identified. One trial *(259)* reported costs of hospital care between the period immediately following vaginal birth up to the time of discharge as US$ 382.22 (213 women) in the early discharge group (24 hours or less with one home visit) and US$ 647.67 (217 women) in the late discharge group (at least 48 hours). The same trial reported a reduced combined cost of community care and maternal and neonatal readmissions for the early discharge group, where costs were US$ 28.66 less.

Additional considerations

A cost-minimization analysis *(260)* reported early postnatal discharge after vaginal or caesarean birth combined with home midwifery support resulted in a significant cost saving (hospitalization and community care, and non-medical costs) per mother–infant dyad in the early discharge group (24 hours or less) compared with the late discharge group (at least 48 hours). There were no significant differences in average hospital readmission, hospital outpatient care, or direct non-medical or indirect costs.

A paper *(261)* exploring the possible cost savings related to reducing length of stay after birth, the consequences for postnatal services in the community, and the impact on quality of care suggests that reducing the length of time women and newborns spend in health facilities after birth implies savings in cost, as staff and bed numbers could be reduced. However, the cost savings may be reduced if quality and access to services are maintained. Simply reducing staffing in proportion with the length of stay increases the workload for each staff member that could result in poorer quality of care and increased staff stress quality. Safety of care would also require corresponding increases in community-based postnatal care.

Table 3.77 Main resource requirements for early discharge following vaginal birth

Resource	Description
**Staff**	• Same designated staff for postnatal care of women and newborns in health facilities (nurse, midwife, doctor, social worker or another health worker)<br>• Might require additional personnel for follow-up after early discharge (home visits, phone-based follow-up, outpatient postnatal care contacts)
**Training**	• Same as regular practice-based training for health workers<br>• Might require additional personnel and education or training sessions for follow-up after early discharge
**Supplies**	• Same as usual care for in-facility postnatal care<br>• Might require redistribution or additional supplies for follow-up after early discharge
**Equipment and infrastructure**	• Same basic and adequate equipment for postnatal care that is available in sufficient quantities at all times in the postnatal care ward<br>• In general, may reduce bed occupancy, unless high maternity volumes<br>• Might require additional equipment available for follow-up after early discharge
**Time**	• Same time requirements for admission and discharge from postnatal ward<br>• Less staff time on the postnatal ward, and the same or increased staff time for follow-up after early discharge (travel time for home visits, time for home visits, phone-based contacts, outpatient postnatal contacts)<br>• Increased time for women and caregivers after discharge (e.g. to travel to health workers for outpatient contacts and time the woman's support person might have to take off work following discharge)
**Supervision and monitoring**	• Regular supportive supervision and review by ward/clinic/facility lead<br>• Might require building and enhancing systems for follow-up after early discharge (integration of midwives or nurses into home-based postnatal care, establishing networks of health workers, integration of facility and community postnatal care)

Equity

No direct evidence was identified on the impact on health equity of a policy of early discharge from health facilities after uncomplicated, term vaginal birth.

An analysis of factors associated with length of stay after childbirth using data from Demographic and Health Surveys (DHS) in 30 countries suggests women of older age and those attended by doctors had longer length of stays. Women attended by non-skilled staff consistently had shorter lengths of stay than those attended by nurse-midwives, as well as wealthier women, or those who delivered by caesarean birth in the private sector *(11)*.

A policy of early discharge from a health facility after uncomplicated, term vaginal birth may decrease equity if coverage of postnatal care before and after discharge for woman–infant dyads is lower in low socioeconomic groups or in rural areas, or if women and families are expected to cover the cost of postnatal care visits after discharge or other costs (e.g. transport to outpatient care, child care support of siblings, loss of income if a partner/family member required time off work). However, early discharge policies may increase equity if accompanied by strategies (e.g. home visits) to ensure follow-up after discharge for all woman–infant dyads.

Additional considerations

Another study using DHS data from 33 sub-Saharan African countries suggests the percentage of women receiving postnatal checks before discharge from health facilities varied widely across all countries. Women who were more educated, wealthier, who received more antenatal care visits, or who had a caesarean birth were more likely to have received a pre-discharge check. In contrast, women who gave birth at lower-level public facilities (versus a public hospital) or with a nurse/midwife (versus a doctor) were less likely to have received a postnatal check *(262)*.

A study based on data from 25 sub-Saharan African countries from 2000 to 2016 *(263)* showed that the percentage of births occurring in facilities was significantly lower for the poorest women compared

with the wealthiest women, although the extent of wealth-based differences had reduced over time. Another study based on data from 43 DHS from 2003 to 2013 also found increases in health facility births in Africa and Asia, in almost all wealth groups, urban and rural areas and public and private health facilities. However, socioeconomic differences persisted, with wealthier women and those living in urban areas more likely to access facility care for childbirth *(264)*.

Acceptability

Evidence from a qualitative evidence synthesis exploring the perspectives of women, men and health workers on discharge (22) indicates that care for women is often seen to be overlooked during the postnatal care period, with predominant emphasis put on the care of the baby (moderate confidence in the evidence) and assumptions that women receive the information they need during antenatal care (low confidence in the evidence). The discharge process is often viewed as rushed by both women and health workers, with too much information, and many time limitations and health workforce shortages (low confidence in the evidence). Women and parents would appreciate more knowledge and practical skills related to taking care of themselves and their newborns, and mitigating postpartum depression (moderate confidence in the evidence). Women and partners, as well as health workers, appreciate the engagement and preparation of parents and families in relation to postnatal care (moderate confidence in the evidence). Health workers indicated that they would appreciate more tailored guidelines and training for providing postnatal education to women and families (moderate confidence in the evidence).

Evidence from a qualitative evidence synthesis exploring women's experiences of postnatal care *(28)* indicates that women need help with infant feeding, bathing and changing, as well as opportunities for recuperation and rest (high confidence in the evidence). Women, especially first-time mothers, may require time to assimilate pre-discharge information to feel confident in their ability to cope at home (high confidence in the evidence). This includes specific information about infant behaviours (feeding, sleeping and crying cues) and infant safety, development and well-being. Some women look to health workers for information to meet personal needs relating to perineal trauma, pain, wound care and emotional well-being (high confidence in the evidence). The same review also highlights the importance of the postnatal environment and suggests that, for some women, their inability to control the often noisy and disruptive atmosphere on postnatal wards can generate feelings of frustration and despair, prompting some to leave earlier than planned (moderate confidence in the evidence).

Feasibility

The qualitative evidence synthesis on the perspectives of women, men and health workers *(91)* suggests that the lack of time due to staff shortages (low confidence in the evidence), lack of staff training (moderate confidence in the evidence), unavailability of information in different languages, financial/insurance constraints affecting the length of stay, and societal norms affecting how postnatal care education is received (moderate confidence in the evidence) may limit the delivery of discharge preparation approaches.

A qualitative evidence synthesis of health workers' views and experiences of postnatal care found no direct evidence relating to views on timing of discharge after birth *(29)*. However, indirect evidence suggest that lack of personnel and heavy workload constrained the availability and quality of services, including care around the time of discharge after childbirth. Administrative duties related to discharge and paperwork added to health workers' workload and made some midwives feel that their ability to provide quality, woman-centred care was compromised as they were not able to spend enough time with each woman and to assess their needs; problems for the mother or her baby were therefore not always fully addressed. Providers perceived the need to build trustful, sensitive relationships with women, and to provide them with sufficient and timely information (low confidence in the evidence).

Table 3.78 Summary of judgements: Early discharge following vaginal birth compared with usual discharge

Domain	Judgement
Desirable effects	Small
Undesirable effects	Don't know
Certainty of the evidence	Very low
Values	Probably no important uncertainty or variability
Balance of effects	Does not favour either
Resources required	Negligible costs or savings
Certainty of the evidence on required resources	No included studies
Cost-effectiveness	Don't know
Equity	Varies
Acceptability	Varies
Feasibility	Varies

Comparison 2: Early discharge following caesarean birth compared with usual discharge

Five trials (4641 women) only included women who were planning, or who had, a caesarean birth. One trial each was conducted in Bangladesh, Denmark, Egypt, Malaysia and the USA, and they were published between 1994 and 2016. Early discharge policies varied post-caesarean, from the first to the third day afterwards. Standard practices for the time of discharge post-caesarean varied across settings, from 48 hours to seven days post-caesarean. Two trials included home visits in the intervention arm. In two trials, postnatal care after discharge was similar in the intervention and comparison groups: an appointment at the clinic two and six weeks after discharge in one trial, and strict instructions about wound care and breastfeeding in another.

Maternal outcomes

Maternal mortality: A narrative synthesis of two trials reported no maternal deaths within one year after caesarean birth among the 1545 women allocated to a policy of early discharge or the 1653 women allocated to usual discharge.

Short-term maternal morbidity: Low-certainty evidence suggests that a policy of early discharge (co-interventions not reported) after caesarean birth may reduce the number of women reporting health problems in the first six weeks postpartum when compared with usual discharge (1 trial, 200 women; RR 0.25, 95% CI 0.11 to 0.59). Low-certainty evidence suggests that a policy of early discharge (with no co-interventions) after caesarean birth may have little or no effect on the number of women with postpartum depression within six months when compared with usual discharge (2 trials, 3340 women; RR 1.08, 95% CI 0.44 to 2.64).

Health service use: Low-certainty evidence suggests that a policy of early discharge (with no co-interventions) after caesarean birth may make little or no difference to the risk of maternal readmission within six weeks when compared with usual discharge (4 trials, 3605 women; RR 1.05, 95% CI 0.74 to 1.49). Moderate-certainty evidence suggests that a policy of early discharge (with home visits) after caesarean birth probably has little or no effect on the number of women who had extra contacts with health workers due to maternal health issues within six weeks when compared with usual discharge (2 trials, 464 women; RR 0.72, 95% CI 0.43 to 1.20).

Maternal functioning/well-being and *experience of postnatal care* were not reported in the included trials for this subgroup. Cost outcomes are reported under Resources.

Newborn/infant outcomes

Neonatal/infant mortality: It is uncertain whether a policy of early discharge has any effect on the risk of infant mortality within 28 days when compared with usual discharge (very low-certainty evidence).

Health service use: It is uncertain whether a policy of early discharge after caesarean birth has any effect on infant readmission for neonatal morbidity within seven days when compared with usual discharge (very low-certainty evidence). Moderate-certainty evidence suggests that a policy of early discharge (with no co-interventions) after caesarean birth probably increases the risk of infant readmission for neonatal morbidity within 28 days when compared with usual discharge (4 trials, 3605 participants; RR 1.57, 95% CI 1.24 to 1.99).

Breastfeeding status: Low-certainty evidence suggests a policy of early discharge (with no co-interventions)

after caesarean birth may have little or no effect on the number of women breastfeeding (exclusively or partially) at six weeks postpartum when compared with usual discharge (2 trials, 3340 participants; RR 0.99, 95% CI 0.83 to 1.18).

Severe neonatal morbidity was not reported in the included trials.

Additional considerations

The Cochrane systematic review did not include separate analysis by time of early discharge for vaginal and caesarean births. Additional analyses were conducted to complement this evidence summary, assessing the effects of early discharge at less than 24 hours versus later discharge by mode of birth (see Web Supplement).[47]

Values

Evidence around the values of women is the same as for the previous comparison.

Resources

No economic evaluations of policies of early discharge from health facilities after caesarean birth were identified.

One trial *(265)* reported on the costs of hospital care for the period immediately following an unplanned caesarean birth up to the time of discharge. These costs were US$ 7648 (71 women) in the group with early discharge, home visits and phone-based follow-up (mean stay of 3.6 days) and US$ 10 971 (71 women) in the late discharge group (mean stay of 4.8 days) (cost difference between groups of US$ 3323). The same trial reported combined costs of community care (mean cost of nurse-specialist visits [in hospital and at home], home caregiver charges, acute care visits [following discharge] and rehospitalization charges), from US$ 516 (61 women) for those in the early discharge group (24 hours or less after caesarean birth) to US$ 519 (61 women) for those in the late discharge group (at least 48

Table 3.79 Main resource requirements for early discharge following caesarean birth

Resource	Description
**Staff**	• Same designated staff for postnatal care of women and newborns in health facilities (nurse, midwife, doctor, social worker or another provider)<br>• Might require additional personnel for follow-up after early discharge (home visits, phone-based follow-up)
**Training**	• Same as regular practice-based training for health workers<br>• Might require additional personnel and education or training sessions for follow-up after early discharge
**Supplies**	• Same as usual for in-facility postnatal care<br>• Might require additional supplies for follow-up after early discharge, including caesarean wound care
**Equipment and infrastructure**	• Same basic and adequate equipment for postnatal care that is available in sufficient quantities at all times in the postnatal care ward<br>• Might require additional equipment available for follow-up after early discharge
**Time**	• Same time requirements for admission and discharge from postnatal ward; varies depending on the length of stay after childbirth<br>• Might require less staff time overall before discharge and same or increased time for follow-up after early discharge (travel time for home visits, time for home visits, phone-based contacts)<br>• Increased time for women and caregivers (to travel to health workers, time that the woman's support person had to take off work following discharge)
**Supervision and monitoring**	• Regular supportive supervision and review by ward/clinic/facility lead<br>• Might require building and enhancing systems for follow-up after early discharge (integration of midwives or nurses into home-based postnatal care, establishing networks of health workers, integration of facility and community postnatal care)

47 The Web Supplement is available at: https://www.who.int/publications/i/item/9789240045989

hours after caesarean birth) (cost difference between groups of US$ 3).

Additional considerations

Additional considerations around resources are the same as for the previous comparison.

Equity

No direct evidence was identified on the impact on health equity of a policy of early discharge from health facilities after caesarean birth. Other evidence is the same as for the previous comparison.

Additional considerations

Additional considerations around equity are the same as for the previous comparison.

Acceptability

Evidence around acceptability is the same as for the previous comparison.

Feasibility

Evidence around feasibility is the same as for the previous comparison.

Table 3.80 Summary of judgements: Early discharge following caesarean birth compared with usual discharge

Domain	Judgement
Desirable effects	Small
Undesirable effects	Moderate
Certainty of the evidence	Low
Values	Probably no important uncertainty or variability
Balance of effects	Does not favour either
Resources required	Negligible costs or savings
Certainty of the evidence on required resources	No included studies
Cost-effectiveness	Don't know
Equity	Varies
Acceptability	Varies
Feasibility	Varies

C.3 Criteria to be assessed prior to discharge from the health facility after birth

RECOMMENDATION 46

Prior to discharging women and newborns after birth from the health facility to the home, health workers should assess the following criteria to improve maternal and newborn outcomes:

- **the woman's and baby's physical well-being and the woman's emotional well-being;**
- **the skills and confidence of the woman to care for herself and the skills and confidence of the parents and caregivers to care for the newborn; and**
- **the home environment and other factors that may influence the ability to provide care for the woman and the newborn in the home, and care-seeking behaviour.** *(Recommended)*

Remarks

- In making this recommendation, the Guideline Development Group considered discharge criteria for women and term newborns without complications described in policy and research documents as identified in a scoping review *(91)*.
- These criteria should be assessed to guide health workers to identify and manage problems before discharge, to provide information as per the individual woman, newborn and family needs, and to establish links to follow-up care and additional support that may be required.
- Effective counselling and communication strategies, using culturally acceptable methods that respect and facilitate shared decision-making, are integral to the assessment of discharge criteria.

Summary of evidence and considerations

Effects of the interventions

Evidence was derived from a scoping review on discharge preparation and readiness in facilities prior to discharge after birth *(91)*. The identified policy and research documents in the scoping review did not assess the effects of using criteria at discharge.

Additional considerations

The scoping review identified 13 policy documents and 17 research documents with discharge criteria. The research documents included research studies (9), review articles (3), commentaries (2), a thesis (1), a medical news article (1), and an unpublished evaluation report (1). From the 13 policy documents, 12 were postnatal specific documents and one was concerned with discharge of preterm infants. From the 17 research documents, 14 were concerned with postnatal discharge, two were specific to discharge of preterm infants and one focused on discharge of hospitalized children. Policy documents originated from Canada, India, the United Kingdom and the USA, and five had a global focus. Research documents originated from Canada, Chile, France, Ireland, Poland, Spain, Turkey, the United Kingdom, the USA and the Bolivarian Republic of Venezuela.

Three minimum discharge criteria were the most commonly referred to in the policy and research documents: (i) assessment of maternal and infant physiological stability; (ii) knowledge, ability and confidence regarding women's self-care and newborn care; and (iii) availability to care for the woman and newborn following discharge. Most documents mentioned assessment of maternal and infant physiological stability as a criterion. In policy documents, the most commonly reported components were physical examination, nutrition and weight status of the newborn. Research documents mirrored this, with components for assessing physical condition of the newborn mentioned more often than assessment of maternal health. Most documents reported assessment of knowledge, ability and confidence regarding self-care and infant care, including breastfeeding. Policy (8) and research (9) documents mentioned assessment of availability of obstetric/midwifery and infant care following discharge. Assessing timely follow-up arrangements was the most frequently reported component. Other components included identification of a health facility in case of emergency, and links to community postnatal services (e.g. follow-up instructions or plans and immunizations).

An assessment of the support available at home was much more frequently reported in research (14) than in policy documents (3). Research documents mentioned a broad range of home environment factors (e.g. domestic violence, financial concerns, and the presence of second-hand smoke). Research documents also more frequently reported on the assessment of women's emotional well-being (e.g. depression and where and when to seek support, substance abuse, and availability of support at home) and social risk factors (e.g. language barriers, local residence or access barriers to services, and age of mother).

Values

See Box 3.14 in section 3.C: Health systems and health promotion interventions.

In addition, a qualitative evidence synthesis on the perspectives of women, men and health workers related to postnatal hospital discharge *(22)* suggests that women value postnatal education for themselves and their partners and families to ensure parental confidence, and the opportunity to practice care with the support of a midwife (moderate confidence in the evidence). Both women and men value their autonomy in relation to the discharge process (moderate confidence in the evidence), including decisions regarding the timing of the discharge, adequate recognition by staff at the hospital, and availability of care without direct interference and intrusiveness.

A qualitative evidence synthesis exploring what women want from postnatal care *(21)* indicates that women value a variety of information and sources of support to help them cope with the transition to motherhood (high confidence in the evidence). To assist with this transition, women describe a range of health worker characteristics including the ability to offer safe, kind, respectful care and the sensitivity to acknowledge individual needs and cultural preferences (moderate to high confidence in the evidence).

Resources

The scoping review on discharge preparation and readiness in facilities prior to discharge after birth included any type of document describing discharge after birth, including published research of economic evaluations. No economic studies were identified.

Table 3.81 Main resource requirements for assessing discharge readiness prior to discharge from the health facility after birth

Resource	Description
**Staff**	• Nurse, midwife, doctor, social worker, or another provider
**Training**	• Varies depending on the criteria used for discharge assessment and usual care<br>• Might require additional health workers (nurse, midwife, physician) education or training and organized initiatives to enhance assessment of discharge preparedness and readiness
**Supplies**	• Varies depending on the criteria used as part of discharge assessment and usual available care<br>• Might require use of educational materials and job aids
**Equipment and infrastructure**	• Varies depending on the criteria used as part of discharge assessment and available usual care<br>• Might require building and enhancing systems for discharge assessment, such as by way of establishing networks of health workers, hospitals, insurers, social agencies and community organizations, to ensure care is streamlined and integrated
**Time**	• Varies depending on the criteria used as part of discharge assessment and usual available care<br>• Likely to require additional time for the health worker associated with implementing the criteria
**Supervision and monitoring**	• Varies depending on the criteria used as part of discharge assessment and usual available care<br>• Might require additional monitoring and assessment of the quality of discharge assessment and teaching (e.g. as a process indicator for measuring providers' skills and ability to prepare women/parents/caregivers)

Equity

No direct evidence was identified from the scoping review on the impact on health equity of assessing discharge readiness prior to discharge from the health facility after birth. Use of the criteria as part of discharge assessment may increase equity, as it may identify women, newborns and families most in need of further care and support, thereby identifying targeted interventions that lead to efficient management and use of health-care resources.

Acceptability

The qualitative evidence synthesis on the perspectives of women, men, and health workers from the scoping review found that parents would appreciate procedures during postnatal hospital discharge that would enhance their knowledge and practical skills related to taking care of themselves and their newborns and mitigate postpartum depression (moderate confidence in the evidence) *(22)*.

The findings also indicate that care for women is often seen to be overlooked during the postnatal period, with predominant emphasis put on the care of the baby (moderate confidence in the evidence) and assumptions that women receive the information they need during antenatal care (low confidence in the evidence). The discharge process is often viewed as rushed by both women and health workers, with too much information, and many time limitations and health workforce shortages (low confidence in the evidence). In some instances, women may prefer to get home quickly (low confidence in the evidence). Health workers indicated they would appreciate more tailored guidelines and training for providing postnatal education to women and families (moderate confidence in the evidence), and women and men, as well as health workers, appreciate the engagement and preparation of both parents and families in postnatal care (moderate confidence in the evidence).

Indirect evidence from a qualitative synthesis of women's experiences of postnatal care *(28)* indicates that women welcome clear and consistent discharge information, particularly if it is tailored to suit their individual needs (high confidence in the evidence). Evidence from the same review also indicates that, while women recognize the clinical priority of monitoring infant or neonatal outcomes, they also feel that postnatal assessments should incorporate maternal psychological and emotional well-being (high confidence in the evidence). Women are therefore less likely to appreciate criteria for discharge readiness that focus solely on infant-related clinical and developmental outcomes and/or clinical or physiological maternal outcomes (high confidence in the evidence).

Feasibility

The qualitative evidence synthesis on the perspectives of women, men and health workers on discharge suggests that the lack of time due to staff shortages (low confidence in the evidence), lack of staff training (moderate confidence in the evidence), unavailability of information in different languages, financial/insurance constraints affecting the length of stay, and societal norms affecting how postnatal care education is received (moderate confidence in the evidence) may limit the use of criteria for discharge assessment *(22)*.

Indirect evidence from a qualitative synthesis of women's experiences of postnatal care *(28)* suggests that in some contexts there are staff shortages, a lack of basic resources and a lack of privacy in postnatal settings, all of which may impact on providers' capacity to complete a more comprehensive discharge assessment of women in their care (low to moderate confidence in the evidence).

Indirect evidence from a qualitative evidence synthesis of health workers' views and experiences of postnatal

care *(29)* suggests that a lack of personnel and heavy workload constrained the availability and quality of services, including care around the time of discharge after childbirth. Administrative duties related to discharge and paperwork added to health workers' workload and made some midwives feel that their ability to provide quality, woman-centred care was compromised as they were not able to spend enough time with each woman and to assess their needs; thus problems for the mother or her baby were not always fully addressed. Indirect evidence also suggests that postnatal care providers felt they lack sufficient training on newborn examinations (moderate confidence in the evidence). Providers perceived the need to build trustful, sensitive relationships with women, and to provide them with sufficient and timely information (low confidence in the evidence). The lack of continuity of care and common policies or guidelines across different cadres and levels of maternal health services may limit the offer of consistent information and breastfeeding counselling (moderate confidence in the evidence).

Table 3.82 Summary of judgements: Use of criteria for discharge assessment compared with no criteria or other criteria

Domain	Judgement
Desirable effects	Don't know
Undesirable effects	Don't know
Certainty of the evidence	No included studies
Values	Probably no important uncertainty or variability
Balance of effects	Don't know
Resources required	Varies
Certainty of the evidence on required resources	No included studies
Cost-effectiveness	Don't know
Equity	Probably increased
Acceptability	Probably yes
Feasibility	Varies

C.4 Approaches to strengthen preparation for discharge from the health facility to home after birth

RECOMMENDATION 47

Information provision, educational interventions and counselling are recommended to prepare women, parents and caregivers for discharge from the health facility after birth to improve maternal and newborn health outcomes, and to facilitate the transition to the home. Educational materials, such as written/digital education booklets, pictorials for semi-literate populations and job aids should be available.
(Recommended)

Remarks

- The Guideline Development Group (GDG) agreed there was insufficient evidence to determine if any particular approach to strengthen preparation for discharge was more effective than others. Direct and indirect evidence identified approaches with the following components: counselling, education and information provision; the availability of educational resources including job aids; activities to strengthen the skills of the care providers; and ensuring linkages are made for follow-up care after discharge.
- The GDG highlighted that linkages to ensure the continuity of care after discharge should be established, including with the community health workforce, other social services or additional support as available and needed.

Summary of evidence and considerations

Effects of the interventions (EB Table C.4)

Evidence was derived from a scoping review on discharge preparation and readiness in facilities prior to discharge after birth *(91)*. The review included eight research papers on interventions to improve the delivery of discharge preparation, of which one RCT and one non-randomized evaluation used a comparison group and were considered in this evidence summary.

Comparison 1: Written education booklets for women compared with control leaflets

One RCT (387 women) conducted in Lebanon randomized postpartum women with a live birth to receive a written education booklet compared with a group receiving control leaflets on children's safety. Outcomes were assessed 6–20 weeks postpartum.

Maternal outcomes

Health service use: Moderate-certainty evidence suggests that written education booklets probably increase postpartum visits to a health professional compared with control leaflets (1 trial; 387 women; proportion visiting a health professional in the intervention group: 85%; proportion visiting a health professional in the control group: 55%; difference in the proportions: 30%; $P < 0.001$).

Experience of postnatal care: Moderate-certainty evidence suggests that written education booklets probably increase maternal satisfaction when compared with control leaflets (1 trial; 387 women; proportion satisfied in the intervention group: 57.2%; proportion satisfied in the control group: 38.9%; difference in the proportions: 18.3%, $P < 0.001$).

Maternal morbidity, maternal functioning/well-being, self-care in the home and *discharge preparedness* were not reported in the included study.

Newborn outcomes

No newborn outcomes were reported in the included study.

Health systems outcomes

No health systems outcomes were reported in the included study.

Comparison 2: Discharge education by a designated nurse compared with usual care

One non-randomized study (60 women) conducted in the USA assessed the effect of discharge education (by a designated nurse compared with routine care among women with healthy infants. The study assessed discharge preparedness in women prior to discharge.

Maternal outcomes

Discharge preparedness: It is uncertain whether discharge education by a designated nurse increases discharge preparedness in terms of personal status, knowledge, coping ability and expected support when compared with routine care (very low-certainty evidence).

Maternal morbidity, maternal functioning/well-being, self-care in the home, health service use and *experience of postnatal care* were not reported in the included study.

Newborn outcomes

No newborn outcomes were reported in the included study.

Health systems outcomes

No health systems outcomes were reported in the included study.

Additional considerations

The scoping review identified one non-randomized study (80 women) assessing the effect of discharge education through sessions starting at 32–36 weeks of pregnancy until 4–6 weeks after childbirth, compared with routine care among women with healthy infants *(266)*. The first session during pregnancy covered labour readiness and childbirth and postpartum issues; the second session was implemented before discharge and included newborn care and breastfeeding; and a third session was 4–6 weeks after birth, with education about self-efficacy and quality of life after childbirth. The reported outcome in this study was discharge preparedness and reported quality of life.

It is uncertain whether discharge education through sessions starting at 32–36 weeks of pregnancy increase discharge preparedness when compared with routine care (92.5% in intervention group versus 67.5% in control group, $P = 0.005$) (very low-certainty evidence).

A systematic review published in 2013 also evaluated the impact of educational interventions after birth on the health of the baby and the knowledge of the parents *(267)*. Educational interventions included infant sleep enhancement, infant behaviour, general post-birth health, infant care and infant safety. The review found insufficient evidence to determine the effects of any approach, and the authors concluded the benefits of educational programmes to participants and their newborns remain unclear.

Values

See Box 3.14 in section 3.C: Health systems and health promotion interventions.

In addition, a qualitative evidence synthesis on the perspectives of women, men and health workers related to postnatal hospital discharge *(22)* suggests that women value postnatal education for themselves and their partners and families to ensure parental confidence, and the opportunity to practice care with the support of a midwife (moderate confidence in the evidence). Both women and men value their autonomy in relation to the discharge process (moderate confidence in the evidence), including decisions regarding the timing of the discharge, adequate recognition by staff at the hospital, and availability of care without direct interference and intrusiveness.

In addition, a qualitative evidence synthesis exploring what women want from postnatal care *(21)* indicates that women value a variety of information and sources of support to help them cope with the transition to motherhood (high confidence in the evidence). To assist with this transition, women describe a range of health worker characteristics including the ability to offer safe, kind, respectful care, and the sensitivity to acknowledge individual needs and cultural preferences (moderate to high confidence in the evidence).

Resources

The scoping review on discharge preparation and readiness in facilities prior to discharge after birth included any type of document describing discharge after birth, including published research of economic evaluations. No economic studies were identified.

Table 3.83 Main resource requirements for strengthening preparation for discharge from the health facility to home after birth

Resource	Description
**Staff**	• Designated staff for discharge preparation (discharge nurse, staff able to provide family centred care)
**Training**	• Varies depending on the approach taken to strengthen discharge preparation and usual care<br>• Might require additional nurse, midwife and health worker education or training sessions
**Supplies**	• Varies depending on the approach taken to strengthen discharge preparation<br>• Might require written educational materials for women and handbooks for health workers, as well as discharge forms, discharge folder (e.g. to record education and follow the woman throughout), resource packs
**Equipment and infrastructure**	• Varies depending on the approach taken to strengthen discharge preparation and usual care<br>• Might require building and enhancing systems for delivery of discharge preparation interventions, such as by integrating midwives or nurses into home-based postnatal care, establishing networks of health workers, hospitals, insurers, social agencies and community organizations to ensure that care is streamlined, and individualized discharge care plans
**Time**	• Varies depending on the approach taken to strengthen discharge preparation and usual care<br>• Likely to require additional time for staff to participate in training, and resources (human and financial) for cascade training
**Supervision and monitoring**	• Varies depending on the approach taken to strengthen discharge preparation and usual care<br>• Might require additional supervision, monitoring and support for staff to implement discharge preparation

Equity

No direct evidence was identified from the scoping review on the impact on health equity of approaches for delivering discharge preparation. Approaches to deliver discharge preparation may increase equity, as these may facilitate delivery of targeted care to women, newborns and families in accordance with their needs and contexts. However, delivery of such interventions may require health-care infrastructure and trained health workers, which are limited in many low-income countries. Discharge preparedness interventions that use written materials could impact equity if efforts are not employed to address the needs of populations who do not read, or if different language needs are not considered.

Acceptability

The qualitative evidence synthesis on the perspectives of women, men, and health workers on discharge *(22)* found that mothers and fathers would appreciate procedures during postnatal hospital discharge that would enhance their knowledge and practical skills related to taking care of themselves and their newborns, and how to mitigate postpartum depression (moderate confidence in the evidence). Care for women is often overlooked during the postnatal period, with predominant emphasis on the care of the baby (moderate confidence in the evidence), and an assumption that women receive the information they need during antenatal care (low confidence in the evidence). The discharge process is often viewed as rushed by both women and health workers, with too much information and many time limitations and health workforce shortages (low confidence in the evidence). In some instances, women may prefer to get home quickly (low confidence in the evidence). Health workers indicated they would appreciate more tailored guidelines and training for providing postnatal education to women and families (moderate confidence in the evidence), and both women and men, as well as health workers, appreciate the engagement and preparation of parents and families in postnatal care (moderate confidence in the evidence).

Indirect evidence from a qualitative synthesis of women's experiences of postnatal care *(28)* indicates that women welcome clear and consistent discharge information, particularly if it is tailored to suit their individual needs (high confidence in the evidence). Evidence from the same review also indicates that women may be coming to terms with a wide variety of challenging emotions during the early postnatal period (including joy, fatigue, depression, exhaustion and trauma) so may not be receptive to large amounts of information relating to discharge practices (moderate to high confidence in the evidence). Women are therefore likely to appreciate flexible approaches to discharge preparation, incorporating multiple contacts with health workers, opportunities to discuss issues and concerns with relevant staff, and information on how to access services and staff post-discharge (moderate to high confidence in the evidence).

Feasibility

The qualitative evidence synthesis on the perspectives of women, men, and health workers *(22)* suggests that the lack of time due to staff shortages (low confidence in the evidence), lack of staff training (moderate confidence in the evidence), unavailability of information in different languages, financial/insurance constraints affecting the length of stay and societal norms affecting how postnatal care education is received (moderate confidence in the evidence) may limit the delivery of discharge preparation approaches.

Indirect evidence from a qualitative synthesis of women's experiences of postnatal care *(28)* suggests that in some contexts there are staff shortages, a lack of basic resources and a lack of privacy in postnatal settings, all of which may impact on the capacity to provide adequate discharge preparation for women (low to moderate confidence in the evidence).

Indirect evidence from a qualitative evidence synthesis of health workers' views and experiences of postnatal care *(29)* suggest that lack of personnel and heavy workload constrained the availability and quality of services, including care around the time of discharge after childbirth. The lack of continuity of care and common policies or guidelines across different cadres and levels of the maternal health services may also limit the offer of consistent information and breastfeeding counselling (moderate confidence in the evidence).

Table 3.84 Summary of judgements: Approaches for strengthening discharge preparation compared with usual care

Domain	Judgement
Desirable effects	Varies
Undesirable effects	Don't know
Certainty of the evidence	Moderate
Values	Probably no important uncertainty or variability
Balance of effects	Don't know
Resources required	Varies
Certainty of the evidence on required resources	No included studies
Cost-effectiveness	Don't know
Equity	Varies
Acceptability	Probably yes
Feasibility	Varies

C.5 Home visits for postnatal care contacts

RECOMMENDATION 48

Home visits during the first week after birth by skilled health personnel or a trained community health worker are recommended for the postnatal care of healthy women and newborns. Where home visits are not feasible or not preferred, outpatient postnatal care contacts are recommended. *(Recommended)*

Remarks

- In making this recommendation, the Guideline Development Group (GDG) considered evidence from trials where home visits for the provision of postnatal care were conducted mainly during the first week after birth.
- The content of postnatal care during home visits in the trials included assessments of the woman and newborn's physical well-being and the woman's emotional well-being with referral for further care where necessary, health education, counselling and breastfeeding promotion and support.
- The GDG noted that most trials showing a reduction in neonatal mortality were conducted in rural, low-resource settings with low access to health services and included community packages with home visits by trained community health workers, accompanied by antenatal home visits and community mobilization.
- The capacity of the health system to provide postnatal care home visits should be assessed based on local availability of skilled and trained health work force, distribution of tasks among the health workforce and the competing responsibilities with other health programmes, capacity to provide initial and continuous training and supervision, content of the postnatal care home visits, accessibility for hard to reach populations, coordination between facility- and community-based services, and sustainability of the home visits programme and of the supply systems.

Summary of evidence and considerations: Home visits for postnatal care contacts compared with usual care

Effects of the interventions (EB Table C.5a)

A systematic review *(244)* assessing effectiveness and cost-effectiveness of home visits during the early postnatal period compared with no home visits, including nine RCTs with 93 083 newborns, contributed to the neonatal mortality outcome. Trials were conducted n Bangladesh (2), Ghana (1), India (3), Pakistan (2) and the Syrian Arab Republic (1). Trained community health workers (CHWs) implemented the intervention in all studies except for two, which trained a broader group of health workers (CHWs, doctors, midwives or nurses). Most studies commenced home visits in the antenatal period. The number of postnatal visits ranged from one to eight, with more than three visits in four trials. Postnatal visit timing ranged from day 1 to day 28 after birth.

A Cochrane systematic review *(243)* assessing maternal and neonatal outcomes of different home visiting schedules during the early postnatal period, including 16 RCTs with 11 718 women, contributed to the other priority outcomes. This review excluded trials in which women were enrolled and received an intervention during the antenatal period. Two trials (969 women) were considered in this evidence summary, including one individually randomized three-arm trial (903 women), and one RCT (66 women). The three-arm trial was conducted in the Syrian Arab Republic, and included women of any parity who had vaginal or caesarean birth (1103 women). The other trial was conducted in Turkey and included only primiparous women who had a vaginal birth. Women were discharged from hospital after 24 hours in the two trials. The number and content of visits, and the cadres of health workers conducting the visits, varied. One trial compared one postnatal visit at three days after birth by a trained supporter that focused on breastfeeding education with no postnatal home visits. The three-arm trial compared one home visit (on day 1 after birth) and four home visits (on days 1, 3, 7 and 30 after birth) from registered midwives, with no home visits or planned postnatal care following hospital discharge.

Comparison: Home visits for postnatal care contacts compared with usual care

Maternal outcomes

Short-term maternal morbidity: Low-certainty evidence suggests postnatal home visits may have little or no effect on severe maternal morbidity when compared with usual care (2 trials, 876 women; RR 0.97, 95% CI 0.80 to 1.17). It is uncertain whether postnatal home visits have any effect on secondary postpartum haemorrhage or on abdominal pain up to 42 days postpartum when compared with usual care (very low-certainty evidence). Low-certainty evidence suggests postnatal home visits may have little or no effect on back pain up to 42 days postpartum when compared with usual care (2 trials, 876 women; RR 0.96, 95% CI 0.83 to 1.11). It is uncertain whether postnatal home visits have any effect on maternal fever up to 42 days postpartum, on urinary tract complications up to 42 days postpartum, or on dyspareunia when compared with usual care (very low-certainty evidence).

Experience of postnatal care: It is uncertain whether postnatal home visits have any effect on maternal satisfaction with postnatal care when compared with usual care (very low-certainty evidence).

Health service use: It is uncertain whether postnatal home visits have any effect on unscheduled visits to hospital when compared with usual care (very low-certainty evidence). Low-certainty evidence suggests postnatal home visits may have little or no effect on maternal contraceptive use when compared with usual care (2 trials, 856 women; RR 0.98, 95% CI 0.82 to 1.16).

Maternal mortality, long-term maternal morbidity, maternal functioning/well-being and *cost* were not reported in the included trials.

Newborn/infant outcomes

Neonatal/infant mortality: Moderate-certainty evidence suggests postnatal home visits probably improve neonatal mortality when compared with no home visits (9 trials, 93 083 newborns; RR 0.76, 95% CI 0.62 to 0.92).

Severe neonatal/infant morbidity: It is uncertain whether postnatal home visits have any effect on infant jaundice or on infant respiratory tract infections within 42 days of birth when compared with usual care (very low-certainty evidence). Low-certainty evidence suggests postnatal home visits may reduce infant diarrhoea within 42 days of birth when compared with usual care (2 trials, 861 infants; RR 0.85, 95% CI 0.74 to 0.98).

Health service use: Low-certainty evidence suggests postnatal home visits may have little or no effect on infant immunization when compared with usual care (2 trials, 868 infants; RR 0.99, 95% CI 0.96 to 1.01). It is uncertain whether postnatal home visits have any effect on unscheduled visits to the hospital when compared with usual care (very low-certainty evidence).

Breastfeeding status: It is uncertain whether postnatal home visits have any effect on exclusive breastfeeding up to 6 weeks of age when compared with no home visits (very low-certainty evidence). Low-certainty evidence suggests postnatal home visits may increase exclusive breastfeeding up to 6 months of age when compared with usual care (3 trials, 816 infants; RR 1.50, 95% CI 1.15 to 1.94). Low-certainty evidence suggests postnatal home visits may have little or no effect on any breastfeeding up to 6 months of age when compared with usual care (2 trials, 822 infants; RR 1.01, 95% CI 0.99 to 1.04). It is uncertain whether postnatal home visits have any effect on mean duration of any breastfeeding when compared with usual care (very low-certainty evidence).

Long-term morbidity and *growth* were not reported in the systematic review.

Additional considerations

A systematic review *(244)* assessing effectiveness and cost-effectiveness of home visits during the early postnatal period compared with no home visits, presented the following subgroup analysis.[48]

- Three postnatal home visits (4 trials; RR 0.70, 95% CI 0.53 to 0.91) versus less than three postnatal home visits (5 trials; RR 0.77, 95% CI 0.61 to 0.98; heterogeneity $P = 0.043$).
- Home visits by CHWs (7 trials; RR 0.69, 95% CI 0.55 to 0.87) versus visits by health workers (2 trials; RR 1.26, 95% CI 0.37 to 4.30; heterogeneity $P = 0.001$).
- Community mobilization efforts with home visits to promote newborn care practices (6 trials; RR 0.69, 95% CI 0.54 to 0.88) than home visits

48 Information on the trials and participants that contributed to this analysis was not available.

alone (3 trials; RR 0.97, 95% CI 0.90 to 1.05; heterogeneity P = 0.001).

- Curative (injectable antibiotics) and preventive interventions (5 trials; RR 0.82, 95% CI 0.63 to 1.05) versus only preventive interventions (4 trials; RR 0.70, 95% CI 0.48 to 1.03; heterogeneity P = 0.016).

A community-based, cluster RCT *(268)*, published after the systematic review *(244)*, showed that home-based care delivered by dedicated CHWs (Shishu Rakshak and Anganwadi workers) was effective in reducing neonatal and infant mortality rates in five districts in India, including settings with high rates of facility births.

Values

See Box 3.14 in section 3.C: Health systems and health promotion interventions.

In addition, evidence from a qualitative evidence synthesis exploring what women want from postnatal care *(21)* indicates that women may experience periods of low mood, loneliness, anxiety and fatigue during the postnatal period (moderate confidence in the evidence) and appreciate the advice, reassurance and support (practical and emotional) they receive from health workers and family members during this time (high confidence in the evidence). Some women may struggle with labour and birth-induced trauma (physical and psychological) (high confidence in the evidence) and/or experience difficulties with breastfeeding or find it difficult to embrace their maternal identity (moderate confidence in the evidence) so are likely to value home visits by health workers to resolve these concerns. In addition, women tend to prioritize the needs of their baby during the postnatal period (moderate confidence in the evidence) so are likely to value clinical and developmental outcomes associated with their infant.

Resources

A systematic review *(244)* assessing the effectiveness and cost-effectiveness of home visits during the early postnatal period compared with no home visits identified two economic evaluations *(269, 270)* (high quality), conducted alongside clinical trials in Ghana and Bangladesh, reporting on the incremental cost for neonatal mortality outcomes. Home-based neonatal care strategies were found to be cost-effective as reported in terms of cost per neonatal death averted, newborn life-year saved or DALY averted, after costs were inflated to 2016 prices and using the GDP per capita as a benchmark.

Additional considerations

A multicountry economic analysis of community-based maternal and newborn care evaluations included five cluster-RCTs (from Ethiopia, Ghana, South Africa, Uganda and the United Republic of Tanzania) and programmatic before/after assessments (from Malawi and the Plurinational State of Bolivia) using the Cost of Integrated Newborn Care tool *(271)*. In five of the six countries, the programme would be highly cost-effective (cost per DALY averted < GDP/capita) by WHO thresholds, even if only achieving a reduction of one neonatal death per 1000 live births. The study found the main driver of costs was the number of CHWs, accounting for over 96% of costs in five of the countries. The set-up and running costs standardized per 100 000 population was less than US$ 1 per capita per year for six of the seven countries.

Table 3.85 Main resource requirements for home visits for postnatal care contacts compared with usual care

Resource	Description
**Staff**	• Designated, trained staff for postnatal home visits
**Training**	• Regular practice-based training for health workers<br>• Additional personnel and education or training sessions for staff conducting home visits
**Supplies** *(272)*	• Equivalent annual costs of home visit kits per CHW ranged from US$ 15 to US$ 116 (four visits during antenatal and postnatal period/100 000 population)<br>• Medical supplies (e.g. painkillers, iron tablets, contraceptives, anthelminthics, mosquito nets, gloves)<br>• Community/household registers to record findings of the home visits and referral slips, counselling cards or flip chart<br>• Staff supplies (e.g. boots, umbrella and bag, mobile phone)<br>• Home-based records<br>• Information/counselling cards for women/parents/caregivers, with home visitor contact information
**Equipment and infrastructure** *(272)*	• Functional birth notification system for staff performing the home visits<br>• Portable equipment to conduct home visits (e.g. weighing scales, thermometer, clock/timer, bag and mask)<br>• Access to transport to conduct postnatal home visits (e.g. bicycles, motor vehicles)
**Time**	• Total time spent on home visit programme activities (home visits, administrative duties, preparation of visits) was 3–13 hours per week *(271)*<br>– Time per home visit was a median of 23–45 minutes<br>– Additional time needed for home visits (e.g. to carry out a general assessment of the home environment and mother–infant interaction)<br>– Transport time to client's home for postnatal home visits of 20–45 minutes
**Supervision and monitoring**	• Trained supervisors, regular coordination meetings between health facilities/districts and staff conducting the home visits<br>• Systems to report stock-outs of supplies for postnatal home visits

Equity

No direct evidence was identified on the impact on health equity of postnatal home visits compared with usual care. Postnatal home visits may increase equity if coverage is achieved among woman–infant dyads from low socioeconomic groups and rural areas who are less likely to receive postnatal care, or after home births. Postnatal home visits may further increase equity if they reduce costs for women and families, including cost of transport and childcare of siblings, thus supporting and enabling attendance at outpatient postnatal care. However, it may decrease equity if coverage of home visits is lower in low socioeconomic groups or rural areas, or if women and families are expected to cover the cost of postnatal care visits.

Additional considerations

Infants face the highest risk of dying in their first month after birth, at an average global ratio of 18 deaths per 1000 live births in 2017 *(9, 273)*. Sub-Saharan Africa and South Asia present the highest neonatal mortality ratios (up to 27 deaths per 1000 live births in 2017) *(273)*, with large disparities between the poorest and richest households *(274)*.

Acceptability

Evidence from a qualitative evidence synthesis exploring women's experiences of postnatal care *(28)* indicates that women appreciate and value the practical, psychosocial and emotional support they receive from health workers during the postnatal period (high confidence in the evidence). Evidence also suggests that, once women are at home, they appreciate a variety of contact opportunities with postnatal services to smooth their transition into motherhood (moderate confidence in the evidence). The provision of home visits is highlighted by women in a number of different contexts as being of particular benefit since they are convenient and more relaxing for women and may give health workers important insights into the family's domestic

circumstances (moderate confidence in the evidence). The availability of drop-in clinics, out-of-hours services, and telephone or online services is also appreciated by women (and families) who require ongoing reassurance and support from health workers at home (moderate confidence in the evidence). In situations where home visits are already in place, women appreciate being seen by the same health professional to establish trust and familiarity with personal circumstances (moderate confidence in the evidence).

Feasibility

A qualitative evidence synthesis exploring women's experiences of postnatal care *(28)* found no direct evidence relating to the feasibility of postnatal home visits. Indirect evidence suggests the resources required (additional staff, travelling costs) to provide home visits to the community may be prohibitive in some LMIC settings, particularly in rural areas (low confidence in the evidence).

A qualitative evidence synthesis of health workers' views and experiences of postnatal care *(29)* suggests conducting home visits may sometimes be challenging for health workers, given cultural norms, difficulties accessing the homes (poor road conditions, long distances, inconvenient means of transport). Lack of personnel and a heavy workload constrained their availability to conduct postnatal care visits (low confidence in the evidence). Health workers would sometimes prioritize some services over home visits. Public health nurses perceived home visits to be less valuable and not properly understood by their professional colleagues and managers, and expressed concern that if such programmes were eliminated some women might not have access to alternative postnatal care (low confidence in the evidence). Health workers suggested reasons women may not attend clinics include lack of transport, lack of money, misconceptions that health workers are hoarding supplies and making unwarranted financial gains, cultural beliefs and practices, lack of knowledge of the importance of some services and language barriers (moderate confidence in the evidence).

Additional considerations

A multicountry, mixed-method programme review *(275)* in 12 low- and lower-middle income countries found that countries implemented postnatal care home visits mainly as part of their broader community mobilization of maternal and child health programmes. Countries used a variety of visiting schedules, which focused on the first two weeks after birth and were conducted by skilled personnel or trained CHWs. Coverage of postnatal care visits less than 48 hours after birth ranged from 44% to 93% for women and from 15% to 91% for newborns after facility births. Coverage was lower for both women and newborns after home births. Countries have responded in various ways to low-performing postnatal care home visit programmes, suspending programmes, reducing schedules for visits in the first two weeks after birth, or making no changes to their programming.

Table 3.86 Summary of judgements: Home visits for postnatal care contacts compared with usual care

Domain	Judgement
Desirable effects	Moderate
Undesirable effects	Don't know
Certainty of the evidence	Moderate
Values	Probably no important uncertainty or variability
Balance of effects	Probably favours postnatal home visits
Resources required	Moderate costs
Certainty of the evidence on required resources	Low
Cost-effectiveness	Probably favours postnatal home visits
Equity	Probably increased
Acceptability	Probably yes
Feasibility	Varies

Summary of evidence and considerations: Home visits for postnatal care contacts compared with routine outpatient postnatal care

Effects of the interventions (EB Table C.5b)

Evidence was derived from a Cochrane systematic review assessing outcomes (maternal and newborn mortality) of different home-visiting schedules during the early postpartum period *(243)*, including 16 trials with 12 080 women. Eight trials with 5029 women comparing postnatal home visits versus outpatient postnatal care were included in this evidence summary. Trials were conducted in Canada (1), the Islamic Republic of Iran (2), Spain (2) and the USA (3).

All trials compared home visits by nurses (4747 women) and trained midwives (682 women) with postnatal care in clinics or a referral to a health service centre for routine postnatal checks (usual care). The timing, number and content of home visits varied considerably across these trials, as well as the control conditions. In the intervention arms, all trials had a fixed visiting schedule, ranging from one to three postpartum home visits. Timing of the first visit varied from three to four days after birth to within the first week after birth.

Comparison: Home visits for postnatal care contacts compared with routine outpatient postnatal care

Maternal outcomes

Short-term maternal morbidity: Low-certainty evidence suggests postnatal home visits may have little or no effect on postpartum depression up to 42 days after childbirth when compared with outpatient postnatal care (2 trials, 2177 women; RR 1.10, 95% CI 0.93 to 1.30). It is uncertain whether postnatal home visits have any effect on postpartum depression at 60 days when compared with outpatient postnatal care (very low-certainty evidence). Low-certainty evidence suggests postnatal home visits may have little or no effect on the mean maternal anxiety score (last assessment up to 42 days postpartum) when compared with outpatient postnatal care (1 trial, 513 women; MD 0.3 higher, 95% CI 1.08 lower to 1.68 higher). It is uncertain whether postnatal home visits have any effect on postpartum depression and anxiety (Hospital Anxiety and Depression Scale score) when compared with outpatient postnatal care (very low-certainty evidence).

Experience of postnatal care: Low-certainty evidence suggests postnatal home visits may improve maternal satisfaction with postnatal care when compared with outpatient postnatal care (2 trials, 2368 women; RR 1.36, 95% CI 1.14 to 1.62). Low-certainty evidence suggests postnatal home visits may have little or no effect on the mean satisfaction score for postnatal care when compared with outpatient postnatal care (1 trial, 513 women; MD 0.1 lower, 95% CI 0.88 lower to 0.68 higher).

Health service use: Low-certainty evidence suggests postnatal home visits may have little or no effect on emergency health-care visits when compared with outpatient postnatal care (3 trials, 3242 women; RR 1.04, 95% CI 0.82 to 1.33). It is uncertain whether postnatal home visits may have any effect on hospital readmissions up to two weeks when compared with outpatient postnatal care (very low-certainty evidence).

Maternal mortality, long-term maternal morbidity and *cost* were not reported in the systematic review. *Maternal functioning/well-being* was not reported in the included trials.

Newborn/infant outcomes

Breastfeeding status: Low-certainty evidence suggests postnatal home visits may have little or no effect on exclusive breastfeeding up to six weeks when compared with outpatient postnatal care (1 trial, 513 newborns; RR 1.05, 95% CI 0.93 to 1.18). Moderate-certainty evidence suggests postnatal home visits probably have little or no effect on any breastfeeding up to six months when compared with outpatient postnatal care (1 trial, 1000 infants; RR 1.09, 95% CI 1.00 to 1.18). It is uncertain whether postnatal home visits have any effect on breastfeeding discontinuation after 30 days when compared with outpatient postnatal care (very low-certainty evidence). Moderate-certainty evidence suggests postnatal home visits probably have little or no effect on breastfeeding discontinuation in the first six weeks when compared with outpatient postnatal care (2 trials, 2177 newborns; RR 0.93, 95% CI 0.78 to 1.12).

Health service use: Low-certainty evidence suggests postnatal home visits may have little or no effect on infant health-care utilization when compared with outpatient postnatal care (3 trials, 3257 infants; RR 1.15, 95% CI 0.95 to 1.38). Low-certainty evidence suggests postnatal home visits may have little or

no effect on infant hospital readmissions when compared with outpatient postnatal care (3 trials, 2690 infants; RR 1.20, 95% CI 0.71 to 2.02).

Neonatal/infant mortality and *severe neonatal morbidity* were not reported in the included trials.

Values

See Box 3.14 in section 3.C: Health systems and health promotion interventions.

In addition, evidence from a qualitative evidence synthesis exploring what women want from postnatal care *(21)* indicates that women may experience periods of low mood, loneliness, anxiety and fatigue during the postnatal period (moderate confidence in the evidence) and appreciate the advice, reassurance and support (practical and emotional) they receive from health workers and family members during this time (high confidence in the evidence). Some women may struggle with labour and birth-induced trauma (physical and psychological) (high confidence in the evidence) and/or experience difficulties with breastfeeding or find it difficult to embrace their maternal identity (moderate confidence in the evidence) so are likely to value home visits by health workers to resolve these concerns. In addition, women tend to prioritize the needs of their baby during the postnatal period (moderate confidence in the evidence) so are likely to value clinical and developmental outcomes associated with their infant.

Resources

No economic evaluations of postnatal home visits compared with routine outpatient postnatal care were identified.

Additional considerations

Two trials conducted in the USA reported increased cost of 60–90 minute postnatal home visits compared with hospital-based postnatal care within 48 hours of discharge for low-risk woman-infant dyads. One study *(276)* estimated the cost of a postnatal home visit by a nurse to be US$ 265, compared with US$ 22 per woman–infant dyad for a 1–2 hour hospital-based group visit led by a registered nurse. Women in the control group could also opt for an individual 15-minute visit with a registered nurse (cost: US$ 52) or individual 15-minute paediatrician visit (cost: US$ 92). Additional costs of a 10-minute visit to the obstetrics and gynaecology clinic was estimated to be US$ 92. Another study *(277)* estimated the cost of a postnatal home visit by a nurse to be US$ 255, compared with US$ 120 for a 20-minute paediatric clinic visit. The additional cost of a 10-minute visit to the obstetrics and gynaecology clinic was estimated to be US$ 82.

Table 3.87 Main resource requirements for home visits for postnatal care contacts compared with routine outpatient postnatal care

Resource	Description
**Staff**	• Designated, trained, motivated staff for postnatal care of women and newborns in facilities/clinics/office or at home<br>• Additional personnel might be required to conduct home visits
**Training**	• Same as regular practice-based training for health workers<br>• Might require additional personnel and education or training sessions for staff conducting home visits
**Supplies**	• Same medical supplies (e.g. painkillers, iron tablets, contraceptives, anthelminthics, gloves, mosquito nets)<br>• For postnatal home visits, community/household registers to record findings of the home visits and referral slips, counselling cards or flip chart<br>• Staff supplies (e.g. boots, umbrella and bag, mobile phone)<br>• Home-based records<br>• Information/counselling cards for women/parents/caregivers, with home visitor contact information
**Equipment and infrastructure**	• Functional birth notification for staff performing the home visits or scheduling outpatient contacts<br>• For outpatient contacts, a clean, comfortable waiting room for women and their companions, and a clean, private examination room<br>• For postnatal home visits, portable equipment to conduct home visits (e.g. weighing scales, thermometer, clock/timer, bag and mask)<br>• Access to transport to conduct postnatal home visits (e.g. bicycles, motor vehicles)
**Time**	• Same time as for postnatal care in facilities/clinics/office or at home for both women and newborns, or additional time for postnatal home visits (e.g. to carry out a general assessment of the home environment and mother–infant interaction)<br>• For outpatient contacts, 10–20 minutes per contact<br>• Transport time to facility/office for outpatient contact<br>• For postnatal home visits, transport time to client's home
**Supervision and monitoring**	• Trained supervisors, and regular coordination meetings between health facilities/districts and staff conducting the home visits<br>• Systems to report stock-outs of supplies for postnatal home visits

Equity

No direct evidence was identified on the impact on health equity of postnatal home visits compared with routine outpatient care. Postnatal home visits may increase equity if coverage is achieved among women from low socioeconomic groups and rural areas, who are less likely to receive postnatal care in health facilities. Postnatal home visits may further increase equity if they reduce costs for women and family, including cost of transport and childcare of siblings, thus supporting and enabling attendance at outpatient postnatal care. However, it may decrease equity if coverage of home visits is lower in low socioeconomic groups or rural areas, or if women and families are expected to cover the cost of postnatal care visits.

Additional considerations

Postnatal home visits may increase equity if also offered after home births, in particular if offered after home births in settings with low facility-birth coverage and where home births are higher among women living in poverty and/or in rural areas.

Acceptability

Evidence from a qualitative evidence synthesis exploring women's experiences of postnatal care *(28)* indicates that women appreciate and value the practical, psychosocial and emotional support they receive from health workers during the postnatal period (high confidence in the evidence). Evidence also suggests that, once women are at home, they appreciate a variety of contact opportunities with postnatal services to smooth their transition into motherhood (moderate confidence in the evidence). The provision of home visits is highlighted by women in a number of different contexts as being of particular benefit since they are convenient and more relaxing for women and may give health workers important insights into the family's domestic

circumstances (moderate confidence in the evidence). The availability of drop-in clinics, out-of-hours services, and telephone or online services is also appreciated by women (and families) who require ongoing reassurance and support from health workers at home (moderate confidence in the evidence). In situations where home visits are already in place, women appreciate being seen by the same care provider to establish trust and familiarity with personal circumstances (moderate confidence in the evidence).

Feasibility

A qualitative evidence synthesis exploring women's experiences of postnatal care *(28)* found no direct evidence relating to the feasibility of postnatal home visits. Indirect evidence suggests the resources required (additional staff, travelling costs) to provide home visits to the community may be prohibitive in some LMIC settings, particularly in rural areas (low confidence in the evidence).

A qualitative evidence synthesis of health workers' views and experiences of postnatal care *(29)* suggests conducting home visits may sometimes be challenging for health workers, given cultural norms, difficulties accessing the homes (poor road conditions, long distances, inconvenient means of transport). Lack of personnel and heavy workload constrained their availability to conduct postnatal care visits (low confidence in the evidence). Health workers would sometimes prioritize some services over home visits. Public health nurses perceived home visitation to be less valued and not properly understood by their professional colleagues and managers, and expressed concern that if such programmes were eliminated some women might not have access to alternative postnatal care (low confidence in the evidence). Health workers suggested the reasons women may not attend clinics include lack of transport, lack of money, misconceptions that health workers are hoarding supplies and making unwarranted financial gains, cultural beliefs and practices, lack of knowledge of the importance of some services, and language barriers (moderate confidence in the evidence).

Additional considerations

A multicountry, mixed-method programme review *(275)* in 12 low- and lower-middle income countries found that countries implemented postnatal care home visits mainly as part of their broader community mobilization of maternal and child health programmes. Countries used a variety of visiting schedules, which focused on the first two weeks after birth and were conducted by skilled personnel or trained CHWs. Coverage of postnatal care visits less than 48 hours after birth ranged from 44% to 93% for women and from 15% to 91% for newborns after facility births. Coverage was lower for both women and newborns after home births. Countries have responded in various ways to low-performing postnatal care home visit programmes, suspending programmes, reducing schedules for visits in the first two weeks after birth, or making no changes to their programming.

Table 3.88 Summary of judgements: Home visits for postnatal care contacts compared with routine outpatient postnatal care

Domain	Judgement
Desirable effects	Trivial
Undesirable effects	Trivial
Certainty of the evidence	Low
Values	Probably no important uncertainty or variability
Balance of effects	Does not favour either
Resources required	Moderate costs
Certainty of the evidence on required resources	No included studies
Cost-effectiveness	Don't know
Equity	Probably increased
Acceptability	Probably yes
Feasibility	Varies

C.6 Midwifery continuity of care

RECOMMENDATION 49

Midwife-led continuity-of-care (MLCC) models, in which a known midwife or small group of known midwives supports a woman throughout the antenatal, intrapartum and postnatal continuum, are recommended for women in settings with well-functioning midwifery programmes. *(Context-specific recommendation)*

Remarks

- This recommendation has been integrated from the 2016 *WHO recommendations on antenatal care for a positive pregnancy experience (16)*, where it was considered a context-specific recommendation.
- The following remarks were made by the Guideline Development Group responsible for the original recommendation.
  - MLCC models are models of care in which a known and trusted midwife (caseload midwifery), or small group of known midwives (team midwifery), supports a woman throughout the antenatal, intrapartum and postnatal periods, to facilitate a healthy pregnancy, childbirth and postnatal period and healthy self-care and parenting practices.
  - MLCC models are complex interventions, and it is unclear whether the pathway of influence producing these positive effects is the continuity-of-care, the midwifery philosophy of care, or both. The midwifery philosophy inherent in MLCC models may or may not be enacted in standard midwifery practice in other models of care.
  - Policy-makers in settings without well-functioning midwifery programmes should consider implementing this model only after successfully scaling up the number and quality of practising midwives. In addition, stakeholders may wish to consider ways of providing continuous care through other care providers, because women value continuity-of-care.
  - The panel noted that, with this model of care, it is important to monitor resource use and health worker burnout and workload, to determine whether caseload or team care models are more sustainable in individual settings.
  - MLCC requires that well-trained midwives are available in sufficient numbers for each woman to see only one or a small group of midwives throughout pregnancy and during childbirth. This model may therefore require a shift in resources to ensure that the health system has access to a sufficient number of midwives with reasonable caseloads.
  - The introduction of MLCC may lead to a shift in the roles and responsibilities of midwives as well as other health workers who have previously been responsible for antenatal and postnatal care. Where this is the case, implementation is likely to be more effective if all relevant stakeholders are consulted and human resources departments are involved. In some settings, government-level consultation with professional organizations could also aid implementation processes.
  - The need for additional one-off or continuing training and education should be assessed, and should be provided where necessary.

C.7 Task sharing components of postnatal care delivery

RECOMMENDATION 50a

Task sharing the promotion of health-related behaviours for maternal and newborn health[a] to a broad range of cadres, including lay health workers, auxiliary nurses, nurses, midwives and doctors, is recommended. *(Recommended)*

RECOMMENDATION 50b

Task sharing the provision of recommended postpartum contraception methods[b] to a broad range of cadres, including auxiliary nurses, nurses, midwives and doctors, is recommended. *(Recommended)*

Remarks

- These recommendations have been adapted and integrated from the 2012 WHO publication *Optimizing health worker roles to improve access to key maternal and newborn health interventions through task shifting (278)*.
- The postnatal care Guideline Development Group (GDG) agreed that lay health workers who are trained and supervised can independently conduct safe and effective catch-up postpartum HIV testing, as per Recommendations 2a and 2b in this guideline, integrated from the 2019 WHO *Consolidated guidelines on HIV testing services (41)*.
- The postnatal care GDG noted that universal access to and use of long-lasting insecticidal nets remains the goal for all people, including postnatal women and newborns in malaria-endemic settings *(279)*.

a This includes promotion of the following: postnatal care, family planning (distribution of condoms [male and female] and other barrier methods, initiation and distribution of combined oral contraceptives, progestin only oral contraceptives, emergency contraception, and information and general instructions on Standard Days Method, TwoDay Method® and lactational amenorrhoea method), postpartum HIV catch-up testing and retesting, sleeping under insecticide-treated nets, nutritional advice; nutritional supplements, basic newborn care, exclusive breastfeeding and immunization according to national guidelines.

b This includes: initiating and maintaining injectable contraceptives using a standard syringe with needle for intramuscular or subcutaneous injection, insertion of intrauterine devices (IUDs) and insertion of contraceptive implants.

C.8 Recruitment and retention of staff in rural and remote areas

RECOMMENDATION 51

Policy-makers should consider a bundle of interventions covering education, regulation, incentives and personal and professional support to improve health workforce development, attraction, recruitment and retention in rural and remote areas. *(Recommended)*

Remarks

- This recommendation has been adapted and integrated from the updated 2021 *WHO guideline on health workforce development, attraction, recruitment and retention in rural and remote areas (280)*.
- Recommendations from the above source guideline (abridged) addressing education, regulation, incentives and support include the following.
  - *Education:* Use targeted admission policies to enrol students who live or have spent some childhood years in rural areas in health worker education programmes, and locate teaching and learning institutions closer to rural areas; expose students of a wide array of health worker disciplines to rural and remote communities and rural clinical practices; include rural health topics in health worker pre-service and in-service training of health workers; and design and enable access to continuing education and professional development programmes that meet the needs of rural health workers to support their retention.
  - *Regulation:* Introduce and regulate enhanced scope of practice for health workers in rural and remote areas; introduce different types of health workers to rural practices to meet the needs of communities, based on people-centred service delivery models; respect the rights of health workers when compulsory service in rural and remote areas exists, with fair, transparent and equitable management, support and incentives; and provide scholarships, bursaries or other education subsidies to health workers in return for service in rural and remote areas.
  - *Incentives:* Employ a package of fiscally sustainable financial and nonfinancial incentives to influence health workers' decisions to relocate to and remain in rural and remote areas.
  - *Support:* Invest in rural infrastructure and services to ensure decent living conditions for health workers and their families; ensure a safe and secure working environment for health workers; provide decent work that respects the fundamental rights of health workers; foster the creation of health workforce support networks for health workers in rural and remote areas; develop and strengthen career development and advancement programmes, and career pathways for health workers in rural and remote areas; support the development of networks, associations and journals for health workers in rural and remote areas to facilitate knowledge exchange; and adopt social recognition measures at all levels for health workers in rural and remote areas to raise the profile of rural health workers.

C.9 Involvement of men in postnatal care and maternal and newborn health

RECOMMENDATION 52

Interventions to promote the involvement of men during pregnancy, childbirth and after birth are recommended to facilitate and support improved self-care of women, home care practices for women and newborns, and use of skilled care for women and newborns during pregnancy, childbirth and the postnatal period, and to increase the timely use of facility care for obstetric and newborn complications.

These interventions are recommended, provided they are implemented in a way that respects, promotes and facilitates women's choices and their autonomy in decision-making, and that supports women in taking care of themselves and their newborns. *(Recommended with targeted monitoring and evaluation)*

Remarks

- This recommendation has been retained, following review of new evidence, from the 2015 *WHO recommendations on health promotion interventions for maternal and newborn health (250)*.
- The Guideline Development Group (GDG) agreed that, despite the availability of additional studies specific to the postnatal period, the evidence base continues to be heterogeneous and of mixed certainty, and therefore the GDG decided not to modify the existing 2015 recommendation.
- A diverse set of interventions was identified in the effectiveness review and the qualitative evidence synthesis, but there was insufficient evidence to identify whether any of the different implementation approaches were more effective for improving maternal and newborn health outcomes.
- The GDG indicated that both the benefits and the harms that can result from interventions are important, but that the harms can be mitigated through a well-designed and closely monitored intervention, which involves women in the design and monitoring of interventions to involve men, and asks women about their experiences of men's involvement.
- The GDG refers to the important implementation considerations highlighted in the previous WHO guideline, particularly the call for these interventions to be implemented in a way that respects, promotes and facilitates women's choices and autonomy in decision-making, and supports women in taking care of themselves and their newborns.
- The GDG recognized that the involvement of fathers is an important component of early childhood health and development (see Recommendations 38 and 39 in this guideline).

Summary of evidence and considerations

Effects of the interventions (EB Table C.9)

Evidence was derived from a systematic review *(281)* that identified 26 studies with postnatal care outcomes, including two cluster-RCTs, 13 RCTs, two non-randomized trials, one analytical cohort, and eight quasi-experimental studies. Studies were conducted in 16 countries across all six WHO regions, including LMIC and HICs. As data were not meta-analysed in the review, effects are described based on the direction of the effect estimates (positive, negative, null).

The different interventions were categorized into the following categories:

- couples education – interventions that included educational activities with couples, conducted in the home or in a facility, with either an individual couple or in groups;
- men's education – educational activities directed towards men, conducted in groups or individually, in the health facility or the community, or through text-messaging;
- multicomponent interventions that included either men only or couples education activities as well as community-mobilization, mass media efforts, home visits, etc.;
- having a companion during labour and birth, including having the father cut the umbilical cord after birth.

Comparison 1: Couples education compared with no intervention or usual care

Maternal outcomes

Health service use: Low-certainty evidence suggests couples education may have a positive effect on women having at least one postnatal visit within two weeks of childbirth compared with no intervention or usual care (1 trial, 261 women; RR 1.29, 95% CI 1.04 to 1.60). High-certainty evidence suggests couples education has a positive effect on women having two or more postnatal visits within six weeks compared with no intervention or usual care (1 trial, 1101 women; RR 1.23, 95% CI 1.11 to 1.37).

Self-care: High certainty evidence suggests couples education has a positive effect on the timely initiation of a modern contraceptive method (1 trial, 610 women; RR 1.11, 95% CI 1.00 to 1.24), and on the use of any contraceptive method at three months after childbirth (1 trial, 1085 women; RR 1.16, 95% CI 1.04 to 1.30), compared with no intervention or usual care. Moderate-certainty evidence suggests couples education probably makes little or no difference to the use of a modern contraceptive method at six months after childbirth compared with no intervention or usual care (1 trial, 921 women; RR 1.01, 95% CI 0.90 to 1.12).

Maternal morbidity and *maternal functioning/well-being* were not reported in the included studies.

Newborn/infant outcomes

Breastfeeding status: Low-certainty evidence suggests couples education may make little or no difference to the initiation of breastfeeding within 1 hour of birth compared with no intervention or usual care (1 trial, 1222 newborns; RR 1.06, 95% CI 0.82 to 1.36). Moderate-certainty evidence from four contributing studies suggests that couples education probably has a positive effect on (two studies) or makes little or no difference to (two studies) exclusive breastfeeding up to 3 months of age compared with no intervention or usual care (data not meta-analysed due to heterogeneity in the interventions).

Any breastfeeding at 6 months of age

- Evidence from RCTs: Moderate-certainty evidence suggests couples education probably makes little or no difference to any breastfeeding at 6 months of age compared with no intervention or usual care (1 trial, 1298; newborns; RR 1.01, 95% CI 0.87 to 1.19).
- Evidence from non-RCTs: It is uncertain whether couples education has any effect on any breastfeeding at 6 months of age compared with no intervention or usual care (very low-certainty evidence).

It is uncertain whether couples education has any effect on breastfeeding initiation before discharge (quasi-RCTs); exclusive breastfeeding up to 4–6 weeks of age (RCTs); exclusive breastfeeding at 1 month of age (quasi-RCTs); exclusive breastfeeding at 2 months of age (RCTs); exclusive breastfeeding at 4 months of age (RCTs and quasi-RCTs); exclusive breastfeeding at 6 months of age (RCTs); or exclusive breastfeeding discontinuation in the first six months after childbirth (non-RCTs), compared with no intervention or usual care (all very low-certainty evidence).

Neonatal morbidity, family care practices and *health service use* were not reported in the included studies.

Intra-household relationship dynamics

Couple communication and household decision-making: Low-certainty evidence suggests couples education may make little or no difference to a mother's reporting of co-parenting at 6 weeks (1 trial, 189 women; SMD 0.17 higher, 95% CI 0.12 lower to 0.45 higher) and at 12 weeks (1 trial, 189 women; SMD 0.18 higher, 95% CI 0.10 lower to 0.46 higher), compared with no intervention or usual care.

Father-child interaction and attachment: Low-certainty evidence suggests couples education may have a positive effect on the quality of the father–child interaction at six months compared with no intervention or usual care (1 trial, 165 fathers; SMD 0.46 higher, 95% CI 0.15 higher to 0.77 higher). It is uncertain whether couples education has any effect on fathers' involvement at four or eight weeks after childbirth; paternal responsibility at six months after childbirth; paternal engaged interaction or paternal parallel interaction at six months after childbirth; or total accessibility at six months after childbirth, compared with no intervention or usual care (all RCTs; very low-certainty evidence).

Gender and power dynamics within couples and *adverse effects* were not reported in the included studies.

Comparison 2: Couples education compared with women's education alone

Maternal outcomes

Health service use: Low-certainty evidence suggests couples education may have a positive effect on women having at least one postnatal care visit within two weeks of childbirth, compared with women's education alone (1 trial, 258 women; RR 1.25, 95% CI 1.01 to 1.54).

Maternal morbidity, maternal functioning/well-being and *self-care* were not reported in the included studies.

Newborn/infant outcomes

Breastfeeding status: It is uncertain whether couples education has any effect on exclusive breastfeeding at four or at six months after childbirth compared with women's education alone (non-RCTs; very low-certainty evidence).

Neonatal morbidity, family care practices and *health service use* were not reported in the included studies.

Intra-household relationship dynamics

No outcomes relating to intra-household relationship dynamics were reported in the included studies.

Comparison 3: Men's education compared with no intervention or usual care

Maternal outcomes

Health service use: It is uncertain whether men's education has any effect on women having at least one postnatal care visit within seven days of childbirth compared with no intervention or usual care (quasi-RCT; very low-certainty evidence).

Maternal morbidity: It is uncertain whether men's education has any effect on general maternal psychosocial problems at three weeks after the intervention compared with no intervention or usual care (RCT; very low-certainty evidence). Low-certainty evidence suggests men's education may have a positive effect on general psychosocial problems at six weeks after the intervention compared with no intervention or usual care (1 trial, 60 women; SMD 0.96 lower, 95% CI 1.50 lower to 0.43 lower).

Maternal functioning/well-being and *self-care* were not reported in the included studies.

Newborn/infant outcomes

Health service use: It is uncertain whether men's education has any effect on delayed bathing by at least two days compared with no intervention or usual care (quasi-RCT; very low-certainty evidence).

Breastfeeding status: Low-certainty evidence suggests men's education may make little or no difference to exclusive breastfeeding at six weeks after childbirth compared with no intervention or usual care (1 trial, 551 newborns; adjusted OR 1.09, 95% CI 0.79 to 1.51). Low-certainty evidence suggests men's education may have a positive effect on breastfeeding until six months after childbirth compared with no intervention or usual care (1 trial, 100 newborns; RR 1.24, 95% CI 1.04 to 1.47).

Neonatal morbidity and *family care practices* were not reported in the included studies.

Intra-household relationship dynamics

Father-child interaction and attachment: It is uncertain whether men's education has any effect on paternal involvement at four or at eight weeks after childbirth; on paternal responsiveness at six months after the intervention; or on paternal bonding difficulties at six months after the intervention, compared with no intervention or usual care (RCTs; very low-certainty evidence).

Couple communication and household decision-making, gender and power dynamics within couples and *adverse effects* were not reported in the included studies.

Comparison 4: Father as a labour companion compared with no companion

Maternal outcomes

Maternal morbidity: It is uncertain whether the father as a labour companion has any effect on depressive symptoms 6–8 weeks after childbirth or on anxiety 6–8 weeks after childbirth compared with no companion (non-RCT; very low-certainty evidence).

Maternal functioning/well-being, self-care and *health service use* were not reported in the included study.

Newborn/infant outcomes

No newborn/infant outcomes were reported in the included study.

Intra-household relationship dynamics
Father–child interaction and attachment: It is uncertain whether having the father as a labour companion has any effect on father–infant bonding on the first day after childbirth or in the first month after childbirth compared with no companion (quasi-RCT; very low-certainty evidence).

Couple communication and household decision-making, gender and power dynamics within couples and *adverse effects* were not reported in the included study.

Comparison 5: Father as a labour companion compared with a female friend as a labour companion

One study reported on having the father as a labour companion compared with a friend.

Maternal outcomes
Maternal morbidity: It is uncertain whether having the father as a labour companion has any effect on depressive symptoms 6–8 weeks after childbirth or on anxiety 6–8 weeks after childbirth compared with having a friend as a labour companion (non-RCT; very low-certainty evidence).

Maternal functioning/well-being, self-care and *health service use* were not reported in the included study.

Newborn/infant outcomes
No newborn/infant outcomes were reported in the included study.

Intra-household relationship dynamics
No outcomes relating to intra-household relationship dynamics were reported in the included study.

Comparison 6: Multicomponent interventions compared with no intervention or usual care

Maternal outcomes
Health service use: It is uncertain whether multicomponent interventions have an effect on women receiving any postnatal care from a skilled professional within two days after childbirth compared with no intervention or usual care (very low-certainty evidence).

Maternal morbidity, maternal functioning/well-being and *self-care* were not reported in the included studies.

Newborn/infant outcomes
Breastfeeding status
Breastfeeding initiation within the first hour of childbirth

- Evidence from RCTs: High-certainty evidence suggests multicomponent interventions have a positive effect on breastfeeding initiation within the first hour compared with no intervention or usual care (1 trial, 3449 newborns; RR 1.42, 95% CI 1.35 to 1.49).
- Evidence from non-RCTs: It is uncertain whether multicomponent interventions have any effect breastfeeding initiation within the first hour compared with no intervention or usual care (very low-certainty evidence).

Moderate-certainty evidence suggests multi-component interventions probably have a positive effect on exclusive breastfeeding at two months after childbirth compared with no intervention or usual care (1 trial, 70 newborns; RR 1.43, 95% CI 1.11 to 1.85).

Exclusive breastfeeding at four months after childbirth

- Evidence from RCTs: Low-certainty evidence suggests multicomponent interventions may make little or no difference to exclusive breastfeeding at four months after childbirth (1 trial, 70 newborns; RR 0.77, 95% CI 0.58 to 1.01) compared with no intervention or usual care.
- Evidence from non-RCTs: It is uncertain whether multicomponent interventions have any effect on exclusive breastfeeding at four months after childbirth compared with no intervention or usual care (very low-certainty evidence).

It is uncertain whether multicomponent interventions have any effect on early initiation of exclusive breastfeeding; exclusive breastfeeding at one month after childbirth; exclusive breastfeeding until six months after childbirth; or exclusive breastfeeding cessation at six months after childbirth, compared with no intervention or usual care (all non-RCTs; very low-certainty evidence).

Neonatal morbidity, family care practices, and *health service use* were not reported in the included studies.

Intra-household relationship dynamics
Father-child interaction and attachment: It is uncertain whether multicomponent interventions have any effect on father–infant play, caretaking, affection or attachment at one month after birth, compared with no intervention or usual care (all non-RCTs; very low-certainty evidence).

Couple communication and household decision-making, gender and power dynamics within couples and *adverse effects* were not reported in the included studies.

Values

See Box 3.14 in section 3.C: Health systems and health promotion interventions.

In addition, a qualitative evidence synthesis explored women, men, and health workers' perceptions of interventions to influence men's involvement in maternal and newborn health *(30)*. The findings indicated that men, women and health workers value access to maternal and newborn health services and quality maternal and newborn health care (high confidence in the evidence). Women, men and health workers also value the practical and emotional support provided by men to women and newborns (high confidence in the evidence). Men in particular value opportunities for enhanced father-newborn bonding (high confidence in the evidence). It is therefore highly likely that increased access to, and use of, maternal and newborn health services, as well as improved care of women and newborns in the home associated with improved men's engagement, will be valued by women and men as well as health workers. Good maternal and newborn health is important to most men (high confidence in the evidence), and if men believe that participating in the intervention and adopting behaviours recommended by the intervention will improve maternal and newborn health, then many men are happy to participate in interventions and adhere to intervention messages.

Resources

No economic evaluations of interventions to promote men's involvement in maternal and newborn health were identified.

Additional considerations

One study in the effectiveness review assessed costs associated with implementing strategies to improve existing antenatal care services in South Africa *(282)*, including the dissemination of information and education for couples and introduction of strengthened counselling for pregnant women and their partners through individual and group couples counselling. The overall costs of the intervention were almost 1 million Rand (amounting to US$ 73 000), including the costs associated with conducting the formative research, developing the intervention, training, supervision, monitoring and delivery of the group couples counselling sessions. The study asserts that in future years, the overall costs could be reduced. However, the cost per couple counselled would still be over 300 Rand, which would require further changes to make the intervention affordable *(282)*.

Table 3.89 Main resource requirements for interventions to promote men's involvement

Resource	Description
**Staff**	• Health workers (midwives, community/village health workers, nurses), trained peer educators, community volunteers, researchers, licensed parent educators, translators (where necessary), community mobilizers, male facilitators, tele-calling agency
**Training**	• Training of those delivering the intervention (including the training of trainers, e.g. for community-based information, education and communication interventions)<br>• Structured professional training for health workers (midwives/nurses/doctors)
**Supplies**	• Education materials (e.g. leaflets, booklets)<br>• Guides and other materials for group sessions (e.g. dolls, models)
**Equipment and infrastructure**	• Subject to intervention design, might require redesigning or enhancing organization of postnatal care, such as by establishing and integrating networks of community health workers, health visitors and social workers<br>• Might require structural changes to reduce overcrowding in health facilities or revisions of facility protocols to enable men's attendance
**Time**	• Time associated with training<br>• Time to deliver the intervention
**Supervision and monitoring**	• Supervision sessions and monitoring of visits

Equity

The qualitative evidence synthesis exploring women's, men's, and health workers' perceptions of interventions to influence men's involvement in maternal and newborn health *(30)* found evidence that interventions can support improved gender equity between women and men in couple or co-parent relationships. Interventions can help men and women to share the care of newborns more equally, by empowering men as confident caregivers, and supporting them to feel that their involvement in their child's care can be equal to that of the child's mother (moderate confidence in the evidence). Interventions can also foster mutual understanding and support, and facilitate communication and shared decision-making about maternal and newborn health (high confidence in the evidence). There is some evidence that increased mutual understanding and shared decision-making may contribute to more equitable couple or co-parent relationships.

The qualitative review also found evidence that risks of harm associated with poorly designed, targeted or implemented interventions are distributed inequitably, with these risks more likely to affect single women, women from low-income families, and women in settings and/or relationships characterized by gender power imbalances (moderate confidence in the evidence).

Women, men and families from poorer households are less able to access the reported benefits of facility-based interventions to involve men, including benefits relating to men's presence as labour companions, as these men are often in insecure paid work, under economic pressure to travel or migrate for work, or lack access to parental or carer's leave (low confidence in the evidence).

Interventions that seek to encourage partner attendance at antenatal appointments can stigmatize, and reduce the quality of services provided to, women who are not accompanied by a man (moderate confidence in the evidence).

Women in settings with highly gender-unequal social norms, and/or women in relationships likely to be characterized by a substantial gender power imbalance (including girls aged less than 18 years, girls and women in relationships with much older men, girls and women in polygamous relationships, and girls and women in violent or controlling relationships), are more likely to be negatively impacted by poorly designed interventions that perpetuate and/or extend the impact of existing unequal gender norms (moderate confidence in the evidence).

Acceptability

The qualitative evidence synthesis exploring women's, men's, and health workers' perceptions of interventions to influence men's involvement in maternal and newborn health (30) found that men and women may find interventions to influence men's engagement in maternal and newborn health acceptable because they believe that these interventions will contribute to good maternal and newborn health (high confidence in the evidence) and/or happy, mutually supportive couple relationships (low confidence in the evidence). Conversely, where women or men believe these interventions will increase conflict in their couple relationships, then they find the interventions less acceptable/unacceptable (low confidence in the evidence).

Some women, men and health workers may not want men to engage differently, because of beliefs that women have a greater capacity compared with men to provide care, particularly for newborns. Women may also be concerned about losing decision-making autonomy in the domain of maternal and newborn health and/or losing access to women-only spaces relating to maternal and newborn health that can provide a valued social space for women to connect with and support each other (low confidence in the evidence).

Established gender norms can make intervention messages about men being competent caregivers, sharing responsibility for maternal and newborn health, and providing support to women and care for newborns, unacceptable to some men, women and health workers (low confidence in the evidence).

Additional considerations

Additional findings from the qualitative synthesis note that the acceptability of interventions to men can change over time. Men are typically more comfortable with participating in interventions or adhering to intervention messages when they perceive it is common for men, particularly their peers, to be involved in maternal and newborn health. Information, coaching and encouragement, delivered through interventions, can increase men's confidence

to participate in further interventions and in maternal and newborn health.

Feasibility

The findings from the qualitative synthesis *(30)* indicate that interventions that promote men's participation in facility-based maternal and newborn health services are more feasible to implement where there is an enabling health system environment (high confidence in the evidence). In LMIC settings, there may be limited health system capacity to make maternal and newborn health services more men-friendly by investing in health worker training, changing service delivery, changing physical spaces, or offering new services. This may make men feel unwelcome and discouraged from participating.

Established gender norms that are supportive of women, and not men, being responsible for maternal and newborn health discourage some women and men from participating in interventions – either due to their own internalized norms, health workers' norms and attitudes, or pressure from peers, family and community members. This forms a demand-side barrier to men's and women's uptake of and adherence to interventions (low confidence in the evidence).

Some men are not available to participate in interventions due to insecure paid work, economic pressure to travel or migrate for paid work, or lack of access to parental leave. Tailoring interventions by location and time so that men are available may enable higher participation (low confidence in the evidence).

Table 3.90 Summary of judgements: Interventions to promote men's involvement in maternal and newborn health compared with no intervention, usual care or other intervention

Domain	Judgement
Desirable effects	Varies
Undesirable effects	Varies
Certainty of the evidence	Very low
Values	No important uncertainty or variability
Balance of effects	Probably favours interventions to promote men's involvement
Resources required	Varies
Certainty of the evidence on required resources	No included studies
Cost-effectiveness	Don't know
Equity	Varies
Acceptability	Varies
Feasibility	Probably yes

C.10 Home-based records

RECOMMENDATION 53

The use of home-based records, as a complement to facility-based records, is recommended for the care of pregnant and postpartum women, newborns and children, to improve care-seeking behaviour, men's involvement and support in the household, maternal and child home care practices, infant and child feeding, and communication between health workers and women, parents and caregivers. *(Recommended)*

Remarks

- This recommendation has been adapted and integrated from the 2018 *WHO recommendations on home-based records for maternal, newborn and child health (283)*, where the overall certainty of evidence was judged to be low.
- A home-based record – such as women-held case notes, vaccination cards, child health books or integrated maternal and child health books – is a health document used to record the history of health services received by an individual. It is kept in the household, in either paper or electronic format, by the individual or their caregiver and is intended to be integrated into the health information system and to complement records maintained by health facilities.
- The source guideline notes that there was insufficient evidence available to determine if any specific type, format or design of home-based record is more effective. It noted that policy-makers should involve stakeholders to discuss the important considerations relating to the type, content and implementation of home-based records.
- The following remarks were among those made by the Guideline Development Group responsible for the original recommendation.
  – Countries currently using home-based records should consider appropriate use, design and content, as well as sustainable financing, to maximize their use and impact.
  – In remote and fragile settings, where health systems are weak or where health information systems are absent or poor, and in locations where caregivers may use multiple health facilities, home-based records may be of greater value than in more developed settings and health systems.

C.11 Digital targeted client communication

RECOMMENDATION 54

WHO recommends digital targeted client communication for behaviour change regarding sexual, reproductive, maternal, newborn and child health, under the condition that concerns about sensitive content and data privacy are adequately addressed. *(Context-specific recommendation)*

Remarks

- This recommendation has been integrated from the 2019 WHO guideline *Recommendations on digital interventions for health system strengthening (284)*, where it was considered a context-specific recommendation.
- Digital targeted client communication refers to the transmission of customized health information for different audience segments (often based on health status or demographic categories). Targeted client communication may include:
  - transmission of health-event alerts to a specified population group;
  - transmission of health information based on health status or demographics;
  - alerts and reminders to clients; and/or
  - transmission of diagnostic results (or of the availability of results).
- The Guideline Development Group (GDG) responsible for the original recommendation considered this intervention to offer the potential to improve health behaviours and reduce inequities among individuals with access to mobile devices. However, it highlighted that measures should be taken to address inequities in access to mobile devices so that further inequity is not perpetuated in accessing health information and services, including mechanisms to ensure individuals who do not have access to mobile devices can still receive appropriate services.
- The GDG responsible for the original recommendation also raised the need to address potential concerns about sensitive content and data privacy, including potential negative unintended consequences. This could be done, for example, through mechanisms that actively allow individuals to opt out of services.

C.12 Digital birth notifications

RECOMMENDATION 55

WHO recommends the use of digital birth notifications under these conditions:

- **in settings where the notifications provide individual-level data to the health system and/or a civil registration and vital statistics (CRVS) system;**
- **the health system and/or CRVS system has the capacity to respond to the notifications.** *(Context-specific recommendation)*

Remarks

- This recommendation has been integrated from the 2019 WHO guideline *Recommendations on digital interventions for health system strengthening (284)*, where it was considered a context-specific recommendation.
- The source guideline notes the following.
  - Responses by the health system should include the capacity to accept the notifications and trigger appropriate health and social services, such as the initiation of postnatal services.
  - Responses by the civil registration and vital statistics (CRVS) system should include the capacity to accept the notifications and to validate the information, in order to trigger the subsequent process of birth registration and certification.
- The following remarks were made by the Guideline Development Group (GDG) responsible for the original recommendation.
  - The GDG acknowledged the limited evidence but emphasized that birth notification represents a vital first step in a care cascade that can ultimately lead to increased and timely access to health services and other social services. The GDG also believed that the use of mobile devices to perform this task was likely to provide a more expedient means of effecting the notification and subsequent health services.
  - The GDG members noted that while birth notification should not be viewed as a substitute for legal birth registration, it could provide an opportunity to accelerate the registration by linking birth notifications to national civil registration systems. The GDG also recognized that digital notification of births could facilitate providing newborns with a legal identity and future access to health and other social services.
  - It should also be noted that increases in the notification of births and deaths would require that civil registration services have, in turn, the capacity to manage a higher demand for registration and certification services.
  - The ability for the health system and/or CRVS system to respond and act appropriately on the birth and death notification was seen as a critical component for successful implementation. If such linkages are not in place, the notification of birth and death events would not add any value and would incur an additional cost for the system.

4. Implementation of the WHO postnatal care recommendations

This guideline aims to improve the quality of essential, routine postnatal care for women and newborns with the ultimate goal of improving maternal and newborn health and well-being. These recommendations need to be delivered within an appropriate model of postnatal care, adapted to the needs of different countries, local contexts, and individual women, newborns, parents, caregivers and families.

While the members of the Guideline Development Group (GDG) proposed implementation considerations for each recommendation (see Web Annex 5), they also reflected on considerations for the adoption, adaptation and implementation of the set of recommendations within this guideline to ensure availability, accessibility, acceptability and quality of postnatal care services for all women and newborns, in accordance with a human rights-based approach. Providers of postnatal health services must consider the needs of – and provide equal care to – all individuals and their newborns.

The WHO postnatal care model places the woman–newborn dyad at the centre of care (see Fig. 4.1). The foundation of this postnatal care model is Recommendation 44, which supports a minimum of four postnatal care contacts. The first contact refers to continued care in the health facility for at least the first 24 hours after birth or a first postnatal contact within the first 24 hours for a home birth. At least three additional postnatal care contacts occur between 48 and 72 hours, between 7 and 14 days, and during week six after birth. The overarching aim is to provide women, newborns, parents and caregivers with respectful, individualized, person-centred care at every contact. This includes the provision of effective clinical practices (assessments, referrals and management), relevant and timely information, and psychosocial and emotional support, by kind, competent and motivated health workers who are working within a well-functioning health system. An effective referral system, including communication between facility- and community-based care, and between health and transport systems in case of complications, are also essential components of this postnatal care model. Within this model, the word "contact" implies an active interaction between women, newborns, parents and caregivers, and care providers.

All the recommendations included in this document will require review by national, regional and local health system planners to ensure they are adapted, resourced and integrated into maternal, newborn and child health programmes. Several recommendations that are highlighted below will require a broad health systems approach and a strengthened focus on continuity of care, integrated service delivery, availability of supplies and commodities and empowered health workers. Implementation considerations for the WHO model can be found in Box 4.1.

In particular, the GDG considered the first two weeks after birth a key time to identify health problems and to support transition to well-woman and well-infant care. This current guideline confirms the importance of postnatal care during the first 24 hours after birth, regardless of the place of birth, and more specifically recommends a minimum 24-hour stay after birth in the health facility, with continuous care and monitoring during that stay (Recommendation 45). Expanded criteria before discharge have been identified to assess and manage potential problems and to prepare the transition to the home (Recommendations 46 and 47).

Whether the health system is set up for a home visit in the first week, or the woman and newborn need to seek routine outpatient postnatal care at the health facility or in the community, national discussions are encouraged to address the barriers and facilitators to ensure these critical contacts happen. A home visit for postnatal care within the first two days after birth, where feasible, has again been highlighted as critical to reduce mortality and morbidity, and to support the transition to the home. Some women and newborns will require additional contacts, or referral to specialized care, based on their health and needs.

Figure 4.1 Schematic representation of the WHO postnatal care model

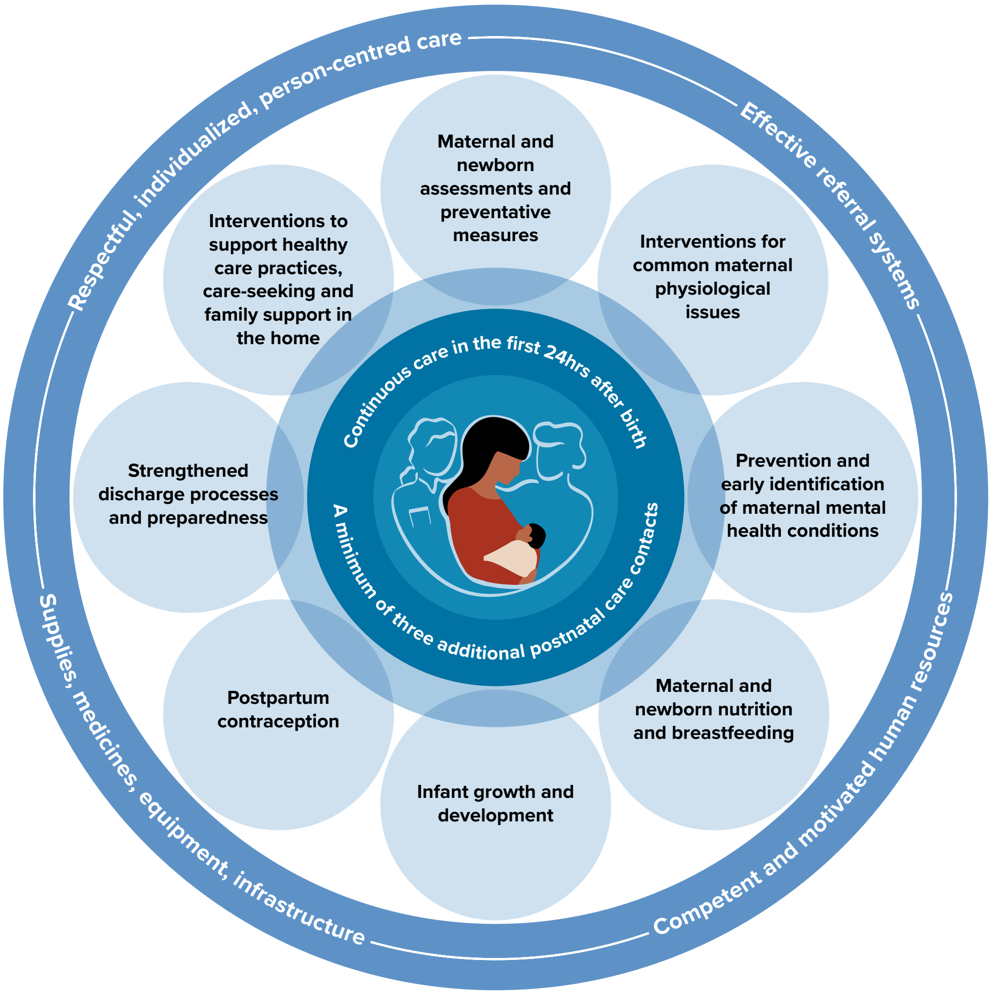

Health workers need to work as a team to address the needs of the woman and the newborn during the stay in the health facility and once they reach their home. This requires a functional relationship and communication between health workers and between the different levels of the system. The GDG highlighted the importance of establishing links with the health workers who will provide care and support after discharge, and to ensure seamless handover and transitions. One recommendation on midwifery continuity-of-care (Recommendation 49) for those contexts with strong midwifery programmes adds an additional level of relationship and trust-building between the team of midwives and the woman and family. Trained community health workers will also play a vital role in providing care and support in the home, and providing links between communities and health facilities.

The guideline also includes new recommendations on maternal and newborn assessments, including for common maternal mental health conditions (Recommendation 18) and newborn screening for hyperbilirubinaemia and eye and hearing conditions (Recommendations 26–29). Discussions are required on how to organize screening services depending on the condition and ensure that confirmatory diagnosis and subsequent treatment plus rehabilitation and follow-up is available.

In the context of humanitarian emergencies, the adaptation of the recommendations should consider their integration and alignment with other response strategies. Additional considerations should be made to the unique needs of women, newborns, parents, caregivers and families in emergency settings, including their values and preferences. Context-specific tools may be required in addition to standard tools to support the implementation by stakeholders of the recommendations in humanitarian emergencies.

Box 4.1 Considerations for the adoption, scale-up and implementation of the WHO postnatal care model

Health policy considerations for adoption and scale-up of the model

- A firm government commitment to scale up and increase coverage of postnatal care for all women and newborns is needed, irrespective of social, economic, ethnic, racial or other factors. National support must be secured for the whole package of recommendations, not just for specific components.
- To set the policy agenda, to secure broad anchoring and to ensure progress in policy formulation and decision-making, representatives of training facilities and the relevant medical specialties and professional societies should be included in participatory processes at all stages, including prior to an actual policy decision, to secure broad support for scaling-up.
- To facilitate negotiations and planning, situation-specific information on the expected impact of implementation of the postnatal care model on service users, health workers and costs should be compiled and disseminated.
- To be able to adequately ensure access for all women and newborns to quality postnatal care, in the context of universal health coverage, strategies for raising public funding for health care will need revision. In low-income countries, donors could play a significant role in supporting the scale-up of implementation. Sponsoring mechanisms that support domestically driven processes to scale up the whole model are more likely to be helpful than mechanisms that support only a part of the package.

Health system or organizational-level considerations for implementation of the model

- National and subnational subgroups may be established to adapt and implement these recommendations, including development or revision of existing national/sub-national guidelines or protocols based on the WHO postnatal care model.
- Long-term planning is needed for resource generation and budget allocation to address the shortage of skilled health personnel and trained community health workers, to improve facility infrastructure and referral pathways, and to strengthen and sustain high-quality postnatal care services.
- Introduction of the postnatal care model should involve pre-service training institutions and professional bodies, so that training curricula for postnatal care can be updated as quickly and smoothly as possible.
- In-service training and supervisory models will need to be developed according to health workers' professional requirements, considering the content, duration and procedures for the selection of health workers for training. These models can also be explicitly designed to address staff turnover, particularly in low-resource settings.
- Standardized tools will need to be developed for supervision, ensuring that supervisors are able to support and enable health workers to deliver integrated, comprehensive postnatal care services.
- A strategy for task sharing may need to be developed to optimize the use of human resources.
- Tools or "job aids" for implementation at the different levels of health facility care and communities will need to be developed or updated with all key information in accordance with the postnatal care model.
- Strategies will need to be devised to improve supply chain management according to local requirements, such as developing protocols for the procedures of obtaining and maintaining the stock of supplies, encouraging health workers to collect and monitor data on the stock levels and strengthening the provider-level coordination and follow-up of medicines and health-care supplies required for implementation of the postnatal care model.
- Development or revision of national guidelines and/or health facility-based protocols based on the WHO postnatal care recommendations is needed.
- Good-quality supervision, communication and transport links between community, primary and higher-level facilities need to be established to ensure that referral pathways are efficient.
- Successful implementation strategies should be documented and shared as examples of best practice for other implementers.

User-level considerations for implementation of the model

- Community-sensitizing activities should be undertaken to disseminate information about the importance of each component of postnatal care, and women's and babies' rights to receive postnatal care for their health and well-being. This information should provide details about the timing and content of the recommended contacts, and about the expected user fees.
- It may be possible to reduce waiting times by reorganizing postnatal care services and/or client flow.

Note: For specific implementation considerations related to the individual recommendations, see Web Annex 5.

5. Dissemination

This guideline will be disseminated through WHO regional and country offices, ministries of health, professional organizations, WHO collaborating centres, other United Nations agencies and nongovernmental organizations and professional associations. This guideline will be available on the WHO website and also as a printed publication. Online versions will be available via the websites of the WHO relevant departments. Technical meetings will be held between WHO and stakeholders to share the recommendations and derivative products. Updated recommendations are also routinely disseminated during meetings or scientific conferences attended by WHO maternal and perinatal staff.

Evidence briefs on selected recommendations will be developed for policy-makers and programme managers and for health workers. These evidence briefs, which will highlight the recommendations and implementation related contextual issues, will be developed and disseminated in collaboration with FIGO, ICM, IPA, UNFPA, UNICEF and USAID.

The executive summary and recommendations from this publication will be translated into the six United Nations languages for dissemination through the WHO regional and country offices.

In addition, a number of articles presenting the recommendations and key implementation considerations will be published, in compliance with WHO's open access and copyright policies. Relevant WHO clusters, departments and partnerships, such as the Partnership for Maternal, Newborn and Child Health (PMNCH), will also be part of this dissemination process.

WHO in collaboration with other partners, will support national and subnational working groups to adopt, adapt and implement the guideline. This will include the development or revision of existing national policies, guidelines or protocols in line with the WHO recommendations, as well as tools to support adaptation and implementation processes. This also includes technical support for local guideline implementers in the development of training materials, and quality indicators.

6. Applicability issues

A number of factors (barriers) may hinder the effective implementation and scale-up of this guideline. These factors may be related to the behaviours of women, parents, caregivers or families or health workers and to the organization of care or health service delivery. As part of efforts to implement this recommendation, health system stakeholders may wish to consider the following potential barriers:

- difficult access to health services and health workers for women and newborns including lack of transport, geographical conditions, financial barriers;
- lack of human resources with the necessary expertise and skills to implement, supervise and support recommended practices, including client counselling;
- lack of infrastructure to support interventions (e.g. lack of electricity for refrigeration; lack of access to clean water and sanitation; lack of access to digital interventions/devices; lack of physical space to conduct individual care and counselling)
- lack of time and/or understanding of the value of newly recommended interventions among health workers and health system administrators;
- lack of physical resources (e.g. equipment, supplies, medicines and nutritional supplements);
- lack of opportunities for continuing education and professional development for health workers;
- resistance of health workers to change from non-evidence-based to evidence-based practices (e.g. promoting dry cord care and avoiding unnecessary use of antibiotics);
- lack of effective referral mechanisms and care pathways for women and newborns identified as needing additional care (e.g. subsequent to universal screening for health conditions);
- lack of capacity to assess the mother and newborn together during postnatal contacts (e.g. where the mother attends the postnatal visit without the newborn);
- lack of health information management systems designed to document and monitor recommended practices (patient records, registers, etc.).

Given the potential barriers noted above, a phased approach to adoption, adaptation and implementation of the guideline recommendations may be prudent. Various strategies for addressing these barriers and facilitating implementation are provided in the lists of implementation considerations in Chapter 4 and Web Annex 5.

7. Monitoring and evaluating the impact of the guideline

The implementation and impact of these recommendations will be monitored at the health service, sub-national and national levels based on clearly defined criteria and indicators that are associated with locally-agreed targets. In collaboration with the monitoring and evaluation teams of the WHO Departments of MCA and SRH, data on country- and regional-level adoption of the recommendations will be collected and evaluated in the short to medium term across individual WHO Member States through the WHO Sexual, Reproductive, Maternal, Newborn, Child And Adolescent Health (SRMNCAH) Policy Survey. A full monitoring framework will be developed once the guidelines are finalized. In the meantime, the Guideline Development Group (GDG) suggests the following indicators to be considered, which have been adapted from current global recommended indicators.[49]

- *Length of stay in health facilities after childbirth:* The mean length of stay (days) in health facilities following childbirth; disaggregated by mode of birth (vaginal/caesarean birth).
- *Early routine postnatal care for women (within two days):* The percentage of women who have postnatal contact with a health worker within two days of birth; calculated as the number of women who have a postnatal contact with a health worker while in a health facility or at home following childbirth, or a postnatal care visit, within two days of childbirth of their most recent live birth in the *N* years prior to assessment, divided by the total number of women giving birth in the *N* years prior to assessment (regardless of place of birth).
- *Early routine postnatal care for newborns (within two days):* The percentage of newborns who have postnatal contact with a health worker within two days of birth; calculated as the number of newborns who have postnatal contact with a health worker while in health facility or at home following birth, or a postnatal care visit, within two days after birth, divided by the total number of last live births in the *N* years prior assessment (regardless of place of birth).
- *Hepatitis B birth dose vaccination:* The percentage of children who have received the first dose of the hepatitis B vaccine in the first 24 hours after birth.

It is important to note there are limitations with these indicators which will be reviewed as the monitoring framework is developed. The Mother and Newborn Information for Tracking Outcomes and Results (MoNITOR) group will be consulted to identify the recommended indicators for measuring postnatal care coverage as well indicators for the quality of care that capture core content of postnatal care contacts with the woman and newborn.

49 WHO maternal, newborn, child and adolescent health and ageing data portal: www.who.int/data/maternal-newborn-child-adolescent-ageing/maternal-and-newborn-data/maternal-and-newborn---coverage.

8. Updating of the guideline

In accordance with the procedures for updating WHO guidelines, a systematic and continuous process of identifying and bridging evidence gaps following guideline implementation will be employed. An Executive Guideline Steering Group (GSG) for maternal and newborn health recommendations will convene biannually to review WHO's current portfolio of maternal and newborn health recommendations, and to prioritize new and existing questions for recommendation development and updating, particularly for recommendations supported by very low- or low-certainty evidence, where new recommendations or a change in the published recommendations may be warranted. Accordingly, the recommendations included in this guideline will be regularly reviewed and prioritized as needed by the Executive GSG. In the event that new evidence (that could potentially impact the current evidence base for any of the recommendations) is identified, the recommendation will be updated. If no new reports or information are identified for a particular recommendation, the recommendation will be revalidated.

Any concern about the validity of any recommendation will be promptly communicated via the website for the guideline,[50] and plans will be made to update the recommendation, as needed. WHO welcomes suggestions regarding additional questions for inclusion in future updates of this guideline; suggestions can be addressed by email to WHO MCA (mncah@who.int) and WHO SRH (srhmph@who.int).

50 Available at: https://www.who.int/publications/i/item/9789240045989

9. References

1. Global strategy for women's, children's and adolescents' health (2016–2030). New York (NY): Every Woman Every Child; 2015.

2. Strategies toward ending preventable maternal mortality. Geneva: World Health Organization; 2015.

3. Every Newborn: an action plan to end preventable deaths. Geneva: World Health Organization; 2014.

4. Comprehensive implementation plan on maternal, infant and young child nutrition. Geneva: World Health Organization; 2014.

5. World Health Organization (WHO), United Nations Children's Fund, World Bank Group. Nurturing care for early childhood development: a framework for helping children survive and thrive to transform health and human potential. Geneva: WHO; 2018.

6. WHO technical consultation on postpartum and postnatal care. Geneva: World Health Organization; 2010.

7. Gon G, Leite A, Calvert C, Woodd S, Graham WJ, Filippi V. The frequency of maternal morbidity: a systematic review of systematic reviews. Int J Gynaecol Obstet. 2018;141(Suppl.1):20–38. doi:10.1002/ijgo.12468.

8. Kassebaum NJ, Bertozzi-Villa A, Coggeshall MS, Shackelford KA, Steiner C, Heuton KR, et al. Global, regional, and national levels and causes of maternal mortality during 1990–2013: a systematic analysis for the Global Burden of Disease Study 2013. Lancet. 2014;384(9947):980–1004. doi:10.1016/S0140-6736(14)60696-6.

9. Hug L, Alexander M, You D, Alkema L. National, regional, and global levels and trends in neonatal mortality between 1990 and 2017, with scenario-based projections to 2030: a systematic analysis. Lancet Glob Health. 2019;7(6):e710–20. doi:10.1016/S2214-109X(19)30163-9.

10. United Nations Children's Fund (UNICEF), World Health Organization, World Bank Group and United Nations Inter-agency Group for Child Mortality Estimation. Levels and trends in child mortality: report 2020. New York: (NY): UNICEF; 2020.

11. Campbell OM, Cegolon L, Macleod D, Benova L. Length of stay after childbirth in 92 countries and associated factors in 30 low- and middle-income countries: compilation of reported data and a cross-sectional analysis from nationally representative surveys. PLoS Med. 2016;13(3):e1001972. doi:10.1371/journal.pmed.1001972.

12. Requejo J, Diaz T, Park L, Chou D, Choudhury A, Guthold R, et al. Assessing coverage of interventions for reproductive, maternal, newborn, child, and adolescent health and nutrition. BMJ. 2020;368:l6915. doi:10.1136/bmj.l6915.

13. Ending preventable newborn deaths and stillbirths by 2030. Moving faster towards high-quality universal health coverage in 2020–2025. New York (NY) and Geneva: United Nations Children's Fund, World Health Organization; 2020.

14. United Nations Sustainable Development Goals [website]. Sustainable Development Goals (https://www.un.org/sustainabledevelopment/sustainable-development-goals/, accessed 20 January 2022).

15. WHO recommendations on postnatal care of the mother and newborn. Geneva: World Health Organization; 2014.

16. WHO recommendations on antenatal care for a positive pregnancy experience. Geneva: World Health Organization; 2016.

17. WHO recommendations on intrapartum care for a positive childbirth experience. Geneva: World Health Organization; 2018.

18. Postnatal care NICE guideline NG194. National Institute for Health and Care Excellence; 2021.

19. WHO handbook for guideline development. Geneva: World Health Organization; 2014.

20. Standards for improving quality of maternal and newborn care in health facilities. Geneva: World Health Organization; 2016.

21. Finlayson K, Crossland N, Bonet M, Downe S. What matters to women in the postnatal period: a meta-synthesis of qualitative studies. PLoS One. 2020;15(4):e0231415. doi:10.1371/journal.pone.0231415.

22. Harvey C, Movsisyan A, Portela A, Smith H. Stakeholder perspectives on discharge preparation and discharge readiness after birth: a qualitative evidence synthesis (in preparation).

23. Alonso-Coello P, Oxman AD, Moberg J, Brignardello-Petersen R, Akl EA, Davoli M, et al.; GRADE Working Group. GRADE Evidence to Decision (EtD) frameworks: a systematic and transparent approach to making well informed healthcare choices. 2: Clinical practice guidelines. BMJ. 2016;353:i2089. doi:10.1136/bmj.i2089.

24. State of inequality: reproductive, maternal, newborn and child health. Geneva: World Health Organization; 2015.

25. GRADE Working Group [website] (http://gradeworkinggroup.org/, accessed 20 January 2022).

26. GRADE-CERQual Project Group [website] (https://www.cerqual.org, accessed 20 January 2022).

27. Evers S, Goossens M, de Vet H, van Tulder M, Ament A. Criteria list for assessment of methodological quality of economic evaluations: consensus on health economic criteria. Int J Technol Assess Health Care. 2005;21(2):240–5.

28. Sacks E, Langlois E, Ziegler D, Sauve C, Downe S, Finlayson K, et al. Factors that influence uptake of routine postnatal care: a qualitative evidence synthesis (submitted).

29. Munabi-Babigumira S, Lewin S, Glenton C, Velez M, Gonçalves-Bradley DC, Bohren MA. Factors that influence the provision of postnatal care by health workers: a qualitative evidence synthesis [protocol]. Cochrane Database Syst Rev. 2021;(7):CD014790.

30. Comrie-Thomson L, Gopal P, Eddy K, Baguiya A, Gerlach N, Sauvé C, et al. How do women, men and health providers perceive interventions to influence men's engagement during pregnancy, childbirth and postnatal to improve maternal and newborn health outcomes? A qualitative evidence synthesis. Soc Sci Med. 2021;291:114475. doi:10.1016/j.socscimed.2021.114475.

31. Prady SL, Uphoff EP, Power M, Golder S. Development and validation of a search filter to identify equity-focused studies: reducing the number needed to screen. BMC Med Res Methodol. 2018;18(1):106. doi:10.1186/s12874-018-0567-x.

32. Higgins JPT, Thomas J, Chandler J, Cumpston M, Li T, Page MJ, Welch VA, editors. Cochrane handbook for systematic reviews of interventions. Version 6.1 (updated September 2020). Cochrane; 2020.

33. Walsh D, Downe S. Appraising the quality of qualitative research. Midwifery. 2006;22(2):108–19. doi:10.1016/j.midw.2005.05.004.

34. OneHealth Model: intervention treatment assumptions (draft 28 September). Geneva, Glastonbury (CT): United Nations InterAgency Working Group on Costing and the Futures Institute; 2013.

35. WHO compendium of innovative health technologies for low-resource settings. Geneva: World Health Organization; 2015.

36. Langlois ÉV, Miszkurka M, Zunzunegui MV, Ghaffar A, Ziegler D, Karp I. Inequities in postnatal care in low- and middle-income countries: a systematic review and meta-analysis. Bull World Health Organ. 2015;93(4):259–70G. doi:10.2471/BLT.14.140996.

37. Declarations of interest. In: About WHO – ethics [website]. Geneva: World Health Organization; 2020 (https://www.who.int/about/ethics/declarations-of-interest, accessed 20 January 2022).

38. Reporting the effects of an intervention in EPOC reviews. Cochrane Effective Practice and Organisation of Care; 2018.

39. Integrated management of pregnancy and childbirth: pregnancy, childbirth, postpartum and newborn care: a guide for essential practice, third edition. Geneva: World Health Organization; 2015.

40. WHO recommendations for the prevention and treatment of postpartum haemorrhage. Geneva: World Health Organization; 2012.

41. Consolidated guidelines on HIV testing services. Geneva: World Health Organization; 2019.

42. Consolidated guidelines on the use of antiretroviral drugs for treating and preventing HIV infection. Recommendations for a public health approach, second edition. Geneva: World Health Organization; 2016.

43. WHO consolidated guidelines on tuberculosis. Module 2: screening. Systematic screening for tuberculosis disease. Geneva: World Health Organization; 2021.

44. Manresa M, Pereda A, Bataller E, Terre-Rull C, Ismail KM, Webb SS. Incidence of perineal pain and dyspareunia following spontaneous vaginal birth: a systematic review and meta-analysis. Int Urogynecol J. 2019;30(6):853–68. doi:10.1007/s00192-019-03894-0.

45. East CE, Dorward EDF, Whale RE, Liu J. Local cooling for relieving pain from perineal trauma sustained during childbirth. Cochrane Database Syst Rev. 2020;(10):CD006304.

46. Deussen AR, Ashwood P, Martis R, Stewart F, Grzeskowiak LE. Relief of pain due to uterine cramping/involution after birth. Cochrane Database Syst Rev. 2020;(10):CD004908.

47. Milsom I, Altman D, Cartwright R, Lapitan MC, Nelson R, Sjöström S, et al., editors. Epidemiology of urinary incontinence (UI) and other lower urinary tract symptoms (LUTS), pelvic organ prolapse (POP) and anal incontinence (AI). In: Abrams P, Cardozo L, Wagg A, Wein A, editors. Incontinence: 6th International Consultation on Incontinence 2017; Tokyo: International Continence Society; 2017.

48. Woodley SJ, Lawrenson P, Boyle R, Cody JD, Mørkved S, Kernohan A, et al. Pelvic floor muscle training for preventing and treating urinary and faecal incontinence in antenatal and postnatal women. Cochrane Database Syst Rev. 2020;(5):CD007471.

49. Zakarija-Grkovic I, Stewart F. Treatments for breast engorgement during lactation. Cochrane Database Syst Rev. 2020;(9):CD006946.

50. Crepinsek MA, Taylor EA, Michener K, Stewart F. Interventions for preventing mastitis after childbirth. Cochrane Database Syst Rev. 2020;(9):CD007239.

51. Abalos E, Gyte GML, Sguassero Y. Paracetamol/acetaminophen (single administration) for perineal pain in the early postpartum period. Cochrane Database Syst Rev. 2021;(1):CD008407.

52. Shepherd E, Grivell RM. Aspirin (single dose) for perineal pain in the early postpartum period. Cochrane Database Syst Rev. 2020;(7):CD012129.

53. Wuytack F, Smith V, Cleary BJ. Oral non-steroidal anti-inflammatory drugs (single dose) for perineal pain in the early postpartum period. Cochrane Database Syst Rev. 2021;(1):CD011352.

54. Yildizhan R, Yildizhan B, Sahin S, Suer N. Comparison of the efficacy of diclofenac and indomethacin suppositories in treating perineal pain after episiotomy or laceration: a prospective, randomized, double-blind clinical trial. Arch Gynecol Obstet. 2009;280(5):735. doi:10.1007/s00404-009-1006-3.

55. Hedayati H, Parsons J, Crowther CA. Rectal analgesia for pain from perineal trauma following childbirth. Cochrane Database Syst Rev. 2003;(3):CD003931.

56. Hedayati H, Parsons J, Crowther CA. Topically applied anaesthetics for treating perineal pain after childbirth. Cochrane Database Syst Rev. 2005;(2):CD004223.

57. Medical Sciences for Health (MSH). International medical products price guide, 2015 edition. Medford: MSH; 2016.

58. World Health Organization Model List of Essential Medicines, 21st list. Geneva: World Health Organization; 2019.

59. Briggs GG, Freeman RK, Yaffe SJ. Drugs in pregnancy and lactation: a reference guide to fetal and neonatal risk, eighth edition. Philadelphia (PA): Lippincott Williams & Wilkins; 2008.

60. Moossdorff-Steinhauser HFA, Bols EMJ, Spaanderman MEA, Dirksen CD, Weemhoff M, Nieman FHM, et al. Long-term effects of motherfit group therapy in pre-(MOTHERFIT1) and post-partum women (MOTHERFIT2) with stress urinary incontinence compared to care-as-usual: study protocol of two multi-centred, randomised controlled trials. Trials. 2019;20(1):237. doi:10.1186/s13063-019-3331-6.

61. Salmon VE, Hay-Smith EJC, Jarvie R, Dean S, Terry R, Frawley H, et al. Implementing pelvic floor muscle training in women's childbearing years: a critical interpretive synthesis of individual, professional, and service issues. Neurourol Urodynam. 2020;39(2):863–70. doi:10.1002/nau.24256.

62. Hay-Smith J, Dean S, Burgio K, McClurg D, Frawley H, Dumoulin C. Pelvic-floor-muscle-training adherence "modifiers": a review of primary qualitative studies – 2011 ICS state-of-the-science seminar research paper III of IV. Neurourol Urodyn. 2015;34(7):622–31. doi:10.1002/nau.22771.

63. Responsive feeding infosheet [fact sheet]. Supporting close and loving relationships. London: United Nations Children's Fund UK; 2016 (https://www.unicef.org.uk/babyfriendly/baby-friendly-resources/relationship-building-resources/responsive-feeding-infosheet/, accessed 20 January 2022).

64. The evidence and rationale for the UNICEF UK Baby Friendly Initiative standards. London: United Nations Children's Fund UK; 2013 (https://www.unicef.org.uk/babyfriendly/about/evidence-and-rationale-for-the-baby-friendly-standards/, accessed 20 January 2022).

65. Guttman N, Zimmerman DR. Low-income mothers' views on breastfeeding. Soc Sci Med. 2000;50(10):1457–73. doi:10.1016/S0277-9536(99)00387-1.

66. Mitra AK, Khoury AJ, Hinton AW, Carothers C. Predictors of breastfeeding intention among low-income women. Matern Child Health J. 2004;8(2):65–70. doi:10.1023/B:MACI.0000025728.54271.27.

67. Khosravan S, Mohammadzadeh-Moghadam H, Mohammadzadeh F, Fadafen SAK, Gholami M. The effect of Hollyhock (Althaea Officinalis L) leaf compresses combined with warm and cold compress on breast engorgement in lactating women: a randomized clinical trial. J Evid Based Complementary Altern Med. 2017;22(1):25–30. doi:10.1177/2156587215617106.

68. Dehghani M, Babazadeh R, Khadivzadeh T, Pourhoseini SA, Esmaeili H. Effect of breast oketani-massage on the severity of breast engorgement. Iran J Obstet Gynecol Infertil. 2017;20(5):30–8. doi:10.22038/ijogi.2017.9078.

69. Lang DL, Zhao F-L, Robertson J. Prevention of postpartum haemorrhage: cost consequences analysis of misoprostol in low-resource settings. BMC Pregnancy Childbirth. 2015;15:305. doi:10.1186/s12884-015-0749-z.

70. Gallos I, Williams H, Price M, Pickering K, Merriel A, Tobias A, et al. Uterotonic drugs to prevent postpartum haemorrhage: a network meta-analysis. Health Technol Assess. 2019;23:9. doi:10.3310/hta23090.

71. Bhagat S, Agarwal M, Roy V. Serratiopeptidase: a systematic review of the existing evidence. Int J Surg. 2013;11(3):209–17. doi:10.1016/j.ijsu.2013.01.010.

72. Wilson E, Woodd SL, Benova L. Incidence of and risk factors for lactational mastitis: a systematic review. J Hum Lact. 2020;36(4):673–86. doi:10.1177/0890334420907898.

73. Higgins PD, Johanson JF. Epidemiology of constipation in North America: a systematic review. Am J Gastroenterol. 2004;99(4):750–9. doi:10.1111/j.1572-0241.2004.04114.x.

74. Turawa EB, Musekiwa A, Rohwer AC. Interventions for preventing postpartum constipation. Cochrane Database Syst Rev. 2020;(8):CD011625.

75. Guideline: protecting, promoting and supporting breastfeeding in facilities providing maternity and newborn services. Geneva: World Health Organization; 2017.

76. Guideline: counselling of women to improve breastfeeding practices. Geneva: World Health Organization; 2018.

77. Jahanfar S, Ng CJ, Teng CL. Antibiotics for mastitis in breastfeeding women. Cochrane Database Syst Rev. 2013;(2):CD005458.

78. Healthy diet [fact sheet]. Geneva: World Health Organization; 2020 (https://www.who.int/en/news-room/fact-sheets/detail/healthy-diet, accessed 20 January 2022).

79. WHO recommendations for augmentation of labour. Geneva: World Health Organization; 2014.

80. Downe S, Finlayson K, Tunçalp Ö, Gülmezoglu AM. Provision and uptake of routine antenatal services: a qualitative evidence synthesis. Cochrane Database Syst Rev. 2019;(6):CD012392.

81. Breastfeeding and maternal medication. Recommendations for drugs in the eleventh WHO model list of essential drugs. Geneva: World Health Organization; 2002.

82. WHO recommendations for prevention and treatment of maternal peripartum infections. Geneva: World Health Organization; 2015.

83. Preventive chemotherapy to control soil-transmitted helminth infections in at-risk population groups. Geneva: World Health Organization; 2017.

84. WHO guideline on control and elimination of human schistosomiasis. Geneva: World Health Organization; 2022.

85. Fisher J, Cabral de Mello M, Patel V, Rahman A, Tran T, Holton S, et al. Prevalence and determinants of common perinatal mental disorders in women in low- and lower-middle-income countries: a systematic review. Bull World Health Organ. 2012;90(2):139–149G. doi:10.2471/BLT.11.091850.

86. Muzik M, Thelen K, Rosenblum KL. Perinatal depression: detection and treatment. Neuropsychiatry. 2011;1(2):179–95. doi:10.2217/npy.10.8.

87. Andermann A, Blancquaert I, Beauchamp S, Déry V. Revisiting Wilson and Jungner in the genomic age: a review of screening criteria over the past 40 years. Bull World Health Organ. 2008;86(4):317–9. doi:10.2471/blt.07.050112.

88. Waqas A, Koukab A, Meraj H, Dua T, Chowdhary N, Fatima B, et al. Screening programs for common maternal mental health disorders among perinatal women: report of the systematic review of evidence. BMC Psychiatry. 2022;22(1):54. doi:10.1186/s12888-022-03694-9.

89. Morrell CJ, Warner R, Slade P, Dixon S, Walters S, Paley G, et al. Psychological interventions for postnatal depression: cluster randomised trial and economic evaluation. The PoNDER trial. Health Technol Assess. 2009;13(30):iii–iv,xi–xiii,1–153. doi:10.3310/hta13300.

90. World Health Organization (WHO), Calouste Gulbenkian Foundation. Social determinants of mental health. Geneva: WHO; 2014.

91. Smith HJJ, Portela AG, Harvey C. Discharge preparation and readiness after birth: a scoping review of global policies, guidelines and literature. BMC Pregnancy Childbirth (in press).

92. Armstrong SJ, Small RE. The paradox of screening: rural women's views on screening for postnatal depression. BMC Public Health. 2010;10(1):744. doi:10.1186/1471-2458-10-744.

93. Buist A, Condon J, Brooks J, Speelman C, Milgrom J, Hayes B, et al. Acceptability of routine screening for perinatal depression. J Affect Disord. 2006;93(1–3):233–7. doi:10.1016/j.jad.2006.02.019.

94. Waqas A, Kokab A, Meraj H, Dua T, Chowdhary N, Fatima B, et al. Prevention of common mental disorders among women in the perinatal period: a critical mixed-methods review and meta-analysis. Glob Ment Health (in press).

95. Dukhovny D, Dennis CL, Hodnett E, Weston J, Stewart DE, Mao W, et al. Prospective economic evaluation of a peer support intervention for prevention of postpartum depression among high-risk women in Ontario, Canada. Am J Perinatol. 2013;30(8):631–42. doi:10.1055/s-0032-1331029.

96. Ride J, Lorgelly P, Tran T, Wynter K, Rowe H, Fisher J. Preventing postnatal maternal mental health problems using a psychoeducational intervention: the cost-effectiveness of What Were We Thinking. BMJ Open. 2016;6(11):e012086. doi:10.1136/bmjopen-2016-012086.

97. Delaney AM, George Dalmida S, Gaydos L. When it is not postpartum depression: understanding the postpartum mental health needs of mothers and how they are being met. Issues Ment Health Nurs. 2015;36(6):416–23. doi:10.3109/01612840.2014.1002645.

98. Raymond NC, Pratt RJ, Godecker A, Harrison PA, Kim H, Kuendig J, et al. Addressing perinatal depression in a group of underserved urban women: a focus group study. BMC Pregnancy Childbirth. 2014;14:336. doi:10.1186/1471-2393-14-336.

99. Thurtle V. First time mothers' perceptions of motherhood and PND. Community Practitioner. 2003;76:261–5.

100. Iron supplementation in postpartum women. Geneva: World Health Organization; 2016.

101. Daily iron supplementation in adult women and adolescent girls. Geneva: World Health Organization; 2016.

102. Intermittent iron and folic acid supplementation in menstruating women. Geneva: World Health Organization; 2011.

103. Haemoglobin concentrations for the diagnosis of anaemia and assessment of severity. Vitamin and Mineral Nutrition Information System. Geneva: World Health Organization; 2011.

104. International Nutritional Anemia Consultative Group, World Health Organization, United Nations Children's Fund. Guidelines for the use of iron supplements to prevent and treat iron deficiency anaemia. Washington (DC): ILSI Press; 1998.

105. Healthy eating during pregnancy and breastfeeding. Booklet for mothers. Copenhagen: World Health Organization; 2001.

106. Vitamin A supplementation in postpartum women. Geneva: World Health Organization; 2011.

107. WHO guidelines on physical activity and sedentary behaviour. Geneva: World Health Organization; 2020.

108. Ensuring human rights in the provision of contraceptive information and services: guidance and recommendations. Geneva: World Health Organization; 2014.

109. Medical eligibility criteria for contraceptive use. Geneva: World Health Organization; 2015.

110. Contraceptive eligibility for women at high risk of HIV. Geneva: World Health Organization; 2019.

111. WHO consolidated guideline on self-care interventions for health: sexual and reproductive health and rights. Geneva: World Health Organization; 2019.

112. Rahi J, Gilbert CE. Epidemiology and the worldwide impact of visual impairment in children. In: Lyons CJ, Lambert SR, editors. Taylor and Hoyt's Pediatric Ophthalmology and Strabismus, sixth edition [e-book]. London, New York, Oxford, Philadelphia, St Louis, Sydney, Toronto: Elsevier; 2017.

113. Malik ANJ, Evans JR, Gupta S, Mariotti S, Gordon I, Bowman R, et al. Universal newborn eye screening: a systematic review of the literature and review of international guidelines (submitted).

114. Lieu JEC, Kenna M, Anne S, Davidson L. Hearing loss in children: a review. JAMA. 2020;324(21):2195–205. doi:10.1001/jama.2020.17647.

115. Butcher E, Dezateux C, Cortina-Borja M, Knowles RL. Prevalence of permanent childhood hearing loss detected at the universal newborn hearing screen: Systematic review and meta-analysis. PLoS One. 2019;14(7):e0219600. doi:10.1371/journal.pone.0219600.

116. World report on hearing. Geneva: World Health Organization; 2021.

117. Bhutani VK, Zipursky A, Blencowe H, Khanna R, Sgro M, Ebbesen F, et al. Neonatal hyperbilirubinemia and Rhesus disease of the newborn: incidence and impairment estimates for 2010 at regional and global levels. Pediatr Res. 2013;74 Suppl 1(Suppl 1):86–100. doi:10.1038/pr.2013.208.

118. Olusanya BO, Kaplan M, Hansen TWR. Neonatal hyperbilirubinaemia: a global perspective. Lancet Child Adolesc Health. 2018;2(8):610–20. doi:10.1016/s2352-4642(18)30139-1.

119. Sun M, Ma A, Li F, Cheng K, Zhang M, Yang H, et al. Sensitivity and specificity of red reflex test in newborn eye screening. J Pediatr. 2016;179:192–6.e4. doi:10.1016/j.jpeds.2016.08.048.

120. Haargaard B, Nyström A, Rosensvärd A, Tornqvist K, Magnusson G. The Pediatric Cataract Register (PECARE): analysis of age at detection of congenital cataract. Acta Ophthalmol. 2015;93(1):24–6. doi:10.1111/aos.12445.

121. Magnusson G, Persson U. Screening for congenital cataracts: a cost-consequence analysis of eye examination at maternity wards in comparison to well-baby clinics. Acta Paediatrica. 2005;94(8):1089–95. doi:10.1111/j.1651-2227.2005.tb02050.x.

122. American Academy of Pediatrics. Red reflex examination in neonates, infants, and children. Pediatrics. 2008;122(6):1401–4. doi:10.1542/peds.2008-2624.

123. UNICEF supply catalogue [website]. United Nations Children's Fund; 2018 (https://supply.unicef.org, accessed 20 January 2022).

124. Lowe J, Cleland CR, Mgaya E, Furahini G, Gilbert CE, Burton MJ, et al. The Arclight ophthalmoscope: a reliable low-cost alternative to the standard direct ophthalmoscope. J Ophthalmol. 2015;2015:743263. doi:10.1155/2015/743263.

125. Kong L, Fry M, Al-Samarraie M, Gilbert C, Steinkuller PG. An update on progress and the changing epidemiology of causes of childhood blindness worldwide. JAAPOS. 2012;16(6):501–7. doi:10.1016/j.jaapos.2012.09.004.

126. Gilbert CE, Lepvrier-Chomette N. Gender inequalities in surgery for bilateral cataract among children in low-income countries: a systematic review. Ophthalmology. 2016;123(6):1245–51. doi:10.1016/j.ophtha.2016.01.048.

127. Fabian ID, Abdallah E, Abdullahi SU, Abdulqader RA, Adamou Boubacar S, Ademola-Popoola DS, et al.; Global Retinoblastoma Study Group. Global retinoblastoma presentation and analysis by national income level. JAMA Oncol. 2020;6(5):685–95. doi:10.1001/jamaoncol.2019.6716.

128. Malik ANJ, Mafwiri M, Gilbert C, Kim MJ, Schellenberg J. Integrating eye health training into the primary child healthcare programme in Tanzania: a pre-training and post-training study. BMJ Paediatr Open. 2020;4(1):e000629. doi:10.1136/bmjpo-2019-000629.

129. Mndeme FG, Mmbaga BT, Kim MJ, Sinke L, Allen L, Mgaya E, et al. Red reflex examination in reproductive and child health clinics for early detection of paediatric cataract and ocular media disorders: cross-sectional diagnostic accuracy and feasibility studies from Kilimanjaro, Tanzania. Eye. 2021;35:1347–53. doi:10.1038/s41433-020-1019-5.

130. Universal Newborn Hearing Screening (UNHS) review group. Effectiveness of universal newborn hearing screening: a systematic review and meta-analysis (in preparation).

131. Nelson HD, Bougatsos C, Nygren P. Universal newborn hearing screening: systematic review to update the 2001 US Preventive Services Task Force Recommendation. Pediatrics. 2008;122(1):e266–76. doi:10.1542/peds.2007-1422.

132. Patel H, Feldman M, Canadian Paediatric S, Community Paediatrics C. Universal newborn hearing screening. Paediatr Child Health. 2011;16(5):301–5. doi:10.1093/pch/16.5.301.

133. Sharma R, Gu Y, Ching TYC, Marnane V, Parkinson B. Economic evaluations of childhood hearing loss screening programmes: a systematic review and critique. Appl Health Econ. 2019;17(3):331–57. doi:10.1007/s40258-018-00456-1.

134. Olusanya BO, Emokpae A, Renner JK, Wirz SL. Costs and performance of early hearing detection programmes in Lagos, Nigeria. Trans R Soc Trop Med Hyg. 2009;103(2):179–86. doi:10.1016/j.trstmh.2008.07.001.

135. Heidari S, Olyaee Manesh A, Rajabi F, Moradi-Joo M. Cost-effectiveness analysis of automated auditory brainstem response and otoacoustic emission in universal neonatal hearing screening. Iran J Pediatr. 2017;27(2):e5229. doi:10.5812/ijp.5229.

136. Gupta S, Sah S, Som T, Saksena M, Yadav CP, Sankar MJ, et al. Challenges of implementing universal newborn hearing screening at a tertiary care centre from India. Indian J Pediatr. 2015;82(8):688–93. doi:10.1007/s12098-015-1688-4.

137. Verkleij ML, Heijnsdijk EAM, Bussé AML, Carr G, Goedegebure A, Mackey AR, et al. Cost-effectiveness of neonatal hearing screening programs: a micro-simulation modeling analysis. Ear Hear. 2021;42(4):909–16. doi:10.1097/AUD.0000000000000981.

138. Coates H, Gifkins K. Newborn hearing screening. Australian Prescriber. 2003;26(4):82–4. doi:10.18773/austprescr.2003.062.

139. Olusanya BO, Newton VE. Global burden of childhood hearing impairment and disease control priorities for developing countries. Lancet. 2007;369(9569):1314–7. doi:10.1016/S0140-6736(07)60602-3.

140. Van Kerschaver E, Boudewyns AN, Declau F, Van de Heyning PH, Wuyts FL. Socio-demographic determinants of hearing impairment studied in 103 835 term babies. Eur J Public Health. 2012;23(1):55–60. doi:10.1093/eurpub/cks010.

141. Lantos PM, Maradiaga-Panayotti G, Barber X, Raynor E, Tucci D, Hoffman K, et al. Geographic and racial disparities in infant hearing loss. Otolaryngol Head Neck Surg. 2018:194599818803305. doi:10.1177/0194599818803305.

142. Frary CD, Thomsen P, Gerke O. Risk factors for non-participation in the Danish universal newborn hearing screening program: a population-based cohort study. Int J Pediatr Otorhinolaryngol. 2020;135:110079. doi:10.1016/j.ijporl.2020.110079.

143. Young A, Tattersall H. Parents' of deaf children evaluative accounts of the process and practice of universal newborn hearing screening. J Deaf Stud Deaf Educ. 2005;10(2):134–45. doi:10.1093/deafed/eni014.

144. Young A, Tattersall H. Universal newborn hearing screening and early identification of deafness: parents' responses to knowing early and their expectations of child communication development. J Deaf Stud Deaf Educ. 2007;12(2):209–20. doi:10.1093/deafed/enl033.

145. Arnold CL, Davis TC, Humiston SG, Bocchini JA, Jr., Bass PF, 3rd, Bocchini A, et al. Infant hearing screening: stakeholder recommendations for parent-centered communication. Pediatrics. 2006;117(5 Pt 2):S341–54. doi:10.1542/peds.2005-2633N.

146. Jatto ME, Ogunkeyede SA, Adeyemo AA, Adeagbo K, Saiki O. Mothers' perspectives of newborn hearing screening programme. Ghana Med J. 2018;52(3):158–62. doi:10.4314/gmj.v52i3.9.

147. Shulman S, Besculides M, Saltzman A, Ireys H, White KR, Forsman I. Evaluation of the universal newborn hearing screening and intervention program. Pediatrics. 2010;126 Suppl 1:S19–27. doi:10.1542/peds.2010-0354F.

148. Recommendations for management of common childhood conditions. Evidence for technical update of pocket book recommendations. Geneva: World Health Organization; 2012.

149. Khurshid F, Rao SPN, Sauve C, Gupta S. Universal screening for hyperbilirubinemia in term healthy newborns at discharge: a systematic review and meta-analysis (submitted).

150. Wainer S, Parmar SM, Allegro D, Rabi Y, Lyon ME. Impact of a transcutaneous bilirubinometry program on resource utilization and severe hyperbilirubinemia. Pediatrics. 2012;129(1):77–86. doi:10.1542/peds.2011-0599.

151. Olusanya BO, Imosemi DO, Emokpae AA. Differences between transcutaneous and serum bilirubin measurements in Black African neonates. Pediatrics. 2016;138(3). doi:10.1542/peds.2016-0907.

152. Taylor JA, Burgos AE, Flaherman V, Chung EK, Simpson EA, Goyal NK, et al. Discrepancies between transcutaneous and serum bilirubin measurements. Pediatrics. 2015;135(2):224–31. doi:10.1542/peds.2014-1919.

153. Bhutani VK, Gourley GR, Adler S, Kreamer B, Dalin C, Johnson LH. Noninvasive measurement of total serum bilirubin in a multiracial predischarge newborn population to assess the risk of severe hyperbilirubinemia. Pediatrics. 2000;106(2):E17. doi:10.1542/peds.106.2.e17.

154. McClean S, Baerg K, Smith-Fehr J, Szafron M. Cost savings with transcutaneous screening versus total serum bilirubin measurement for newborn jaundice in hospital and community settings: a cost-minimization analysis. CMAJ Open. 2018;6(3):E285–91. doi:10.9778/cmajo.20170158.

155. Olusanya BO, Ogunlesi TA, Slusher TM. Why is kernicterus still a major cause of death and disability in low-income and middle-income countries? Arch Dis Child. 2014;99(12):1117–21. doi:10.1136/archdischild-2013-305506.

156. Lipstein EA, Nabi E, Perrin JM, Luff D, Browning MF, Kuhlthau KA. Parents' decision-making in newborn screening: opinions, choices, and information needs. Pediatrics. 2010;126(4):696–704. doi:10.1542/peds.2010-0217.

157. Maisels MJ. Neonatal transcutaneous bilirubin measurements: an opportunity to enhance laboratory utilization and improve patient care. Clin Chem. 2012;58(10):1395-6. doi:10.1373/clinchem.2012.183616.

158. Xie B, da Silva O, Zaric G. Cost-effectiveness analysis of a system-based approach for managing neonatal jaundice and preventing kernicterus in Ontario. Paediatr Child Health. 2012;17(1):11–16. doi:10.1093/pch/17.1.11.

159. Lunze K, Bloom DE, Jamison DT, Hamer DH. The global burden of neonatal hypothermia: systematic review of a major challenge for newborn survival. BMC Med. 2013;11(1):24. Doi:10.1186/1741-7015-11-24.

160. Kelly PA, Classen KA, Crandall CG, Crenshaw JT, Schaefer SA, Wade DA, et al. Effect of timing of the first bath on a healthy newborn's temperature. J Obstet Gynecol Neonatal Nurs. 2018;47(5):608–19. doi:10.1016/j.jogn.2018.07.004.

161. Ruschel LM, Pedrini DB, da Cunha MLC. Hypothermia and the newborn's bath in the first hours of life. Rev Gaucha Enferm. 2018;39:e20170263. doi:10.1590/1983-1447.2018.20170263.

162. Simpson EL, Chalmers JR, Hanifin JM, Thomas KS, Cork MJ, McLean WH, et al. Emollient enhancement of the skin barrier from birth offers effective atopic dermatitis prevention. J Allergy Clin Immunol. 2014;134(4):818–23. doi:10.1016/j.jaci.2014.08.005.

163. Dyer JA. Newborn skin care. Semin Perinatol. 2013;37(1):3–7. doi:10.1053/j.semperi.2012.11.008.

164. Lawn JE, Blencowe H, Oza S, You D, Lee AC, Waiswa P, et al. Every newborn: progress, priorities, and potential beyond survival. Lancet. 2014;384(9938):189–205. doi:10.1016/s0140-6736(14)60496-7.

165. Imdad A, Bautista RMM, Senen KAA, Uy MEV, Mantaring Iii JB, Bhutta ZA. Umbilical cord antiseptics for preventing sepsis and death among newborns. Cochrane Database Syst Rev. 2013;(5):CD008635.

166. Coffey PS, Brown SC. Umbilical cord-care practices in low- and middle-income countries: a systematic review. BMC Pregnancy Childbirth. 2017;17(1):68. doi:10.1186/s12884-017-1250-7.

167. Krous HF, Beckwith JB, Byard RW, Rognum TO, Bajanowski T, Corey T, et al. Sudden infant death syndrome and unclassified sudden infant deaths: a definitional and diagnostic approach. Pediatrics. 2004;114(1):234–8. doi:10.1542/peds.114.1.234.

168. Priyadarshi M, Balachander B, Sankar MJ. Effect of sleep position in term healthy newborns on neonatal mortality and sudden infant death syndrome (SIDS): a systematic review (submitted).

169. Filiano JJ, Kinney HC. A perspective on neuropathologic findings in victims of the sudden infant death syndrome: the triple-risk model. Biol Neonate. 1994;65(3-4):194–7. doi:10.1159/000244052.

170. Priyadarshi M, Balachander B, Gupta S, Sankar MJ. Timing of bathing in term healthy newborns: a systematic review (submitted).

171. Callaghan-Koru JA, Nonyane BAS, Guenther T, Sitrin D, Ligowe R, Chimbalanga E, et al. Contribution of community-based newborn health promotion to reducing inequities in healthy newborn care practices and knowledge: evidence of improvement from a three-district pilot program in Malawi. BMC Public Health. 2013;13(1):1052. doi:10.1186/1471-2458-13-1052.

172. Nonyane BA, KC A, Callaghan-Koru JA, Guenther T, Sitrin D, Syed U, et al. Equity improvements in maternal and newborn care indicators: results from the Bardiya district of Nepal. Health Policy Plan. 2015;31(4):405–14. doi:10.1093/heapol/czv077.

173. Chamberlain J, McCarty S, Sorce J, Leesman B, Schmidt S, Meyrick E, et al. Impact on delayed newborn bathing on exclusive breastfeeding rates, glucose and temperature stability, and weight loss. J Neonatal Nurs. 2019;25:74–7. doi:10.1016/j.jnn.2018.11.001.

174. Lund C. Bathing and beyond: current bathing controversies for newborn infants. Adv Neonatal Care. 2016;16 Suppl 5S:S13–20. doi:10.1097/anc.0000000000000336.

175. Priyadarshi M, Balachander B, Gupta S, Sankar MJ. Emollients application in term healthy newborns: a systematic review (submitted).

176. Chalmers JR, Haines RH, Mitchell EJ, Thomas KS, Brown SJ, Ridd M, et al. Effectiveness and cost-effectiveness of daily all-over-body application of emollient during the first year of life for preventing atopic eczema in high-risk children (The BEEP trial): protocol for a randomised controlled trial. Trials. 2017;18(1):343. doi:10.1186/s13063-017-2031-3.

177. Chlorhexidine Umbilical Review Group. Efficacy and safety of umbilical cord cleansing with chlorhexidine in neonates – an individual participant data (IPD) meta-analysis (in preparation).

178. Information Exchange System. Alert No. 133. Chlorhexidine 7,1% digluconate (CHX) aqueous solution or gel (10ml) – reports of serious eye injury due to errors in administration. Geneva: World Health Organization; 2019.

179. Coffey PS, Metzler M, Islam Z, Koehlmoos TP. Willingness to pay for a 4% chlorhexidine (7.1% chlorhexidine digluconate) product for umbilical cord care in rural Bangladesh: a contingency valuation study. BMC Int Health Hum Rights. 2013;13:44. doi:10.1186/1472-698x-13-44.

180. Arifeen SE, Mullany LC, Shah R, Mannan I, Rahman SM, Talukder MR, et al. The effect of cord cleansing with chlorhexidine on neonatal mortality in rural Bangladesh: a community-based, cluster-randomised trial. Lancet. 2012;379(9820):1022–8. doi:10.1016/s0140-6736(11)61848-5.

181. World Health Organization Model List of Essential Medicines for Children, 7th list. Geneva: World Health Organization; 2019.

182. Willinger M, Hoffman HJ, Hartford RB. Infant sleep position and risk for sudden infant death syndrome: report of meeting held January 13 and 14, 1994, National Institutes of Health, Bethesda, MD. Pediatrics. 1994;93(5):814–9.

183. Cheng TL, Monteiro N, DiMeglio LA, Chien AT, Peeples ES, Raetz E, et al. Seven great achievements in pediatric research in the past 40 y. Pediatr Res. 2016;80(3):330–7. doi:10.1038/pr.2016.95.

184. Task Force on Sudden Infant Death Syndrome, Moon RY. SIDS and other sleep-related infant deaths: expansion of recommendations for a safe infant sleeping environment. Pediatrics. 2011;128(5):e1341–67. doi:10.1542/peds.2011-2285.

185. Katz D, Shore S, Bandle B, Niermeyer S, Bol KA, Khanna A. Sudden infant death syndrome and residential altitude. Pediatrics. 2015;135(6):e1442–9. doi:10.1542/peds.2014-2697.

186. Spencer N, Logan S. Sudden unexpected death in infancy and socioeconomic status: a systematic review. J Epidemiol Community Health. 2004;58(5):366–73. doi:10.1136/jech.2003.011551.

187. Hwang SS, Smith RA, Barfield WD, Smith VC, McCormick MC, Williams MA. Supine sleep positioning in preterm and term infants after hospital discharge from 2000 to 2011. J Perinatol. 2016;36(9):787–93. doi:10.1038/jp.2016.80.

188. Smylie J, Fell DB, Chalmers B, Sauve R, Royle C, Allan B, et al. Socioeconomic position and factors associated with use of a nonsupine infant sleep position: findings from the Canadian Maternity Experiences Survey. Am J Public Health. 2014;104(3):539–47. doi:10.2105/ajph.2012.301061.

189. Hirai AH, Kortsmit K, Kaplan L, Reiney E, Warner L, Parks SE, et al. Prevalence and factors associated with safe infant sleep practices. Pediatrics. 2019;144(5). doi:10.1542/peds.2019-1286.

190. Zachry AH, Kitzmann KM. Disparities in sleep position awareness and compliance. South Med J. 2010;103(4):311–5. doi:10.1097/SMJ.0b013e3181d3ce78.

191. Pease A, Garstang JJ, Ellis C, Watson D, Ingram J, Cabral C, et al. Decision-making for the infant sleep environment among families with children considered to be at risk of sudden unexpected death in infancy: a systematic review and qualitative metasynthesis. BMJ Paediatr Open. 2021;5(1):e000983. doi:10.1136/bmjpo-2020-000983.

192. World Health Organization. Hepatitis B vaccines: WHO position paper. Wkly Epidemiol Rec. 2017;27(92):369–92.

193. World Health Organization. Polio vaccines: WHO position paper. Wkly Epidemiol Rec. 2016;12(91):145–68.

194. World Health Organization. BCG vaccines: WHO position paper. Wkly Epidemiol Rec. 2018;8(93):73–96.

195. WHO recommendations for routine immunization – summary tables [website]. Geneva: World Health Organization; 2021 (https://www.who.int/teams/immunization-vaccines-and-biologicals/policies/who-recommendations-for-routine-immunization---summary-tables, accessed 20 January 2022).

196. World Health Organization (WHO), Food and Agriculture Organization of the United Nations. Vitamin and mineral requirements in human nutrition, second edition. Geneva: WHO; 2004.

197. Haider BA, Sharma R, Bhutta ZA. Neonatal vitamin A supplementation for the prevention of mortality and morbidity in term neonates in low and middle income countries. Cochrane Database Syst Rev. 2017;(2):CD006980.

198. Global prevalence of vitamin A deficiency in populations at risk 1995–2005. WHO global database on vitamin A deficiency. Geneva: World Health Organization; 2009.

199. Stevens GA, Bennett JE, Hennocq Q, Lu Y, De-Regil LM, Rogers L, et al. Trends and mortality effects of vitamin A deficiency in children in 138 low-income and middle-income countries between 1991 and 2013: a pooled analysis of population-based surveys. Lancet Glob Health. 2015;3(9):e528–36. doi:10.1016/S2214-109X(15)00039-X.

200. Koo W, Walyat N. Vitamin D and skeletal growth and development. Curr Osteoporos Rep. 2013;11(3):188–93. doi:10.1007/s11914-013-0156-1.

201. Winzenberg T, Jones G. Vitamin D and bone health in childhood and adolescence. Calcif Tissue Int. 2013;92(2):140–50. doi:10.1007/s00223-012-9615-4.

202. Nutritional rickets: a review of disease burden, causes, diagnosis, prevention and treatment. Geneva: World Health Organization; 2019.

203. Við Streym S, Højskov CS, Møller UK, Heickendorff L, Vestergaard P, Mosekilde L, et al. Vitamin D content in human breast milk: a 9-mo follow-up study. Am J Clin Nutr. 2016;103(1):107–14. doi:10.3945/ajcn.115.115105.

204. Imdad A, Rehman F, Davis E, Ranjit D, Surin GSS, Attia SL, et al. Effects of neonatal nutrition interventions on neonatal mortality and child health and development outcomes: a systematic review. Campbell Syst Rev. 2019;17(1):e1141. doi:10.1002/cl2.1021.

205. Neonatal Vitamin A Supplementation Evidence group. Early neonatal vitamin A supplementation and infant mortality: an individual participant data meta-analysis of randomised controlled trials. Arch Dis Child. 2019;104(3):217–26. doi:10.1136/archdischild-2018-315242.

206. Humphrey JH, Agoestina T, Juliana A, Septiana S, Widjaja H, Cerreto MC, et al. Neonatal vitamin A supplementation: effect on development and growth at 3 y of age. Am J Clin Nutr. 1998;68(1):109–17. doi:10.1093/ajcn/68.1.109.

207. Klemm RD, Labrique AB, Christian P, Rashid M, Shamim AA, Katz J, et al. Newborn vitamin A supplementation reduced infant mortality in rural Bangladesh. Pediatrics. 2008;122(1):e242–50. doi:10.1542/peds.2007-3448.

208. Edmond KM, Newton S, Shannon C, O'Leary M, Hurt L, Thomas G, et al. Effect of early neonatal vitamin A supplementation on mortality during infancy in Ghana (Neovita): a randomised, double-blind, placebo-controlled trial. Lancet. 2015;385(9975):1315–23. doi:10.1016/s0140-6736(14)60880-1.

209. Masanja H, Smith ER, Muhihi A, Briegleb C, Mshamu S, Ruben J, et al. Effect of neonatal vitamin A supplementation on mortality in infants in Tanzania (Neovita): a randomised, double-blind, placebo-controlled trial. Lancet. 2015;385(9975):1324–32. doi:10.1016/s0140-6736(14)61731-1.

210. Mazumder S, Taneja S, Bhatia K, Yoshida S, Kaur J, Dube B, et al. Efficacy of early neonatal supplementation with vitamin A to reduce mortality in infancy in Haryana, India (Neovita): a randomised, double-blind, placebo-controlled trial. Lancet. 2015;385(9975):1333–42. doi:10.1016/s0140-6736(14)60891-6.

211. Ahmad SM, Raqib R, Huda MN, Alam MJ, Monirujjaman M, Akhter T, et al. High-dose neonatal vitamin A supplementation transiently decreases thymic function in early infancy. J Nutr. 2020;150(1):176–83. doi:10.1093/jn/nxz193.

212. Klemm RDW, Pokharel RK, Bichha RP, Nargis M, Mahmud Z, Oot L, et al. Testing the feasibility of delivering vitamin A to newborns in Nepal and Bangladesh. Sight and Life. 2011;25:30-7.

213. Codex Alimentarius Commission. Standard on infant formula and formulas for special medical purposes intended for infants. Formerly CAC/RS 72-1972. Adopted as a worldwide standard in 1981. Geneva: World Health Organization; 2007.

214. Tan ML, Abrams SA, Osborn DA. Vitamin D supplementation for term breastfed infants to prevent vitamin D deficiency and improve bone health. Cochrane Database Syst Rev. 2020;(12):CD013046.

215. Optimal feeding of low birth-weight infants in low- and middle-income countries. Geneva: World Health Organization; 2011.

216. Chandy DD, Kare J, Singh SN, Agarwal A, Das V, Singh U, et al. Effect of vitamin D supplementation, directly or via breast milk for term infants, on serum 25 hydroxyvitamin D and related biochemistry, and propensity to infection: a randomised placebo-controlled trial. Br J Nutr. 2016;116(1):52–8. doi:10.1017/s0007114516001756.

217. Floreskul V, Juma FZ, Daniel AB, Zamir I, Rawdin A, Stevenson M, et al. Cost-effectiveness of vitamin D supplementation in pregnant woman and young children in preventing rickets: a modeling study. Front Public Health. 2020;8(439). doi:10.3389/fpubh.2020.00439.

218. Roth DE, Abrams SA, Aloia J, Bergeron G, Bourassa MW, Brown KH, et al. Global prevalence and disease burden of vitamin D deficiency: a roadmap for action in low- and middle-income countries. Ann N Y Acad Sci. 2018;1430(1):44–79. doi:10.1111/nyas.13968.

219. Wagner CL, Greer FR. Prevention of rickets and vitamin D deficiency in infants, children, and adolescents. Pediatrics. 2008;122(5):1142–52. doi:10.1542/peds.2008-1862.

220. Simon AE, Ahrens KA. Adherence to vitamin D intake guidelines in the United States. Pediatrics. 2020;145(6):e20193574. doi:10.1542/peds.2019-3574.

221. Umaretiya PJ, Oberhelman SS, Cozine EW, Maxson JA, Quigg SM, Thacher TD. Maternal preferences for vitamin D supplementation in breastfed infants. Ann Fam Med. 2017;15(1):68–70. doi:10.1370/afm.2016.

222. Taylor JA, Geyer LJ, Feldman KW. Use of supplemental vitamin D among infants breastfed for prolonged periods. Pediatrics. 2010;125(1):105–11. doi:10.1542/peds.2009-1195.

223. Heinig MJ. Vitamin D and the breastfed infant: controversies and concerns. J Hum Lact. 2003;19(3):247–9. doi:10.1177/0890334403255420.

224. Vitamin D expert panel meeting final report. Atlanta, GA: Centers for Disease Control; 2001 (https://www.cdc.gov/nccdphp/dnpa/nutrition/pdf/Vitamin_D_Expert_Panel_Meeting.pdf, accessed 20 January 2022).

225. Crocker B, Green TJ, Barr SI, Beckingham B, Bhagat R, Dabrowska B, et al. Very high vitamin D supplementation rates among infants aged 2 months in Vancouver and Richmond, British Columbia, Canada. BMC Public Health. 2011;11(1):905. doi:10.1186/1471-2458-11-905.

226. Gallo S, Jean-Philippe S, Rodd C, Weiler HA. Vitamin D supplementation of Canadian infants: practices of Montreal mothers. Appl Physiol Nutr Metab. 2010;35(3):303–9. doi:10.1139/h10-021.

227. Hemmingway A, Fisher D, Berkery T, Murray DM, Kiely ME. Adherence to the infant vitamin D supplementation policy in Ireland. Eur J Nutr. 2020:1337–45. doi:10.1007/s00394-020-02334-w.

228. Field T. Pediatric massage therapy research: a narrative review. Children (Basel). 2019;6(6). doi:10.3390/children6060078.

229. Kulkarni A, Kaushik JS, Gupta P, Sharma H, Agrawal RK. Massage and touch therapy in neonates: the current evidence. Indian Pediatr. 2010;47:771–6. doi:10.1007/s13312-010-0114-2.

230. Diego MA, Field T. Moderate pressure massage elicits a parasympathetic nervous system response. Int J Neurosci. 2009;119(5):630–8. doi:10.1080/00207450802329605.

231. Schanberg S, Davalos M, Malphurs J. Massage with oil has more positive effects on normal infants. J Prenat Perinat Psychol Health. 1996;11(2):75.

232. Field T. Massage therapy research review. Complement Ther Clin Pract. 2016;24:19–31. doi:10.1016/j.ctcp.2016.04.005.

233. Priyadarshi M, Kumar V, Balachander B, Gupta S, Sankar MJ. Effect of whole-body massage on growth and neurodevelopment in term healthy newborns: a systematic review (submitted).

234. Bennett C, Underdown A, Barlow J. Massage for promoting mental and physical health in typically developing infants under the age of six months. Cochrane Database Syst Rev. 2013;(4):CD005038.

235. Zhang M, Wang L, Wang Y, Tang J. The influence of massage on neonatal hyperbilirubinemia: a meta-analysis of randomized controlled trials. J Matern-Fetal Neonatal Med. 2019;32(18):3109–14. doi:10.1080/14767058.2018.1455183.

236. Gürol A, Polat S. The effects of baby massage on attachment between mother and their infants. Asian Nurs Res. 2012;6(1):35–41. doi:10.1016/j.anr.2012.02.006.

237. Keller MK, Rehm M. A qualitative case study: fathers experiences massaging their infants. J Behav Soc Sci. 2015;2:29–39.

238. Improving early childhood development: WHO guideline. Geneva: World Health Organization; 2020.

239. Acceptable medical reasons for use of breast-milk substitutes. Geneva: World Health Organization, United Nations Children's Fund; 2009.

240. Protecting, promoting and supporting breastfeeding in facilities providing maternity and newborn services: implementing the revised Baby-friendly Hospital Initiative Geneva: World Health Organization, United Nations Children's Fund; 2018.

241. International code of marketing of breast-milk substitutes. Geneva: World Health Organization; 1981.

242. Jones E, Stewart F, Taylor B, Davis PG, Brown SJ. Early postnatal discharge from hospital for healthy mothers and term infants. Cochrane Database Syst Rev. 2021;(6):CD002958.

243. Yonemoto N, Nagai S, Mori R. Schedules for home visits in the early postpartum period. Cochrane Database Syst Rev. 2021;(7):CD009326.

244. Tiruneh GT, Shiferaw CB, Worku A. Effectiveness and cost-effectiveness of home-based postpartum care on neonatal mortality and exclusive breastfeeding practice in low-and-middle-income countries: a systematic review and meta-analysis. BMC Pregnancy Childbirth. 2019;19(1):507. doi:10.1186/s12884-019-2651-6.

245. Bernstein HH, Spino C, Baker A, Slora EJ, Touloukian CL, McCormick MC. Postpartum discharge: do varying perceptions of readiness impact health outcomes? Ambul Pediatr. 2002;2(5):388–95. doi:10.1367/1539-4409(2002)002<0388:pddvpo>2.0.co;2.

246. Dol J, Kohi T, Campbell-Yeo M, Tomblin Murphy G, Aston M, Mselle L. Exploring maternal postnatal newborn care postnatal discharge education in Dar es Salaam, Tanzania: barriers, facilitators and opportunities. Midwifery. 2019;77:137–43. doi:10.1016/j.midw.2019.07.009.

247. Malagon-Maldonado G, Connelly CD, Bush RA. Predictors of readiness for hospital discharge after birth: building evidence for practice. Worldviews Evid Based Nurs. 2017;14(2):118–27. doi:10.1111/wvn.12208.

248. Tokhi M, Comrie-Thomson L, Davis J, Portela A, Chersich M, Luchters S. Involving men to improve maternal and newborn health: a systematic review of the effectiveness of interventions. PLoS One. 2018;13(1):e0191620. doi:10.1371/journal.pone.0191620.

249. José Santos S. MenCare in Latin America: challenging harmful masculine norms and promoting positive changes in men's caregiving – EMERGE Case Study 5. Brighton: Institute of Development Studies, Promundo-US, Sonke Gender Justice; 2015.

250. WHO recommendations on health promotion interventions for maternal and newborn health. Geneva: World Health Organization; 2015.

251. International Initiative for Impact Evaluation (3ie). An evidence map of social, behavioural and community engagement interventions for reproductive, maternal, newborn and child health. Geneva: World Health Organization; 2017.

252. Ruane-McAteer E, Amin A, Hanratty J, Lynn F, Corbijn van Willenswaard K, Reid E, et al. Interventions addressing men, masculinities and gender equality in sexual and reproductive health and rights: an evidence and gap map and systematic review of reviews. BMJ Glob Health. 2019;4(5):e001634. doi:10.1136/bmjgh-2019-001634.

253. Gunn J, Lumley J, Chondros P, Young D. Does an early postnatal check-up improve maternal health: results from a randomised trial in Australian general practice. Br J Obstet Gynaecol. 1998;105(9):991–7. doi:10.1111/j.1471-0528.1998.tb10263.x.

254. Timing of first postnatal contact by midwife. In: Postnatal care NICE guideline NG194. National Institute for Health and Care Excellence; 2021.

255. Dol J, Richardson B, Bonet M, Langlois EV, Parker R, Scott H, et al. Timing of maternal and neonatal mortality and morbidity in healthy women and newborns during the postnatal period: a systematic review protocol. JBI Evid Synth. 2021;19(3):629–43. doi:10.11124/jbies-20-00036.

256. Oza S, Cousens SN, Lawn JE. Estimation of daily risk of neonatal death, including the day of birth, in 186 countries in 2013: a vital-registration and modelling-based study. Lancet Glob Health. 2014;2(11):e635–44. doi:10.1016/s2214-109x(14)70309-2.

257. Oza S, Lawn JE, Hogan DR, Mathers C, Cousens SN. Neonatal cause-of-death estimates for the early and late neonatal periods for 194 countries: 2000–2013. Bull World Health Organ. 2015;93(1):19–28. doi:10.2471/BLT.14.139790.

258. Jones E, Taylor B, MacArthur C, Bradshaw S, Hope L, Cummins C. Early postnatal discharge for infants: a meta-analysis. Pediatrics. 2020;146(3):e20193365. doi:10.1542/peds.2019-3365.

259. Sainz Bueno JA, Romano MR, Teruel RG, Benjumea AG, Palacín AF, González CA, et al. Early discharge from obstetrics-pediatrics at the Hospital de Valme, with domiciliary follow-up. Am J Obstet Gynecol. 2005;193(3):714–26. doi:10.1016/j.ajog.2005.01.015.

260. Petrou S, Boulvain M, Simon J, Maricot P, Borst F, Perneger T, et al. Home-based care after a shortened hospital stay versus hospital-based care postpartum: an economic evaluation. BJOG. 2004;111(8):800–6. doi:10.1111/j.1471-0528.2004.00173.x.

261. Bowers J, Cheyne H. Reducing the length of postnatal hospital stay: implications for cost and quality of care. BMC Health Serv Res. 2016;16(1):16. doi:10.1186/s12913-015-1214-4.

262. Benova L, Owolabi O, Radovich E, Wong KLM, Macleod D, Langlois EV, et al. Provision of postpartum care to women giving birth in health facilities in sub-Saharan Africa: a cross-sectional study using Demographic and Health Survey data from 33 countries. PLoS Med. 2019;16(10):e1002943. doi:10.1371/journal.pmed.1002943.

263. Doctor HV, Radovich E, Benova L. Time trends in facility-based and private-sector childbirth care: analysis of Demographic and Health Surveys from 25 sub-Saharan African countries from 2000 to 2016. J Glob Health. 2019;9(2):020406. doi:10.7189/jogh.09.020406.

264. Montagu D, Sudhinaraset M, Diamond-Smith N, Campbell O, Gabrysch S, Freedman L, et al. Where women go to deliver: understanding the changing landscape of childbirth in Africa and Asia. Health Policy Plan. 2017;32(8):1146–52. doi:10.1093/heapol/czx060.

265. Brooten D, Roncoli M, Finkler S, Arnold L, Cohen A, Mennuti M. A randomized trial of early hospital discharge and home follow-up of women having cesarean birth. Obstet Gynecol. 1994;84(5):832–8.

266. AltuntuĞ K, Ege E. Effects of health education on mothers' readiness for postpartum discharge from hospital, on postpartum complaints, and quality of life. Turkish J Res Devel Nurs. 2013;15(2):45–56.

267. Bryanton J, Beck CT, Montelpare W. Postnatal parental education for optimizing infant general health and parent-infant relationships. Cochrane Database Syst Rev. 2013;(11):CD004068.

268. Rasaily R, Saxena N, Pandey S, Garg BS, Swain S, Iyengar SD, et al. Effect of home-based newborn care on neonatal and infant mortality: a cluster randomised trial in India. BMJ Glob Health. 2020;5(9):e000680. doi:10.1136/bmjgh-2017-000680.

269. LeFevre AE, Shillcutt SD, Waters HR, Haider S, El Arifeen S, Mannan I, et al. Economic evaluation of neonatal care packages in a cluster-randomized controlled trial in Sylhet, Bangladesh. Bull World Health Organ. 2013;91(10):736–45. doi:10.2471/BLT.12.117127.

270. Pitt C, Tawiah T, Soremekun S, ten Asbroek AH, Manu A, Tawiah-Agyemang C, et al. Cost and cost-effectiveness of newborn home visits: findings from the Newhints cluster-randomised controlled trial in rural Ghana. Lancet Glob Health. 2016;4(1):e45–56. doi:10.1016/S2214-109X(15)00207-7.

271. Daviaud E, Owen H, Pitt C, Kerber K, Bianchi Jassir F, Barger D, et al. Overview, methods and results of multi-country community-based maternal and newborn care economic analysis. Health Policy Plan. 2017;32(suppl 1):i6–20. doi:10.1093/heapol/czx055.

272. Barger D, Owen H, Pitt C, Kerber K, Sitrin D, Mayora C, et al. Multi-country analysis of the cost of community health workers kits and commodities for community-based maternal and newborn care. Health Policy Plan. 2017;32(suppl 1):i84–92. doi:10.1093/heapol/czx038.

273. United Nations Children's Fund (UNICEF), World Health Organization, World Bank Group, United Nations Inter-agency Group for Child Mortality Estimation. Levels and trends in child mortality: report 2019. New York (NY): UNICEF; 2019.

274. Chao F, You D, Pedersen J, Hug L, Alkema L. National and regional under-5 mortality rate by economic status for low-income and middle-income countries: a systematic assessment. Lancet Glob Health. 2018;6(5):e535–47. doi:10.1016/S2214-109X(18)30059-7.

275. McPherson R, Hodgins S. Postnatal home visitation: lessons from country programs operating at scale. J Glob Health. 2018;8(1):010422. doi:10.7189/jogh.08.010422.

276. Escobar GJ, Braveman PA, Ackerson L, Odouli R, Coleman-Phox K, Capra AM, et al. A randomized comparison of home visits and hospital-based group follow-up visits after early postpartum discharge. Pediatrics. 2001;108(3):719–27. doi:10.1542/peds.108.3.719.

277. Lieu TA, Braveman PA, Escobar GJ, Fischer AF, Jensvold NG, Capra AM. A randomized comparison of home and clinic follow-up visits after early postpartum hospital discharge. Pediatrics. 2000;105(5):1058–65. doi:10.1542/peds.105.5.1058.

278. WHO recommendations: optimizing health worker roles to improve access to key maternal and newborn health interventions through task shifting. Geneva: World Health Organization; 2012.

279. Guidelines for malaria vector control. Geneva: World Health Organization; 2019.

280. WHO guideline on health workforce development, attraction, recruitment and retention in rural and remote areas. Geneva: World Health Organization; 2021.

281. Baguiya A, Portela A, Moyvisan A, Gerlach N, Gopal P, Sauvé C, et al. Effectiveness of male involvement intervention on maternal and newborn health outcomes (in preparation).

282. Kunene B, Beksinska M, Zondi S, Mthembu N, Mullick S, Ottolenghi E, et al. Involving men in maternity care: South Africa. Washington (DC): Population Council; 2004.

283. WHO recommendations on home-based records for maternal, newborn and child health. Geneva: World Health Organization; 2018.

284. WHO guideline: recommendations on digital interventions for health system strengthening. Geneva: World Health Organization; 2019.

Annex 1: Contributors to the guideline

WHO Steering Group (Geneva, Switzerland)

Rajiv Bahl
Unit Head
Department of Maternal, Newborn, Child and Adolescent and Health and Ageing

Tarun Dua
Programme Manager
Department of Mental Health and Substance Use

Olufemi T. Oladapo
Unit Head
Department of Sexual and Reproductive Health and Research

Fernando Althabe
Medical Officer
Department of Sexual and Reproductive Health and Research

Mercedes Bonet
Medical Officer
Department of Sexual and Reproductive Health and Research

Neerja Chowdhary
Technical Officer
Department of Mental Health and Substance Use

Karen Edmond
Scientist
Department of Maternal, Newborn, Child and Adolescent and Health and Ageing

Anayda Portela
Technical Officer
Department of Maternal, Newborn, Child and Adolescent and Health and Ageing

Shuchita Gupta
Medical Officer
Department of Maternal, Newborn, Child and Adolescent and Health and Ageing

Lisa Rogers
Technical Officer
Department of Nutrition and Food Safety

João Paulo Souza
Consultant
Department of Maternal, Newborn, Child and Adolescent and Health and Ageing

Guideline Development Group

Shabina Ariff
Director of Neonatal Intensive Care Unit
Consultant Pediatrician and Neonatologist
Department of Pediatrics and Child Health
Aga Khan University
Karachi, Pakistan

Abdullah Baqui
Director
International Center for Maternal and Newborn Health
Johns Hopkins Bloomberg School of Public Health
Baltimore, United States of America (USA)

Blami Dao
Technical Director
Western and Central Africa
Jhpiego, an affiliate of Johns Hopkins University
Baltimore, USA

Louise Tina Day
EN-BIRTH Research Manager
London School of Hygiene and Tropical Medicine
London, United Kingdom of Great Britain and Northern Ireland

Abiy Seifu Estifanos
Lecturer
School of Public Health of Addis Ababa University
Addis Ababa, Ethiopia

Duncan Fisher
Co-founder and Editor
Family Included
United Kingdom

Jane Fisher (Chair, Maternal mental health)
Director, Global and Women's Health
Head, Division of Social Sciences
School of Public Health and Preventive Medicine
Monash University
Melbourne, Australia

Zelee Hill
Associate Professor
Institute for Global Health
Faculty of Population Health Sciences
University College
London, United Kingdom

Caroline Homer
Co-Program Director
Maternal, Child and Adolescent Health
Burnet Institute
Melbourne, Australia

Tamar Kabakian-Khasholian
Associate Professor
Department of Health Promotion and Community Health
Faculty of Health Sciences
American University of Beirut
Beirut, Lebanon

Mary Kinney
Researcher
School of Public Health
University of Western Cape
Cape Town, South Africa

Tina Lavender
Professor of Maternal and Newborn Health
Department of Women's and Children's Health
University of Liverpool
Liverpool, United Kingdom

Pisake Lumbiganon
Professor of Obstetrics and Gynaecology
Convenor, Thai Cochrane Network
Faculty of Medicine
Khon Kaen University
Khon Kaen, Thailand

Address Malata
Professor
Malawi University of Science and Technology
Limbe, Malawi

James Neilson (Chair, Maternal health, health systems and health promotion)
Coordinating Editor
Cochrane Pregnancy and Childbirth Group
University of Liverpool
Liverpool, United Kingdom

Ibone Olza
Co-founder, El Parto es Nuestro
Director of European Institute of Perinatal Mental Health
Madrid, Spain

Malvarappu Prakasamma
Professor
Academy for Nursing Studies and Women's Empowerment Research Studies
Telangana, India

Siddarth Ramji (Chair, Newborn health)
Director-Professor of Pediatrics and Neonatology
Department of Neonatology
Maulana Azad Medical College
New Delhi, India

Parminder Suchdev
Associate Director, Emory Global Health Institute
Director, Global Health Office of Pediatrics
Professor, Department of Pediatrics and Hubert Department of Global Health
Emory University
Atlanta, USA

Mark Tomlinson
Professor
Institute for Life Course Health Research
Stellenbosch University
Stellenbosch, South Africa

Haifa Wahabi
Chair of Evidence-based Healthcare and Knowledge Translation
College of Medicine
King Saud University
Riyadh, Saudi Arabia

External Review Group

Rafat Jan
Professor and Associate Dean
Outreach and Policy Unit
School of Nursing and Midwifery
Aga Khan University
Karachi, Pakistan

Silke Mader
Chairwoman of the Executive Board
European Foundation for the Care of Newborn Infants
Munich, Germany

Matthews Mathai
Independent Consultant in International Maternal and Perinatal Health
St John's, Canada

Linda Richter
Professor
DST-NRF Centre of Excellence in Human Development
University of the Witwatersrand
Johannesburg, South Africa

Jane Sandall
Professor of Social Science and Women's Health
Department of Women and Children's Health
King's College London
London, United Kingdom

Steve Wall
Senior Director
Newborn Health
Save the Children US
Washington, DC, USA

Technical Working Group

Edgardo Abalos
Director
Centro Rosarino de Estudios Perinatales
Rosario, Santa Fe, Argentina

Monica Chamillard
Medical Doctor
Centro Rosarino de Estudios Perinatales
Rosario, Santa Fe, Argentina

Virginia Diaz
Medical Doctor
Centro Rosarino de Estudios Perinatales
Rosario, Santa Fe, Argentina

Soo Downe
Professor of Midwifery Studies
University of Lancashire
Preston, United Kingdom

Kenneth Finlayson
Research Associate
School of Community Health and Midwifery
University of Central Lancashire
Preston, United Kingdom

Leanne Jones
Deputy Managing Editor
Cochrane Pregnancy and Childbirth
Liverpool Women's NHS Foundation Trust
Liverpool, United Kingdom

Ani Movsisyan
Consultant
Pettenkofer School of Public Health
Ludwig Maximilian University of Munich
Munich, Germany

Susan Munabi-Babigumira
Researcher
Department of Global Health/Cochrane Effective Practice and Organisation of Care Group Division for Health Services
Norwegian Institute of Public Health
Oslo, Norway

Julia Pascale
Medical Doctor
Centro Rosarino de Estudios Perinatales
Rosario, Santa Fe, Argentina

Yanina Sguassero
Medical Doctor
Centro Rosarino de Estudios Perinatales
Rosario, Santa Fe, Argentina

Aleena Wojcieszek
Consultant
Dr Aleena Wojcieszek Consulting
Brisbane, Australia

External Partners and Meeting Observers

Carlos Fuchtner
President
International Federation of Gynecology and Obstetrics
London, United Kingdom

Gagan Gupta
Health Specialist
United Nations Children's Fund (UNICEF)
New York, USA

Tedbabe Degefie Hailegebriel
Senior Adviser
Maternal and Newborn Health
UNICEF
New York, USA

Petra ten Hoope-Bender
Technical Adviser
Sexual and Reproductive Health
United Nations Population Fund (UNFPA)
Geneva, Switzerland

William Keenan
Professor, Department of Pediatrics at Saint Louis University
President, International Pediatric Association
Saint Louis, USA

Smita Kumar
Senior Newborn Advisor
Office of Maternal Child Health and Nutrition
United States Agency for International Development (USAID)
Washington, DC, USA

Rebecca Levine
Senior Maternal Health Advisor
USAID
Washington, DC, USA

Jeffrey Smith
Deputy Director
Implementation Research and Demonstration for Scale
Bill & Melinda Gates Foundation
Washington, DC, USA

Ann Yates
Lead Midwife Advisor
International Confederation of Midwives
The Hague, The Netherlands

Willibald Zeck
Global Maternal and Newborn Thematic Fund Coordinator
UNFPA
New York, USA

WHO Regional Office Representatives

Regional Office for Africa

Triphonie Nkurunziza
Medical Officer
Reproductive, Maternal Health and Ageing Team

Assumpta Muriithi
Medical Officer
Reproductive, Maternal Health and Ageing Team
Child and Adolescent Health Programme

Regional Office for the Americas/Pan American Health Organization

Bremen De Mucio
Advisor on Sexual and Reproductive Health
Latin American Center of Perinatology, Women and Reproductive Health

Pablo Duran
Advisor, Perinatal Health
Latin American Center of Perinatology
Women and Reproductive Health

Regional Office for Europe

Oleg Kuzmenko
Technical Officer
Reproductive and Maternal Health

Regional Office for the Eastern Mediterranean

Jamela Al-Raiby
Regional Advisor
Child and Adolescent Health Programme

Karima Gholbzouri
Regional Advisor
Sexual and Reproductive Health and Research

Regional Office for South-East Asia

Anoma Jayathilaka
Medical Officer
Maternal and Reproductive Health

Annex 2: Summary of declarations of interests from the Guideline Development Group (GDG) members and how they were managed

Name	Expertise contributed to the guideline development	Declared interest(s)	Management of conflict(s) of interest
Shabina Ariff	Neonatology, content expert and end-user	None declared	Not applicable
Abdullah Baqui	Neonatology, content expert and end-user	Research grant from WHO to conduct trial to assess impact of simpler antibiotics regimen for young infant infections (US$ 500 215)<br>Director, International Center for Maternal and Newborn Health at John Hopkins University, and in this capacity advocates for postnatal care	This declared conflict of interest was not considered significant enough to pose any risk to the guideline development process or to reduce its credibility
Blami Dao	Obstetrics, content expert and end-user	None declared	Not applicable
Louise Tina Day	Obstetrics, neonatology, content expert and end-user	London School of Hygiene and Tropical Medicine – salary support for EN-BIRTH study<br>Bill & Melinda Gates Foundation – travel and participation costs for meetings in December 2019 and February 2020 (US$ 1000)	This declared conflict of interest was not considered significant enough to pose any risk to the guideline development process or to reduce its credibility
Abiy Seifu Estifanos	Neonatology, content expert and end-user	None declared	Not applicable
Duncan Fisher	Fathers' and parents' group representative	Fees for speaking at GOLD Learning conferences, ceased in April 2020 (US$ 750 x 2)	This declared conflict of interest was not considered significant enough to pose any risk to the guideline development process or to reduce its credibility
Jane Fisher	Mental health, content expert and end-user	Research grants in the field of perinatal mental health, early parenting and the promotion of early childhood development to: the Global and Women's Health Unit at Monash University, Ramsay Hospital Research Foundation, Australian Department of Health, Australian National Health and Medical Research Council, Victorian Department of Education and Training, Grand Challenges Canada, the World Bank, Sexual Violence Research Initiative, and National Health and Medical Research Council (total approximately A$ 3 million, including Can$ 500 000)<br>Immediate Past President of the International Marcé Society (2018–2020)	This declared conflict of interest was not considered significant enough to pose any risk to the guideline development process or to reduce its credibility
Zelee Hill	Health promotion, content expert and end-user	None declared	Not applicable
Caroline Homer	Midwifery, content expert and end-user	None declared	Not applicable

Name	Expertise contributed to the guideline development	Declared interest(s)	Management of conflict(s) of interest
Tamar Kabakian-Khasholian	Health promotion, content expert and end-user	None declared	Not applicable
Mary Kinney	Health promotion, content expert and end-user	None declared	Not applicable
Tina Lavender	Midwifery, content expert and end-user	Paid an honorarium for chairing symposium on newborn skin care at International Confederation of Midwives' conference (£5000)<br>Received National Institute for Health Research grant funding at University of Liverpool for research on prevention and management of stillbirth (approximately £2.5 million)<br>Paid consultancy for research steering group funded by Lunaler Group (£7000)	This declared conflict of interest was not considered significant enough to pose any risk to the guideline development process or to reduce its credibility
Pisake Lumbiganon	Obstetrics, content expert and end-user	None declared	Not applicable
Address Malata	Midwifery, content expert and end-user	None declared	Not applicable
James Neilson	Obstetrics, content expert and end-user	None declared	Not applicable
Ibone Olza	Women representative, mental health	Teaching and supervising students at European Institute of Perinatal Mental Health (€300 per class, around 10 classes per month)<br>Fees for speaking at conferences (less than €1000 per year)<br>Activist for El Parto es Nuestro, NGO for women's rights in childbirth, and elected board member of the International Marcé Society<br>COST Action Devotion research on birth trauma and perinatal mental health	This declared conflict of interest was not considered significant enough to pose any risk to the guideline development process or to reduce its credibility
Malvarappu Prakasamma	Nursing, content expert and end-user	None declared	Not applicable
Siddarth Ramji	Neonatology, content expert and end-user	None declared	Not applicable
Parminder Suchdev	Nutrition, content expert and end-user	Research and salary support from Centers for Disease Control and Prevention for monitoring and evaluating nutritional interventions (20% of salary)	This declared conflict of interest was not considered significant enough to pose any risk to the guideline development process or to reduce its credibility
Mark Tomlinson	Health promotion, content expert and end-user	None declared	Not applicable
Hayfaa Wahabi	Obstetrics, content expert and end-user	None declared	Not applicable

Annex 3: Template "summary of judgements" table for evidence-to-decision domains

**Desirable effects**	Don't know	Varies		Trivial	Small	Moderate	Large
**Undesirable effects**	Don't know	Varies		Large	Moderate	Small	Trivial
**Certainty of the evidence**	No included studies			Very low	Low	Moderate	High
**Values**				Important uncertainty or variability	Possibly important uncertainty or variability	Probably no important uncertainty or variability	No important uncertainty or variability
**Balance of effects**	Don't know	Varies	Favours control	Probably favours control	Does not favour either	Probably favours intervention	Favours intervention
**Resources required**	Don't know	Varies	Large costs	Moderate costs	Negligible costs or savings	Moderate savings	Large savings
**Certainty of the evidence on required resources**	No included studies			Very low	Low	Moderate	High
**Cost-effectiveness**	Don't know	Varies	Favours control	Probably favours control	Does not favour either	Probably favours intervention	Favours intervention
**Equity**	Don't know	Varies	Reduced	Probably reduced	Probably no impact	Probably increased	Increased
**Acceptability**	Don't know	Varies		No	Probably no	Probably yes	Yes
**Feasibility**	Don't know	Varies		No	Probably no	Probably yes	Yes

For more information, please contact:

World Health Organization
Avenue Appia 20
CH-1211 Geneva 27
Switzerland

Department of Sexual and Reproductive Health and Research
Email: srhmph@who.int
Website: www.who.int/teams/sexual-and-reproductive-health-and-research-(srh)

Department of Maternal, Newborn, Child and Adolescent Health and Ageing
Email: mncah@who.int
Website: www.who.int/teams/maternal-newborn-child-adolescent-health-and-ageing/

Department of Mental Health and Substance Use
Email: mhgap-info@who.int
Website: www.who.int/teams/mental-health-and-substance-use

Department of Nutrition and Food Safety
Email: nutrition@who.int
Website: www.who.int/teams/nutrition-and-food-safety/overview

978-92-4-004598-9

World Health Organization | USAID FROM THE AMERICAN PEOPLE | MCHIP Maternal and Child Health Integrated Program | Maternal and Child Survival Program

April 2015 www.mcsprogram.org

Postnatal Care for Mothers and Newborns

Highlights from the World Health Organization 2013 Guidelines

Background

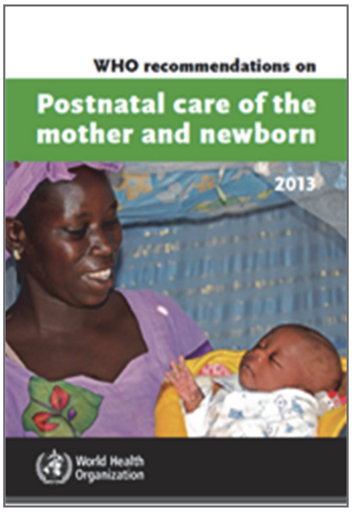

The days and weeks following childbirth—the postnatal period—are a critical phase in the lives of mothers and newborn babies. Most maternal and infant deaths occur in the first month after birth: almost half of postnatal maternal deaths occur within the first 24 hours,[1] and 66% occur during the first week.[2] In 2013, 2.8 million newborns died in their first month of life—1 million of these newborns died on the first day.[3,4]

Considerable progress has been made globally in improving maternal health. Around the world, 72% of women give birth attended by skilled personnel,[5] and the maternal mortality ratio has decreased from 380 to 210 per 100,000 live births between 2000 and 2013. Yet, in South-East Asia and sub-Saharan Africa only 67% and 48% of women give birth with the assistance of skilled personnel, respectively.[5] Postnatal care reaches even fewer women and newborns: less than half of women receive a postnatal care visit within 2 days of childbirth.[4] An analysis of Demographic and Health Survey data from 23 sub-Saharan African countries found that only 13% of women who delivered at home received postnatal care within 2 days of birth.[6]

The World Health Organization (WHO) recently updated global guidelines on postnatal care for mothers and newborns through a technical consultation process. The new guidelines address the timing and content of postnatal care for mothers and newborns with a special focus on resource-limited settings in low- and middle-income countries.[7] They complement other recommendations on maternal, perinatal and newborn health,[8,9] as well as those recommendations on which type of health care worker can safely deliver key maternal and newborn health care interventions,[10] which went through a similar guidelines development process.

Although this brief focuses on postnatal care, the importance of antenatal and intrapartum care within a continuum is recognized to have the greatest impact on maternal and newborn survival.

This brief presents the WHO recommendations while highlighting changes and recommended best practices. It is intended to assist policy-makers, programme managers, educators, and providers involved in caring for women and newborns after birth. Operationalization of these guidelines may help end preventable death, improve health outcomes, strengthen community-based health systems, address gender and equity issues, and emphasize respectful and women-centred maternity care.[11] Strategies to improve quality and achieve equitable use of postnatal care should be selected to maximize population-level results in low-resource settings.

A Unifying Term—Postnatal Care

Because the interchangeable use of the terms "postpartum" referring to issues pertaining to the mother and "postnatal" referring to those concerning the baby creates sometimes confusion, the adoption of just a single term "postnatal" should be used for all issues pertaining to the mother and the baby after birth up to 6 weeks (42 days).

Source: WHO Technical Consultation on Postpartum and Postnatal Care. WHO/MPS/10.03. World Health Organization 2010

Best Practices: Postnatal Care for All Mothers and Newborns

- **Provide postnatal care in the first 24 hours to all mothers and babies—regardless of where the birth occurs.** A full clinical examination should be done around 1 hour after birth, when the baby has had his/her first breastfeed. The baby should be checked again before discharge. For home births, the first postnatal contact should be as early as possible within 24 hours of birth and, if possible, an extra contact for home births at 24–48 hours is desirable. Mobile phone-based postnatal care contacts between mothers and the health

system may be useful. The content of postnatal care is described in the next two sections.

- **Ensure healthy women and their newborns stay at a health facility at least 24 hours and are not discharged early.** This recommendation is an update from 2006, and the minimum duration of stay was lengthened from 12 to 24 hours. Evidence suggests discharge is acceptable only if a mother's bleeding is controlled, mother and baby do not have signs of infection or other diseases, and the baby is breastfeeding well.
- **All mothers and babies need at least four postnatal checkups in the first 6 weeks.** This is a notable change to the previous guidance, which recommended only two postnatal checkups within 2 to 3 days and at 6 weeks after birth. Now, in addition to postnatal care with two full assessments on the first day, three additional visits are recommended: day 3 (48–72 hours), between days 7–14 and 6 weeks after birth. These contacts can be made at home or in a health facility, depending on the context and the provider. Additional contacts may be needed to address issues or concerns.

Postnatal Care Highlights

- Provide postnatal care in first 24 hours for every birth:
  - Delay facility discharge for at least 24 hours.
  - Visit women and babies with home births within the first 24 hours.
- Provide every mother and baby a total of four postnatal visits on:
  - First day (24 hours)
  - Day 3 (48–72 hours)
  - Between days 7–14
  - Six weeks
- Offer home visits by midwives, other skilled providers or well-trained and supervised community health workers (CHWs).
- Use chlorhexidine after home deliveries in high newborn mortality settings.
- Re-emphasize and support elements of quality postnatal care for mother and newborn, including identification of issues and referrals.

Table 1. Provision of Postnatal Care to Mothers and Newborns: Policy and Programme Actions Based On the New WHO Guidelines

WHO Recommendation 2013	Policy/Programme Action
**RECOMMENDATION 1: Timing of discharge from a health facility after birth**	
After an uncomplicated vaginal birth in a health facility, healthy mothers and newborns should receive care in the facility for at least 24 hours after birth.* **(NEW in 2013)**<br>** For the newborn, this care includes an immediate assessment at birth, a full clinical examination around 1 hour after birth and before discharge.*	• Ensure respectful, women-centred quality care[a] is provided for all births.<br>• Review if increased infrastructure (beds, etc.) and staff in postnatal wards are required to provide care respectfully and comfortably for women to stay longer.<br>• Align policies (such as national institutional delivery incentive and insurance schemes) with recommendation.<br>• Adapt and use a simple discharge checklist.[12]
**RECOMMENDATION 2: Number and timing of postnatal contacts**	
If birth is ***in a health facility***, mothers and newborns should receive postnatal care in the facility for at least 24 hours after birth.[a] **(NEW in 2013)**	• Ensure that national standards, quality improvement tools and training curricula promote three assessments in the first 24 hours for the newborn: an immediate assessment at birth; a full clinical examination around 1 hour after birth and again before discharge.<br>• Coordinate postnatal care with the Baby-Friendly Hospital Initiative[13] to ensure that facility-based procedures and outreach to the community support optimal breastfeeding practices.<br>• Update facility-based providers and promote best practices in postnatal care including pre-discharge counselling, according to the new guidelines.
If birth is ***at home***, the first postnatal contact should be as early as possible within 24 hours of birth. **(NEW in 2013)**	• Review current policies and programmes to strengthen delivery and early postnatal care for home births by midwives, other skilled providers and/or well-trained, supervised CHWs.
At least three additional postnatal contacts are recommended for all mothers and newborns, on day 3 (48–72 hours), between days 7–14, and 6 weeks after birth. **(NEW in 2013)**	• Ensure national standards, quality improvement tools, training curricula and behaviour change communication (BCC) messages/materials to explicitly promote the three additional postnatal care checkups (a total of four from birth in the first 6 weeks) through home visits and facility-based care.<br>• Review/revise national monitoring systems to include the process indicator for postnatal care visits—number of mothers/newborns who received postnatal within 2 days of childbirth (regardless of place of delivery)—for all births.

WHO Recommendation 2013	Policy/Programme Action
**RECOMMENDATION 3: Home visits for postnatal care**	
Home visits in the first week after birth are recommended for care of the mother and newborn.	• Determine how best to integrate home visits for postnatal care into responsibilities and training of midwives, other skilled providers and/or well-trained, supervised CHWs.<br>• Explore appropriate mHealth strategies to communicate with mothers who may be difficult to physically reach.
[a] WHO guidelines, *Pregnancy, childbirth, postpartum and newborn care: A guide for essential practice,* define this standard of care; they can be found at http://www.who.int/maternal_child_adolescent/documents/924159084x/en/.	

Related Highlights from Other WHO Guidelines

- Encourage women to deliver with a skilled birth attendant at a health facility so they receive quality intrapartum and postnatal care including administration of a uterotonic during the third stage of labour. Professional skilled care is important for all women and newborns during labour, childbirth and the first day after birth.
- Promote respectful and women-centred maternity care where women are treated with kindness, dignity and respect. Respectful maternity care is an essential part of postnatal care particularly in health facilities. It promotes best practices (such as rooming in, unless separation is medically necessary), recognizes that women and their families should be fully informed on all aspects of care, and values counselling as an opportunity to answer questions and address concerns.

Best Practices: Postnatal Care for Newborns

- **Strengthen postnatal care through home visits and at health facilities.** Elements of postnatal care are re-emphasized from the 2006 guidelines without many significant changes.
- **At each of the four postnatal care checkups, newborns should be assessed for key clinical signs of severe illness and referred as needed.** Nine clinical signs (listed in Recommendation 4 in Table 2 below) have been identified as danger signs that can be identified at home by a CHW or by a skilled provider in a health facility. Evidence suggests that simple algorithms are valid tools in both settings.
- **Continue to promote early and exclusive breastfeeding (EBF) within delivery settings including antenatal care, at delivery, and in all postnatal care visits.** Consistent with previous WHO guidelines, evidence shows EBF reduces the risks of mortality and morbidity in the first month of life (compared to partial and predominant breastfeeding) and improves post-neonatal outcomes. It also encourages improved birth spacing by delaying the return to fecundity. Given the increases in institutional deliveries in many developing countries, policies and programmes should actively promote facility-based counselling and support for EBF including counselling on common breastfeeding problems and ways to manage them if they occur.
- **Consider the use of chlorhexidine for umbilical cord care for babies born at home** to reduce newborn mortality. For newborns who are born at home in settings with high neonatal mortality (30 or more neonatal deaths per 1,000 live births) it is recommended to apply chlorhexidine (7.1% chlorhexidine digluconate aqueous solution or gel, delivering 4% chlorhexidine) daily to the umbilical cord stump during the first week of life. This is a new recommendation, and clean, dry cord care remains the standard recommendation for newborns born in health facilities and at home in low neonatal mortality settings. The use of chlorhexidine in these situations may be considered only to replace application of a harmful traditional substance, such as cow dung, to the cord stump.
- **Reinforce key newborn care messages among families and providers.** WHO re-emphasizes key elements of newborn care including delayed bathing, skin-to-skin contact and immunization. Given the vulnerability of preterm and low-birth-weight babies, interventions are needed to identify these newborns in home and facility settings and ensure they receive special care.

Table 2. Postnatal Care for Newborns: Policy and Programme Actions Based On the New WHO Guidelines

WHO Recommendation 2013	Policy/Programme Action
**RECOMMENDATION 4: Assessment of the baby**	
The following signs should be assessed during each postnatal care contact, and the newborn should be referred for further evaluation if any of the signs is present: *stopped feeding well, history of convulsions, fast breathing (breathing rate of ≥60 per minute), severe chest in-drawing, no spontaneous movement, fever (temperature ≥37.5 °C), low body temperature (temperature <35.5 °C), any jaundice in first 24 hours of life, or yellow palms and soles at any age.*<br>The family should be encouraged to seek health care early if they identify any of the above danger signs in-between postnatal care visits.	• Review and adapt available community-based and facility-based job aids for clinical assessments (such as integrated management of childhood illness, integrated management of pregnancy and childbirth) based on simple clinical signs of severe newborn illnesses.<br>• Integrate recognition of clinical signs into CHW and skilled provider trainings.<br>• Review/revise educational messages to emphasize newborn danger signs and care-seeking in counselling of pregnant and postnatal women, families and communities.
**RECOMMENDATION 5: Exclusive breastfeeding (EBF)**	
All babies should be exclusively breastfed from birth until 6 months of age. Mothers should be counselled and provided support for EBF at each postnatal contact.	• Reinforce early EBF and EBF messages during pregnancy and during all postnatal care visits.<br>• Ensure breastfeeding is actively promoted in all health facilities.<br>• Identify and address problems that prevent EBF (e.g., not initiating breastfeeding within 1 hour after birth, not giving colostrum, giving pre-lacteal feeds, breast health issues, mothers' perceptions that their breast milk is not sufficient, lack of knowledge about breastfeeding frequently and from both breasts to ensure breast milk supply).<br>• Integrate lactational amenorrhoea method (LAM) and EBF messages to ensure LAM criteria are followed and the major barriers to EBF are addressed that threaten the effectiveness of LAM.<br>• Prepare mothers for transitioning their infants to complementary foods with continued breastfeeding at 6 months and modern family planning methods for mothers using LAM.
**RECOMMENDATION 6: Cord care**	
Daily chlorhexidine (7.1% chlorhexidine digluconate aqueous solution or gel, delivering 4% chlorhexidine) application to the umbilical cord stump during the first week of life is recommended for newborns who are born at home in settings with high neonatal mortality (30 or more neonatal deaths per 1,000 live births). **(NEW in 2013)**<br>Clean, dry cord care is recommended for newborns born in health facilities and at home in low neonatal mortality settings. Use of chlorhexidine in these situations may be considered only to replace application of a harmful traditional substance, such as cow dung, to the cord stump.	• In settings with high neonatal mortality, ensure chlorhexidine is available for home births for immediate use by mothers. Related policy/programme issues may include: inclusion on the national List of Essential Medicines for Children; drug registration; local production or procurement; training; supply chain maintenance; and training community midwives and health workers, etc.<br>• Reinforce community messages about clean, dry cord care and add chlorhexidine messages, as appropriate.
**RECOMMENDATION 7: Other postnatal care for the newborn**	
Bathing should be delayed until 24 hours after birth. If this is not possible due to cultural reasons, bathing should be delayed for at least 6 hours. Appropriate clothing of the baby for ambient temperature is recommended. This means one to two layers of clothes more than adults, and use of hats/caps. The mother and baby should not be separated and should stay in the same room 24 hours a day.<br>Communication and play with the newborn should be encouraged. Immunization should be promoted as per existing WHO guidelines.	• Review BCC messages and facility standards to ensure families and providers are informed on these key newborn care messages.<br>• Review national policies and standards with WHO guidelines[14] and revise/strengthen as appropriate.<br>• Encourage skin-to-skin care as part of kangaroo mother care, but also to keep babies warm in cold environments and for all newborns for at least 1 hour after birth.

WHO Recommendation 2013	Policy/Programme Action
Preterm and low-birth-weight babies should be identified as soon as possible and should be provided special care as per existing WHO guidelines.	• Develop approaches to identify and refer preterm and low- birth-weight babies, appropriate for home and facility births.<br>• Review clinical standards to promote special care, such as feeding of low-birth-weight infants [15] and kangaroo mother care.[11]

POSTNATAL CARE-RELATED RECOMMENDATIONS ON NEWBORN CARE FROM OTHER WHO GUIDELINES

- Immediately at birth, all babies should be dried thoroughly and their breathing assessed. The cord should be clamped and cut only after 1–3 minutes, unless the baby needs resuscitation. Routine suctioning must not be done.
- During the first hour after birth, the baby should be in skin-to-skin contact with the mother for warmth and the initiation of breastfeeding.
- A full clinical examination (including weight, danger signs, eyes, cord) and other preventive care should be done around 1 hour after birth, when the baby has had his/her first breastfeed. This care includes giving vitamin K prophylaxis and hepatitis B vaccination as soon as possible after birth (within 24 hours).
- When skilled health personnel attend the newborn, whether at home or in a facility, additional care should be provided. This care includes basic newborn resuscitation with bag and mask for newborns not breathing spontaneously and full clinical examinations at the recommended times.

Best Practices: Postnatal Care for Mothers

- **Strengthen postnatal care for mothers through home visits and at health facilities.** Elements of care are re-emphasized from the 2006 guidelines without many significant changes. Postnatal care includes counselling on family planning, maternal mental health, nutrition and hygiene, and gender-based violence.

Table 3. Postnatal Care for Mothers: Policy and Programme Actions Based On the New WHO Guidelines

WHO Recommendation 2013	Policy/Programme Action
**RECOMMENDATION 8: Assessment of the mother**	
First 24 hours after birth: All postpartum women should have regular assessment of vaginal bleeding, uterine contraction, fundal height, temperature and heart rate (pulse) routinely during the first 24 hours starting from the first hour after birth. Blood pressure should be measured shortly after birth. If normal, the second blood pressure measurement should be taken within 6 hours. Urine void should be documented within 6 hours.	• Ensure that national standards and training curricula for skilled birth attendants include these elements of postnatal care.<br>• Introduce or re-emphasize standards at the facility level using quality improvement tools and checklists.
Beyond 24 hours after birth:<br>At each subsequent postnatal contact, enquiries should continue to be made about general well-being and assessments made regarding the following: urination and urinary incontinence, bowel function, healing of any perineal wound, headache, fatigue, back pain, perineal pain and perineal hygiene, breast pain, uterine tenderness and lochia.	• Review national standards and trainings for skilled birth attendants and CHWs include these elements of postnatal care.<br>• Introduce or re-emphasize standards at the facility level and for home postnatal care visits using quality improvement tools, job aids, and checklists.
Breastfeeding should be assessed at each postnatal contact.	• Ensure national standards, quality improvement tools and training curricula includes updated breastfeeding policy about managing breastfeeding problems, Baby-Friendly Hospital Initiative principles for facility births, and community outreach.
At each postnatal contact, women should be asked about their emotional wellbeing, what family and social support they have and their usual coping strategies for dealing with day-to-day matters. All women and their families/partners should be encouraged to tell their health care professional about any changes in mood, emotional state and behaviour that are outside of the woman's normal pattern.	• Review/revise national standards, quality improvement tools and training curricula to include emotional wellbeing assessment.

WHO Recommendation 2013	Policy/Programme Action
At 10–14 days after birth, all women should be asked about resolution of mild, transitory postpartum depression ("maternal blues"). If symptoms have not resolved, the woman's psychological well-being should continue to be assessed for postpartum depression, and if symptoms persist, evaluated.	• Review/revise national standards, quality improvement tools and training curricula to include counselling for postpartum depression.<br>• Ensure linkages/referrals to available maternal mental health services for evaluation.
Women should be observed for any risks, signs and symptoms of domestic abuse.	• Ensure linkages/referrals within facilities and at the community level to available gender-based violence services.
Women should be told whom to contact for advice and management.	
All women should be asked about resumption of sexual intercourse and possible dyspareunia as part of an assessment of overall well-being 2–6 weeks after birth.	• Integrate messages on postpartum pregnancy risk and family planning, including LAM and postpartum intrauterine contraceptive device (IUD).<br>• Review national health management information systems tools to construct new feasible postpartum family planning indicator(s) to be tracked and reported (e.g., percentage of postpartum women accepting a contraceptive method prior to discharge [disaggregated by method: LAM, postpartum IUD, postpartum tubal ligation, condoms]; percentage of women bringing children for vaccination who accept a family planning method in the same visit).
If there are any issues of concern at any postnatal contact, the woman should be managed and/or referred according to other specific WHO guidelines.[a]	
**RECOMMENDATION 9: Counselling**	
All women should be given information about the physiological process of recovery after birth and told that some health problems are common, with advice to report any health concerns to a health care professional, in particular, signs and symptoms of postpartum haemorrhage, pre-eclampsia/eclampsia, infection and thromboembolism. **(NEW in 2013)**	• Review/revise national standards, quality improvement tools and training curricula for skilled birth attendants and CHWs to include these elements of postnatal counselling and care, particularly the addition of screening for thromboembolism.<br>• Introduce or re-emphasize standards at the facility level and for home postnatal care visits using quality improvement tools, job aids, and checklists.<br>• Review/revise educational messages to emphasize postnatal danger signs and care seeking in counselling of pregnant and postnatal women, families and communities.
Women should be counselled on nutrition.	• Emphasize with mothers and their family members the importance of eating a greater amount and variety of healthy foods.<br>• Review/revise national standards, quality improvement tools and training curricula for providers to ensure adequate counselling skills on nutrition in the context of local practices and taboos, particularly adolescents and very thin women.<br>• Review/revise CHW training curriculum, CHW job aids and BCC materials to emphasize key postnatal nutrition messages.
Women should be counselled on hygiene, especially handwashing.	• Review/revise CHW training curriculum, CHW job aids and BCC materials to emphasize hygiene and handwashing for postnatal (especially if the woman experienced a severe perineal tear), newborn, and infant care.
Women should be counselled on birth spacing and family planning. Contraceptive options should be discussed, and contraceptive methods should be provided if requested.	• Integrate messages on postpartum pregnancy risk and family planning, including LAM.
Women should be counselled on safer sex including use of condoms.	

WHO Recommendation 2013	Policy/Programme Action
In malaria-endemic areas, mothers and babies should sleep under insecticide-impregnated bed nets.	
All women should be encouraged to mobilize as soon as appropriate following the birth. They should be encouraged to take gentle exercise and make time to rest during the postnatal period.	• Review community messages for family members, such as partners and mothers-in-law, to encourage them to help ensure the woman eats enough and avoids strenuous physical work.
**RECOMMENDATION 10: Iron and folic acid supplementation**	
Iron and folic acid supplementation should be provided for at least 3 months after delivery.[b]	• Review national standards, quality improvement tools, and training curricula on postnatal iron and folic acid supplementation for postnatal mothers.<br>• Strengthen iron and folic acid distribution and compliance among postnatal mothers.<br>• Review/update national standards to remove vitamin A supplementation for postnatal women[16] and transition to promoting dietary sources of vitamin A for postnatal mothers.
**RECOMMENDATION 11: Prophylactic antibiotics**	
The use of antibiotics among women with a vaginal delivery and a third or fourth degree perineal tear is recommended for prevention of wound complications. **(NEW in 2013)**	• Review/revise national standards, quality improvement tools, and training curricula to include use of antibiotics for women with a third or fourth degree perineal tear.
There is insufficient evidence to recommend the routine use of antibiotics in all low-risk women with a vaginal delivery for prevention of endometritis.	
**RECOMMENDATION 12: Psychosocial support**	
Psychosocial support by a trained person is recommended for the prevention of postpartum depression among women at high risk of developing this condition. There is insufficient evidence to recommend routine formal debriefing to all women to reduce the occurrence/risk of postpartum depression or to recommend the routine distribution of, and discussion about, printed educational material for prevention of postpartum depression.	• Review/revise national standards, quality improvement tools, and training curricula on counselling for postpartum depression.<br>• Ensure linkages/referrals to available maternal mental health services for evaluation.
Health professionals should provide an opportunity for women to discuss their birth experience during their hospital stay.	
A woman who has lost her baby should receive additional supportive care.	• Review/revise national standards, quality improvement tools, and training curricula to integrate culturally appropriate services (including counselling) for women experiencing miscarriages, stillbirths, and newborn deaths.

[a] The WHO guidelines, *Pregnancy, childbirth, postpartum and newborn care: A guide for essential practice,* that define this standard of care can be found at http://www.who.int/maternal_child_adolescent/documents/924159084x/en/.
[b] Currently, there is no evidence to change this recommendation. WHO is working on developing specific guidelines for maternal nutrition interventions after birth.

Postnatal Care-Related Recommendations from Other WHO Guidelines

Continue to ensure all women who give birth receive active management of third stage of labour and close monitoring immediately after birth as part of their delivery care that reduces the risk of postpartum haemorrhage in the postnatal period.

[1] Every Newborn, An Executive Summary for *The Lancet*'s Series. May 2014.
[2] Nour N. 2008. An Introduction to Maternal Mortality. *Reviews in Obstetrics & Gynecology*. 1:77–81.
[3] The Inter-agency Group for Child Mortality Estimation (UN IGME). 2014. *Levels & Trends in Child Mortality, Report 2014*. United Nations Children's Fund.
[4] Lawn JE et al. 2014. Every Newborn: Progress, Priorities, and Potential Beyond Survival. *Lancet* 384:189–205.
[5] WHO. 2014. *World Health Statistics 2014*. Geneva: WHO.
[6] Warren C, Daly P, Toure L, and Mongi P. 2006. Postnatal Care. Pp. 79–90 in *Opportunities for Africa's Newborns: Practical Data Policy and Programmatic Support for Newborn Care in Africa*, edited by J. Lawn and K. Kerber. Cape Town, South Africa: Partnership for Maternal, Newborn and Child Health.
[7] WHO. *WHO Recommendations on Postnatal Care of the Mother and Newborn*. October 2013. Geneva: WHO.
[8] WHO. 2013. *Recommendations on Maternal and Perinatal Health.* Geneva: WHO. Guidelines on maternal, newborn, child and adolescent health approved by the WHO guidelines review committee.
[9] Ibid.
[10] WHO recommends optimizing health worker roles to improve access to key maternal and newborn health interventions through task shifting (WHO, 2012).
[11] For information and resources on respectful maternity care, see http://www.k4health.org/toolkits/rmc.
[12] WHO guidelines recommend a safe childbirth checklist such as http://www.plosone.org/article/info%3Adoi%2F10.137%2Fjournal.pone.0035151#s5.
[13] http://www.who.int/nutrition/topics/bfhi/en/.
[14]http://www.who.int/reproductivehealth/publications/maternal_perinatal_health/924159084X/en/index.html.
[15] WHO. 2011. Guidelines on Optimal Feeding of Low Birth-weight Infants in Low- and Middle-income Countries.
[16] Vitamin A supplementation in postpartum women is not included in these guidelines. More information can be found at http://whqlibdoc.who.int/publications/2011/9789241501774_eng.pdf.

This brief was made possible by the generous support of the American people through the United States Agency for International Development (USAID) under the terms of the Leader with Associates Cooperative Agreement GHS-A-00-08-00002-00 and the Cooperative Agreement AID-OAA-A-14-00028. All reasonable precautions have been taken by the World Health Organization and USAID to verify the information contained in this publication. However, the published material is being distributed without warranty of any kind, either expressed or implied. The responsibility for the interpretation and use of the material lies with the reader. In no event shall the World Health Organization be liable for damages arising from its use. The contents are the responsibility of the Maternal and Child Survival Program and do not necessarily reflect the views of WHO, USAID or the United States Government.

Requests for further information on this brief or permission to reproduce or translate this publication should be addressed to MCSP Communications, e-mail: info@mcsprogram.org. For further information on the WHO guidelines, please contact reproductivehealth@who.int or mncah@who.int.

© World Health Organization and Jhpiego 2015. All rights reserved. WHO/RHR/15.05.

WHO Department of Maternal, Newborn, Child and Adolescent Health
http://www.who.int/maternal_child_adolescent

WHO Department of Reproductive Health and Research
http://www.who.int/reproductivehealth
